# Mathematical modeling suggests 14-3-3 proteins modulate RAF paradoxical activation

**DOI:** 10.1371/journal.pcbi.1013297

**Published:** 2025-08-01

**Authors:** Gaurav Mendiratta, Kodye Abbott, Yao-Cheng Li, Jingting Yu, Peter Carlip, Melinda Tong, Jianfeng Huang, Maxim N. Shokhirev, Thomas McFall, Geoffrey M. Wahl, Edward C. Stites

**Affiliations:** 1 Integrative Biology Laboratory, Salk Institute for Biological Studies, La Jolla, California, United States of America; 2 Department of Laboratory Medicine, Yale School of Medicine, New Haven, Connecticut, United States of America; 3 Gene Expression Laboratory, Salk Institute for Biological Studies, La Jolla, California, United States of America; 4 The Razavi Newman Integrative Genomics and Bioinformatics Core (IGC), Salk Institute for Biological Studies, La Jolla, California, United States of America; 5 Department of Biochemistry and MCW Cancer Center, Medical College of Wisconsin, Milwaukee, Wisconsin, United States of America; 6 Yale Cancer Center, Yale School of Medicine, New Haven, Connecticut, United States of America; University of Virginia, UNITED STATES OF AMERICA

## Abstract

RAF inhibitor “paradoxical activation” (PA) is a phenomenon where RAF kinase inhibitors increase RAF kinase signaling. Through mathematical modeling and experimental data analysis, we recently demonstrated that the combination of conformational autoinhibition (CA) with the disruption of CA by RAF inhibitors plays an important role in PA. 14-3-3 proteins are known to modulate RAF CA and RAF dimerization. We here extend our mathematical model to include both roles of 14-3-3 proteins, and we derive rigorous analytical expressions of RAF signal regulation as modulated by 14-3-3 proteins. We then use the model to investigate how 14-3-3 proteins may modulate PA. We mathematically show 14-3-3 protein stabilization of the autoinhibited form of RAF should potentiate PA, while 14-3-3 protein stabilization of the active RAF dimer should reduce PA. Our analysis suggests that the net-effect will often be a potentiation of PA, and that 14-3-3 proteins may be capable of inducing PA for RAF inhibitors that normally show little to no PA. We test model-based insights experimentally with two different approaches: forced increases in 14-3-3 expression (which we find amplifies PA) and evolved resistance assays (which suggest increased 14-3-3 expression may contribute to resistance to RAF inhibitors). Overall, this work supports a role for 14-3-3 in modulating RAF-inhibitor mediated paradoxical activation.

## Author summary

RAF kinase inhibitors demonstrate a counterintuitive behavior: these small molecules promote inhibition of isolated RAF kinase proteins, but when applied to a cellular biochemical system these small molecules cause RAF kinase activation. This phenomenon is clinically important, as it contributes to off-target toxicity and to therapeutic resistance. Mathematical models have recently helped reveal the mechanisms that underly this “paradoxical activation” phenomenon. Additionally, mathematical modeling recently demonstrated that the regulation of RAF kinase activation through conformational changes plays a major role in the paradoxical activation of RAF. In the present study, we investigate how proteins that modulate the conformational changes alter the response to RAF inhibitors. Our mathematical modeling suggests 14-3-3 proteins, which bind and stabilize an inactive form of RAF, can increase the paradoxical activation effect, and we confirm this model-based hypothesis experimentally. Consideration of the mathematical modeling results led us to hypothesize that elevated levels of 14-3-3 protein expression may contribute to RAF inhibitor resistance. Our experiments further confirm that alterations in 14-3-3 expression modulate paradoxical activation, and our experimental observations support the hypothesis that elevated 14-3-3 protein expression may contribute to RAF inhibitor resistance. Altogether, this study demonstrates how biochemically detailed mathematical models can lead to discoveries relevant to clinical pharmacology.

## Introduction

**Fig 1:**
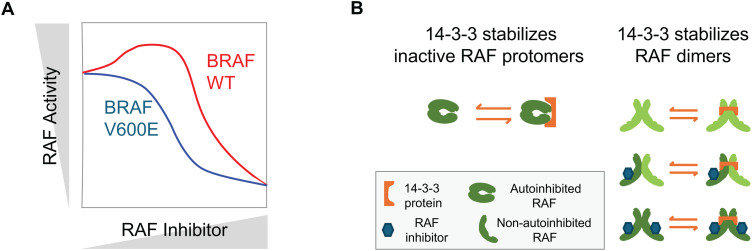
Model of the conformational autoinhibition stabilization (CAS) and dimer stabilization (DS) roles of 14-3-3 proteins on RAF activation. (A) Schematic of the Paradoxical Activation (PA) phenomenon. (B) Schematic of 14-3-3 interactions with RAF that may also influence PA. These interactions include stabilizing conformationally inactive RAF (CAS role) and stabilizing RAF dimers (DS role).

The RAF kinases (BRAF, CRAF, and ARAF) are key conduits of proliferation signals from the RAS GTPases (KRAS, NRAS, and HRAS) [1]. Cancers commonly harbor mutations in the genes coding for these proteins [2,3]. Many small molecule RAF inhibitors have been developed, and several have proven clinically useful for some BRAF V600 mutant cancers and have been FDA approved [4]. Surprisingly, it has been found that in other contexts RAF inhibitors can actually increase RAF signaling; this phenomenon is commonly referred to as “paradoxical activation” (PA) (Fig 1A) [5–8]. Not only does PA make RAF inhibitors ineffective for targeting RAS mutant cancers, PA also contributes to off-target effects when RAF inhibitors are used on BRAF V600E mutant cancers [9,10]. Thus, PA is a phenomenon that has multiple clinically significant implications. Despite numerous studies, the mechanisms driving PA are still not fully understood [11–13].

Mathematical models have previously proven useful for yielding new insights into PA [14–18]. Mathematical models that are based upon well-characterized reaction mechanisms have the potential to yield new insights into the response of a pathway to a pharmaceutical agent [19–23]. Additionally, such computational studies can reveal mechanisms of sensitivity and of resistance to treatment [24,25].

We recently performed a theoretical analysis that revealed PA results, in part, by RAF kinase conformational autoinhibition [16]. Conformational transitions are among the many steps involved in the complex regulation of RAF kinase activation [1]. The two key conformations of RAF kinases are the “autoinhibited” form, where an association between the kinase domain of RAF with its N-terminus positions RAF in a conformation that cannot dimerize [1,26], and the “non-autoinhibited form” where the kinase domain and the N-terminus are no longer associated and other regulatory steps, like dimerization and kinase domain conformational rearrangements, can occur [1,27]. Experimentalists had hypothesized that RAF inhibitor binding, which promotes a net transition out of the auto-inhibited state, may play a role in PA [28], and our theoretical analysis found that conformational autoinhibition was required to best explain experimental observations [16].

In this study, we investigate how 14-3-3 proteins, which are known to stabilize both the autoinhibited form of RAF and the dimerized form of RAF, might influence PA. We begin our consideration of 14-3-3 proteins and their possible influence on PA with a series of mathematical models. Our modeling suggests that 14-3-3 proteins can increase the range of conditions where PA occurs, suggesting drugs that normally have little to no PA may develop a pronounced PA response with increased 14-3-3 expression. We test this insight experimentally, and our experiments find elevated 14-3-3 expression can amplify PA for existing RAF inhibitors. Our experiments also find that elevated 14-3-3 expression can result in PA for newer RAF inhibitors that were developed to have little to no PA. Our studies of cancer cells treated for an extended period with RAF inhibitors find elevated expression of 14-3-3 emerges over time, suggesting a possible role for 14-3-3 proteins in emerged resistance. Overall, this work suggests 14-3-3 proteins are an important variable that modulates PA.

## Results

### Development of a RAF/14-3-3 model

In light of our previous work on RAF and paradoxical activation, which found that conformational autoinhibition plays an important role in paradoxical activation, we asked how proteins that stabilize the autoinhibited form of RAF might influence PA. 14-3-3 proteins are phosphoserine binding proteins that interact with a large number of proteins and contribute to a wide variety of cellular processes [29]. The RAF kinases are well-known binding partners for 14-3-3 [30]. 14-3-3 proteins bind and stabilize the autoinhibited form of BRAF (Fig 1B) [31–34]. However, 14-3-3 also stabilizes RAF dimers (Fig 1B) [32,33,35,36]. We therefore extended our model to include both of these sets of reactions, and we then mathematically investigated how they may impact PA.

**Fig 2:**
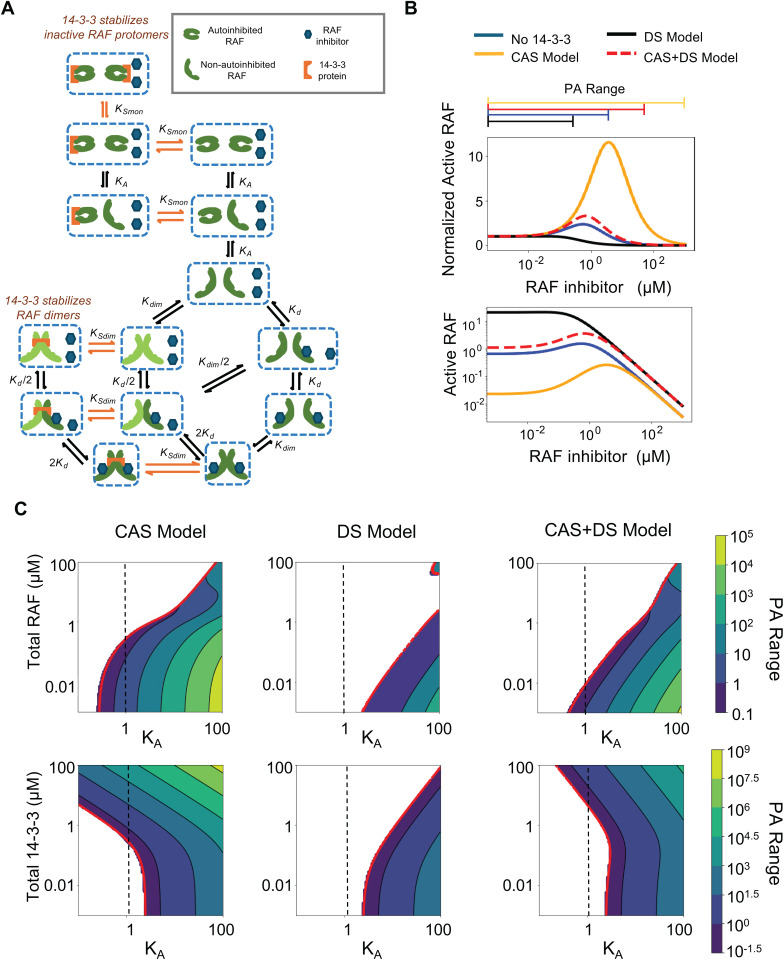
14-3-3 proteins promote paradoxical activation by stabilizing the autoinhibited form of RAF kinases. (**A**) Schematic of the modeled mechanism of 14-3-3 protein stabilization of the RAF autoinhibited form (CAS role) and the RAF dimer (DS role). (**B**) Predicted dose responses for the four different RAF activation models (no 14-3-3, CAS Model, DS Model, CAS + DS Model) to illuminate the consequences of 14-3-3 stabilizing (or not stabilizing) the autoinhibited form and/or the RAF dimer. Active RAF is shown normalized to the drug free level (above) and to the percent of total RAF (below) as a function of total inhibitor concentration. (**C**) Magnitudes of PA as a function of autoinhibition propensity (K_A_), RAF abundance, and 14-3-3 abundance are shown in different CAS, DS and CAS + DS models with 14-3-3 proteins. In this figure, the magnitude of PA is measured as the range of drug concentrations where the inhibitor causes activation above the baseline condition with no inhibitor. The contours represent the predicted concentration at which the drug becomes an inhibitor (PA Range).

We extended our previously developed model that described RAF dimerization, activation, and inhibition to include 14-3-3 proteins (Fig 2A). Within the previous model, RAF could adopt an autoinhibited conformation (which could neither dimerize nor bind drug) and a non-autoinhibited conformation (which could bind drug and/or dimerize) [27]. RAF bound to a drug was assumed to only be capable of transitioning to the autoinhibited state after dissociation of the bound drug. RAS-GTP was implicitly assumed to be required for wild-type RAF activation [1]. Active RAF was defined as the sum of all RAF protomers that were both within a RAF dimer and not bound to a RAF inhibitor. To this model, we added the protein 14-3-3, its binding to conformationally autoinhibited RAF, and its binding to dimerized RAF. Thus, the updated model now includes the two known roles for 14-3-3 in the regulation RAF signals: conformational autoinhibition stabilization (CAS), and dimer stabilization (DS).

We only consider 14-3-3 dimers in our model because it is dimeric 14-3-3 that is found bound with autoinhibited RAF and to dimerized RAF. Both the complex of a 14-3-3 dimer with autoinhibited RAF and the complex of a 14-3-3 dimer with dimerized RAF involve the 14-3-3 dimer binding to two different phosphorylated serine residues on RAF. For each of the 14-3-3 roles, our model only includes the 14-3-3 unbound and 14-3-3 doubly-bound states; this simplification is justified with a simpler model that does not include RAF inhibitor binding [37].

Closed-form, analytic expressions for the steady-state solution for this system were derived with the principal of detailed balance and total protein and drug conservation (Table 1, S1 Appendix) [15,38]. Analytic solutions for conditions with and without drug are obtained for the base model and for the models incorporating 14-3-3. These solutions provide global predictions for the possible behaviors of each considered system. The analytic solutions also provide parameter-value insensitive insights in the corresponding limits, and the solutions reveal how the different parameters of the system together influence model outputs. They also provide precise cross-checks for the generality of numerical simulations.

**Table 1:** Paradoxical activation (PA) of the RAS pathway and its analysis with a series of analytical mathematical models.

Model Type	Conformational Autoinhibition (CA)	Conformational Autoinhibition Stabilization with 14-3-3 (CAS)	Dimer Stabilization with 14-3-3 (DS)	Conformational Autoinhibition Stabilization and Dimer Stabilization (CAS + DS)
**Core Model Assumptions**	• Monomeric RAF can autoinhibit • Non-autoinhibited RAF can dimerize and bind drug • RAF dimers signal downstream	• CA model assumptions • 14-3-3 stabilizes RAF monomers	• CA model assumptions • 14-3-3 stabilizes RAF dimers	• CA model assumptions • 14-3-3 stabilizes RAF monomers • 14-3-3 stabilizes RAF dimers
**Baseline Active RAF (without drug)**	$\frac{{\left(\sqrt{1+E3}-1\right)}^{2}}{E3}$	$\frac{{\left(\sqrt{1+E5}-1\right)}^{2}}{E5}$	$\frac{{\left(\sqrt{1+E7}-1\right)}^{2}}{E7}$	$\frac{{\left(\sqrt{1+E10}-1\right)}^{2}}{E10}$
**Active RAF (relative to total RAF)**	$\frac{{\left(E1-\sqrt{E1^{2}+E2}\right)}^{2}}{E2(1+d_{rel})}$	$\frac{{\left(E4-\sqrt{E4^{2}+E2}\right)}^{2}}{E2(1+d_{rel})}$	$\frac{{\left(E1-\sqrt{E1^{2}+E6}\right)}^{2}}{E6(1+d_{rel})}$	$\frac{{\left(E9-\sqrt{E9^{2}+E8}\right)}^{2}}{E8(1+d_{rel})}$
**RAF Dimers**	$\frac{1+d_{rel}}{2}\times Active\;RAF$	$\frac{1+d_{rel}}{2}\times Active\;RAF$	$\frac{1+d_{rel}}{2}\times Active\;RAF$	$\frac{1+d_{rel}}{2}\times Active\;RAF$
**PA Conditions**	RAF_rel_ < (1 + 3K_A_)(K_A_-1)(1/8)	RAF_rel_ < (K_A_S_rel_ + K_A_-1)(3K_A_S_rel_ + 3K_A_ + 1)(1/8) [1]	RAF_rel_ < (1 + 3K_A_) (K_A_-1)/(8(1 + s_rel_)) [2]	K_A_> K_Smon_/(3(K_Smon_+s)) x $(1+2\sqrt{1+\frac{6RAF_{rel}(K_{Sdim}+s)}{K_{Sdim}}}$) [3]

Definitions and Footnotes:

$E1\;=1+K_{A}+d_{rel}$

$E2\;=8RAF_{rel}{\left(1+d_{rel}\right)}^{2}$

$E3\;=\frac{8RAF_{rel}}{{\left(1+K_{A}\right)}^{2}}$

$E4\;=E1+K_{A}\times S_{rel}$

$\begin{array}{c} E5\;=\frac{8\;RAF_{rel}\;}{\;\left(1+K_{A}+K_{A}s_{rel}\right)2\;} \end{array}$

$E6\;=E2\times (1+s_{rel})$

$E7\;=E3\times (1+s_{rel})$

E8 =E6

$E9\;=E1+K_{A}\times [s]/K_{Smon}$

$\begin{array}{c} E10\;=\frac{8\;RAF_{rel}\;}{\;\left(1+K_{A}+K_{A}[s]/K_{Smon}\right)2\;}\times \left(1+\frac{\left[s\right]}{K_{Sdim}}\right) \end{array}$

$s_{rel}=\frac{\left[Unbound\;14-3-3\;(s)\right]}{K_{Sdim}}$

$S_{rel}=\frac{\left[14-3-3\right]}{K_{Smon}}$

$RAF_{rel}=\frac{\left[RAF\right]}{K_{\dim}}$

$d_{rel}=\frac{\left[Unbound\;drug\;(d)\right]}{K_{d}}$

[1] in the limit when [14-3-3] available-to-bind-with-RAF >> [RAF]

[2] in the limit when ds/dd_rel_→0

[3] true when [14-3-3] >> [RAF]

Analytic conclusions in equation form for the conformational autoinhibition mechanism and associated 14-3-3 models. Core model assumptions, predicted expressions for active RAF at baseline (no drug), active RAF, total RAF dimers and PA conditions are shown in the rows for each of the autoinhibition mechanism and associated models. From left to right, the columns identify results within the base model of RAF activation, the conformational autoinhibition stabilization (CAS) role of 14-3-3, dimer stabilization (DS) role of 14-3-3 and the complete 14-3-3 model with CAS + DS roles. A key that presents the abbreviated expressions, which allow presentation into similar functional forms for corresponding model results, is appended to the bottom of the table.

### Computational evaluation of the dual roles of 14-3-3 on RAF signal regulation

Modeling both roles for 14-3-3 suggests that 14-3-3 proteins potentiate PA (Figs 2B and S1A, S1B). To evaluate whether this was due to stabilization of the autoinhibited form, due to stabilization of dimers, or due to both, we considered simplified models where the role of 14-3-3 was limited to only conformational autoinhibition stabilization (CAS) or was limited to only dimer stabilization (DS) (Table 1, S1 Appendix). Analytic results for baseline activity in the absence of drug provides an accurate and global cross-check for agreement between model assumptions and solutions. We find that when no drug is present, increasing 14-3-3 always (monotonically) reduces RAF signaling in the CAS model and always increases signaling in the DS model (Table 1: first row). Analytic expressions for the conditions on the parameters under which PA may occur are also shown in Table 1, last row. PA conditions in the base model show that with a sufficient bias in the equilibrium towards the inactive state of RAF (K_A_ > 1 and sufficiently low RAF levels) PA will be observed. In the CAS sub-model, the presence of 14-3-3 relaxes the constraints on PA thereby increasing the range of conditions where PA would be observed. Moreover, from the fact that the 14-3-3 concentration occurs additively in the constraint relationship we can conclude that this constraint can only be further relaxed through increasing 14-3-3 levels. In the presence of 14-3-3, the DS sub-model strengthens the constraints on PA, thereby reducing the range of conditions where PA would be observed. Further, the 14-3-3 concentration occurs in the denominator alone; therefore, this constraint can only become stricter with increasing 14-3-3 levels, which reduces the range of conditions where PA occurs. We also show numerically that the CAS-only case displayed PA for a wider range of conditions than the full model, and that the DS-only case displayed PA for a smaller range of conditions than the full model (Figs 2C and S1C–H). Additionally, we show that the PA was increased for the CAS-only case and was reduced for the DS-only case (Figs 2C and S1C–H). This was true whether PA was measured by the range of inhibitor conditions that result in increased RAF signaling or by the net increase in RAF signaling above the baseline signal that occurs in the absence of drug.

In a simplified form of the CAS + DS model where 14-3-3 binds autoinhibited and dimerized RAF equivalently, increases in 14-3-3 expression will typically result in an increased range of PA and an increased magnitude of PA (Figs 2C and S1H). However, the analysis also reveals that there are a smaller number of conditions where a limited range of increases in 14-3-3 expression would result in decreased PA range and decreased magnitude of PA. From any set of conditions, however, enhanced PA will result provided a sufficiently large increase in 14-3-3 expression occurs. The model also clarifies that this enhancement of PA is mediated by the contributions of the CAS mechanism. Transfection of 14-3-3 is likely to limit to this condition of high 14-3-3 levels and is therefore likely to potentiate PA, and the experiments we present later in this work involve 14-3-3 transfection.

Numerical analysis of the CAS + DS model was performed to evaluate conditions where the affinities of 14-3-3 for autoinhibited RAF monomer and for RAF dimer differ. This more global numerical analysis finds a complex, non-monotonic relationship between the expression level of 14-3-3 with RAF activation (S2A–C Fig), and between the expression level of 14-3-3 with PA range (S2D Fig). Altogether, these analytic and numeric results reveal that the dual activities of RAF that individually promote and inhibit RAF activation do not simply “cancel each other out” but rather have a complex net effect that has the potential to impact PA.

### Experiments: 14-3-3 overexpression amplifies paradoxical activation

Our analytical and numerical results both suggested increased 14-3-3 expression may amplify PA, and that increased 14-3-3 expression could introduce PA to a system where it would otherwise not have been present. We reasoned that the artificial modulation of 14-3-3 expression levels would be a reasonable approach for testing these mathematical model-based insights.

**Fig 3:**
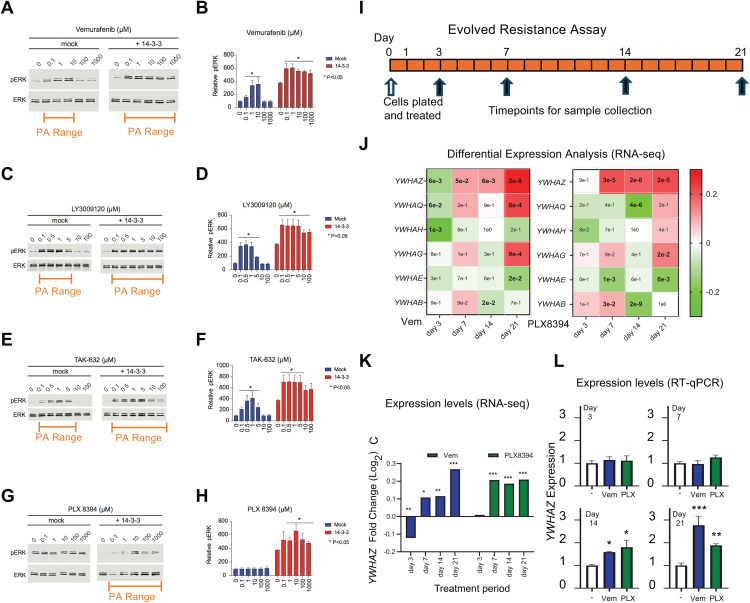
Increased 14-3-3 expression can potentiate paradoxical activation, dimerization, and resistance to RAF inhibitors. (A, C, E, G) Immunoblots of 14-3-3 and mock transfected SK-MEL-2 cells that were treated with increasing doses of vemurafenib, LY3009120, TAK-632, or PLX8394, for a period of two hours. Results are representative of three independent experiments. (B, D, F, H) Densitometry-based quantification of the ratio of phosphorylated ERK to total ERK for the indicated doses of the indicated RAF inhibitor from three independent assays in SK-MEL-2 cells. The quantified data are means ± SD. One-way ANOVA followed by post-hoc Tukey’s test for multiple comparisons was performed to evaluate differences between drug and vehicle treatments. (I) Schematic portraying the experiment where SK-MEL-2 cells were treated for 21 days with vemurafenib, PLX8394, or DMSO. Samples were saved for gene expression measurements at the specified timepoints (black arrows). (J) RNA-seq was performed on the SK-MEL-2 lysates from the indicated time points to evaluate changes in the expression of 14-3-3 family genes. The inset numbers represent adjusted p-value for differential expression of each gene in treated samples relative to respective DMSO control samples. The color chart shows log2 fold change. Data are aggregate from three independent experiments. (K) Fold change in *YWHAZ* expression after treatment with vemurafenib and with PLX8394. (L) Relative expression levels measured by RT-qPCR of *YWHAZ* for vemurafenib and PLX8394 treated cells relative to DMSO treated cells at the same time points, as measured by RT-qPCR. p-values were calculated using comparative Ct method. P-values: * represents a value less than 0.05, ** represents <0.01, *** represents <0.001. Data are mean and SD from three independent experiments.

Among the seven 14-3-3 encoding genes, we choose *YWHAZ* (which codes for 14-3-3ζ protein) for experimental analysis based on the previous literature [39–42]. We transfected a *YWHAZ* expression construct into NRAS Q61R mutant SK-MEL-2 cells and observed elevated basal signaling, i.e., increased ERK phosphorylation, possibly reflecting the promotion of RAF dimers by 14-3-3 (S3A Fig). We then performed RAF inhibitor dose responses for 14-3-3 ζ and mock transfected cells using the RAF inhibitor vemurafenib (Fig 3A,3B). We used phosphorylated ERK as a downstream readout for elevated RAF kinase signaling; we note phosphorylated ERK has commonly been used as a readout of RAF signaling in studies of PA, and that when both phosphorylated MEK and phosphorylated ERK have been utilized in experimental studies of PA these observables have been qualitatively similar [5–7,11]. We observed PA for both SK-MEL-2 cells transfected to express additional, exogenous, 14-3-3 ζ and that were mock transfected. In other words, vemurafenib was observed to cause an increase in ERK phosphorylation above the baseline level observed with no vemurafenib (PA). The cells transfected to express additional 14-3-3 ζ displayed significant widening of the PA response compared to the mock transfected cells, i.e., the range of vemurafenib doses that showed elevated ERK phosphorylation above baseline was larger in 14-3-3 ζ transfected cells than in the mock transfected cells. Similar results were observed in an additional RAS mutant cell line, demonstrating the generality of our results (S3A–C Fig). To evaluate the role of 14-3-3 ζ expression levels upon inhibitor induced dimerization, we utilized a recombinase enhanced luciferase complementation assay (ReBiL) [43,44] (S3D–F Fig). We observed a pattern of increased RAF dimerization with increased RAF inhibitor that was potentiated by increased 14-3-3 ζ expression (S3G, S3H Fig).

We next performed similar experiments for the third generation RAF inhibitors LY3009120, TAK-632, and PLX8394. These drugs were all developed to display less paradoxical activation than the previous generation RAF inhibitors [13,45,46]. However, descriptions of the development of these drugs do not discuss a consideration of 14-3-3 expression as a possible modulator of PA. For all three of these inhibitors, 14-3-3 ζ overexpression resulted in a widening of the range of drug concentrations that show PA (Fig 3C-H). Additionally, the ReBiL assay revealed increased RAF dimerization with both increased 14-3-3 ζ expression and the introduction of LY3009120 and TAK-632 (S3G,S3H Fig). Thus, we find that elevated 14-3-3 expression can cause a higher degree of the PA phenomenon for third generation RAF inhibitors. Notably, the third generation RAF inhibitor PLX-8394 typically shows essentially no PA [46], but we found that an increase in 14-3-3 expression can result in some PA for this inhibitor.

### Experiments: Increased 14-3-3 expression promotes resistance of RAS mutant cells to RAF inhibitors

We considered that reactivation of RAS/RAF signaling can promote resistance to RAF inhibitors [47,48]. Our modeling suggested that elevated 14-3-3 expression could expand the conditions where a RAF inhibitor results in RAF activation, and our experiments confirmed this hypothesis. We therefore hypothesized that there might therefore be a role for elevated 14-3-3 expression in the promotion of resistance to RAF inhibitors.

To investigate whether 14-3-3 expression levels in a population of cells may increase to promote acquired resistance to treatment, we performed an evolved resistance assay [49] where we propagated SK-MEL-2 cells in media supplemented with PLX8394 or vemurafenib for a period of 21 days (Fig 3I). We measured gene expression in cell lysates collected over the course of 21 days by RNA-seq. We focused on expression levels of the 14-3-3 family of genes. Notably, a statistically significant trend toward increased expression was observed for *YWHAZ* in both the PLX8394 and vemurafenib treated cells (Fig 3J,3K). No consistent trend was observed for the other genes that encode 14-3-3 family proteins (Fig 3J). We performed quantitative RT-qPCR to measure *YWHAZ* transcript levels within the same lysates (Fig 3L). By day seven and through the remaining experiment, these measurements also detected a reproducible and statistically significant increase in *YWHAZ* transcript for both vemurafenib and PLX8394 treated cells, thus confirming the observation from the RNA-seq data.

### Reconciliation of experimental data with the mathematical model

The two roles of 14-3-3 on RAF signaling, CAS and DS, were individually found to have different impacts on PA. For example, the CAS-only model is consistent with the experimental observation that elevated 14-3-3 expression increases PA (Fig 3). In contrast, the DS only model would suggest the opposite. Thus, the experimental observation of a wider range of RAF inhibitor concentrations that result in elevated RAF signaling after 14-3-3 overexpression suggests the CAS role of 14-3-3 may be stronger than the DS role.

However, we also note that that the overexpression of 14-3-3 resulted in increased ERK phosphorylation at baseline (S3A Fig). This is consistent with the DS-only model, where increased 14-3-3 expression would increase basal signaling (Figs 2 and S1 Fig, Table 1 ). In contrast, elevated 14-3-3 expression would result in decreased basal signaling for the CAS-only model. Thus, the experimental observation of elevated baseline signaling after 14-3-3 overexpression suggests the DS role of 14-3-3 may be stronger than the CAS role, the opposite of what was inferred from the observed increase in PA.

We therefore considered the full model with both CAS and DS present to reconcile the experimental data. Within the full CAS and DS model, there is an effective affinity for 14-3-3 binding to the autoinhibited form of RAF and an effective affinity for 14-3-3 binding to the RAF dimer. We considered different measures of RAF signaling under a wide range (four orders of magnitude) of relative affinities for these two different 14-3-3/RAF interactions (S2 Fig). With respect to elevated basal signaling with elevated 14-3-3 expression (S2A Fig), we find that this follows from the combined CAS + DS model for a wide range of conditions, from a strong favoring of DS to a slight favoring of CAS. With respect to increased PA range (S2D Fig), we find that this behavior also follows from the combined CAS + DS model for a wide range of conditions, but now ranging from a strong favoring of CAS to a slight favoring of DS. Notably, these two ranges overlap when the two affinities are reasonably similar (~2-3x different or less). Thus, our experimental data and mathematical modeling suggest that 14-3-3 proteins interact with RAF monomers and dimers with similar affinity. This analysis also reveals that which of CAS and DS dominates can differ between experimental observations.

## Discussion

Our mathematical analysis suggests that 14-3-3 proteins, through their effect in promoting the conformationally autoinhibited form of RAF, can amplify the PA phenomenon. Our rigorous analytical and numerical conclusions are supported by experiments where we observe 14-3-3 proteins amplifying PA in three different cell lines, and where we detect PA directly with three different physiological read-outs (intracellular phosphorylation, proliferation, and dimerization).

Our analysis considers the steady-state behavior of a simplified portrayal of RAF signaling. However, we note that our experiments are performed at a two-hour time point and not at steady-state. This is because the ultimate steady-state of the RAF pathway will be impacted by slowly acting feedback loops that exert their impact over the timescale of multiple days [50]. These feedback loops are not included in our model; therefore, our model is better thought of as describing the asymptotic behavior before the effects of the slowly activating feedback loops exert their influence. Experimental measurements of PA via phosphorylation immunoblots are commonly done at one to four hour time points [5–7,11], before the slowly acting feedback processes have a large impact. Thus, our steady-state model and other published steady-state models of PA [15,16] are believed to describe the reproducible and stable PA phenomenon regularly observed soon after treatment. Other models have investigated the impact of RAF inhibition on the feedback processes and the process of finding a new steady state [14], such as is typically found after two or three days of RAS/RAF pathway treatment [51]. The combination of these models could potentially provide additional insights into the long-term effects of RAF inhibitor treatment in future work.

Our use of downstream ERK phosphorylation as a readout of RAF kinase activity may introduce a saturable readout of RAF kinase activity, potentially explaining why we can robustly see increases in PA range (which only requires a monotonic relationship between RAF kinase activity and ERK phosphorylation) but not PA fold change (for which the downstream observable would need to be linear as a function of RAF kinase activity for it to be directly measurable). The ability to detect an increase in the range of drug concentrations that display PA would not be limited by saturation and would only require a monotonic relationship between RAF kinase activity and ERK phosphorylation, which seems reasonable to assume.

Although we have experimental evidence that supports our theoretical insights, a more comprehensive experimental exploration of 14-3-3 proteins and PA would be valuable. For example, which of the seven different 14-3-3 proteins are capable of potentiating PA when overexpressed? Which reduce PA when knocked-out and/or knocked-down? Knock-out and knock-down experiments of individual 14-3-3 proteins may not be able to reveal a difference if multiple 14-3-3 proteins can perform CAS and DS roles, but knockdown of multiple 14-3-3 proteins may be possible. These experiments will be further challenged by the pleiotropic roles of 14-3-3 proteins beyond those on the RAF kinases. The role of 14-3-3 in PA could also be investigated with experiments that mutate the phosphorylated serine residues that are important for 14-3-3 CAS and 14-3-3 DS roles. As both CAS and DS involve the same C-terminal serine residues (BRAF S729, ARAF S582, CRAF S621), mutation of these residue would convert the system to the no-14-3-3 modeled system, and mutation of the serine residue only involved in CAS (BRAF S365, ARAF S214, CRAF S269) [52,53] may allow for isolation of the DS role for comparisons with DS-only model predictions. For these mutations, it may be necessary to knock-out the wild-type versions of BRAF, CRAF, and ARAF to detect a signal in addition to introducing the mutant form of the RAF kinase(s) being utilized in the experiment. Moreover, the 14-3-3 types and expression levels may contribute to the differences in PA response across cell-types [52]. 14-3-3 proteins are promiscuous and pleiotropic, so it is possible that part of the effects observed with 14-3-3 transfection follow from other activities of 14-3-3 proteins; it is not possible to rule-out unknown alternative mechanisms involving 14-3-3 proteins.

The treatment of BRAF mutant tumors can be limited by off-target effects in non-BRAF mutant cells, including in cells that harbor a RAS mutation [9,10]. The third generation RAF inhibitors developed to have little to no PA would be anticipated to have less dose-limiting toxicity that follows form PA in BRAF WT cells. Our finding that elevated 14-3-3 could lead to PA in these third generation inhibitors, including PLX8394 led us to hypothesize that elevated 14-3-3 expression may be important in these conditions. Thus, our experiments utilized NRAS mutant SK-MEL-2 cells. We found that elevated 14-3-3 expression contributed to evolved resistance in both PLX8394 and vemurafenib treated cells. Our experiments did not evaluate RAF inhibitor resistance in BRAF mutant cells. Altogether, we show computational and experimental evidence to support that amplified expression of 14-3-3 proteins may emerge as a mechanism of toxicity and/or resistance should third generation RAF inhibitors make it to clinical use. If found to be true, this would suggest that effective inhibition of RAF kinases may ultimately require allosteric inhibitors that do not bind the ATP-binding pocket.

Our proposed model does not explicitly include every known regulatory step of RAF kinase signaling, nor does it explicitly include all RAF binding partners. For example, we do not explicitly include RAF binding to RAS GTPases, nor do we explicitly include RAF binding to MEK kinases. Our model could be extended to include these, and other, binding partners and regulatory reactions. However, exact analytic solutions would no longer be obtainable as the model becomes more complex. We consider our simple, analytic model significant in that it demonstrates how a few known steps in RAF signal regulation are sufficient to generate PA through a non-intuitive mechanism. Our analytic solutions also reveal how specific parameters of the model influence PA (Table 1). Future work will extend the model to include additional reactions and binding partners. Numerical analyses of the extended model could reveal how these other binding partners and reactions modulate PA. Importantly, it is also possible that an extended model would identify other processes that contribute to PA and/or find larger (or smaller) contributions from conformational autoinhibition. Nevertheless, analytic modeling led to novel hypotheses and new experimental results that reveal new aspects of the PA phenomenon, demonstrating a practical benefit of these modeling efforts.

Three major classes of BRAF kinase mutations have been described [54]. The different classes are defined by their mechanism of pathogenic activation, and these differences are manifest in the biochemical parameters for these altered reactions that result in elevated levels of net RAF kinase signaling. We have previously demonstrated that mathematical models can be a useful tool for identifying pathogenic mutant-specific behaviors, including atypical responses to treatment [19,20,22,49,55]. Our approach requires explicit incorporation of the biochemical processes that are altered as a consequence of mutation [19,56–58]. Notably, we have found that a wide variety of insights about mutant protein signaling can be obtained with limited knowledge of specific parameters [22,49,55,59–61]. The model, presented in this manuscript, that includes RAF dimerization, RAF inhibitor interactions, RAF conformational changes, and 14-3-3 binding has demonstrated utility for driving translationally relevant discoveries and will provide a foundation for an extended model that characterizes the different classes of RAF mutations.

## Materials and methods

### Mathematical models and analysis

We developed a series of mathematical models that include conformational autoinhibition (CA), 14-3-3 stabilization of autoinhibited RAF, and 14-3-3 stabilization of dimerized RAF. For each model, we derived analytic equations that provide the equilibrium solution for the system (Table 1). These analytic expressions define the generality of a result and identify specific criteria required for PA to occur. Conclusions from most of our models are presented in theorem-proof form in the S1 Appendix. In addition to this ‘analytical modeling,’ we also perform ‘numerical modeling’ by using specific parameter values to create representative figures to illuminate model-based insights.

We focus on steady-state levels of the different states in which RAF can exist, as portrayed in the diagrams for each model. Between any two states an equilibrium relationship can be expressed as the ratio of abundances in the two states. Conservation of total protein quantities and zero value of total Gibbs free energy change at equilibrium both provide mechanisms to algebraically combine these expressions. We thereby derive expressions that relate the relative abundance of the RAF within its different monomeric and dimeric states. We perform algebraic manipulations and derive analytic solutions which we cross-check using Mathematica software v 12.0 (Wolfram Research). We perform numerical evaluations of these relationships and generate plots of these equations using Python packages including numpy, scipy and matplotlib.

To derive analytical results (Table 1) we assume that levels of unbound 14-3-3 are larger than the levels of unbound RAF, which is consistent with 14-3-3 protein levels being in excess to levels of RAF. Where applicable, parameters not varied in descriptive numerical plots are set as follows.

*K*_*A*_ = 10.0, *K*_*d*_ = 0.1 *µM*, *K*_*dim*_ = 0.1 *µM*, [RAF] = 0.04 *µM*, *K*_*Smon*_ = 0.2 *µM*, *K*_*Sdim*_ = 0.02 *µM*, [14-3-3] = 1.0 *µM*. Numerical simulations do not make any assumptions about the relative levels of 14-3-3 and RAF. Simulations which vary both these levels independent of each other are included in the main and supplementary figures.

We utilize mathematical models to clarify specific mechanisms that contribute to RAF activation and drug response. The models were driven by specific hypotheses that cannot be generalized to include all the features of RAF activation simultaneously due to limitations of available experimental data which does not cover all the required conditions and variations thereof. Moreover, our analytic approach allows a parameter-independent prediction for components of the canonical RAF activation cycle that would not be accessible if we attempted to include every potential feature independent of a focused hypothesis. This is similar to differences in complexity between in-vitro and in-vivo systems – the former often forms a more versatile and precise method of establishing mechanisms while not including most of complexities present in-vivo, thereby providing a precise testing ground for focused hypotheses.

### Cell culture and transfection

SK-MEL-2 cells were purchased from American Type Culture Collection (ATCC). SW48 cells with the G13D genotype were obtained from Horizon Discovery. The DNA expression plasmid used for 14-3-3, 1481 pcDNA3 flag HA 14-3-3 ζ, was a gift from William Sellers (Addgene plasmid # 9002; http://n2t.net/addgene:9002; RRID:Addgene_9002). Cells were grown in EMEM (SK-MEL-2) or RPMI (SW48) containing 10% fetal bovine serum (FBS), penicillin (100 U/ml), streptomycin (100 µg/ml), and l-glutamine (2 mM). Cells were cultured in 10 cm adherent culture dishes (VWR) and incubated at 37°C in 5% CO2. At time point zero, cell media was changed to the cell line’s respective media containing 10% fetal bovine serum (FBS) devoid of antibiotics. 24 hours later cells were transfected with empty packaged lipofectamine, or 5ug of 14-3-3 ζ expression plasmid DNA utilizing lipofectamine 2000 (Thermo Fisher Scientific) following manufacturers protocol. Cells were incubated for 24 hours and then were treated with RAF inhibitors at increasing doses for 2 hours. Cells were then prepared for Western blot analysis. All drugs were suspended and stored in DMSO, and all drug treatment groups carried the same amount of vehicle (DMSO).

### Western blotting

Cell lysates were generated using radioimmunoprecipitation assay buffer [150 mM NaCl, 1% NP-40, 0.5% sodium deoxycholate, 0.1% SDS, 50 mM tris (pH 8.0)] containing protease and phosphatase inhibitor cocktail (Cell Signaling Technology) and incubated on ice for 1 hour, vortexing every five minutes. The total protein concentration was determined by Pierce Protein assay (Thermo Fisher Scientific). Protein samples (5 µg) were resolved by electrophoresis on 12% SDS–polyacrylamide gels and electrophoretically transferred to polyvinylidene difluoride (PVDF) membranes (Millipore Corporation) for 20 min at 25 V. The blots were probed with the mouse anti-phospho-ERK antibody (675502, Biolegend) and rat anti-total-ERK antibody (686902) overnight at 4 degrees Celsius. Blots were washed and probed with goat-anti-mouse Dylight 800 secondary antibody and goat-anti-rabbit AlexaFlour 680 antibody for 1 hour at room temperature. The protein bands were visualized using the Licor CLx Odyssey imaging station (Licor Biosystems). Comparative changes were measured with Licor Image Studio software. One-way ANOVA followed by post-hoc Tukey’s test for multiple comparisons was performed to evaluate differences between drug and vehicle treatments.

### RAF inhibitor resistance assay

SK-MEL-2 cells were grown in EMEM (HyClone) supplemented with 10% fetal bovine serum (Atlanta Biologicals) and 1% Penicillin-Streptomycin (Gibco). The cells were treated with the vehicle (DMSO), PLX8394 (10µM), or Vemurafenib (10 µM). Every 72 hours, the cells were split to approximately 35% confluency and supplied with fresh media and treatment. Total RNA from SK-MEL-2 human melanoma cells was harvested for RT-qPCR and RNA sequencing on days 0, 3, 7, 14, and 21 using the E.Z.N.A Total RNA Kit I (Omega Bio-Tek). The entire experiment was performed with three independent replicates, each beginning on a different day. The quality and quantity of the total RNA was assessed using NanoVueplus Spectrophotometer (GE Healthcare) in sample preparation, and more precisely evaluated prior to sequencing using Agilent TapeStation 4200. RNA-Seq libraries were prepared with 500 ng total RNA using the TruSeq stranded mRNA Sample Preparation Kit according to the manufacturer’s protocol (Illumina). RNA-seq libraries were multiplexed, normalized and pooled for sequencing. The libraries were sequenced on the HiSeq 4000 system (Illumina) at single read 50 bp. Image analysis and base calling were done with Illumina CASAVA-1.8.2. on HiSeq 4000 system and sequenced reads were quality-tested using FASTQC. The GEO accession number for the RNAseq data is GSE179932. Reverse transcription was performed with the iScript Supermix cDNA Synthesis Kit (Bio-Rad). Quantitative polymerase chain reaction was performed by using the PerfeCTa SYBR Green FastMix (Quanta BioSciences) and CFX96 Touch Real-Time PCR Detection System (Bio-Rad). Transcripts were amplified using gene-specific primers for YWHAZ (F: 5’-CAACAAGCATACCAAGAAG-3’; R: 5’-TCATAATAGAACACAGAGAAGT-3’) and GAPDH (F: 5’-ACCACAGTCCATGCCATCAC-3’; R: 5’-GCTTCACCACCTTCTTGATG-3’). The comparative Ct method was used for relative quantification for gene expression data analysis in the quantitative RT-qPCR analysis.

### RNA-Seq analysis

Sequenced reads were quality-tested using FASTQC (v0.11.8) [62] and mapped to the hg19 human genome using the STAR aligner (v2.5.3a) [63] with default parameters. Raw or TPM (Transcripts Per Kilobase Million) gene expression was quantified across all the exons of RefSeq genes with analyzeRepeats.pl in HOMER (v4.11.1) [64], which used the top-expressed isoform as proxy for gene expression. Differential gene expression was performed with the raw gene counts using the R package, DESeq2 (v1.24.0) [65], using replicates to compute within-group dispersion. Differentially expressed genes were defined as having a false discovery rate (FDR) <0.05 when comparing two experimental conditions.

### ReBiL assay to measure RAF dimerization

The full-length human BRAF and CRAF were cloned to pLi635 ReBiL targeting plasmid [44] by Gibson assembly (NEBuilder HiFi E2621L) resulting in pLi814 (nl-BRAF cl-CRAF), which was integrated to U2OS 134–8 HyTK8 via Cre-recombinase mediated cassette exchange (RMCE) [43] resulting in U2OS 134–814 ReBiL cell line. U2OS 134–814 ReBiL cells cultured in T25 flask at 37 °C 7% CO_2_ incubator with 6 ml DMEM (Corning; 10–013-CV) with 10% (vol/vol) FBS, and 10 μg/mL ciprofloxacin (Corning; 61–277-RG) were transfected with 7.5 µg pcDNA3 or pcDNA3_flag_HA_14-3-3 ζ by Lipofectamine 3000 (Thermo Fisher Scientific) and incubated overnight. Approximately 5,000 transfected ReBiL cells were seeded into two 384-well plates and treated with 100 ng/ml doxycycline to induce nluc-BRAF and cluc-CRAF expression for 24 hours. RAF inhibitors were added at indicated concentrations and incubated for 2 hours. One-Glo (Promega E6120) was added to one plate and CellTiter-Glo 2.0 (Promega G9242) was added to another plate. Four technical replicates were performed on the same plate, with all treatments and measurements performed in parallel. Two independent biological replicates were performed. Luminescent signals were measured by Tecan M200 microplate reader (integration time 0.5 sec at 26 °C). Significance analysis was performed assuming normally distributed variation with a two sided t-test.

## Supporting information

S1 Fig PA fold change predicted in 14-3-3 models. (A) Plots portraying predicted drug dose response curves, with active RAF protomers as the readout. Multiple levels of 14-3-3 expression in the CAS + DS model are shown. Active RAF is shown normalized to the drug free level (above) and to the percent of total RAF (below) for the same conditions as Fig 2B. (B) Plots portraying how 14-3-3 proteins would influence total RAF dimers when only the CAS mechanism, only the DS mechanism, or both CAS and DS mechanisms are considered. The RAF dimers are normalized to drug free level (above) and percent of total RAF (below) for the same conditions as Figs 2B and S1A. (C-H) Contour plots with total RAF concentration (C,E,G) and total 14-3-3 concentration (D,F,H) on the y-axis, with the intrinsic autoinhibition parameter K_A_ on the x-axis, and with the predicted maximum fold change in RAF activation as a measure of PA. C,D: CAS-only model; E,F: DS-only model; G,H: CAS + DS model.
(EPS)

S2 Fig Further predictions delineating CAS and DS roles of 14-3-3. For all panels, the x-axis displays for increasing levels of 14-3-3 expression (from 1nM to 100 µM), and the y-axis displays varying degrees of the relative strength of DS and CAS mechanisms. This is specified as a meta-parameter α which is multiplied to the dissociation of 14-3-3 interaction with RAF dimers (*K*_*Sdim*_ = α × *K*_*Sdim*_) and divided from the dissociation constant of 14-3-3 interaction with inactive RAF monomers (*K*_*Smon*_ = *K*_*Smon*_/α). A small α favors strong DS and weak CAS, and a large α favors weak DS and strong CAS role. (A) Contour plot presenting baseline RAF activity when no drug is present, with RAF activity presented as the percent of total RAF in an active state. (B) Contour plot presenting maximum RAF activity obtained across all RAF inhibitor concentrations, presented as a percent of total RAF in an active state. (C) Contour plot presenting the PA fold change (peak RAF activity when drug is present, divided by the baseline level of RAF activity when no drug is present). (D) Contour plot presenting the PA range (range of drug concentrations that lead to a level of RAF activity that is higher than the baseline level of RAF activity found when no drug is present).
(EPS)

S3 Fig Experimental controls of 14-3-3 on RAF dimerization and inhibition. (A) Representative immunoblots of SK-MEL-2 and SW48 cells transfected with flag-tagged 14-3-3 ζ or mock transfected that were inputs for the different RAF inhibitor experiments in Fig 3. Immunoblotting for 14-3-3 utilized an anti-flag antibody. Results are representative of three independent experiments. (B) Immunoblots of 14-3-3 and mock transfected KRAS G13D isogenic SW48 cells that were treated with increasing doses of vemurafenib for a period of two hours. Results are representative of three independent experiments. (C) Densitometry-based quantification of the ratio of phosphorylated ERK to total ERK for the indicated doses of the indicated RAF inhibitor from three independent assays in SW48 cells. The quantified data are means ± SD. One-way ANOVA followed by post-hoc Tukey’s test for multiple comparisons was performed to evaluate differences between drug and vehicle treatments. (D) Schematic of the ReBiL assay and its application in our experiments. (E) Baseline toxicity in ReBiL cells evaluated via proliferation assay. The nluc-BRAF, cluc-CRAF, stable, U20S cell clones had proliferation measured with CellTiter Glo under mock and 14-3-3 transfected conditions after 2 hours of drug exposure. Samples A, B, C, and D were the stocks used for the study of vemurafenib, LY3009120, TAK-632, and PLX8394, respectively in the ReBiL assay. (F) Relative changes in proliferation upon treatment with RAF inhibitor for the mock transfected and 14-3-3 transfected cells. (G) Luminescent signals from ReBiL assay, nluc-BRAF, cluc-CRAF U2OS cells treated with the indicated doses of the indicated RAF inhibitors. The marked stars show significant enhancement relative to DMSO baseline. (H) Assessment of inhibitor induced RAF dimerization by split-luciferase complementation via the ReBiL assay for both mock and 14-3-3 transfected conditions. Paired t-tests were used to evaluate statistical significance. Data shown are mean values from four technical replicates. Panels E-H show mean and ± SD for four technical replicates in each condition and are representative of two independent experimental replicates. P-value scheme: * represents a value less than 0.05, ** represents <0.01, *** represents <0.001.
(EPS)

S1 Appendix Extended description and derivation of the conformational autoinhibition models. Document detailing the development and analysis of the new models presented in this manuscript.
(PDF)

S1 Data Mendiratta RAF 14-3-3.zip. Supplementary files that include the code required to analyze and evaluate the models and to reproduce all of the results presented in this study.
(ZIP)
