# Supplementary material for: Mathematical modeling suggests 14-3-3 proteins modulate RAF paradoxical activation
Source: PLoS Comput Biol. 2025 Aug 1;21(8):e1013297. doi: 10.1371/journal.pcbi.1013297 (PMC12407542; doi:10.1371/journal.pcbi.1013297)
Supplement: S1 Appendix — Document detailing the development and analysis of the new models presented in this manuscript. (PDF) [file pcbi.1013297.s004.pdf]

Appendix : In-Silico modeling of 14-3-3 and RAF interactions

In a previous work we showed that paradoxical activation (PA) is possible in the presence of ATP competitive drugs binding protomers within a dimer of any inducible kinase [1]. Both the inactive RAF (non drug binding state), as well as the RAF dimer state play an important role in the PA mechanism described therein. 14-3-3 proteins bind with RAF and play an important scaffoling role in both states. Here we extend the ordinary differential equation model of RAF from the previous work to include 14-3-3 and RAF interactions. We detail the conformational autoinhibition stabilization (Section 1) and dimer stabilization roles (Section 2) and combine these into a unified model of RAF activation (Section 3). We show the resulting analytical expressions in table 1 and the derivations of which are repreoduced in Mathematica code present in the supplementary code. In section 4 we provide numerical illustrations of the mathematical models used in our work comparing drug dose response of different states of RAF included in each model.

1 Conformational autoinhibition stabilization potentiates PA and reduces baseline signaling (CAS model)

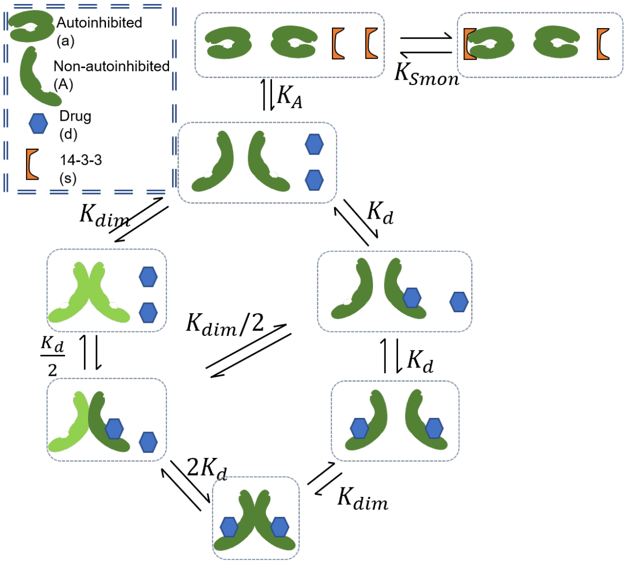

14-3-3 protein dimerize and bind to the inactive state of RAF to stabilize it in a inactivated complex [2, 3]. We previously showed that an inactivated state can add a non-trivial behavior to the dynamics that was absent before [1]. In this section, we show that stabilization of this state has the

potential to induce further PA in response to the drug and expand the conditions under which such phenomena can occur. To study the role of conformational auto-inhibited state stabilization, we define a model of 14-3-3 interaction with inactive RAF and solve the corresponding model shown above. In this section we derive conclusions of the CAS model which is analytically solvable in the limit of high concentration of 14-3-3 or a large dissociation constant $K_{Smon} >> RAF$, where the 14-3-3 binding with inactive RAF monomeric state is related by the following simplified expression.

$$[as] = S_{rel} \times [a] \tag{1}$$

where, $S_{rel} = STOT/K_{Smon}$. The following reactions represent the network in figure above.

$$\begin{aligned} a &\rightleftharpoons A & (2)\\ A + d &\rightleftharpoons Ad & (3)\\ A + A &\leftrightharpoons AA & (4)\\ AA + d &\rightleftharpoons AAd & (5)\\ A + Ad &\leftrightharpoons AAd & (6)\\ AAd + d &\rightleftharpoons AdAd & (7)\\ Ad + Ad &\leftrightharpoons AdAd & (8) \end{aligned}$$

The following expressions correspond to the above reactions.

$$K_{a\to A} = K_A = \frac{[a]}{[A]} \tag{9}$$

$$[Ad] = \frac{[A][d]}{K_d} \tag{10}$$

$$[AA] = \frac{[A]^2}{K_{dim}} \tag{11}$$

$$\begin{aligned} [AAd] &= 2\frac{[AA][d]}{K_d} \\ &= 2\frac{[A]^2[d]}{K_{dim}K_d} & (12) \end{aligned}$$

$$\begin{aligned} [AdAd] &= \frac{[AAd][d]}{2K_d} \\ &= \frac{[A]^2[d]^2}{K_{dim}K_d^2} & (13) \end{aligned}$$

At equilibrium, equations 10-13 and equation 9 apply in the CAS model with the addition of the RAF monomer and 14-3-3 equilibrium represented in equation 1. Over the reaction periods, we assume that the total RAF and total drug concentrations remain unchanged leading to the conservation equations as below.

$$RAF = a + as + A + Ad + 2(AA + AAd + AdAd) \tag{14}$$

Since the drug does not bind with the autoinhibited conformation of the kinase, the total drug equals a sum of unbound, protomer bound and dimer bound drug 15.

$$[d] + [Ad] + [AAd] + 2[AdAd] = Drug \tag{15}$$

Following the steps described previously [1, 4], we analytically solve for the equilibrium concentrations in the CAS model and find the active kinase in proportion to total RAF.

$$\frac{ActiveRAF}{RAF} = ([AAd] + 2[AA])/RAF \tag{16}$$

$$= 2(1 + d_{rel}) RAF_{rel} \left(\frac{[A]}{RAF}\right)^2 \tag{17}$$

$$= \frac{\left(\sqrt{E4^2 + E2} - E4\right)^2}{E2 \times (1 + d_{rel})} \tag{18}$$

where, $E4 = (1 + d_{rel} + K_A(1 + S_{rel}))$ and $E2 = 8(1 + d_{rel})^2 RAF_{rel}$, $d_{rel} = [d]/K_d$, S_{rel} =[14-3-3]$/K_{Smon}$ and $RAF_{rel} = RAF/K_{dim}$. To understand the impact on paradoxical activation we calculate the zeroes of first derivative and signature of second derivative of active RAF, as a function of d_{rel}. The following conditions, when satisfied, predict a paradoxical activation in response to the drug.

$$\frac{1 - K_A}{K_A} < S_{rel} \tag{19}$$

$$RAF_{rel} < \frac{1}{8}(K_A(1 + S_{rel}) - 1) \times (1 + 3K_A(1 + S_{rel})) \tag{20}$$

In the absence of 14-3-3, an equilibrium biased to inactive RAF ($K_A > 1$) was sufficient for existence of paradoxical activation. However, in the presence of an 14-3-3 protein stabilizing that inactive conformation, the strong constraints on the value of K_A are lifted. Even in case of $K_A \leq 1$, paradoxical drug response is predicted where a sufficiently large stabilizing protein (14-3-3) concentration is available (equation 19). The constraints on the concentration of RAF and dimerization rate to create conditions suitable for PA are also relaxed and get further relaxed as a function of increasing 14-3-3 concentration (equation 20).

Note that the total number of RAF dimers in proportion to total RAF, are a product of $(1 + d_{rel})/2$ with active RAF proportion in equation 18.

The supplementary table shows the baseline activity which can be noted to be a monotonically reducing function of 'E5' where,

$$E5 = \frac{8RAF_{rel}}{(1 + K_A(1 + S_{rel}))^2} \tag{21}$$

From the expression for E5 above we can conclude that the baseline activity is reduced when 14-3-3 concentration increases, K_A increases or K_{Smon} reduces (increased binding of 14-3-3 and

RAF). This is consistent with the expectation that when CAS mediating 14-3-3 contributions increase, RAF signaling in the absence of drug is predicted to reduce.

Next, we ask what is the impact on fold change in active RAF relative to no-drug control, as 14-3-3 concentration increases? The fold change defined as maximum active RAF kinase in proportion to baseline active RAF kinase solves to the following expression.

$$MaxFC = \frac{ActiveRAF|_{max}}{ActiveRAF|_{d_{rel}\to 0}} = \frac{8}{27K_A\,(S_{rel}+1)} \times \frac{\left(\sqrt{6RAF_{rel}+1} + \left(6\sqrt{6RAF_{rel}+1} - 9\right)RAF_{rel} - 1\right)}{\left(\sqrt{(K_A S_{rel} + K_A + 1)^2 + 8RAF_{rel}} - K_A S_{rel} - K_A - 1\right)^2} \tag{22}$$

When the conditions for existence of PA derived in equations 19 and 20 are satisfied, the fold change above is a monotonic increasing function of 14-3-3 concentration, K_{dim} and K_A; and a monotonic reducing function of increasing RAF and K_{Smon} (14-3-3 and RAF binding dissociation constant). These conclusions are derived as a function of unbound drug and are solidified once the relationship between unbound and total drug can be globally established. The relationship between total drug available (DTOT) and the unbound (free) drug (d) can be calculated by substituting the equilibrium conditions.

$$\frac{2[A]^2[d]([d]+K_d)}{K_d^2 K_{dim}} + \frac{[A][d]}{K_d} + [d] = Drug \tag{23}$$

We establish a monotonic, direct relationship, by taking the first derivative of the left hand side of above equation with unbound drug and evaluating the conditions when that derivative is non-negative.

$$\frac{d(Drug)}{d[d]} > 0 \tag{24}$$

By splitting this function into three additive terms, we substitute the expression for [A] solved from the equilibrium conditions and show that the derivative relative to unbound drug for each term is entirely non-negative. We do not show the explicit expression for these derivatives here and refer to the attached Mathematica notebook (Section 2.4) instead. We note that these derivatives are positive definite for all positive values of the parameters in the model. Therefore, we obtain that the total Drug is a monotonic increasing function of the unbound drug (for visual example see figure below). The monotonicity of this function implies that our conclusions for paradoxical activation follow through as a function of total drug as well. The relationship between total and unbound drug is numerically demostrated with a few different RAF concentrations in the figure below.

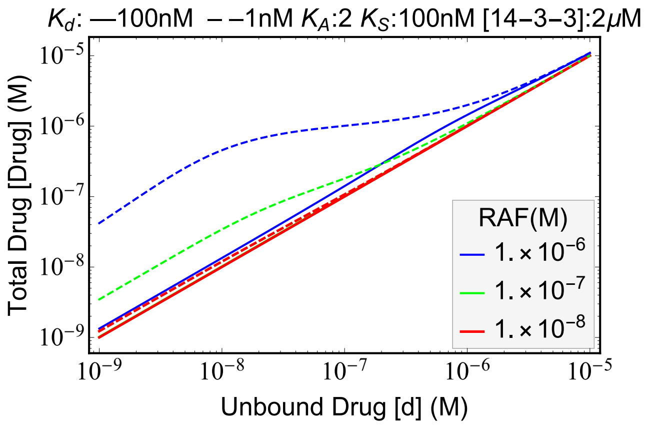

Analytic results in this section were obtained in either of two limits of parameters where 14-3-3 concentration is much higher than RAF or the binding dissociation constant is much smaller. While measurements of sub-cellular concentrations of these proteins simultaneously would be difficult clinically, the former is likely to be true as 14-3-3 protein acts as a scaffold in several pathways beyond MAP kinase including AMPK/mTor as well as TAZ. Moreover, the results are the same if we remove these assumptions and solve the complete model numerically *(see main figures)*.

In conclusion, the CAS model predicts that 14-3-3 when stabilizing auto-inhibited, inactive state of RAF, always reduces the baseline signaling, induces paradoxical activation and expands conditions under which it can occur.

2 14-3-3 mediated RAF dimer stabilization reduces PA and potentiates baseline signaling (DS model)

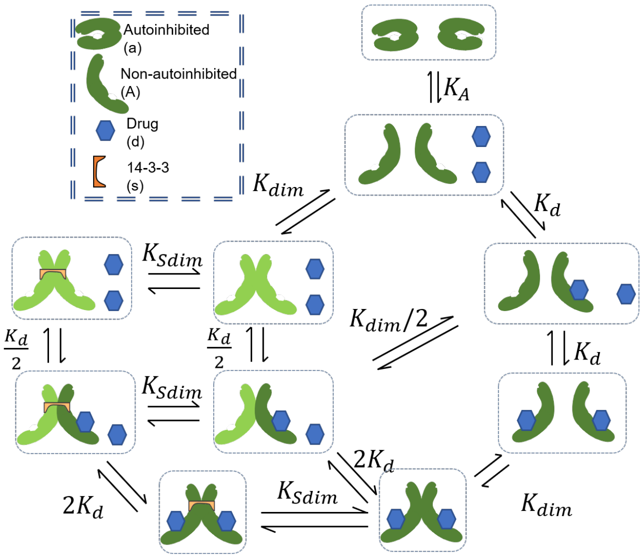

While we have established one role of 14-3-3 protein in RAF activation in the CAS model, 14-3-3 proteins also bind to RAF at a different site to stabilize RAF in an activated quadramer of two RAF and two 14-3-3 proteins [5, 6, 7]. We evaluate how a protein may modify RAF activation

equilibria by stabilizing RAF dimer by defining the DS model as shown above. In this section we outline the derivation of active RAF in DS model and identify conclusions thereof.

For the sake of this exploratory model and also due to lack of detailed data on 14-3-3 and RAF binding kinetics, we assume that the binding of RAF dimer to 14-3-3 dimer is also a simple reaction-diffusion process with a dissociation rate constant, K_{Sdim}. Equilibrium relationships in equations 10-13 and equation 9 from the apply to the DS model and are updated with the expressions below.

$$[AAs] = \frac{[AA][s]}{K_{Sdim}} \tag{25}$$

$$[AAsd] = \frac{2[A]^2[s][d]}{K_{dim}K_{Sdim}K_d} \tag{26}$$

$$[AAsdd] = \frac{[A]^2[s][d]^2}{K_{dim}K_{Sdim}K_d^2} \tag{27}$$

Over the reaction periods, we assume that the total RAF and drug concentrations remain unchanged leading to the conservation equations as below.

$$RAF = a + A + Ad + 2(AA + AAd + AdAd + AAs + AAsd + AAsdd) \tag{28}$$

$$Drug = d + Ad + AAd + AAsd + 2(AdAd + AAsdd) \tag{29}$$

$$STOT = s + AAs + AAsd + AAsdd \tag{30}$$

Following the formalism defined in previous sections, we solve for the unbound RAF [A] by substituting equilibrium relations into RAF conservation equation.

$$\frac{[A]}{RAF} = \frac{2\left(\sqrt{E1^2 + E6} - E1\right)}{E6} \tag{31}$$

where, $E1 = K_A + d_{rel} + 1$, $E6 = 8(1 + d_{rel})^2(1 + s_{rel})RAF_{rel}$ and $s_{rel} = [s]/K_{Sdim}$. The active RAF in proportion to total RAF is defined as sum of drug unbound RAF protomers within RAF dimers.

$$\frac{ActiveRAF}{RAF} = \frac{2([AAs] + [AA]) + AAsd + AAd}{RAF} \tag{32}$$

$$= \frac{2[A]^2([d] + K_d)(K_s + [s])}{K_d K_{dim} K_s RAF} \tag{33}$$

Substituting the expression for unbound RAF, we obtain the expression for Active RAF as shown in the supplementary table. The total dimers share the same relation to active RAF as in previous section.

$$RAF\_Dimers = \frac{1 + d_{rel}}{2} ActiveRAF \tag{34}$$

The baseline activity in response to 14-3-3 is defined as active RAF in the absence of drug. The resulting expression is identical to CAS model albeit as a monotonically increasing function of E7.

$$E7 = \frac{8RAF(1+s_{rel})}{K_{dim}(1+K_A)^2} \tag{35}$$

Therefore, the baseline signaling is an increasing function of RAF, 14-3-3 and reducing function of dissociation constants K_{dim}, K_{Sdim} and the equilibrium constant K_A. This prediction becomes a universal result of the DS model once we can establish that in the absence of drug, total and unbound 14-3-3 are related monotonically and directly. To establish this, we calculate the derivative of [14-3-3] as a function of [s] (see Mathematica Notebook section 3.2) and split the proof of their relationship into two regions of parameter space where [s]>=K_{Sdim} and [s]< K_{Sdim}. In both these limits, we show that the derivative of total [14-3-3] relative to unbound 14-3-3 [s] is a positive definite function thereby establishing the required relationship.

Taking a ratio of active RAF as a function of the drug (equation 33) relative to the baseline signaling, we obtain the fold change predicted a function of the unbound drug parameter d_{rel}.

$$FoldChange = \frac{E7\left(E1-\sqrt{E1^2+E6}\right)^2}{E6\left(\sqrt{E7+1}-1\right)(d_{rel}+1)} \tag{36}$$

Upon taking a derivative relative to s_{rel}, we show that the conditions for this derivative to be positive can never be established by positive valued parameters (Mathematica Notebook Section 3.5). Therefore, we can state that the fold change is always reduces as unbound 14-3-3 levels increase in the DS model. Both the baseline signaling and fold change as a function of the inhibitor become general results of the model when monotonicity is established between total and unbound 14-3-3 and between total and unbound drug. We establish the general monotonic relationship between unbound and total 14-3-3 even in the presence of the drug by taking a derivative of expression for total 14-3-3 as a function of the unbound 14-3-3 and establishing that no solutions exist that equate this derivative to zero (Mathematica Notebook Section 3.6).

2.0.1 Analytic expressions for PA conditions under slowly changing unbound [14-3-3]

Under an assumption that the unbound 14-3-3 [s] changes slowly as a function of the drug as compared to the active RAF kinase itself (Mathematica notebook Section 3.3), we can derive the analytic conditions for DS model to allow PA. Within this limit, when the following condition is satisfied, the first derivative relative to drug concentration of Active RAF is zero and the second derivative is negative.

$$8\mathrm{RAF}_{\mathrm{rel}}(s_{\mathrm{rel}}+1) < (K_A-1)(3K_A+1) \tag{37}$$

Note the similarity between the above expression and the correspondig relationship in a model without 14-3-3 (base model). The above equation is more stringent so as to require not only that conditions supporting PA exist independent of 14-3-3 (i.e. $K_A > 1$) but also that the values of K_A be larger than that required in the absence of 14-3-3 to produce PA. Therefore, 14-3-3 in dimer stabilization role further constraints the conditions under which PA may occur. The same point

can be shown without the above simplifying assumption by numerically solving the conservation and equilibrium equations.

The analytic results on PA in response to drug derived in this model are done as a function of unbound drug. We can also derive a monotonic relationship between unbound and total drug when 14-3-3 varies slowly as a function of the drug (Mathematica Notebook Section 3.4). We can also demonstrate the monotonicity of this relationship numerically for a range of RAF concentrations (below).

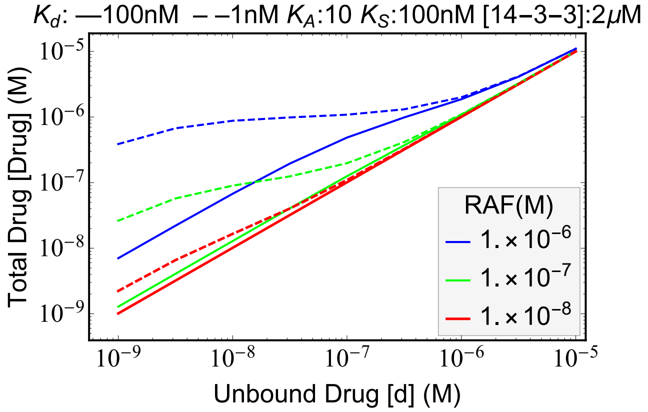

In conclusion, the DS model predicts that as 14-3-3 concentration rises, the baseline signaling rises and paradoxical activation (in proportion to baseline) reduces. Importantly, in this model, 14-3-3 does not induce PA if it is not already present in the absence of 14-3-3 (that is, no PA if $K_A \leq 1$*).*

3 Combining CAS and DS roles of 14-3-3 and RAF interaction

We have previously shown that auto-inhibitory mechanism is sufficient by itself to cause PAMendiratta2023elife. We also found that RAF activation is highly sensitive to the auto-inhibitory dynamical equilibrium with the CAS model in section 1 and showed the impact of stabilizing the RAF dimer with the DS model in section 2. In this section we create a theoretical model of 14-3-3 (denoted in the unbound form [s]) and its interactions with RAF (denoted by a or A) to study the combined role of 14-3-3 proteins in PA induction. We incorporate the auto-inhibited, 14-3-3 stabilized conformation of the kinase formed by the RAF-14-3-3 complex ([as]). We also incorporate the dimer stabilizing role of 14-3-3 where a 14-3-3 dimer forms a complex with a RAF dimer (denoted by [AAs]). The total protein amounts are then given by the following equations.

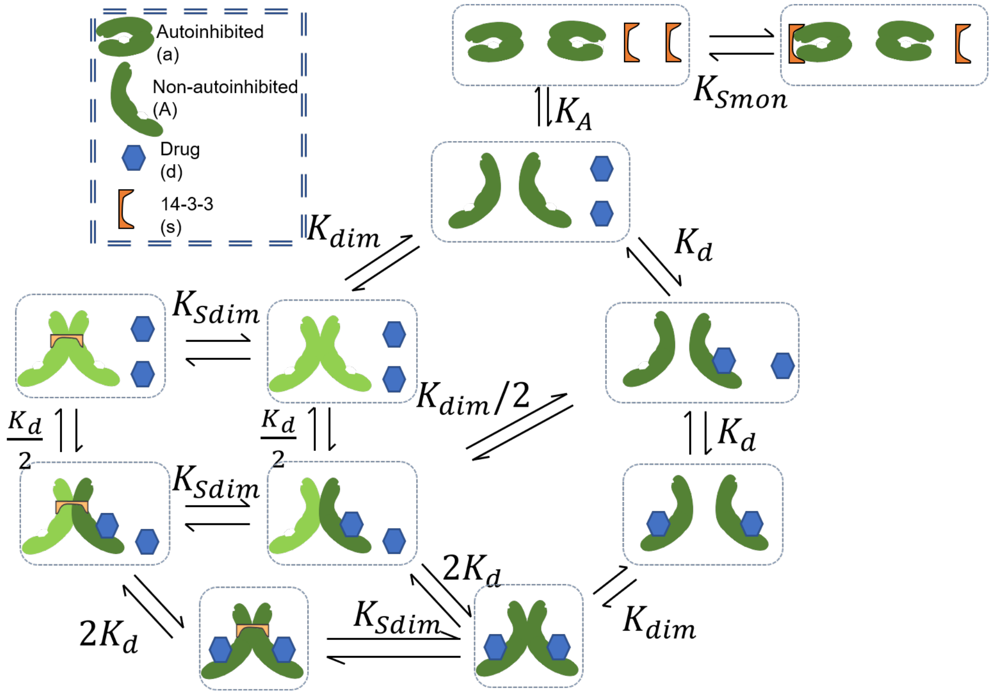

$$[a]+[as]+[A]+[Ad]+ 2([AA]+[AAd]+[AdAd]+[AAs]+[AAsd]+[AAsdd]) = RAF \tag{38}$$

$$[AAs]+[AAsd]+[AAsdd]+[as]+[s] = STOT \tag{39}$$

$$[AAd]+[AAsd]+[Ad]+2([AAsdd]+[AdAd])+[d] = Drug \tag{40}$$

At equilibrium, as in the CAS model, the autoinhibited conformation of the kinase ([as]) can be calculated as a function of the total amount of unbound stabilizing protein ([s]) and unbound inactive kinase ([a]).

$$[as] = \frac{[a][s]}{K_{Smon}} \tag{41}$$

where, K_{Smon} is the dissociation rate constant for binding between RAF monomer and 14-3-3. Note that the assumptions made in analytic components in section 1 to simplify the expressions are not invoked here. The dissociation rate constant for 14-3-3 dimer binding with RAF dimer, denoted K_{Sdim}, in in DS model.

$$[AAs] = \frac{[AA][s]}{K_{Sdim}} \tag{42}$$

We can now use detailed balance for the network diagram above to write the following equilibrium expressions.

$$
\begin{aligned}
[as] &= \frac{[a][s]}{K_{Smon}} \\
[a] &= K_A[A] \\
[Ad] &= \frac{[A][d]}{K_d} \\
[AA] &= \frac{[A]^2}{K_{dim}} \\
[AAd] &= 2\frac{[A]^2[d]}{K_{dim}K_d} \\
[AdAd] &= \frac{[A]^2[d]^2}{K_{dim}K_d^2} \\
[AAs] &= \frac{[A]^2[s]}{K_{dim}K_{Sdim}} \\
[AAsd] &= 2\frac{[A]^2[s][d]}{K_{dim}K_{Sdim}K_d} \\
[AAsdd] &= \frac{[A]^2[s][d]^2}{K_{dim}K_{Sdim}K_d^2}
\end{aligned} \tag{43}
$$

The calculation of total unbound protomer is now more involved due to added complexity of constraint equations and equilibrium relations to be simultaneously solved. The number of active RAF protomers in proportion to total RAF are defined as a combination of 14-3-3 bound and unbound dimer states.

$$= (2([AAs] + [AA]) + AAsd + AAd)/RAF \tag{44}$$

$$= 2\frac{[A]^2([d]/K_d + 1)([s]/K_{Sdim} + 1)}{K_{dim}[RAF]} \tag{45}$$

Substituting for the unbound RAF [A] solved from equation 38, active RAF solves to the following expression.

$$
\frac{ActiveRAF}{[RAF]} = \frac{1}{8(d_{\text{rel}}+1)^3 RAF_{rel}(1+[s]/K_{Sdim})} \times \left((1 + K_A + d_{\text{rel}} + K_A[s]/K_{Smon}) - \sqrt{(d_{\text{rel}} + K_A[s]/K_{Smon} + K_A + 1)^2 + 8(d_{\text{rel}}+1)^2 RAF_{rel}(1+[s]/K_{Sdim})} \right)^2 \tag{46}
$$

where, $RAF_{rel} = RAF/K_{dim}$ and $d_{rel} = [d]/K_d$. The total number of RAF dimers are a sum of RAF dimers bound to drug and 14-3-3 and still share the same simple relationship with active RAF, as in the base model.

$$
\begin{aligned}
Dimers &= [AA] + [AAd] + [AdAd] + [AAs] + [AAsd] + [AAsdd] \\
&\frac{1 + d_{rel}}{2} \times ActiveRAF
\end{aligned} \tag{47}
$$

3.0.1 Analytic expressions for PA conditions under slowly changing unbound [14-3-3]

In order to make analytic evaluation of Active RAF expression in equation 46 more tractable, we evaluate the expression assuming that unbound 14-3-3 is a slowly varying function of unbound drug as compared to the total active kinase (details in Mathematica Notebook section 4.3). As explained before, this assumption is valid when 14-3-3 amount far excceds the kinase which is a likely assumption given plieotropic roles of 14-3-3. However, the assumption's may be questioned with regards to specific 14-3-3 among the seven forms whose expressions are indeed cell-type dependent. However, moving forward with this assumption, we can follow the same formalism as in previous sections and evaluate the conditions of PA by equating first derivative to zero and demanding second derivative of active RAF relative to the variable d_{rel} be negative.

$$K_A > \frac{1}{3\,(1+[s]/K_{Smon})} \times \left(1+2\sqrt{1+6\,(1+[s]/K_{Sdim})\,RAF_{rel}}\right) \tag{48}$$

Therefore, as long as the intrinsic auto-inhibition equilibrium sufficiently favors inactive state (K_A is large enough), PA occurs. Note that with a small value of K_{Smon} and large amount of 14-3-3 (correspondingly large [s]), the condition on intrinsic equilibrium constant is quite weak and could be easily satisfied even if the intrinsic equilibrium prefers active RAF protomer state. However, if the dimerization is strong (small K_{Sdim} or K_{dim}) or RAF concentration is high, the intrinsic auto-inhibition equilibrium is more strongly constrains PA producing conditions. This reproduces the analytic results of sections 1 and 2 where we showed that CAS mechanism expands conditions under which PA may occur while DS mechanism reduces such conditions.

For the relationship derived in equation 48 to be generally applicable to the 14-3-3 RAF interactions - a slowly varying [s] as a function of drug is needed and also a monotonic and direct relationship between total and unbound drug needs to be established within this network model. While it is difficult to show the latter analytically, we evaluate the total drug as a function of unbound drug for a varied range of parameters and show numerical in the figure below that this relationship is indeed direct and monotonic.

Since experiments involve measurement of total 14-3-3 or transfection of exogenous 14-3-3, numerical solutions are derived for total RAF dimers and active RAF protomers without making any simplifying assumptions. The conclusions are in complete agreement with the analytic results obtained in this section, section 2 and section 1.

Non-monotonic PA in response to 14-3-3 may be possible within our model and could be an explanation for the difficulty in 14-3-3 transfection experiments to obtain consistent pERK response. Analytic results identifying under what conditions 14-3-3 induces baseline signaling are described in the next section. The baseline signaling induction characterization also better identifies parameter regions which characterize the 14-3-3 and RAF interaction and help identify expected behavior in response to drug by establishing if one of the 14-3-3 roles dominate or both are balancing each other out.

3.0.2 Baseline signaling without drug in 14-3-3 dual roles model

The RAF active kinase at baseline is calculated by substituting zero drug concentration in equation 46 and is shown in fourth column in the supplementary table. The derivative of baseline RAF signaling relative to expression $E10$ is positive definite and the baseline signaling increases monotonously as a function of E10. Where the expression E10 is,

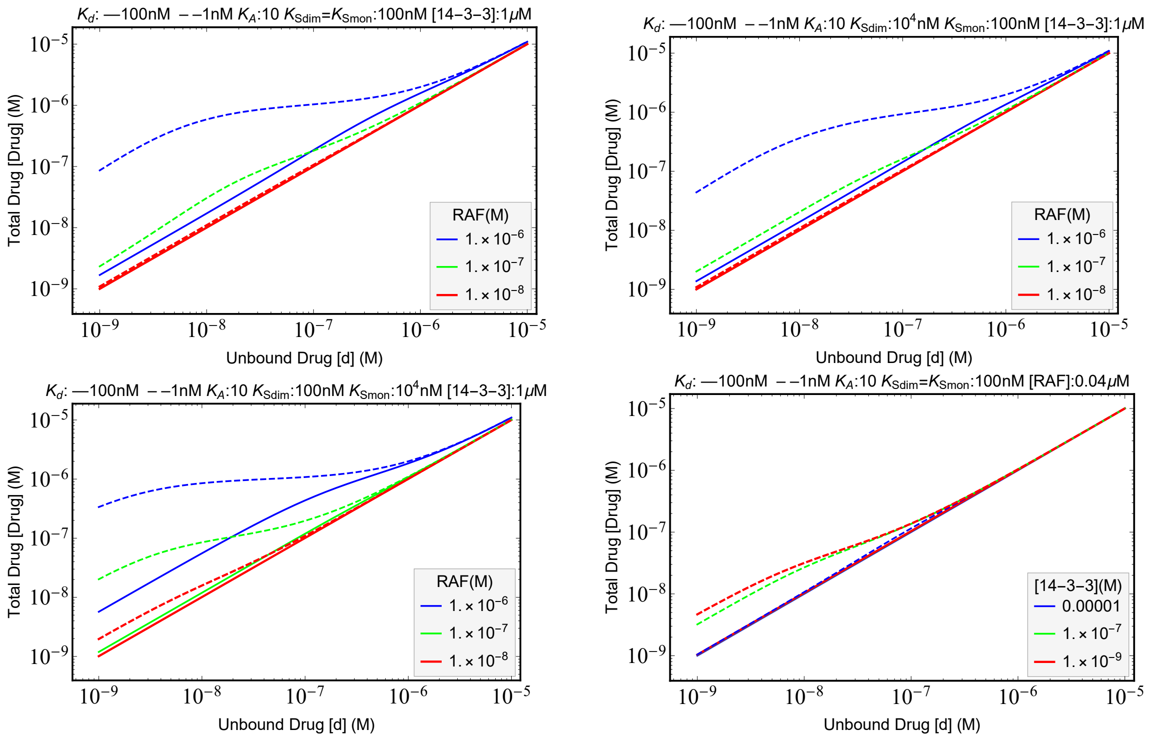

$$E10 = 8RAF_{rel}\frac{[s]/K_{Sdim}+1}{(K_A[s]/K_{Smon}+K_A+1)^2} \tag{49}$$

Taking derivative of above function relative to unbound 14-3-3 concentration ([s])

$$\frac{dE10}{d[s]} = \frac{K_A(-2K_{Sdim}+K_{Smon}-[s])+K_{Smon}}{(K_AK_{Smon}+K_A[s]+K_{Smon})^3} \tag{50}$$

This function becomes zero at only one value of [s].

$$[s] = \left(1+\frac{1}{K_A}\right)K_{Smon} - 2K_{Sdim} \tag{51}$$

For this unbound 14-3-3 concentration to be a physiological value, it has to be positive giving the following contraint on the right hand side,

$$\left(1+\frac{1}{K_A}\right)K_{Smon} < 2K_{Sdim} \tag{52}$$

This expression is easier to satisfy when both intrinsic and 14-3-3 induced auto-inhibition mechanisms are strong (large K_A and small K_{Smon}) and the dimer stabilization induced by 14-3-3 in weak (large K_{Sdim}).

When the intrinsic RAF equilibrium is equally divided among inactive and active state ($K_A = 1$), the condition becomes quite simply, $K_{Smon} < K_{Sdim}$. Since the equation 50 contains unbound 14-3-3 as a negative function in the numerator, with the rest of the term also negative, the derivative E10 relative to [s] becomes negative for the full range of [s] from 0 to [STOT] (also denoted [14-3-3]). That is, upon increase in [14-3-3] concentration, the baseline signaling consistently and monotonically reduces under such condition.

However, when the dimerization dissociation constant is small and 14-3-3 binds strongly to RAF dimers, thereby is necessary for full activation, the possibility of non-monotonic behavior upon increase in [14-3-3] remains open. Since there is only one critical point only simple non-monotonic behaviors are predicted - either an increasing then decreasing function of [14-3-3] or the reverse. To identify which parameter values predict which behavior, we refer back to equation 50 and set the lowest value of [s] - which is zero.

$$\left.\frac{dE10}{d[s]}\right|_{s=0} \propto K_A(-2K_{Sdim}+K_{Smon})+K_{Smon} \tag{53}$$

Requiring that the above be negative reproduces the condition in equation 52. Result being that the function is always negative and therefore increased [14-3-3] always reduces baseline signaling. When the condition in equation 52 is not satisfied, the DS role is sufficiently significant and the derivative of E10 is positive in the limit of low unbound [14-3-3] (which is equal to total [14-3-3] in this limit). Therefore increase in [14-3-3] results in increased baseline signaling. Next, the maximum possible value of [s] is [STOT]. At this value the derivative of E10 is as follows.

$$\left.\frac{dE10}{d[s]}\right|_{s=STOT} \propto K_A(-2K_{Sdim}+K_{Smon})+K_{Smon}-K_A[STOT] \tag{54}$$

If this function is also positive, the baseline RAF signaling is again a monotonic and a direct function of total [14-3-3]. Requiring above equation to be positive imposes the following constraint relation.

$$\left(1+\frac{1}{K_A}\right)K_{Smon} > 2K_{Sdim}+[STOT] \tag{55}$$

The above inequality is satisfied when the auto-inhibition constants both intrinsic and 14-3-3-induced are sufficiently weak (small K_A and large K_{Smon}) combined with strong dimerization (small K_{Sdim}) and low 14-3-3 concentration (small [STOT]). Under this condition, rising [14-3-3] induces baseline signaling.

However, given sufficient [14-3-3], the condition in equation 55 cannot be satisfied. When this happens, the STOT term will start dominating and the baseline signaling is reduced by further 14-3-3. *In fact, due to the nature of the overall dependence on [s] in equation 50, this is a general outcome of our model - sufficient 14-3-3 reduces baseline signaling.*

In the end, there are three possible outcomes of increased levels of 14-3-3 protein concentration,

1. When CAS role of 14-3-3 is strong and DS role weak - increased [14-3-3] reduces baseline signaling

2. When DS role of 14-3-3 is strong and CAS role is weak - increased [14-3-3] first increases then reduces baseline signaling.

While eventual reduction in RAF signaling with sufficient 14-3-3 is robust property of RAF-14-3-3 interaction mechanism, it may not be realizable in a cellular environment as 14-3-3 binds many other proteins beyond RAF. Also it is important to point out that the trick of evaluating the functional forms in asymptotic limits of unbound 14-3-3 allowed us to draw analytic, global conclusions.

3.0.3 Summary

1. When DS role is dominant, baseline RAF signaling is induced by 14-3-3. Under this condition, following are true:

1.1.If CAS role is not too weak: 14-3-3 will still induce PA. Both fold change relative to no-drug and the maximum %active RAF are induced further in the presence of the drug and increased 14-3-3.

1.2. If CAS role is weak: Strong internal auto-inhibition equilibrium bias towards inactive state (KA>>1) is required in addition to 14-3-3 CAS to induce PA drug response.

1.3. If CAS role is weak AND internal auto-inhibition equilibrium strongly favors non-autoinhibited RAF monomer state (KA<1): 14-3-3 will reduce PA.

With DS role dominant, 14-3-3 induction allows for PA induction only when 14-3-3 that is free to interact with RAF is limiting. *This is the limit in which we propose that the cell lines evaluated in this work (and likely, the cells in clinical settings with PA drug response) operate.*

2. When CAS role is dominant, baseline RAF signaling is reduced by 14-3-3. Here, 14-3-3 will generally induce a strong PA and expand conditions under which PA occurs.

3. When DS and CAS roles balance out, [14-3-3] does not change baseline signaling substantially. Here, 14-3-3 induces more PA and ease conditions under which PA occurs via CAS mechanism.

4 Numerical model illustrations

4.1 Base model of RAF autoinhibition, dimerization and drug binding

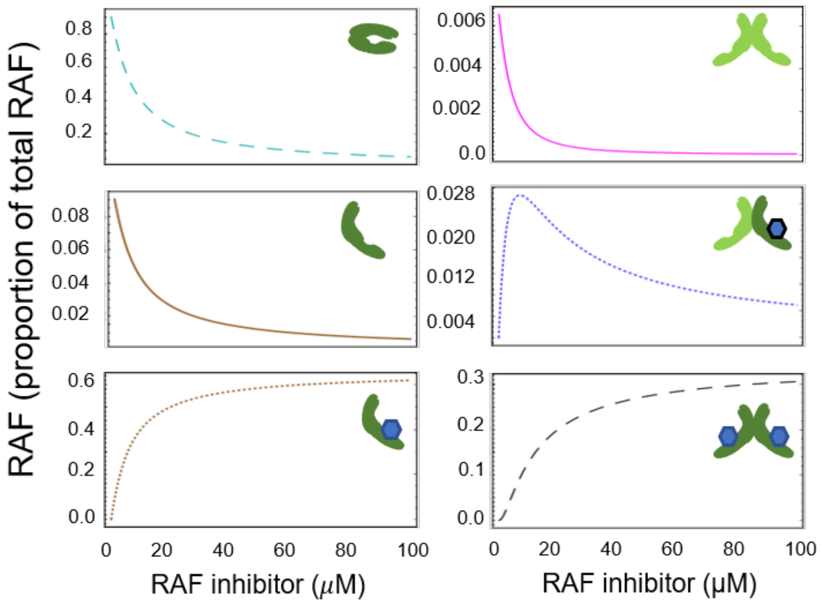

To understand and illustrate how paradoxical activation (PA) of RAF signaling arises from this mechanism, we considered the proportion of RAF in each of its possible states: (i) autoinhibited RAF monomer, (ii) non-autoinhibited RAF monomer that is not bound to drug, (iii) non-

autoinhibited monomer that is bound to drug, (iv) RAF dimer with no drug bound, (v) RAF dimer with one of two kinase domains bound to drug, (vi) RAF dimer with both kinase domains bound to drug. We considered the total amount of kinase activity to be the number of RAF protomers within a dimer that are not bound to drug. The essential role of dimerization for wild-type RAF activation is supported by prior work [8, 9]. As the RAF inhibitors described are ATP competitive [10, 11], there can be no kinase activity within the dimer when both protomers are bound to inhibitor. However, a RAF dimer is believed to be capable of signaling when only one of the two protomers is bound to drug [12].

Before a drug is given, a significant fraction of RAF is autoinhibited and there are low levels of non-autoinhibited RAF and RAF dimers. As RAF inhibitor levels increase, the level of autoinhibited RAF progressively declines. Non-autoinhibited RAF distributes between drug-bound monomeric and dimeric forms while the unbound monomeric form maintains equilibrium with the autoinhibited RAF. The increased quantity of RAF dimers reflects the increased pool of RAF proteins that are non-autoinhibited and therefore capable of dimerization. This results in a drug-dependent increase in RAF dimers bound to drug in one site and thereby the increase in total RAF kinase activation that accounts for PA. The quantity of drug-bound RAF monomer and doubly-drug-bound RAF dimer progressively increases to saturation as a function of the drug amount, resulting in the eventual reduction in RAF kinase activity that is associated with PA dose responses.

4.2 Models of 14-3-3 with both CAS and DS roles

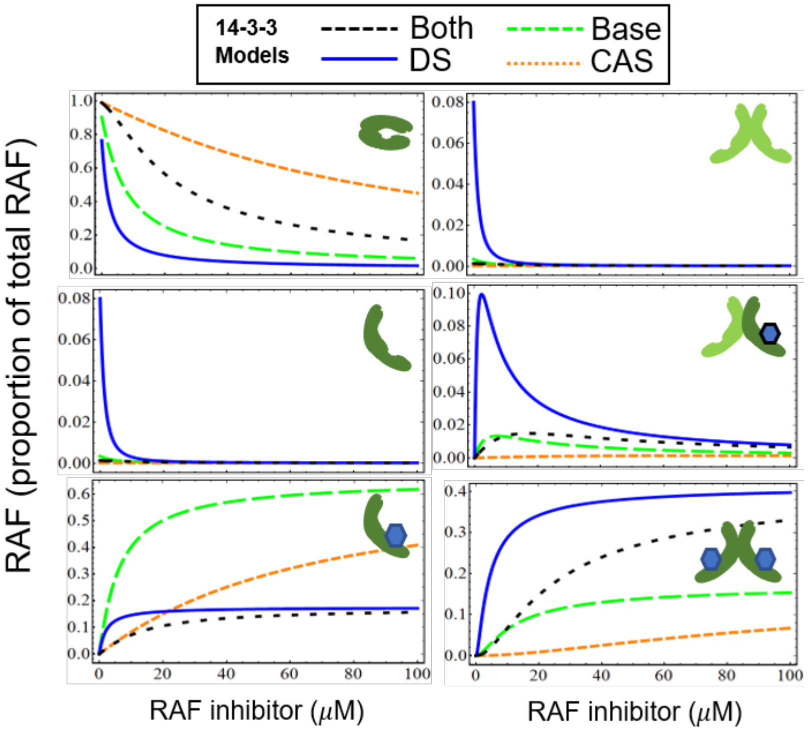

Following up on the crucial role of the conformational autoinhibition mechanism, we constructed an in-silico model of 14-3-3 and RAF interactions. To illustrate how PA is modified in presence of 14-3-3, we considered the proportion of RAF in each of its possible states: (i)

autoinhibited RAF monomer including inactive RAF monomer and RAF monomer bound to 14-3-3 dimer, (ii) non-autoinhibited RAF monomer that is not bound to drug, (iii) non-autoinhibited monomer that is bound to drug, (iv) RAF dimer with no drug bound, (v) RAF dimer with one of two kinase domains bound to drug, (vi) RAF dimer with both kinase domains bound to drug. The RAF dimer states (iv,v,vi) include both the 14-3-3 dimer bound and 14-3-3 unbound states. We do not include all the possible 14-3-3 monomer and RAF monomer states in our models as none of those intermediate states have been crystallized or biochemistry characterized. However, our coarse grained model contain all of the final, stable states which have been recently characterized in structural studies [3, 5]. We therefore expect that this model captures the interaction of RAF and 14-3-3 in a coarse grained picture and represents the salient behaviors when these concentrations are modified significantly. The total amount of kinase activity is defined as before, to be the number of RAF protomers within a dimer that are not bound to drug. These dimers now include both the 14-3-3 bound quadromers and unbound RAF dimers.

Presence of 14-3-3 substantially induces further PA (Black, dashed curves) relative to the base model with no 14-3-3 (green, dashed). The effect on drug-free signaling and the amount of drug induced activation depends on the competition between the two roles of 14-3-3, dimer stabilization (DS) and conformational autoinhibition stabilization (CAS). However, the initial condition from the base model that RAF should mainly be inactive is no longer necessary to generate PA 48. Sufficiently strong but reversible interaction between inactive RAF monomer and 14-3-3 (CAS role) is sufficient to create the reservoir that is tapped by the drug to generate PA instead of inhibition (Orange, dotted curves). At baseline (no drug), CAS model (with no DS 14-3-3 interaction) shows enhancement of inactive RAF state relative to the base, DS or both-role models. However, as drug is added, the large reservoir of inactive RAF is tapped to create a significant enhancement levels of partly-active RAF dimers. When plot relative to the baseline RAF activation, the CAS model shows substantially more PA than any other model.

The DS model shows RAF activation cycle when only dimer stabilization role of 14-3-3 and RAF interaction is present (Blue, solid curves). RAF equilibrium is shifted to the active dimer state at the baseline (no drug) showing a high baseline activation of RAF and lower relative amount of the inactive RAF monomer. However, the active RAF levels rapidly drop upon exposure to the drug which binds with these active protomers resulting in enhancement of both fully and partly inactivated RAF dimers. Due to a lowered size of the reservoir of inactive RAF at baseline, the DS model shows lower PA levels than the base model.

While it may seem from our curves that show active RAF as a percentage of total RAF that dimer stabilization model shows substantially higher levels of activation, it is important to note that we do not know baseline levels in cells. The PA is significant only relative to that baseline which the cells sustain in. In that context, DS model produces no PA and CAS mechanism produces large PA. From this in-silico analysis, we conclude that 14-3-3 likely induces PA via CAS mechanism.

Finally, our use of downstream ERK phosphorylation as a readout of RAF kinase activity may introduce a saturable readout of RAF kinase activity, potentially explaining why we can robustly see increases in PA range (which only requires a monotonic relationship between RAF kinase activity and ERK phosphorylation) but not PA fold change (for which the downstream observable would need to be linear as a function of RAF kinase activity for it to be directly measurable). The ability to detect an increase in the range of drug concentrations that display PA would not be limited by saturation and would only require a monotonic relationship between RAF kinase activity and ERK phosphorylation, which seems reasonable to assume. 14-3-3 proteins are promiscuous and

pleiotropic, so it is possible that part of the effects observed with 14-3-3 transfection follow from other activities of 14-3-3 proteins; it is not possible to rule-out unknown alternative mechanisms involving 14-3-3 proteins.

References

[1] G. Mendiratta, E. Stites, *Elife* **12**, e82739 (2023). 4, 6

[2] E. Freed, M. Symons, S. G. Macdonald, F. McCormick, R. Ruggieri, *Science* **265**, 1713 (1994). 4

[3] E. Park, *et al.*, *Nature* pp. 1–5 (2019). 4, 19

[4] C. Wofsy, B. Goldstein, K. Lund, H. Wiley, *Biophysical journal* **63**, 98 (1992). 6

[5] Y. Kondo, *et al.*, *Science* **366**, 109 (2019). 8, 19

[6] J. A. Thorson, *et al.*, *Molecular and cellular biology* **18**, 5229 (1998). 8

[7] G. Tzivion, Z. Luo, J. Avruch, *Nature* **394**, 88 (1998). 8

[8] J. Hu, *et al.*, *Cell* **154**, 1036 (2013). 18

[9] H. Lavoie, M. Therrien, *Nature reviews Molecular cell biology* **16**, 281 (2015). 18

[10] T. Brummer, C. McInnes, *Oncogene* **39**, 4155 (2020). 18

[11] N. P. Liau, *et al.*, *Nature structural & molecular biology* **27**, 134 (2020). 18

[12] Z. Karoulia, *et al.*, *Cancer cell* **30**, 485 (2016). 18
