# Supplementary material for: Mathematical modeling suggests 14-3-3 proteins modulate RAF paradoxical activation
Source: PLoS Comput Biol. 2025 Aug 1;21(8):e1013297. doi: 10.1371/journal.pcbi.1013297 (PMC12407542; doi:10.1371/journal.pcbi.1013297)
Supplement: S1 Data — Supplementary files that include the code required to analyze and evaluate the models and to reproduce all of the results presented in this study. (ZIP) [file pcbi.1013297.s005.zip › Supplementary Data Mendiratta RAF 14-3-3/Code/Analytical_Validations_Mathematica_All_Models/MathematicaNotebook_AllModels_index.pdf]

In[ ]:= $Version (*Mathematica version used to generate this file.*)

Out[ ]= 12.0.0 for Microsoft Windows (64-bit) (April 6, 2019)

Conformal Auto-inhibition Mechanism and 14-3-3 scaffold

```
baseparams = {KA → 10., Kd → 0.1, Kdim → 0.1, RAF → 0.04, STOT → 1.0, Ksm → 0.2, KsD → 0.02};
(*units: μM ∀ params but KA*)
```

Section 1. Base/CA Model: Auto-inhibition is sufficient to mediate PA

(Published Mendiratta et.al. elife 2023)

- **1.1.1. Analytic Solution to the model**
- **1.1.2. Baseline Signaling (drug-free)**
- **1.1.3. Conditions on parameter regions for activation in response to the drug**
- **1.1.4. Monotonic relationship between unbound (d) and total (DTOT) drug concentrations**
- **1.1.5. Analytic Expressions for maximum Fold Change (FC)**
- **1.1.6. Convert to Python**

Section 2. 14-3-3 proteins stabilize Auto-inhibited state (CAS)

In[ ]:=
```
(*Restart kernel to prevent previous model variables to leak into following results*)
Quit[]
```

- **2.1. Analytic solutions of the model**
- **2.2. Baseline signaling in the absence of drug**
- **2.3. Conditions on parameter regions for activation in response to the drug**
- **2.4. Monotonic Relationship between total and unbound drug.**
- **2.5. Analytic Expressions for maximum Fold Change (FC)**
- **2.6. Convert to Python**

Section 3. 14-3-3 proteins only stabilize Dimer state (DS)

In[ ]:=
```
(*Restart kernel to prevent previous model variables to leak into following results*)
Quit[]
```

- **3.1. Analytic solutions of the model**
- **3.2. Baseline Signaling**
- **3.3. Conditions on parameter regions for activation in response to the drug**
- ***Unbound 14-3-3 is a slowly varying function of drug compared to active kinase***
- ***PA Conditions***
- **3.4. Monotonic Relationship between total and unbound drug.**
- **3.5. Analytic Expressions for Fold Change (FC)**

- **3.6. Relationship between unbound and total 14-3-3**
- **3.7. Convert to Python**

Section 4. 14-3-3 proteins stabilize both Autoinhibited and Dimer state (CAS+DS)

In[ ]:=
```
(*Restart kernel to prevent previous model variables to leak into following results*)
Quit[]
```

- **4.1. Analytic solutions of the model**
- **4.2. Baseline Signaling**
- **4.3. Conditions on parameter regions for activation in response to the drug**
  - ***Unbound 14-3-3 is a slowly varying function of drug compared to active kinase***
  - ***PA conditions***
  - ***Monotonic Relationship between total and unbound drug.***
- **4.4. Analytic Expressions for maximum Fold Change (FC)**
- **4.5. Convert to Python**

Descriptive, example Plots

Note: Initialize baseparams variable (first line) and all first sub sections (*.1) to get the following plots.

In[77]:=
```
Sty[x_] := Style[x, 22, Bold, FontFamily → "Times"];
```

In[88]:=
```
(*the fourth solution is positive definite -
 recheck each time run on a new mathematica version.*)
plfn1 = {(fnActiveRAFbase/(fnActiveRAFbase /. d_rel → 0)) /. {RAF_rel → RAF/Kdim, d_rel → d/Kd},
    (fnActiveRAF1433CAS/(fnActiveRAF1433CAS /. d_rel → 0)) /. {RAF_rel → RAF/Kdim,
      S_rel → STOT/Ksm, d_rel → d/Kd}, ((fnActiveRAF1433DS/(fnActiveRAF1433DS /. d_rel → 0)) /.
       {RAF_rel → RAF/Kdim, s_rel → s/KsD, d_rel → d/Kd}) /. numsol23As,
    (fnActiveRAF1433/(fnActiveRAF1433 /. d_rel → 0) /. {RAF_rel → RAF/Kdim, d_rel → d/Kd} /.
      numsol24As)} /. baseparams;
```

In[95]:=
```
legendlist = Sty["AK\n"];
rvals = {0.07, 0.03, 2., 0.02};
r1 = {Medium, Small, Tiny, Large};
cols = {Green, Orange, Blue, Black};
cs = Table[Directive[ Thickness[0.01], cols[[i]], Dashing[{rvals[[i]], r1[[i]]}]], {i, 4}];
(*dashing style by model*)
LogPlot[plfn1, {d, 0, 100}, Frame -> True, ImageSize -> {500},
 FrameStyle -> Thickness[0.004], FrameTicksStyle -> Directive[25, Black],
 PlotRange -> Full, PlotStyle → cs, PlotLegends -> {"no 14-3-3", "CAS", "DS", "CAS+DS"}]
```

Out[100]=

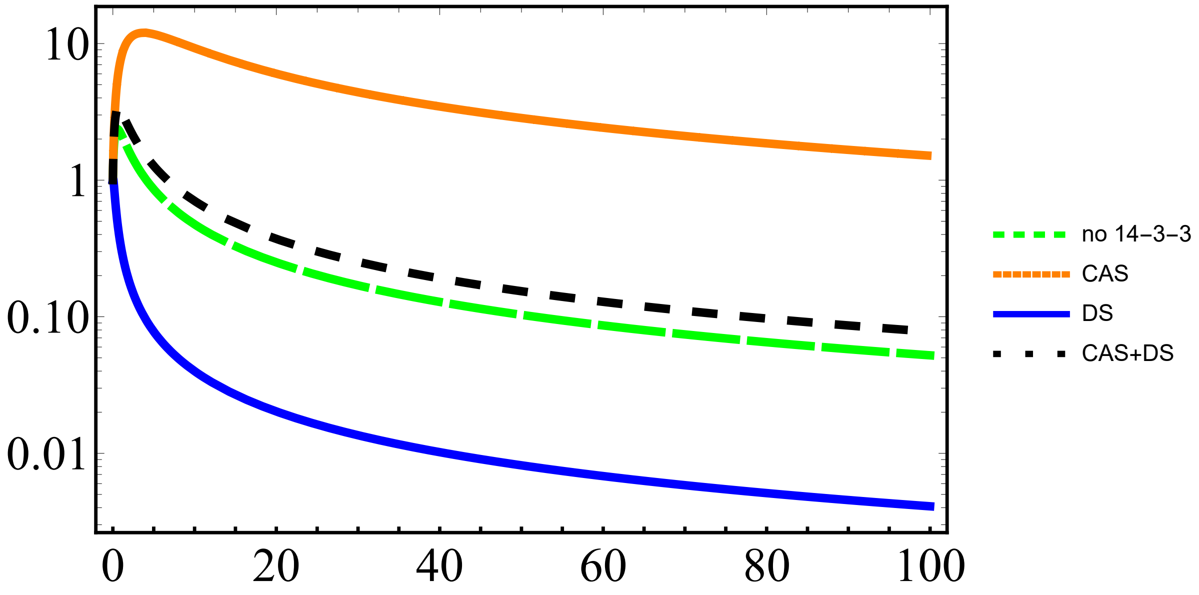

In[101]:=
```
fn2part[1] =
  SimplifyPars[({a, AA, AAd, AdAd, A, Ad} / RAF /. rep21 /. sol21A) /. d_rel → (d / Kd)] /. baseparams;
fn2part[2] = SimplifyPars[({a + as, AA, AAd, AdAd, A, Ad} / RAF /. rep22 /. sol22A) /. d_rel → (d / Kd)] /.
   baseparams;
fn2part[3] = ({a, AA + AAs, AAd + AAsd, AdAd + AAsdd, A, Ad} / RAF /. rep23 /. numsol23As) /.
    d_rel → (d / Kd) /. baseparams;
fn2part[4] = ({a + as, AA + AAs, AAd + AAsd, AdAd + AAsdd, A, Ad} / RAF /. rep24 /. numsol24As) /.
    d_rel → (d / Kd) /. baseparams;
plfn = Table[fn2part[i][[j]], {j, 6}, {i, 4}];
legendlist = Sty /@ { "a\n", "AA\n", " AAd\n", " AdAd\n", " A\n", " Ad\n"};
Table[Plot[Evaluate[plfn[[i]]], {d, 0, 100}, Frame -> True, ImageSize -> {500},
  FrameStyle -> Thickness[0.004], FrameTicksStyle -> Directive[25, Black],
  PlotRange -> Full, PlotStyle → cs, PlotLegends -> legendlist[[i]]], {i, Length[plfn]}]
```

Out[107]= {

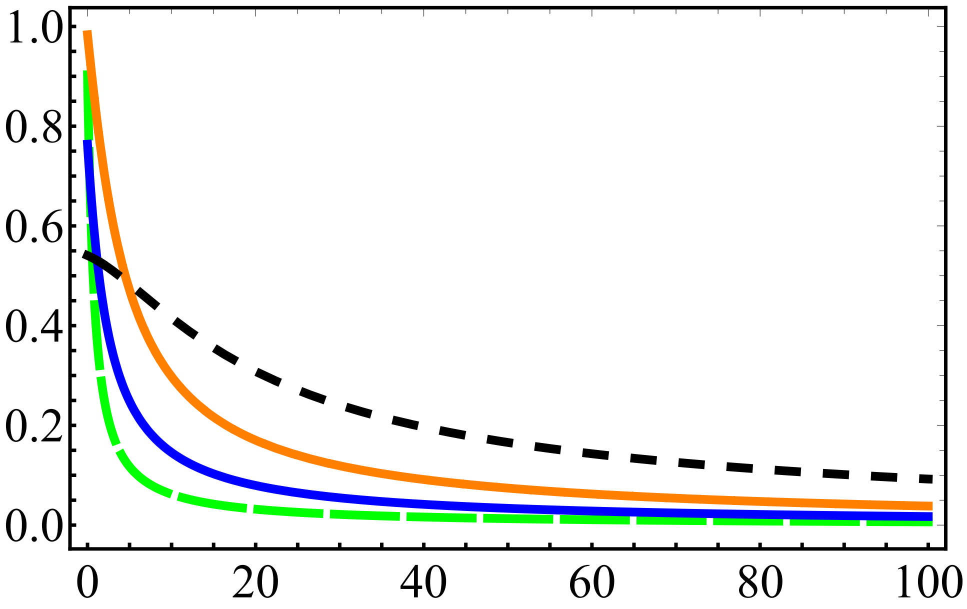
1.0
0.8
0.6
0.4
0.2
0.0
0
20
40
60
80
100

a ,

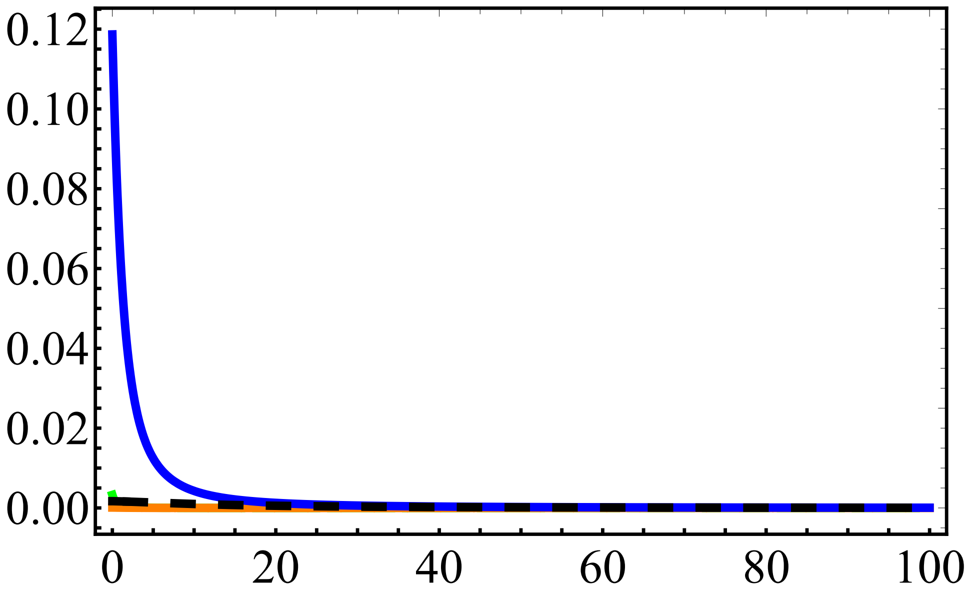
0.12
0.10
0.08
0.06
0.04
0.02
0.00
0
20
40
60
80
100

AA ,

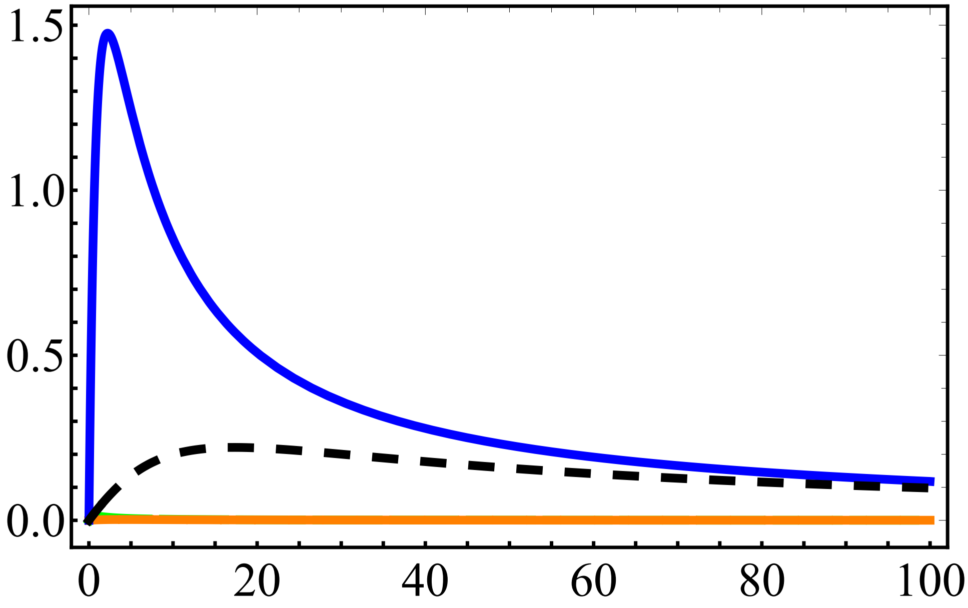

AAd ,

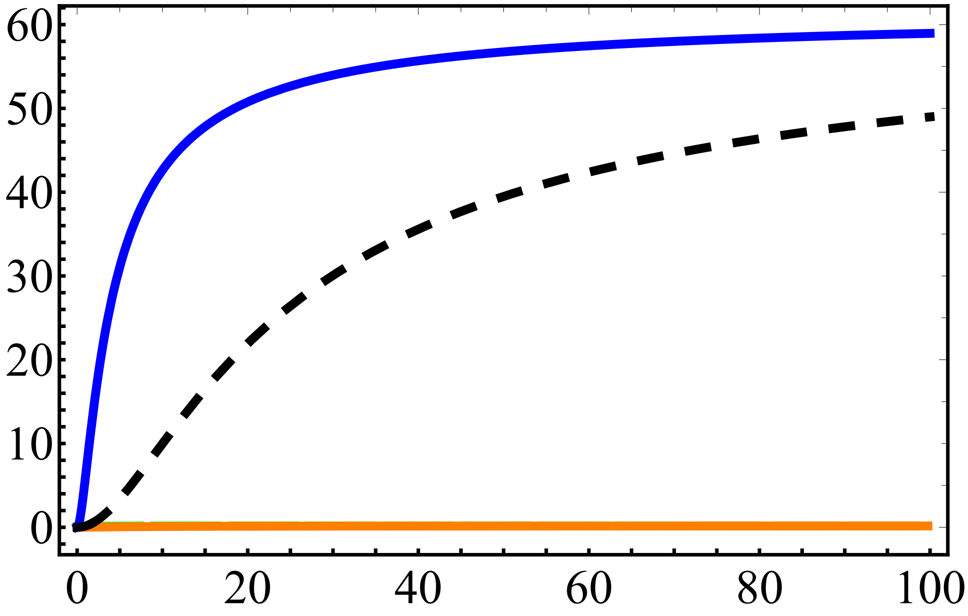

AdAd ,

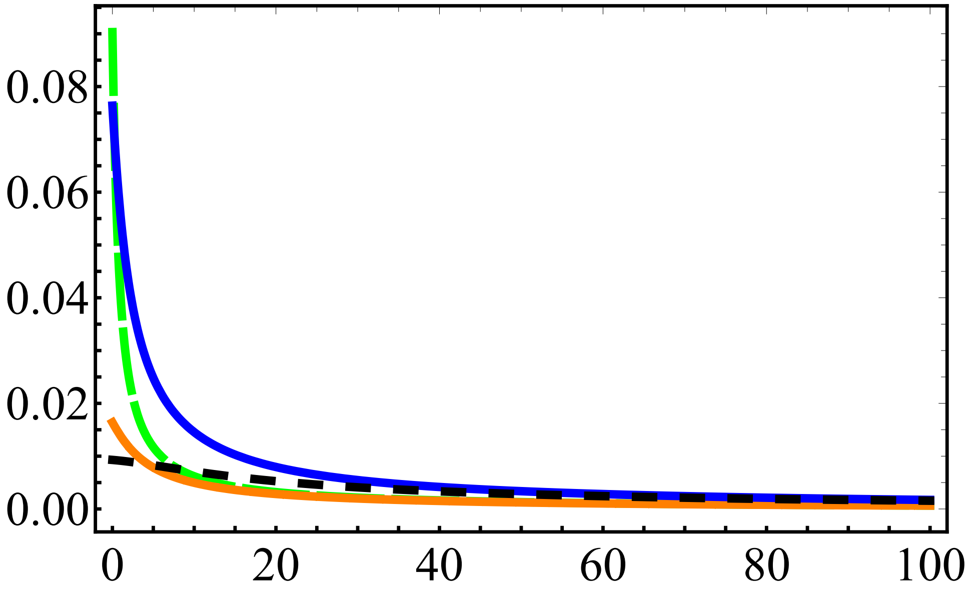

A ,

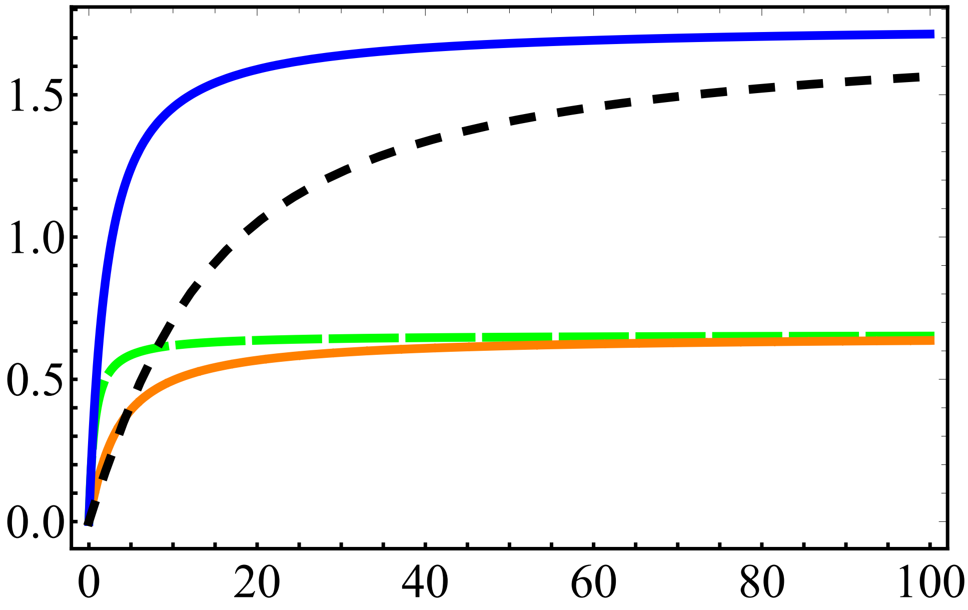

Ad}

{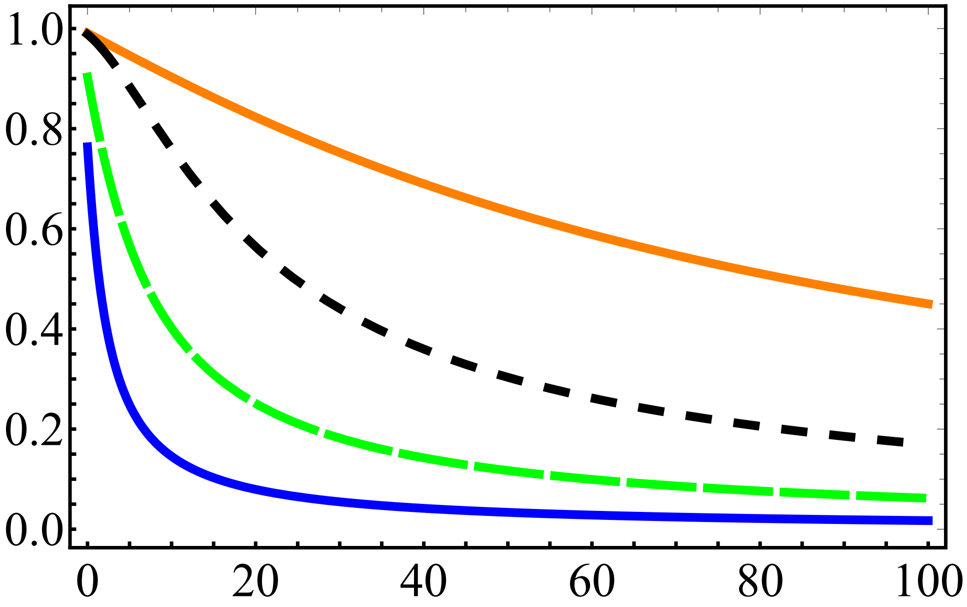
1.0
0.8
0.6
0.4
0.2
0.0
0
20
40
60
80
100

"a\n",

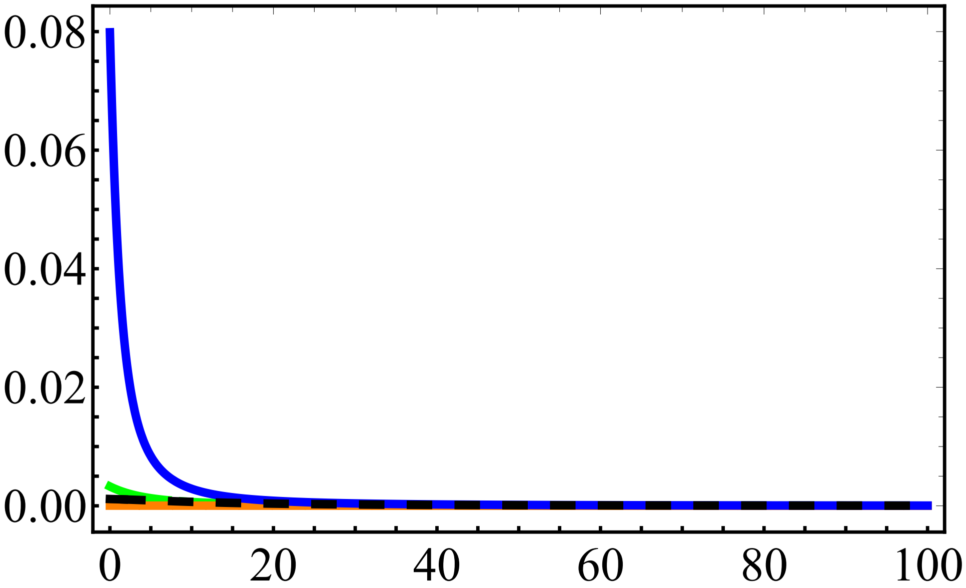
0.08
0.06
0.04
0.02
0.00
0
20
40
60
80
100

"AA\n",

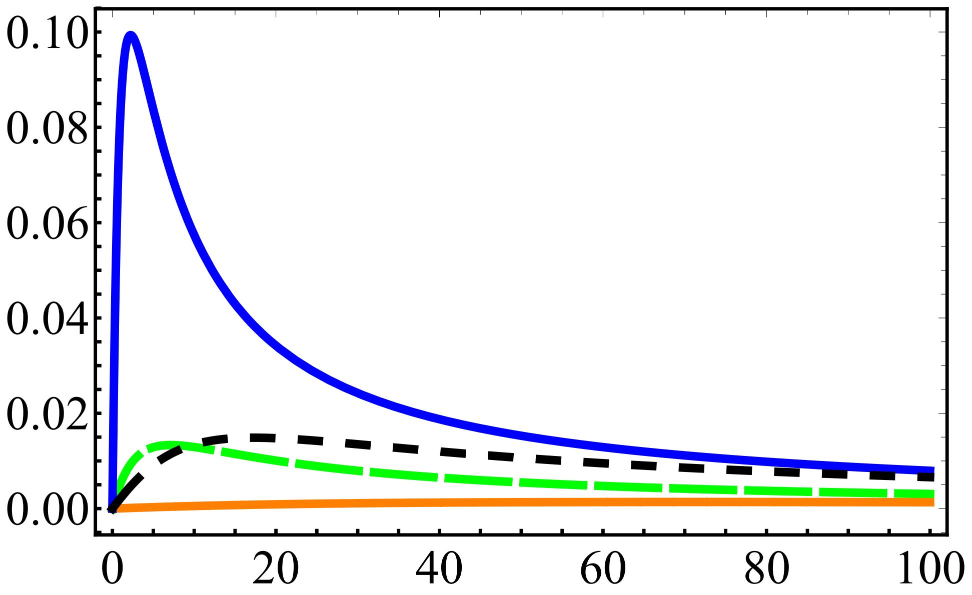
0.10
0.08
0.06
0.04
0.02
0.00
0
20
40
60
80
100

" AAd\n",

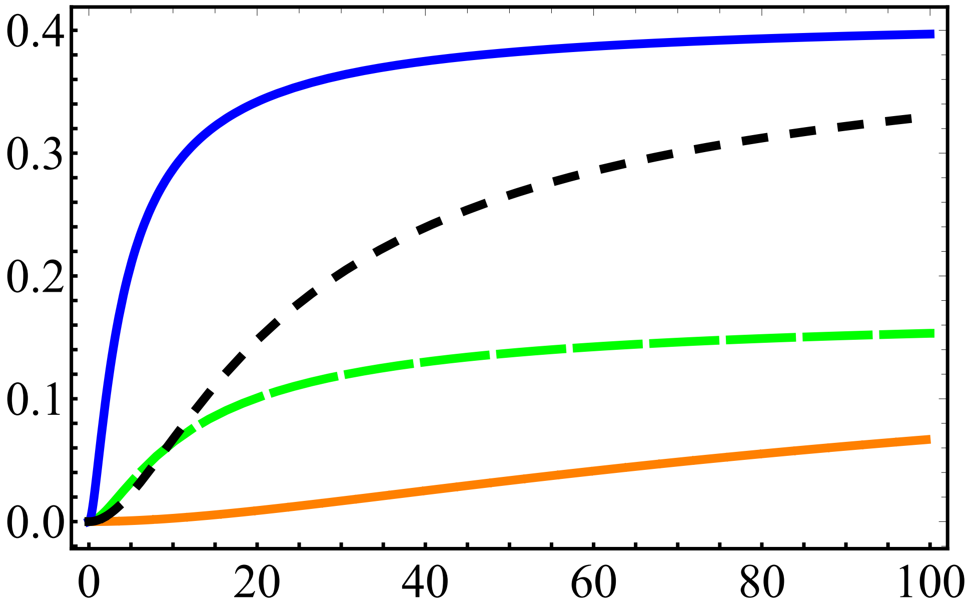
0.4
0.3
0.2
0.1
0.0
0
20
40
60
80
100

" AdAd\n",

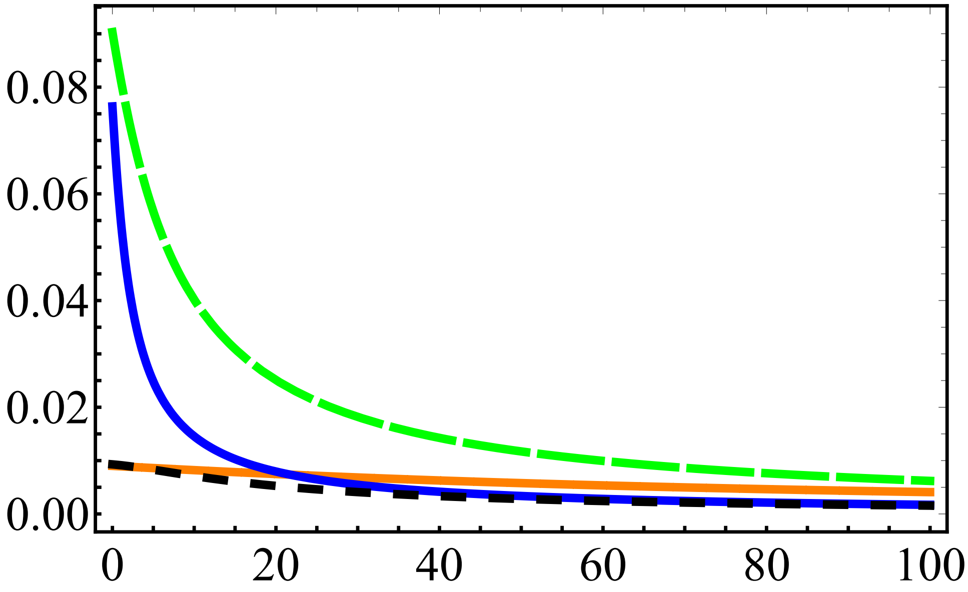
0.08
0.06
0.04
0.02
0.00
0
20
40
60
80
100

" A\n",

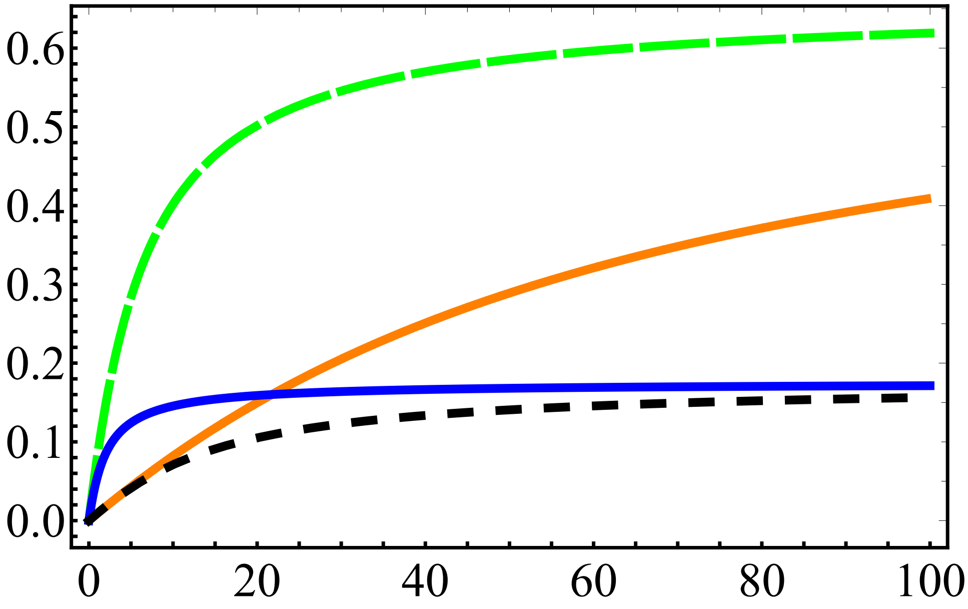
0.6
0.5
0.4
0.3
0.2
0.1
0.0
0
20
40
60
80
100

" Ad\n"}
