# Supplementary material for: Mathematical modeling suggests 14-3-3 proteins modulate RAF paradoxical activation
Source: PLoS Comput Biol. 2025 Aug 1;21(8):e1013297. doi: 10.1371/journal.pcbi.1013297 (PMC12407542; doi:10.1371/journal.pcbi.1013297)
Supplement: S1 Data — Supplementary files that include the code required to analyze and evaluate the models and to reproduce all of the results presented in this study. (ZIP) [file pcbi.1013297.s005.zip › Supplementary Data Mendiratta RAF 14-3-3/Code/Analytical_Validations_Mathematica_All_Models/MathematicaNotebook_AllModels.pdf]

In[ ]:=
```
$Version (*Mathematica version used to generate this file.*)
```
Out[ ]=
```
12.0.0 for Microsoft Windows (64-bit) (April 6, 2019)
```

Conformal Auto-inhibition Mechanism and 14-3-3 scaffold

```
baseparams = {KA → 10., Kd → 0.1, Kdim → 0.1, RAF → 0.04, STOT → 1.0, Ksm → 0.2, KsD → 0.02};
(*units: μM ∀ params but KA*)
```

Section 1. Base/CA Model: Auto-inhibition is sufficient to mediate PA

(Published Mendiratta et.al. elife 2023)

- **1.1.1. Analytic Solution to the model**
- **1.1.2. Baseline Signaling (drug-free)**
- **1.1.3. Conditions on parameter regions for activation in response to the drug**
- **1.1.4. Monotonic relationship between unbound (d) and total (DTOT) drug concentrations**
- **1.1.5. Analytic Expressions for maximum Fold Change (FC)**
- **1.1.6. Convert to Python**

Section 2. 14-3-3 proteins stabilize Auto-inhibited state (CAS)

In[ ]:=
```
(*Restart kernel to prevent previous model variables to leak into following results*)
Quit[]
```

- **2.1. Analytic solutions of the model**

We define the equilibrium rates constrained by using the principle of detailed balance as replacement rules ‘rep’. We then calculate conservation equations for total RAF and total Drug as a function of unbound drug, unbound RAF concentrations and model parameters as in the case of section 1.

In[17]:=
```
vars = {a, A, d, AA, Ad, AAd, AdAd, as};
repcycles = {AA → A^2/Kdim, AAd → (2 A^2 d)/(Kdim Kd), AdAd → (A^2 d^2)/(Kdim Kd^2), Ad → (A d)/Kd, as → (a STOT)/Ksm};
(* as→(STOT a)/(a+Ksm) is the complete expression however,
the reduced expression is valid in the limit of STOT>>RAF and Ksm>>RAF*)
repc1 = {a → A KA};
rep22 = Join[repcycles /. repc1, repc1];
Consrv[eqns_] :=
  {Simplify[eqns[[1]] + eqns[[2]] + eqns[[5]] + eqns[[8]] + 2 (eqns[[4]] + eqns[[6]] + eqns[[7]])],
   Simplify[eqns[[3]] + eqns[[5]] + eqns[[6]] + 2 (eqns[[7]])]};
RafActivity[vars_] := 2 vars[[4]] + vars[[6]];
eqnsconsrv = Thread[Simplify[Consrv[vars] /. rep22] == {RAF, DTOT}];
```

In[24]:=
```
SimplifyPars[x_] := Simplify[x,
   {Kd > 0, RAF > 0, DTOT ≥ 0, d_rel ≥ 0, RAF_rel > 0, Kdim > 0, KA > 0, d > 0, Ksm > 0, S_rel ≥ 0, STOT ≥ 0}];
sol22A = SimplifyPars[Solve[eqnsconsrv[[1]], A]][[2]];
(*the first solution is negtive definite and second is positive.*)
fnActiveRAF1433CAS0 = FullSimplify[SimplifyPars[RafActivity[vars]/RAF /. rep22 /. sol22A]];
repratios = {Kdim → (RAF/RAF_rel), d → (d_rel Kd), STOT → S_rel Ksm};
(*define concentrations relative to equilibrium rate constants*)
fnActiveRAF1433CAS = SimplifyPars[fnActiveRAF1433CAS0 /. repratios];
(*Show the active kianse protomer proportion as a function of relative drug,
stabilizing protein and kinase concentrations*)
rep221 = {(1 + d_rel + KA + KA S_rel) → E4, RAF_rel → E2/8/(1 + d_rel)^2};
fnActiveRAF1433CAS = fnActiveRAF1433CAS /. Kd → 1
(*Note that Kd is set to 1 to enforce the cancellation of Kd in equation 23*)
(fnActiveRAF1433CAS //. rep221)
```

Out[30]=
$$\frac{\left(1+KA+d_{rel}+KA\,S_{rel}-\sqrt{8\,(1+d_{rel})^2\,RAF_{rel}+(1+KA+d_{rel}+KA\,S_{rel})^2}\right)^2}{8\,(1+d_{rel})^3\,RAF_{rel}}$$

Out[31]=
$$\frac{\left(E4-\sqrt{E2+E4^2}\right)^2}{E2\,(1+d_{rel})}$$

Out[∘]=
$$\frac{\left(1+KA+d_{rel}+KA\,S_{rel}-\sqrt{8\,(1+d_{rel})^2\,RAF_{rel}+(1+KA+d_{rel}+KA\,S_{rel})^2}\right)^2}{8\,(1+d_{rel})^3\,RAF_{rel}} \quad (15)$$

Out[∘]=
$$\frac{\left(E4-\sqrt{E2+E4^2}\right)^2}{E2\,(1+d_{rel})} \quad (16)$$

Expression 16 is very close to expression 2 with E4=E1 + $K_A S_{\mathrm{rel}}$ where $S_{\mathrm{rel}} = \frac{\mathrm{STOT}}{K_S}$. To establish parameter regions which show drug induced activation, we find the positive roots of the first derivative of the expression 16.

In[32]:=
```
RafDimers[eqns_] := (eqns[[4]] + eqns[[6]] + eqns[[7]]);
fnDimers = FullSimplify[SimplifyPars[(RafDimers[vars]/RAF /. rep22 /. sol22A) /. repratios]]
SimplifyPars[fnDimers/fnActiveRAF1433CAS]
```

Out[33]=
$$\frac{\left(-Kd\,(1+KA+d_{rel}+KA\,S_{rel})+\sqrt{8\,(Kd+Kd\,d_{rel})^2\,RAF_{rel}+Kd^2\,(1+KA+d_{rel}+KA\,S_{rel})^2}\right)^2}{16\,Kd^2\,(1+d_{rel})^2\,RAF_{rel}}$$

Out[34]=
$$\frac{1}{2}\,(1+d_{rel})$$

Out[∘]=
$$\frac{\left(-Kd\,(1+KA+d_{rel}+KA\,S_{rel})+\sqrt{8\,(Kd+Kd\,d_{rel})^2\,RAF_{rel}+Kd^2\,(1+KA+d_{rel}+KA\,S_{rel})^2}\right)^2}{16\,Kd^2\,(1+d_{rel})^2\,RAF_{rel}} \quad (17)$$

Out[∘]=
$$\frac{1}{2}\,(1+d_{rel}) \quad (18)$$

2.2. Baseline signaling in the absence of drug

```
baselineActiveRAFCAS = fnActiveRAF1433CAS /. d_rel → 0 ; (*RAF activity in absence of drug*)
rep221 = {RAF_rel → E5 (1 + KA + KA S_rel)^2/8};
fnActiveRAFCASnd1 = SimplifyPars[(baselineActiveRAFCAS //. rep221)];
d2ND = SimplifyPars[D[fnActiveRAFCASnd1, E5]];
```

Out[•]=

$$\frac{\left(1 + KA + KA\,S_{rel} - \sqrt{8\,RAF_{rel} + (1 + KA + KA\,S_{rel})^2}\right)^2}{8\,RAF_{rel}} \quad (19)$$

Out[•]=

$$\left\{RAF_{rel} \to \frac{1}{8}\,E5\,(1 + KA + KA\,S_{rel})^2\right\} \quad (20)$$

Out[•]=

$$\frac{\left(-1 + \sqrt{1 + E5}\right)^2}{E5} \quad (21)$$

Out[•]=

$$\frac{\left(-1 + \sqrt{1 + E5}\right)^2}{E5^2\,\sqrt{1 + E5}} \quad (22)$$

The derivative of baseline signaling as a function of E5 is positive definite. Therefore, the **baseline signaling increases** monotonically with **E5.** Since E5 is monotonically proportional to RAF concentration, **baseline signaling** is **directly related** to **[RAF].**
As **E5 is inversely proportional to [14-3-3]** and **KA** also with a monotonic relationship, the **baseline activity** is also **inversely related to KA and to [14-3-3]** monotonically.

2.3. Conditions on parameter regions for activation in response to the drug

In[•]:=
```
eqnsconsrv[[2]] (*same as base model.*)
```

Out[•]=

$$d + \frac{A\,d}{Kd} + \frac{2\,A^2\,d\,(d + Kd)}{Kd^2\,Kdim} == DTOT \quad (23)$$

```
dfn2 = SimplifyPars[D[fnActiveRAF1433CAS, d_rel]];
```

Out[•]=

$$\frac{1}{8\,(1 + d_{rel})^4\,RAF_{rel}}\left(1 + KA + d_{rel} + KA\,S_{rel} - \sqrt{8\,(1 + d_{rel})^2\,RAF_{rel} + (1 + KA + d_{rel} + KA\,S_{rel})^2}\right)\left(2\,(1 + d_{rel})\left(1 - \frac{1 + KA + d_{rel} + 8\,(1 + d_{rel})\,RAF_{rel} + KA\,S_{rel}}{\sqrt{8\,(1 + d_{rel})^2\,RAF_{rel} + (1 + KA + d_{rel} + KA\,S_{rel})^2}}\right) - 3\left(1 + KA + d_{rel} + KA\,S_{rel} - \sqrt{8\,(1 + d_{rel})^2\,RAF_{rel} + (1 + KA + d_{rel} + KA\,S_{rel})^2}\right)\right) \quad (24)$$

```
zeroes = SimplifyPars[Solve[dfn2 == 0, d_rel, VerifySolutions → True]];
```

Out[•]=

$$\left\{\left\{d_{rel} \to -\frac{1 + KA + 8\,RAF_{rel} + KA\,S_{rel} + 2\,KA\,\sqrt{1 + 6\,RAF_{rel}}\,\,(1 + S_{rel})}{1 + 8\,RAF_{rel}}\right\},\left\{d_{rel} \to -\frac{1 + KA + 8\,RAF_{rel} + KA\,S_{rel} - 2\,KA\,\sqrt{1 + 6\,RAF_{rel}}\,\,(1 + S_{rel})}{1 + 8\,RAF_{rel}}\right\}\right\} \quad (25)$$

The first solution is negative definite. Below, we derive the rules which allow the second solution to be positive

In[•]:=
```
exist = FullSimplify[SimplifyPars[Reduce[((d_rel /. zeroes[[2]]) > 0) && (RAF_rel > 0)]]];
```

Out[ ]=

$$(-1 + KA + KA\, S_{rel})\, (1 + 3\, KA + 3\, KA\, S_{rel}) > 8\, RAF_{rel}\ \&\&\ KA + KA\, S_{rel} > 1 \quad (26)$$

We check that the second derivative is negative at the critical point in expression

```
d2fn2 = SimplifyPars[D[dfn2, d_rel]];
d2fn2z = SimplifyPars[d2fn2 /. zeroes[[2]]];
Reduce[(d2fn2z < 0) && (KA >= 0) && (S_rel > 0)]
```

Out[ ]= $KA > 0\ \&\&\ RAF_{rel} > 0\ \&\&\ S_{rel} > 0$

Out[ ]=

$$KA > 0\ \&\&\ RAF_{rel} > 0\ \&\&\ S_{rel} > 0 \quad (27)$$

Note that no additional constraints are added on the model parameter space. Hence, expression 25 represents all of the constraints which allow for this model to produce a paradoxical activation.

- **2.4. Monotonic Relationship between total and unbound drug.**

```
FullSimplify[eqnsconsrv[[2]]];
```

Out[ ]=

$$d + \frac{A\, d}{Kd} + \frac{2\, A^2\, d\, (d + Kd)}{Kd^2\, Kdim} == DTOT \quad (28)$$

```
sd21 = FullSimplify[SimplifyPars[D[A*d /. sol22A, d]]];
rd21 = Reduce[sd21 < 0];
rd21s = SimplifyPars[SimplifyPars[rd21]];
```

Consider the first component of the outermost OR condition: KA cannot be negative, therefore, Kd<=d. However, in that case, in the second condition Kd>d or sum-of-positive-values<=0, both of which are false. Therefore the first condition is always false.

Consider second component of outermost OR condition: when Kd>d, KA<~1/d-Kd which means KA is negative which is a contradiction. Therefore, for positive values of parameters and concentrations none of the set of conditions can be satisfied. Hence by contradiction, sd21>=0

```
sd22 = SimplifyPars[D[Simplify[A^2 d (d + Kd) /. sol22A], d]]
rd22 = Reduce[sd22[[-1]] > 0];
SimplifyPars[rd22]
```

$$Kd \le 2\, d\ \&\&\ Kdim < -\frac{8\, (d + Kd)^2\, Ksm^2\, RAF}{(d\, Ksm + Kd\, (Ksm + KA\, Ksm + KA\, STOT))^2} \quad (29)$$

We split the derivative of second expression of DTOT (eqn 30) into factor product terms. The latter, more complex term is positive when the relationship in equation 31 is satisfied. Since Kdim>0, the second condition in eqn 31 cannot be satisfied therefore, this term in expression 30, of sd22 is never positive. The first bracket in eqn 30 is of the form $x - \text{Sqrt}(1 + x^2)$ which is also negative definite. Hence the product of two negative definite functions is positive definite. and sd22 is therefore positive definite function and it’s integral is a monotonic proportional function of d.

D[DTOT,d]=1+sd11+sd12 which is a positive definite function. Therefore DTOT is a monotonic increasing function of d.

Numerically :

```
fnDTOT = SimplifyPars[eqnsconsrv[[2]][[1]] /. rep22 /. sol22A];
```

```
Sty[x_] := Style[x, 20, FontFamily → "Arial"];
parset01 = {KA → 2., Kdim → 1. × 10^-7 , Ksm → 10^-7, STOT → 2 × 10^-6};
KTarr = {1. × 10^-6, 1. × 10^-7, 1. × 10^-8};
kdarr = {Kd → 1. × 10^-7 , Kd → 1. × 10^-9};
pltfn = Flatten[Table[fnDTOT /. parset01 /. kd, {kd, kdarr}, {RAF, KTarr}]];
ps = {Blue, Green, Directive[Red, Thick], Directive[Blue, Dashed],
   Directive[Green, Dashed], Directive[Red, Dashed, Thick]};
LogLogPlot[pltfn, {d, 10^-9, 10^-5}, Frame → True, FrameTicksStyle → 20, PlotStyle → ps,
 PlotLegends → Placed[LineLegend[ {Blue, Green, Directive[Red, Thick]}, Sty /@ KTarr,
    LegendLabel → Sty["RAF(M)"], LegendFunction → Panel, LabelStyle → 12], {Right, Bottom}],
 FrameLabel → (Sty /@ {"Unbound Drug  [d]  (M)", "Total Drug  [Drug]  (M)"}),
 ImageSize → {500}, FrameStyle → Thickness[0.004],
 PlotLabel → Sty["Kd: —100nM  - -1nM KA:2 KS:100nM  [14-3-3]:2μM"]
 (*N[TableForm[parset01,TableDirections→Row]]*)]
```

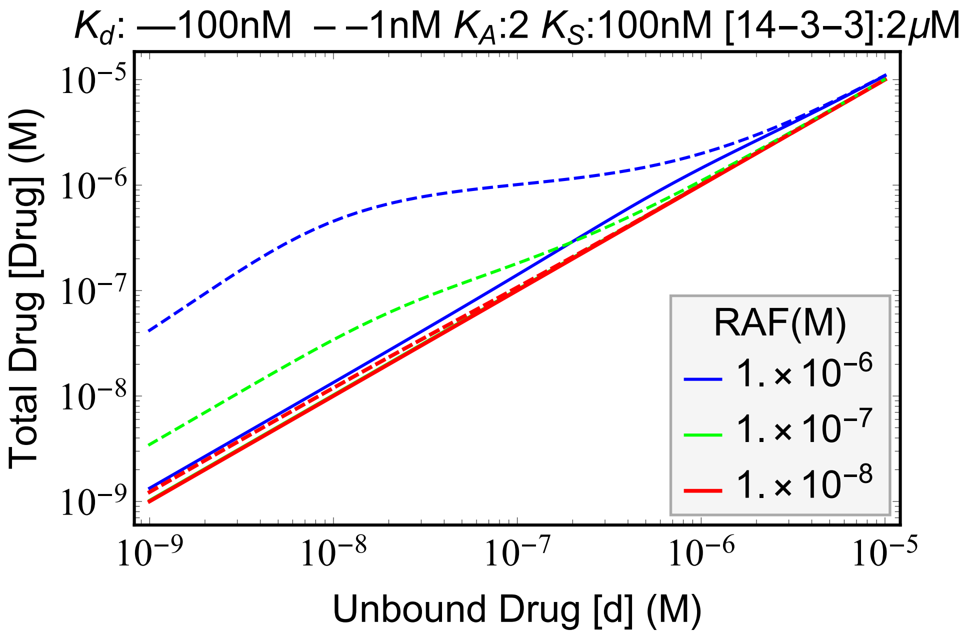

2.5. Analytic Expressions for maximum Fold Change (FC)

Fold change is defined as the ratio between maximum RAF activity (no/conc of active protomers) to that in absence of drug

```
maxActiveRAFCAS = FullSimplify[SimplifyPars[fnActiveRAF1433CAS /. zeroes[[2]]]];
rafFCCAS = SimplifyPars[maxActiveRAFCAS / baselineActiveRAFCAS]
```

Out[ ]=
$$\frac{8\left(-1+\sqrt{1+6\,RAF_{rel}}+RAF_{rel}\left(-9+6\sqrt{1+6\,RAF_{rel}}\right)\right)}{27\,KA\,(1+S_{rel})\left(1+KA+KA\,S_{rel}-\sqrt{8\,RAF_{rel}+(1+KA+KA\,S_{rel})^2}\right)^2}$$

In[ ]:= `{maxActiveRAFCAS, baselineActiveRAFCAS}`

Out[ ]=
$$\left\{\frac{-1+\sqrt{1+6\,RAF_{rel}}+RAF_{rel}\left(-9+6\sqrt{1+6\,RAF_{rel}}\right)}{27\,KA\,RAF_{rel}\,(1+S_{rel})},\ \frac{\left(1+KA+KA\,S_{rel}-\sqrt{8\,RAF_{rel}+(1+KA+KA\,S_{rel})^2}\right)^2}{8\,RAF_{rel}}\right\}$$

When the 'exist' conditions derived above to qualify for existence of PA within this model are satisfied, we can evaluate the functional dependence of the raf fold change expression on RAF_{rel} and KA:

In[ ]:=
```
dRAFrafFC = SimplifyPars[D[rafFCCAS, RAFrel]];
dKArafFC = SimplifyPars[D[rafFCCAS, KA]];
dSrafFC = SimplifyPars[D[rafFCCAS, Srel]];
SimplifyPars[Simplify[Reduce[dRAFrafFC < 0], exist]]
SimplifyPars[Simplify[Reduce[dKArafFC > 0], exist]]
SimplifyPars[Simplify[Reduce[dSrafFC > 0], exist]]
```

Out[ ]= True

Out[ ]= True

Out[ ]= True

Hence, first derivative of the fold change expression relative to KA and S_{rel} is always positive when the model displays PA and the first derivative relative to RAF rel always negative.
In other words, an equilibrium that more and more favors the inactive state (increasing KA or 14-3-3), the fold change continues to increase as a function of KA. And as the concentration of RAF increases or it's dimerization rate increases (Kdim reduces), the fold change relative to baseline continues to reduce.

- **2.6. Convert to Python**

Section 3. 14-3-3 proteins only stabilize Dimer state (DS)

In[ ]:=
```
(*Restart kernel to prevent previous model variables to leak into following results*)
Quit[]
```

- **3.1. Analytic solutions of the model**

As for the other models, we define the equilibrium rates constrained by using the principle of detailed balance as replacement rules 'rep'. We then calculate conservation equations for total RAF and total Drug as a function of unbound drug, unbound RAF concentrations and model parameters.

In[35]:=
```
vars = {a, A, d, AA, Ad, AAd, AdAd, 0, AAs, AAsd, AAsdd, s};
repcycles = {AA → A^2/Kdim, AAd → 2 A^2 d/(Kdim Kd), AdAd → A^2 d^2/(Kdim Kd^2),
   Ad → A d/Kd, as → 0, AAs → A^2 s/(Kdim KsD), AAsd → 2 A^2 s d/(Kdim KsD Kd), AAsdd → A^2 d^2 s/(Kdim KsD Kd^2)};
repc1 = {a → A KA};
repcycles = Join[repcycles /. repc1, repc1];
rep23 = Join[repcycles /. repc1, repc1];
Consrv[eqns_] := {Simplify[eqns[[1]] + eqns[[2]] + eqns[[5]] + eqns[[8]] +
     2 (eqns[[4]] + eqns[[6]] + eqns[[7]] + eqns[[9]] + eqns[[10]] + eqns[[11]])],
   Simplify[eqns[[3]] + eqns[[5]] + eqns[[6]] + eqns[[10]] + 2 (eqns[[7]] + eqns[[11]])],
   Total[eqns[[8 ;; 12]]]};
RafActivity[vars_] := 2 (vars[[4]] + vars[[9]]) + vars[[6]] + vars[[10]]
  (*/(consrv[vars][[1]])*);
eqnsconsrv = Thread[Simplify[Consrv[vars] /. rep23] == {RAF, DTOT, STOT}]
```

Out[42]=
$$\left\{\frac{A\left(Kd\ (d+Kd+KA\ Kd)\ Kdim\ KsD+2\,A\ (d+Kd)^2\ (KsD+s)\right)}{Kd^2\ Kdim\ KsD}==RAF,\right.$$
$$\left. d+\frac{A\,d}{Kd}+\frac{2\,A^2\,d\ (d+Kd)\ (KsD+s)}{Kd^2\ Kdim\ KsD}==DTOT,\ \frac{\left(A^2\ (d+Kd)^2+Kd^2\ Kdim\ KsD\right)\,s}{Kd^2\ Kdim\ KsD}==STOT\right\}$$

Out[ ]=

$$\left\{\frac{A\left(Kd\,(d+Kd+KA\,Kd)\,Kdim\,KsD+2\,A\,(d+Kd)^2\,(KsD+s)\right)}{Kd^2\,Kdim\,KsD}==RAF,\right.$$
$$\left.d+\frac{A\,d}{Kd}+\frac{2\,A^2\,d\,(d+Kd)\,(KsD+s)}{Kd^2\,Kdim\,KsD}==DTOT,\ \frac{\left(A^2\,(d+Kd)^2+Kd^2\,Kdim\,KsD\right)s}{Kd^2\,Kdim\,KsD}==STOT\right\} \tag{30}$$

In[43]:=
```
SimplifyPars[x_] := Simplify[x, {Kd > 0, RAF > 0, DTOT > 0,
    d_rel > 0, RAF_rel > 0, Kdim > 0, KA > 0, d > 0, KsD > 0, s_rel > 0, s > 0, STOT > 0.}];
numsol23As = Quiet[Solve[eqnsconsrv[[{1, 3}]] /. baseparams, {A, s}][[4]]];
(*created to plot example numerical solution*)
sol23A = FullSimplify[SimplifyPars[Solve[eqnsconsrv[[1]], A][[2]]]]
  (*the first solution is negtive definite and second is positive.*)
```

Out[45]=
$$\left\{A\to\frac{Kd\,Kdim\,KsD\left(-d-Kd-KA\,Kd+\frac{1}{\sqrt{\frac{Kdim\,KsD}{(d+Kd+KA\,Kd)^2\,Kdim\,KsD+8\,(d+Kd)^2\,KsD\,RAF+8\,(d+Kd)^2\,RAF\,s}}}\right)}{4\,(d+Kd)^2\,(KsD+s)}\right\}$$

Out[ ]=
$$\left\{A\to\frac{Kd\,Kdim\,KsD\left(-d-Kd-KA\,Kd+\frac{1}{\sqrt{\frac{Kdim\,KsD}{(d+Kd+KA\,Kd)^2\,Kdim\,KsD+8\,(d+Kd)^2\,KsD\,RAF+8\,(d+Kd)^2\,RAF\,s}}}\right)}{4\,(d+Kd)^2\,(KsD+s)}\right\} \tag{31}$$

In[46]:=
```
sol23s = SimplifyPars[Solve[eqnsconsrv[[3]], s]][[1]]
```

Out[46]=
$$\left\{s\to\frac{Kd^2\,Kdim\,KsD\,STOT}{A^2\,(d+Kd)^2+Kd^2\,Kdim\,KsD}\right\}$$

Out[ ]=
$$\left\{s\to\frac{Kd^2\,Kdim\,KsD\,STOT}{A^2\,(d+Kd)^2+Kd^2\,Kdim\,KsD}\right\} \tag{32}$$

In[47]:=
```
fnActiveRAF1433DS1 = FullSimplify[SimplifyPars[RafActivity[vars] / RAF /. rep23 /. sol23A]];
repratios = {Kdim → (RAF / RAF_rel), d → (d_rel Kd), s → s_rel KsD};
(*define concentrations relative to equilibrium rate constants*)
fnActiveRAF1433DS = FullSimplify[SimplifyPars[fnActiveRAF1433DS1 /. repratios]]
FullSimplify[
 SimplifyPars[fnActiveRAF1433DS /. {RAF_rel → E6 / 8 / (1 + d_rel)^2 / (1 + s_rel), KA → (E1 - d_rel - 1)}]]
```

Out[49]=
$$\frac{\left(1+KA+d_{rel}-\sqrt{(1+KA)^2+8\,RAF_{rel}+d_{rel}\,(2+2\,KA+d_{rel}+8\,(2+d_{rel})\,RAF_{rel})+8\,(1+d_{rel})^2\,RAF_{rel}\,s_{rel}}\right)^2}{8\,(1+d_{rel})^3\,RAF_{rel}\,(1+s_{rel})}$$

Out[50]=
$$\frac{\left(E1-\sqrt{E1^2+E6}\right)^2}{E6\,(1+d_{rel})}$$

Out[ ]=
$$\frac{\left(1+KA+d_{rel}-\sqrt{(1+KA)^2+8\,RAF_{rel}+d_{rel}\,(2+2\,KA+d_{rel}+8\,(2+d_{rel})\,RAF_{rel})+8\,(1+d_{rel})^2\,RAF_{rel}\,s_{rel}}\right)^2}{8\,(1+d_{rel})^3\,RAF_{rel}\,(1+s_{rel})} \tag{33}$$

Out[ ]=
$$\frac{\left(E1-\sqrt{E1^2+E6}\right)^2}{E6\,(1+d_{rel})} \tag{34}$$

Above is very close to the corresponding expression from Section 1 with E6 = E2(1 + s_{rel}) where $s_{rel}=\frac{[s]}{K_{SD}}$. To establish parameter regions which show drug induced activation, we find the positive roots of the first derivative of the expression 34.

In[51]:=
```
RafDimers[eqns_] := (eqns[[4]] + eqns[[6]] + eqns[[7]] + eqns[[9]] + eqns[[10]] + eqns[[11]]);
fnDimers = FullSimplify[SimplifyPars[(RafDimers[vars] / RAF /. rep23 /. sol23A) /. repratios]]
fnDimers / fnActiveRAF1433DS
```

Out[52]=

$$\frac{\left(1+KA+d_{rel}-\sqrt{(1+KA)^2+8\,RAF_{rel}+d_{rel}\,(2+2\,KA+d_{rel}+8\,(2+d_{rel})\,RAF_{rel})+8\,(1+d_{rel})^2\,RAF_{rel}\,s_{rel}}\right)^2}{16\,(1+d_{rel})^2\,RAF_{rel}\,(1+s_{rel})}$$

Out[53]=

$$\frac{1}{2}\,(1+d_{rel})$$

Out[ ]=

$$\frac{\left(1+KA+d_{rel}-\sqrt{(1+KA)^2+8\,RAF_{rel}+d_{rel}\,(2+2\,KA+d_{rel}+8\,(2+d_{rel})\,RAF_{rel})+8\,(1+d_{rel})^2\,RAF_{rel}\,s_{rel}}\right)^2}{16\,(1+d_{rel})^2\,RAF_{rel}\,(1+s_{rel})} \quad (35)$$

Out[ ]=

$$\frac{1}{2}\,(1+d_{rel}) \quad (36)$$

■ 3.2. Baseline Signaling

```
baselineActiveRAFDS = SimplifyPars[fnActiveRAF1433DS /. d_rel → 0];
baselineActiveRAFDS1 = SimplifyPars[baselineActiveRAFDS /. RAF_rel → E7 (1 + KA)^2 / (1 + s_rel) / 8];
d3ND = SimplifyPars[D[baselineActiveRAFDS1, E7]];
```

Out[ ]=

$$\frac{\left(-1+\sqrt{1+E7}\right)^2}{E7} \quad (37)$$

Out[ ]=

$$\frac{\left(-1+\sqrt{1+E7}\right)^2}{E7^2\,\sqrt{1+E7}} \quad (38)$$

The derivative relative to E7 of baseline activity is a positive definite function. That is baseline activity is an increasing function of E7. However, E7 is directly proportional to RAF and s_{rel} and therefore baseline activity is an increasing function of [RAF] and unbound 14-3-3. And therefore of 14-3-3.

Also, E7 is inversely proportional to KA and therefore is a reducing function of KA.

▫ *Relationship between unbound and total 14-3-3:*

Unbound vs total 14 − 3 − 3

```
STOTeqn = SimplifyPars[eqnsconsrv[[3]] /. sol23A /. d → 0];
SimplifyPars[Simplify[STOTeqn /. {Kdim Ks1 / ((1 + KA)^2 Kdim Ks1 + 8 RAF (Ks1 + s)) → 1 / (C1^2 (1 + KA)^2)}, C1 > 0]];
STOTd = SimplifyPars[D[STOTeqn[[1]], s]];
STOTdsimp = FullSimplify[
   SimplifyPars[STOTd /. {Kdim Ks1 / ((1 + KA)^2 Kdim Ks1 + 8 RAF (Ks1 + s)) → 1 / (C1^2 (1 + KA)^2)}], C1 > 0];
```

Out[ ]=

$$\frac{1}{16}\,s\left(16+\frac{(-1+C1)^2\,(1+KA)^2\,Kdim\,Ks1}{(Ks1+s)^2}\right)==STOT \quad (39)$$

$$\frac{1}{16}\left(16-\frac{2\,\text{Kdim}\,\text{Ks1}\,s\left(1+\text{KA}-\frac{1}{\sqrt{\frac{\text{Kdim}\,\text{Ks1}}{(1+\text{KA})^2\,\text{Kdim}\,\text{Ks1}+8\,\text{RAF}\,(\text{Ks1}+s)}}}\right)^2}{(\text{Ks1}+s)^3}+\frac{\text{Kdim}\,\text{Ks1}\left(1+\text{KA}-\frac{1}{\sqrt{\frac{\text{Kdim}\,\text{Ks1}}{(1+\text{KA})^2\,\text{Kdim}\,\text{Ks1}+8\,\text{RAF}\,(\text{Ks1}+s)}}}\right)^2}{(\text{Ks1}+s)^2}-\frac{8\,\text{RAF}\,s\left(-1+\sqrt{\frac{\text{Kdim}\,\text{Ks1}}{(1+\text{KA})^2\,\text{Kdim}\,\text{Ks1}+8\,\text{RAF}\,(\text{Ks1}+s)}}+\text{KA}\sqrt{\frac{\text{Kdim}\,\text{Ks1}}{(1+\text{KA})^2\,\text{Kdim}\,\text{Ks1}+8\,\text{RAF}\,(\text{Ks1}+s)}}\right)}{(\text{Ks1}+s)^2}\right)$$

$$\frac{1}{16}\left(16+\frac{(-1+\text{C1})^2\,(1+\text{KA})^2\,\text{Kdim}\,\text{Ks1}\,(\text{Ks1}-s)}{(\text{Ks1}+s)^3}+\frac{8\,(-1+\text{C1})\,\text{RAF}\,s}{\text{C1}\,(\text{Ks1}+s)^2}\right) \tag{41}$$

Is C1>1? Yes. Always. Since the expression is $\frac{\text{numerator+positive number}}{\text{numerator}}$.

```
repC1 = Simplify[Solve[Kdim Ks1 / ((1 + KA)^2 Kdim Ks1 + 8 RAF (Ks1 + s)) == 1/(C1^2 (1 + KA)^2), C1][[2]]]
SimplifyPars[Reduce[(C1 /. repC1) > 1]]
```

$$\left\{\text{C1}\to\frac{\sqrt{(1+\text{KA})^2\,\text{Kdim}\,\text{Ks1}+8\,\text{RAF}\,(\text{Ks1}+s)}}{\sqrt{(1+\text{KA})^2\,\text{Kdim}\,\text{Ks1}}}\right\} \tag{42}$$

Out[∘]= True

Therefore, when KS1>s the derivative of STOT relative to s is definitely positive. This is because the only negative terms may come from (C1-1) which has been shown positive and (Ks1-s).

When KS1<s can STOTd function be negative?

```
In[∘]:= cond10 = Reduce[STOTd < 0];
cond10s = SimplifyPars[cond10]
```

$$2\,\text{Ks1}<s\ \&\&\ \text{RAF}+6\,s+4\sqrt{2}\,\sqrt{s\,(-\text{Ks1}+s)}<2\,\text{Ks1}\ \&\&\ \frac{1}{2\,(1+\text{KA})^2\,\text{Ks1}\,(\text{Ks1}-s)}\left(-4\,\text{Ks1}^3-12\,\text{Ks1}^2\,(\text{RAF}+s)+s\left(\text{RAF}^2+8\,\text{RAF}\,s-4\,s^2\right)-\text{Ks1}\left(\text{RAF}^2+4\,\text{RAF}\,s+12\,s^2\right)+\frac{1}{\text{Abs}[\text{Ks1}-s]}(-\text{Ks1}+s)\sqrt{4\,\text{Ks1}^2-4\,\text{Ks1}\,\text{RAF}+\text{RAF}^2+8\,\text{Ks1}\,s+12\,\text{RAF}\,s+4\,s^2}\ \text{Abs}\left[2\,\text{Ks1}^2-\text{Ks1}\,(\text{RAF}-4\,s)+s\,(\text{RAF}+2\,s)\right]\right)<\text{Kdim}\ \&\&\ \text{Kdim}<\frac{1}{2\,(1+\text{KA})^2\,\text{Ks1}\,(\text{Ks1}-s)}\left(-4\,\text{Ks1}^3-12\,\text{Ks1}^2\,(\text{RAF}+s)+s\left(\text{RAF}^2+8\,\text{RAF}\,s-4\,s^2\right)-\text{Ks1}\left(\text{RAF}^2+4\,\text{RAF}\,s+12\,s^2\right)+\frac{1}{\text{Abs}[\text{Ks1}-s]}(\text{Ks1}-s)\sqrt{4\,\text{Ks1}^2-4\,\text{Ks1}\,\text{RAF}+\text{RAF}^2+8\,\text{Ks1}\,s+12\,\text{RAF}\,s+4\,s^2}\ \text{Abs}\left[2\,\text{Ks1}^2-\text{Ks1}\,(\text{RAF}-4\,s)+s\,(\text{RAF}+2\,s)\right]\right) \tag{43}$$

When is the second condition satisfied? Under the global condition that Ks1<s, the minimum value of s is Ks1.

```
In[∘]:= solcond10s2 = cond10s[[2]] /. s → Ks1
```

$$6\,\text{Ks1}+\text{RAF}<2\,\text{Ks1} \tag{44}$$

Not satisfied at s=Ks1. Being a sum of positive terms, the inequality will not be satisfied for any value of s s.t. s>Ks1.

Hence, by contradiction, Unbound [14-3-3] is a monotonic increasing function of total [14-3-3] in the DS model.

3.3. Conditions on parameter regions for activation in response to the drug

Unbound 14-3-3 is a slowly varying function of drug compared to active kinase

```
dAKbyd = D[SimplifyPars[RafActivity[vars] / RAF /. rep23] /. {s → s[d], A → A[d]}, d]
dAKbyd[[-1]]
dAKbyd[[;; -2]]
```

$$\frac{2\,A[d]^2\,(KsD + s[d])}{Kd\,Kdim\,KsD\,RAF} + \frac{4\,(d + Kd)\,A[d]\,(KsD + s[d])\,A'[d]}{Kd\,Kdim\,KsD\,RAF} + \frac{2\,(d + Kd)\,A[d]^2\,s'[d]}{Kd\,Kdim\,KsD\,RAF}$$

$$\frac{2\,(d + Kd)\,A[d]^2\,s'[d]}{Kd\,Kdim\,KsD\,RAF}$$

$$\frac{2\,A[d]^2\,(KsD + s[d])}{Kd\,Kdim\,KsD\,RAF} + \frac{4\,(d + Kd)\,A[d]\,(KsD + s[d])\,A'[d]}{Kd\,Kdim\,KsD\,RAF}$$

In[ ]:=
```
dsbyd = s'[d] -> SimplifyPars[D[s /. sol3s /. A → A[d], d]]
```

Out[ ]=
$$s'[d] \to -\frac{2\,Kd^2\,(d + Kd)\,Kdim\,KsD\,STOT\,A[d]\,(A[d] + (d + Kd)\,A'[d])}{\left(Kd^2\,Kdim\,KsD + (d + Kd)^2\,A[d]^2\right)^2}$$

In[ ]:=
```
FullSimplify[Simplify[dAKbyd[[-1]] / dAKbyd[[;; -2]]] /. dsbyd]
```

Out[ ]=
$$-\frac{2\,Kd^2\,(d + Kd)^2\,Kdim\,KsD\,STOT\,A[d]^2\,(A[d] + (d + Kd)\,A'[d])}{\left(Kd^2\,Kdim\,KsD + (d + Kd)^2\,A[d]^2\right)^2\,(KsD + s[d])\,(A[d] + 2\,(d + Kd)\,A'[d])}$$

while deriving the full set of conditions isn't possible without working with full solution for [s] and [A] which are unwieldy expressions. It is possible to derive a solution under the limit of slowly varying $s[d_{rel}]$ such that $dsrel/dd_{rel}$=0. So we first establish that the unbound 14-3-3 only slowly varies as a function

```
parset01 = {KA → 10., Kdim → 1. × 10^-7, KsD → 10^-7, STOT → 2 × 10^-6};
sol3SA = Solve[eqnsconsrv[[{1, 3}]], {A, s}];
sol3SA /. parset01 /. {Kd -> 0.1, d -> 0.1, RAF -> 4 × 10^-8}
```

Out[ ]=
$$\{\{A \to -7.12868\times10^{-8} - 2.14205\times10^{-7}\,i,\ s \to -7.98046\times10^{-8} - 6.36318\times10^{-8}\,i\},$$
$$\{A \to -7.12868\times10^{-8} + 2.14205\times10^{-7}\,i,\ s \to -7.98046\times10^{-8} + 6.36318\times10^{-8}\,i\},$$
$$\{A \to -9.90312\times10^{-9},\ s \to 1.9245\times10^{-6}\},\ \{A \to 2.47662\times10^{-9},\ s \to 1.99511\times10^{-6}\}\}$$

```
Sty[x_] := Style[x, "Arial", 26];
dAK = D[fnActiveRAFDS1 /. sol3SA[[4]], d];
repSA = {A'[d] → D[A /. sol3SA[[4]], d]};
sd = D[s /. sol3SA[[4]], d];
lpdats =
  Table[{d1, ((dAKbyd[[-1]] /. A[d] → A) /. {s'[d] -> N[sd /. baseparams /. d → d1]} /. repSA) /.
      sol3SA[[4]] /. baseparams /. d → d1}, {d1, Table[10^i, {i, -9, -3, 0.5}]}];
lpdatAK = Table[{d1, N[dAK /. baseparams /. d → d1]}, {d1, Table[10^i, {i, -9, -3, 0.5}]}];
lpdatAK2sratio =
  Table[{lpdats[[i, 1]], lpdatAK[[i, 2]] / lpdats[[i, 2]]}, {i, 1, Length[lpdats]}];
```

In[ ]:=
```
ListLogLinearPlot[{lpdats, lpdatAK}, Joined -> True, PlotLegends -> {"ds part of dAK", "dAK"},
  PlotRange → {-1, 1}, AxesLabel → {"Drug Concentration (μM)"}]
```

Out[ ]=

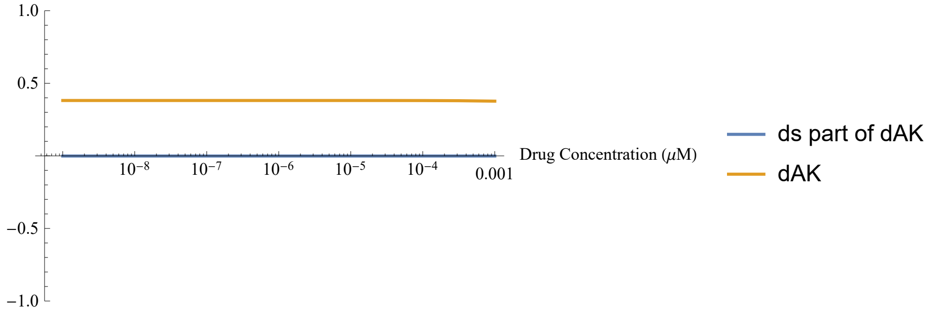

In[ ]:=
```
ListLogLinearPlot[lpdatAK2sratio, Joined → True, PlotLegends → "dAK/(ds part of dAK)",
  PlotRange → {-400, 0}, AxesLabel → {"Drug Concentration (μM)"}]
```

Out[ ]=

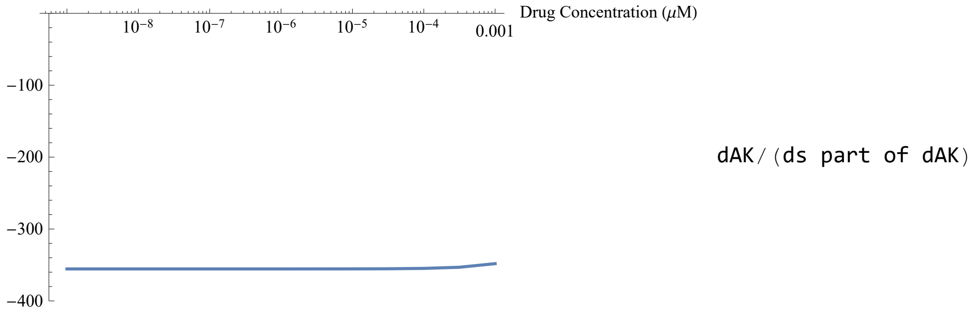

Hence, for all of the relevant range of drug concentrations the total derivative of active kinase relative drug is in far excess of the term which includes derivate of 14-3-3 relative to drug. This also makes sense since the drug does not directly bind with 14-3-3 and 14-3-3 is affected by the indirect change in the dimer concentrations. Under the consideration that 14-3-3 changes slowly, the following expressions can be derived.

PA Conditions

```
dfn3 = FullSimplify[SimplifyPars[D[fnActiveRAF1433DS, drel]]]
```

Out[ ]=

$$\frac{1}{8\,(1+d_{rel})^4\,RAF_{rel}\,(1+s_{rel})}\left(1+KA+d_{rel}-\sqrt{(1+KA)^2+8\,RAF_{rel}+d_{rel}\,(2+2\,KA+d_{rel}+8\,(2+d_{rel})\,RAF_{rel})+8\,(1+d_{rel})^2\,RAF_{rel}\,s_{rel}}\right)\left(-3\left(1+KA+d_{rel}-\sqrt{(1+KA)^2+8\,RAF_{rel}+d_{rel}\,(2+2\,KA+d_{rel}+8\,(2+d_{rel})\,RAF_{rel})+8\,(1+d_{rel})^2\,RAF_{rel}\,s_{rel}}\right)+2\,(1+d_{rel})\left(1+\frac{-1-KA-8\,RAF_{rel}\,(1+s_{rel})-d_{rel}\,(1+8\,RAF_{rel}\,(1+s_{rel}))}{\sqrt{(1+KA)^2+8\,RAF_{rel}+d_{rel}\,(2+2\,KA+d_{rel}+8\,(2+d_{rel})\,RAF_{rel})+8\,(1+d_{rel})^2\,RAF_{rel}\,s_{rel}}}\right)\right) \tag{45}$$

In[ ]:=
```
zeroes = SimplifyPars[Solve[dfn3 == 0, drel, VerifySolutions → True]]
```

Out[ ]=

$$\left\{\left\{d_{rel} \to -\frac{1 + KA + 8\, RAF_{rel}\, (1 + s_{rel}) + 2\, KA \sqrt{1 + 6\, RAF_{rel}\, (1 + s_{rel})}}{1 + 8\, RAF_{rel}\, (1 + s_{rel})}\right\}, \left\{d_{rel} \to -\frac{1 + KA + 8\, RAF_{rel}\, (1 + s_{rel}) - 2\, KA \sqrt{1 + 6\, RAF_{rel}\, (1 + s_{rel})}}{1 + 8\, RAF_{rel}\, (1 + s_{rel})}\right\}\right\} \quad (46)$$

The first solution is negative definite. Below, we derive the rules (expression 13) which allow the second solution to be positive

In[ ]:= **exist = SimplifyPars[Reduce[((d_rel /. zeroes[[2]]) > 0)]]**

Out[ ]=

$$3\, KA > 2\ \&\&\ 1 + 2\, KA + 8\, RAF_{rel}\, (1 + s_{rel}) < 3\, KA^2 \quad (47)$$

In[ ]:= **FullSimplify[3 KA^2 - 2 KA - 1]**

Out[ ]= $(-1 + KA)\, (1 + 3\, KA)$

Note that RHS of second condition becomes negative if KA<1. Therefore, KA>1 is automatically imposed for positive values of RAF and 14-3-3 concentrations. Hence DS role does not induce PA.

We check if the second derivative is negative at the critical point in expression 27.

d2fn3 = SimplifyPars[D[dfn3, d_rel]];

d2fn3z = SimplifyPars[d2fn3 /. zeroes[[2]]]

Out[ ]=

$$-\left(\left((1 + 8\, RAF_{rel}\, (1 + s_{rel}))^2 \left(3 + 96\, RAF_{rel}^3\, (1 + s_{rel})^3 - 3\sqrt{1 + 6\, RAF_{rel}\, (1 + s_{rel})} - 9\, RAF_{rel}\, (1 + s_{rel}) \left(-4 + 3\sqrt{1 + 6\, RAF_{rel}\, (1 + s_{rel})}\right) - 4\, RAF_{rel}^2\, (1 + s_{rel})^2 \left(-31 + 14\sqrt{1 + 6\, RAF_{rel}\, (1 + s_{rel})}\right)\right)\right) \Big/ \left(2\, KA^3\, RAF_{rel}\, (1 + s_{rel}) \left(-1 + 2\sqrt{1 + 6\, RAF_{rel}\, (1 + s_{rel})}\right)^5\right)\right) \quad (48)$$

Reduce[(d2fn3z < 0)]
SimplifyPars[%]

Out[ ]=

$$\left(RAF_{rel} < 0\ \&\&\ \left((KA > 0\ \&\&\ s_{rel} < -1)\ ||\ \left(KA < 0\ \&\&\ \left(-1 < s_{rel} < \frac{-1 - 8\, RAF_{rel}}{8\, RAF_{rel}}\ ||\ \frac{-1 - 8\, RAF_{rel}}{8\, RAF_{rel}} < s_{rel} < \frac{-1 - 6\, RAF_{rel}}{6\, RAF_{rel}}\right)\right)\right)\right)\ ||\ \left(RAF_{rel} > 0\ \&\&\ \left(\left(KA < 0\ \&\&\ \left(\frac{-1 - 6\, RAF_{rel}}{6\, RAF_{rel}} < s_{rel} < \frac{-1 - 8\, RAF_{rel}}{8\, RAF_{rel}}\ ||\ \frac{-1 - 8\, RAF_{rel}}{8\, RAF_{rel}} < s_{rel} < -1\right)\right)\ ||\ (KA > 0\ \&\&\ s_{rel} > -1)\right)\right) \quad (49)$$

Out[ ]= True

Note that no additional constraints are added on the model parameter space. Hence, existence equations above include all of the constraints which allow for this model to produce a paradoxical activation. This is an approximate result, albeit one which is validated by the more complete numerical results plot in supplementary figures.

■ 3.4. Monotonic Relationship between total and unbound drug.

In[ ]:= **eqnsconsrv[[2]]**

Out[ ]= $d + \frac{A\, d}{Kd} + \frac{2\, A^2\, d\, (d + Kd)\, (Ks1 + s)}{Kd^2\, Kdim\, Ks1} == DTOT$

Analytically:

In[∘]:= eqnDTOTandd = FullSimplify[SimplifyPars[eqnsconsrv[[2]][[1]] /. sol23A /. repratios[[{1, 3}]]]]

Out[∘]=
$$\frac{1}{4\,(d+Kd)^3\,(1+s_{rel})}\,d\left(4\,(d+Kd)^3+KA\,Kd\,(d+Kd+KA\,Kd)\,Kdim+4\,(d+Kd)^3\,s_{rel}+4\,(d+Kd)^2\,Kdim\,RAF_{rel}\,(1+s_{rel})-KA\,Kd\,Kdim\sqrt{(d+Kd+KA\,Kd)^2+8\,(d+Kd)^2\,RAF_{rel}\,(1+s_{rel})}\right)$$

In[∘]:= dexp1 = FullSimplify[SimplifyPars[D[eqnDTOTandd, d]][[-1]] /. {(d + Kd + KA Kd)^2 + 8 (d + Kd)^2 RAF_rel (1 + s_rel) → Const1^2}, Const1 > 0]

Out[∘]=
$$4\,(d+Kd)^4+\frac{KA\,Kd\,(-Const1+d+Kd+KA\,Kd)\,(Const1\,(-2\,d+Kd)-d\,(d+Kd))\,Kdim}{Const1}+4\,(d+Kd)^4\,s_{rel}+\frac{4\,(Const1-2\,d\,KA)\,Kd\,(d+Kd)^2\,Kdim\,RAF_{rel}\,(1+s_{rel})}{Const1}$$

Note:

1.All terms are additive

2.First term is quartic - positive definite

3. Third term is product of quartic and positive variable - positive definite

4. Last term, Const1 contains 2d*KA form the first term in it's expression + many more positive terms, hence Const1>2dKA always. Therefore, last term is positive definite.

5. Let us evaluate the Numerators of the terms containing Const1 more:

In[∘]:= dsubexp3 = Simplify[(KA Kd (-Const1 + d + Kd + KA Kd) (Const1 (-2 d + Kd) - d (d + Kd)) Kdim + 4 (Const1 - 2 d KA) Kd (d + Kd)^2 Kdim RAF_rel (1 + s_rel)) /. Const1 → Sqrt[(d + Kd + KA Kd)^2 + 8 (d + Kd)^2 RAF_rel (1 + s_rel)]]

Out[∘]=
$$Kd\,Kdim\left(4\,(d+Kd)^2\,RAF_{rel}\,(1+s_{rel})\left(-2\,d\,KA+\sqrt{(d+Kd+KA\,Kd)^2+8\,(d+Kd)^2\,RAF_{rel}\,(1+s_{rel})}\right)+KA\left(d+Kd+KA\,Kd-\sqrt{(d+Kd+KA\,Kd)^2+8\,(d+Kd)^2\,RAF_{rel}\,(1+s_{rel})}\right)\left(-d\,(d+Kd)+(-2\,d+Kd)\sqrt{(d+Kd+KA\,Kd)^2+8\,(d+Kd)^2\,RAF_{rel}\,(1+s_{rel})}\right)\right)$$

In[∘]:= red3 = Reduce[dsubexp3 < 0]

Out[∘]=
$RAF_{rel} \in \mathbb{R}$ && ((d < 0 && ((s_rel < -1 && (⋯1⋯)) || ((⋯1⋯) && s_rel == ⋯1⋯) || (s_rel > -1 && ((Kd < 2 d && (⋯1⋯)) || (Kd == 2 d && (⋯1⋯)) || (2 d < Kd < d && (⋯1⋯)) || (Kd == d && (⋯1⋯)) || (d < Kd < d/2 && (⋯1⋯)) || (Kd == d/2 && (⋯1⋯)) || (d/2 < Kd < 0 && (⋯1⋯)) || (0 < Kd < -d && (⋯1⋯)) || (KA < 0 && Kd == -d && Kdim < 0) || (-d < Kd < -2 d && (⋯1⋯)) || (Kd == -2 d && (⋯1⋯)) || (Kd > -2 d && (⋯1⋯)))))) || (⋯1⋯) || (⋯1⋯))

large output | show less | show more | show all | set size limit...

In[∘]:= SimplifyPars[red3]

Out[∘]= False

When all parameters and variables are positive the derivative sub expression 3 can never be negative. Therefore, derivative of total drug relative to unbound drug is a MONOTONICALLY INCREASING FUNCTION.

Numerically :

```
sol3SA = Solve[eqnsconsrv[[{1, 3}]], {A, s}];
sol3SA /. parset01 /. {Kd → 0.1, d → 0.1, RAF → 4 × 10^-8}
```

Out[•]= {{A → -7.12868 × 10^-8 - 2.14205 × 10^-7 ⅈ, s → -7.98046 × 10^-8 - 6.36318 × 10^-8 ⅈ},
{A → -7.12868 × 10^-8 + 2.14205 × 10^-7 ⅈ, s → -7.98046 × 10^-8 + 6.36318 × 10^-8 ⅈ},
{A → -9.90312 × 10^-9, s → 1.9245 × 10^-6}, {A → 2.47662 × 10^-9, s → 1.99511 × 10^-6}}

Only the fourth solution is positive for both A and s,

```
fnDTOT = eqnsconsrv[[2]][[1]] /. rep23 /. sol3SA[[4]];

Sty[x_] := Style[x, 20, FontFamily → "Arial"];
parset01 = {KA → 10., Kdim → 1. × 10^-7 , Ks1 → 10^-7, STOT → 2 × 10^-6};
KTarr = {1. × 10^-6, 1. × 10^-7, 1. × 10^-8};
kdarr = { 1. × 10^-7 , 1. × 10^-9};
pltfn = Flatten[Table[{d1, N[fnDTOT /. parset01 /. {Kd -> kd, d → d1, RAF → rt}]},
    {kd, kdarr}, {rt, KTarr}, {d1, Table[10^i, {i, -9, -5, 0.5}]}], 1];
ps = {Blue, Green, Directive[Red, Thick], Directive[Blue, Dashed],
   Directive[Green, Dashed], Directive[Red, Dashed, Thick]};
ListLogLogPlot[pltfn, Joined → True, Frame → True, FrameTicksStyle → 20, PlotStyle → ps,
 PlotLegends → Placed[LineLegend[ {Blue, Green, Directive[Red, Thick]}, Sty /@ KTarr,
    LegendLabel → Sty["RAF(M)"], LegendFunction → Panel, LabelStyle → 12], {Right, Bottom}],
 FrameLabel → (Sty /@ {"Unbound Drug  [d]  (M)", "Total Drug  [Drug]  (M)"}),
 ImageSize → {500}, FrameStyle → Thickness[0.004],
 PlotLabel → Sty["K_d: —100nM  - -1nM K_A:10 K_S:100nM [14-3-3]:2μM"]
 (*N[TableForm[parset01,TableDirections→Row]]*)]
```

Out[•]=

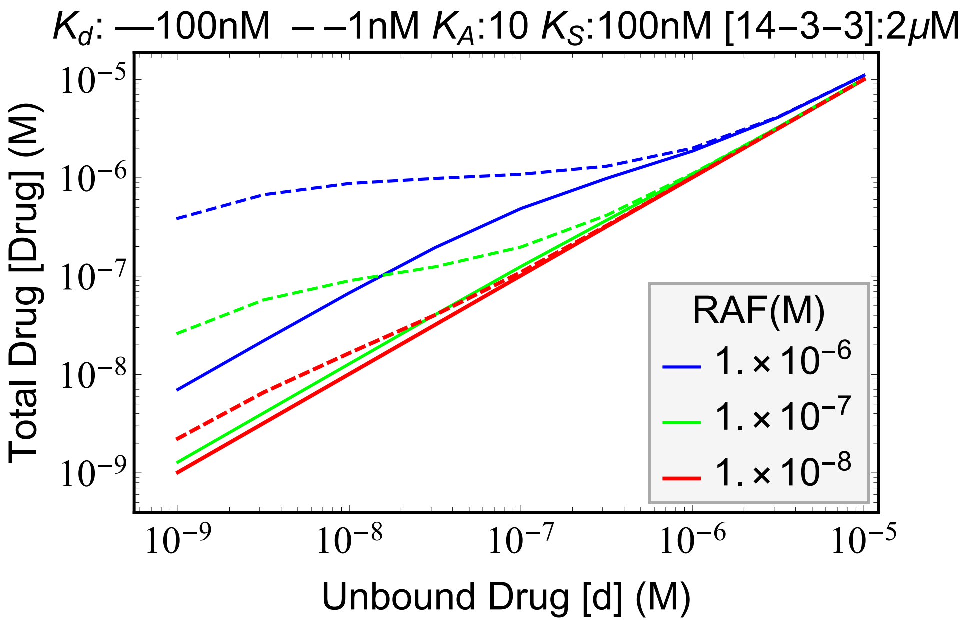

■ 3.5. Analytic Expressions for Fold Change (FC)

In[∘]:=
```
fnActiveRAFDSFC = SimplifyPars[fnActiveRAF1433DS / SimplifyPars[fnActiveRAF1433DS /. d_rel → 0]];
rep3 = {RAF_rel → E6 / 8 / (1 + d_rel)^2 / (1 + s_rel), KA → (E1 - d_rel - 1)};
rep4 = {RAF_rel → E7 (1 + KA)^2 / (1 + s_rel) / 8};
SimplifyPars[(fnActiveRAF1433DS /. rep3) / Simplify[(fnActiveRAF1433DS /. rep4) /. d_rel → 0]];
dfnActiveRAFDSFC = SimplifyPars[D[fnActiveRAFDSFC, s_rel]];
```

Out[∘]=

$$\frac{\left(1+KA+d_{rel}-\sqrt{(1+KA)^2+8\,RAF_{rel}+d_{rel}\,(2+2\,KA+d_{rel}+8\,(2+d_{rel})\,RAF_{rel})+8\,(1+d_{rel})^2\,RAF_{rel}\,s_{rel}}\right)^2}{(1+d_{rel})^3\left(1+KA-\sqrt{(1+KA)^2+8\,RAF_{rel}\,(1+s_{rel})}\right)^2} \tag{50}$$

Out[∘]=

$$\frac{\left(E1-\sqrt{E1^2+E6}\right)^2 E7}{E6\left(-1+\sqrt{1+E7}\right)^2(1+d_{rel})} \tag{51}$$

Out[∘]=

$$\left(8\,RAF_{rel}\left(1+KA+d_{rel}-\sqrt{(1+KA)^2+8\,RAF_{rel}+d_{rel}\,(2+2\,KA+d_{rel}+8\,(2+d_{rel})\,RAF_{rel})+8\,(1+d_{rel})^2\,RAF_{rel}\,s_{rel}}\right)^2\right)\Big/\left((1+d_{rel})^3\sqrt{(1+KA)^2+8\,RAF_{rel}\,(1+s_{rel})}\left(1+KA-\sqrt{(1+KA)^2+8\,RAF_{rel}\,(1+s_{rel})}\right)^3\right)-\left(8\,RAF_{rel}\left(1+KA+d_{rel}-\sqrt{(1+KA)^2+8\,RAF_{rel}+d_{rel}\,(2+2\,KA+d_{rel}+8\,(2+d_{rel})\,RAF_{rel})+8\,(1+d_{rel})^2\,RAF_{rel}\,s_{rel}}\right)\right)\Big/\left((1+d_{rel})\sqrt{(1+KA)^2+8\,RAF_{rel}+d_{rel}\,(2+2\,KA+d_{rel}+8\,(2+d_{rel})\,RAF_{rel})+8\,(1+d_{rel})^2\,RAF_{rel}\,s_{rel}}\left(1+KA-\sqrt{(1+KA)^2+8\,RAF_{rel}\,(1+s_{rel})}\right)^2\right) \tag{52}$$

In[∘]:=
```
SimplifyPars[Solve[(RAF_rel /. rep4) == RAF_rel, E7]]
```

Out[∘]=

$$\left\{\left\{E7\to\frac{8\,RAF_{rel}\,(1+s_{rel})}{(1+KA)^2}\right\}\right\} \tag{53}$$

In[∘]:=
```
Simplify[Reduce[dfnActiveRAFDSFC > 0]]
SimplifyPars[%]
```

Out[∘]=

$$\Bigg(RAF_{rel}<0\;\&\&\;\Bigg(\Bigg(KA<-2\;\&\&\;\Bigg(\left(1+d_{rel}>0\;\&\&\;d_{rel}<0\;\&\&\;RAF_{rel}\left((1+KA)^2+8\,RAF_{rel}\,(1+s_{rel})\right)<0\right)\;||\;\Bigg(\frac{(1+KA)^2+8\,RAF_{rel}+d_{rel}^2\,(1+8\,RAF_{rel})+2\,d_{rel}\,(1+KA+8\,RAF_{rel})}{8\,(1+d_{rel})^2\,RAF_{rel}}+s_{rel}<0\;\&\&\;\left((1+KA+d_{rel}>0\;\&\&\;1+s_{rel}>0)\;||\;\frac{2\,(1+KA)}{2+KA}+d_{rel}<0\right)\Bigg)\Bigg)\Bigg)\;||$$

$$\Bigg(KA==-2\;\&\&\;\Bigg(\left(-1<d_{rel}<0\;\&\&\;8+\frac{1}{RAF_{rel}}+8\,s_{rel}<0\right)\;||\;\left(d_{rel}>1\;\&\&\;-1<s_{rel}<-\frac{1+8\,RAF_{rel}+2\,d_{rel}\,(-1+8\,RAF_{rel})+d_{rel}^2\,(1+8\,RAF_{rel})}{8\,(1+d_{rel})^2\,RAF_{rel}}\right)\Bigg)\Bigg)\;||\;\Bigg(-2<KA<-1\;\&\&$$

$$\Bigg(\left(-1<d_{rel}<0\;\&\&\;RAF_{rel}\left((1+KA)^2+8\,RAF_{rel}\,(1+s_{rel})\right)<0\right)\;||\;\Bigg(-1-KA<d_{rel}<-\frac{2\,(1+KA)}{2+KA}\;\&\&$$

$$-1 < s_{rel} < -\frac{(1 + KA)^2 + 8\,RAF_{rel} + d_{rel}^2\,(1 + 8\,RAF_{rel}) + 2\,d_{rel}\,(1 + KA + 8\,RAF_{rel})}{8\,(1 + d_{rel})^2\,RAF_{rel}}\Big)\ ||$$

$$\Big(d_{rel} \geq -\frac{2\,(1 + KA)}{2 + KA}\ \&\&\ -1 < s_{rel} < -1 - \frac{(1 + KA)^2}{8\,RAF_{rel}}\Big)\Big)\Big)\ ||\ (KA == -1\ \&\&\ -1 < d_{rel} < 0\ \&\&\ s_{rel} < -1)\ ||$$

$$\Big(-1 < KA < 0\ \&\&\ \Big(\Big(d_{rel} < -1\ \&\&\ -1 < s_{rel} < -1 - \frac{(1 + KA)^2}{8\,RAF_{rel}}\Big)\ ||\ (-1 < d_{rel} \leq -1 - KA\ \&\&\ s_{rel} < -1)\ ||$$

$$\Big(-1 - KA < d_{rel} < 0\ \&\&\ \Big(s_{rel} < -1\ ||$$

$$-1 < s_{rel} < -\frac{(1 + KA)^2 + 8\,RAF_{rel} + d_{rel}^2\,(1 + 8\,RAF_{rel}) + 2\,d_{rel}\,(1 + KA + 8\,RAF_{rel})}{8\,(1 + d_{rel})^2\,RAF_{rel}}\Big)\Big)\Big)\Big)\ ||$$

$$\Big(KA == 0\ \&\&\ d_{rel} < -1\ \&\&\ -1 < s_{rel} < -1 - \frac{1}{8\,RAF_{rel}}\Big)\ ||\ \Big(KA > 0\ \&\&$$

$$\Big(\Big(-\frac{2\,(1 + KA)}{2 + KA} < d_{rel} < -1\ \&\&\ \Big(-1 < s_{rel} < -1 - \frac{(1 + KA)^2}{8\,RAF_{rel}}\ ||\ s_{rel} < -1\Big)\Big)\ ||$$

$$\Big(-1 < s_{rel} < -\frac{(1 + KA)^2 + 8\,RAF_{rel} + d_{rel}^2\,(1 + 8\,RAF_{rel}) + 2\,d_{rel}\,(1 + KA + 8\,RAF_{rel})}{8\,(1 + d_{rel})^2\,RAF_{rel}}\ \&\&$$

$$(1 + KA + d_{rel} < 0\ ||\ d_{rel} > 0)\Big)\ ||\ (s_{rel} < -1\ \&\&\ d_{rel} > 0)\Big)\Big)\Big)\Big)\ ||$$

$$\Big(RAF_{rel} > 0\ \&\&\ \Big(\Big(1 + s_{rel} > 0\ \&\&\ \Big(\Big(KA > 0\ \&\&\ \Big(-1 < d_{rel} < 0\ ||\ \frac{2\,(1 + KA)}{2 + KA} + d_{rel} < 0\ ||\ 1 + KA + d_{rel} \leq 0\Big)\Big)\ ||$$

$$(-1 \leq KA < 0\ \&\&\ d_{rel} > 0)\ ||\ (-1 \leq KA \leq 0\ \&\&\ 1 + d_{rel} < 0)\ ||\ (1 + KA < 0\ \&\&\ 1 + KA + d_{rel} \geq 0)\Big)\Big)\ ||$$

$$\Big(1 + KA < 0\ \&\&\ \Big(2 + KA \geq 0\ ||\ \frac{2\,(1 + KA)}{2 + KA} + d_{rel} > 0\Big)\ \&\&\ 1 + d_{rel} < 0\ \&\&$$

$$RAF_{rel}\,\big((1 + KA)^2 + 8\,RAF_{rel}\,(1 + s_{rel})\big) > 0\Big)\ ||$$

$$\Big(\frac{(1 + KA)^2 + 8\,RAF_{rel} + d_{rel}^2\,(1 + 8\,RAF_{rel}) + 2\,d_{rel}\,(1 + KA + 8\,RAF_{rel})}{8\,(1 + d_{rel})^2\,RAF_{rel}} + s_{rel} > 0\ \&\&\ 1 + KA + d_{rel} < 0\ \&\&$$

$$\Big((d_{rel} > 0\ \&\&\ 1 + KA < 0)\ ||\ \Big(1 + KA > 0\ \&\&\ \frac{2\,(1 + KA)}{2 + KA} + d_{rel} > 0\ \&\&\ KA < 0\ \&\&\ 1 + s_{rel} < 0\Big)\Big)\Big)\ ||$$

$$\Big(1 + s_{rel} < 0\ \&\&\ \Big(\Big(RAF_{rel}\,\big((1 + KA)^2 + 8\,RAF_{rel}\,(1 + s_{rel})\big) > 0\ \&\&$$

$$\Big(\Big(1 + KA > 0\ \&\&\ KA < 0\ \&\&\ \Big(d_{rel} > 0\ ||\ \Big(1 + d_{rel} > 0\ \&\&\ \frac{2\,(1 + KA)}{2 + KA} + d_{rel} \leq 0\Big)\Big)\Big)\ ||$$

$$(KA > 0\ \&\&\ -1 < d_{rel} < 0)\Big)\Big)\ ||\ \Big(\frac{2\,(1 + KA)}{2 + KA} + d_{rel} < 0\ \&\&\ KA > 0\ \&\&\ 1 + KA + d_{rel} > 0\ \&\&$$

$$\frac{(1 + KA)^2 + 8\,RAF_{rel} + d_{rel}^2\,(1 + 8\,RAF_{rel}) + 2\,d_{rel}\,(1 + KA + 8\,RAF_{rel})}{8\,(1 + d_{rel})^2\,RAF_{rel}} + s_{rel} > 0\Big)\Big)\Big)\Big)\Big)$$

Out[ ]= False

Therefore the derivative of fold-change relative to unbound 14-3-3 is NEVER positive. Hence it is a monotonically reducing function of 14-3-3 unbound.

In[∘]:=
```
(*The same relationship can also be validated by evaluating the function at the
  maximal fold change derived under slow s[d] assumption from previous sub-section.*)
fnActiveRAFDSFC1 = SimplifyPars[fnActiveRAFDSFC /. zeroes[[2]]]
dfnActiveRAFDSFC1 = SimplifyPars[D[fnActiveRAFDSFC1, s_rel]];
cond1 = Reduce[dfnActiveRAFDSFC1 > 0];
SimplifyPars[Reduce[{exist, cond1}]]
```

Out[∘]=
$$\frac{4\,(1+8\,RAF_{rel}\,(1+s_{rel}))\,\left(-1-4\,RAF_{rel}\,(1+s_{rel})+\sqrt{1+6\,RAF_{rel}\,(1+s_{rel})}\right)^2}{KA\,\left(-1+2\sqrt{1+6\,RAF_{rel}\,(1+s_{rel})}\right)^3\left(1+KA-\sqrt{(1+KA)^2+8\,RAF_{rel}\,(1+s_{rel})}\right)^2}$$

Out[∘]= False

▪ 3.6. Relationship between unbound and total 14-3-3

In[∘]:=
```
eqnsconsrv[[3]]
```

Out[∘]=
$$\frac{\left(A^2\,(d+Kd)^2+Kd^2\,Kdim\,KsD\right)s}{Kd^2\,Kdim\,KsD} == STOT$$

In[∘]:=
```
repratios = {RAF → RAF_rel Kdim, d → Kd d_rel, s → KsD s_rel}
```

Out[∘]= $\{RAF \to Kdim\,RAF_{rel},\ d \to Kd\,d_{rel},\ s \to KsD\,s_{rel}\}$

In[∘]:=
```
solStot = FullSimplify[SimplifyPars[(SimplifyPars[eqnsconsrv[[3]][[1]]] /. repratios) /.
      SimplifyPars[(sol23A /. repratios)]], Kd > 0]
```

Out[∘]=
$$\frac{s_{rel}\left(Kd^2\,Kdim\,KsD+\frac{\left(Kd\,Kdim\,(1+KA+d_{rel})-Kdim\sqrt{Kd^2\,(1+KA+d_{rel})^2+8\,(Kd+Kd\,d_{rel})^2\,RAF_{rel}+8\,(Kd+Kd\,d_{rel})^2\,RAF_{rel}\,s_{rel}}\right)^2}{16\,(1+d_{rel})^2\,(1+s_{rel})^2}\right)}{Kd^2\,Kdim}$$

note that Kd drops out but mathematica cannot perform the simplification so we set Kd to 1.

In[∘]:=
```
solStot = SimplifyPars[solStot /. {KA → (E11 - 1 - d_rel), Kd → 1}]
```

Out[∘]=
$$\frac{s_{rel}\left(Kdim\,KsD+\frac{Kdim^2\left(E11-\sqrt{E11^2+8\,(1+d_{rel})^2\,RAF_{rel}\,(1+s_{rel})}\right)^2}{16\,(1+d_{rel})^2\,(1+s_{rel})^2}\right)}{Kdim}$$

In[∘]:=
```
dsolStot = SimplifyPars[FullSimplify[D[solStot, s_rel]]]
```

Out[∘]=
$$KsD+\left(Kdim\left(-E11^2\,(-1+s_{rel})\left(-E11+\sqrt{E11^2+8\,(1+d_{rel})^2\,RAF_{rel}\,(1+s_{rel})}\right)+4\,(1+d_{rel})^2\,RAF_{rel}\,(1+s_{rel})\left(E11\,(-2+s_{rel})+\sqrt{E11^2+8\,(1+d_{rel})^2\,RAF_{rel}\,(1+s_{rel})}\right)\right)\right)\Big/\left(8\,(1+d_{rel})^2\,(1+s_{rel})^3\sqrt{E11^2+8\,(1+d_{rel})^2\,RAF_{rel}\,(1+s_{rel})}\right)$$

In[∘]:=
```
FullSimplify[SimplifyPars[Solve[dsolStot == 0]]]
```

Out[∘]=
$$\Big\{\Big\{KsD\to\left(2\,E11^2\,Kdim\,(-1+s_{rel})\left(-E11+\sqrt{E11^2+8\,(1+d_{rel})^2\,RAF_{rel}\,(1+s_{rel})}\right)-8\,Kdim\,(1+d_{rel})^2\,RAF_{rel}\,(1+s_{rel})\left(E11\,(-2+s_{rel})+\sqrt{E11^2+8\,(1+d_{rel})^2\,RAF_{rel}\,(1+s_{rel})}\right)\right)\Big/\left(16\,(1+d_{rel})^2\,(1+s_{rel})^3\sqrt{E11^2+8\,(1+d_{rel})^2\,RAF_{rel}\,(1+s_{rel})}\right)\Big\}\Big\}$$

In[ ]:=
$$checkexp = \left(2\,E11^2\,Kdim\,(-1 + s_{rel})\,\left(-E11 + \sqrt{E11^2 + 8\,(1 + d_{rel})^2\,RAF_{rel}\,(1 + s_{rel})}\right) - 8\,Kdim\,(1 + d_{rel})^2\,RAF_{rel}\,(1 + s_{rel})\,\left(E11\,(-2 + s_{rel}) + \sqrt{E11^2 + 8\,(1 + d_{rel})^2\,RAF_{rel}\,(1 + s_{rel})}\right)\right);$$

```
SimplifyPars[Reduce[(checkexp > 0) && (RAF_rel >= 0) && (E11 > 0)]]
```

Out[ ]= False

This function may never be positive. hence there is NO SOLUTION to first derivative of total 14-3-3 as a function of unbound 14-3-3 to be equal to zero.

Hence relationship between total and unbound 14-3-3 is MONOTONIC.

- **3.7. Convert to Python**

Section 4. 14-3-3 proteins stabilize both Autoinhibited and Dimer state (CAS+DS)

In[ ]:=
```
(*Restart kernel to prevent previous model variables to leak into following results*)
Quit[]
```

- **4.1. Analytic solutions of the model**

As for the other models, we define the equilibrium rates constrained by using the principle of detailed balance as replacement rules ‘rep’. We then calculate conservation equations for total RAF and total Drug as a function of unbound drug, unbound RAF concentrations and model parameters.

In[54]:=
```
vars = {a, A, d, AA, Ad, AAd, AdAd, as, AAs, AAsd, AAsdd, s};
```

$$repcycles = \left\{AA \to \frac{A^2}{Kdim},\ AAd \to \frac{2\,A^2\,d}{Kdim\,Kd},\ AdAd \to \frac{A^2\,d^2}{Kdim\,Kd^2},\ Ad \to \frac{A\,d}{Kd},\ as \to \frac{a\,s}{Ksm},\ AAs \to \frac{A^2\,s}{Kdim\,KsD},\ AAsd \to \frac{2\,A^2\,s\,d}{Kdim\,KsD\,Kd},\ AAsdd \to \frac{A^2\,d^2\,s}{Kdim\,KsD\,Kd^2}\right\};$$

```
repc1 = {a → A KA};
repcycles = Join[repcycles /. repc1, repc1];
rep24 = Join[repcycles /. repc1, repc1];
Consrv[eqns_] := {Simplify[eqns[[1]] + eqns[[2]] + eqns[[5]] + eqns[[8]] +
     2 (eqns[[4]] + eqns[[6]] + eqns[[7]] + eqns[[9]] + eqns[[10]] + eqns[[11]])],
   Simplify[eqns[[3]] + eqns[[5]] + eqns[[6]] + eqns[[10]] + 2 (eqns[[7]] + eqns[[11]])],
   Total[eqns[[8 ;; 12]]]};
RafActivity[vars_] := 2 (vars[[4]] + vars[[9]]) + vars[[6]] + vars[[10]];
eqnsconsrv = Thread[Simplify[Consrv[vars] /. rep24] == {RAF, DTOT, STOT}]
```

Out[61]=
$$\left\{\frac{A\,\left(2\,A\,(d + Kd)^2\,Ksm\,(KsD + s) + Kd\,Kdim\,KsD\,(d\,Ksm + Kd\,(Ksm + KA\,Ksm + KA\,s))\right)}{Kd^2\,Kdim\,KsD\,Ksm} == RAF,\ d + \frac{A\,d}{Kd} + \frac{2\,A^2\,d\,(d + Kd)\,(KsD + s)}{Kd^2\,Kdim\,KsD} == DTOT,\ \frac{\left(A\,KA\,Kd^2\,Kdim\,KsD + A^2\,(d + Kd)^2\,Ksm + Kd^2\,Kdim\,KsD\,Ksm\right)\,s}{Kd^2\,Kdim\,KsD\,Ksm} == STOT\right\}$$

In[62]:=
```
SimplifyPars[x_] := Simplify[x,
   {Kd > 0, RAF > 0, DTOT > 0, d_rel > 0, RAF_rel > 0, Kdim > 0, KA > 0, d > 0, s_rel > 0, s > 0, STOT > 0, RAF > 0}];
numsol24As = Quiet[Solve[eqnsconsrv[[{1, 3}]] /. baseparams, {A, s}][[4]]];
(*created for numerical example plot.*)
sol24A = FullSimplify[SimplifyPars[Solve[eqnsconsrv[[1]], A]]];
(*the first solution is negtive definite and second is positive.*)
sol24A = sol24A[[2]]
```

Out[65]=
$$\left\{A \to -\frac{1}{4\,(d+Kd)^2\,Ksm\,(KsD+s)}\,Kd\,Kdim\,KsD\left((d+Kd+KA\,Kd)\,Ksm+KA\,Kd\,s-Ksm\sqrt{\left(\frac{1}{Kdim\,KsD\,Ksm^2}\left(KsD\,Ksm^2\left((d+Kd+KA\,Kd)^2\,Kdim+8\,(d+Kd)^2\,RAF\right)+2\,Ksm\left(KA\,Kd\,(d+Kd+KA\,Kd)\,Kdim\,KsD+4\,(d+Kd)^2\,Ksm\,RAF\right)s+KA^2\,Kd^2\,Kdim\,KsD\,s^2\right)\right)}\right)\right\}$$

In[66]:=
```
sol4s = SimplifyPars[Solve[eqnsconsrv[[3]], s]][[1]]
```

Out[66]=
$$\left\{s \to \frac{Kd^2\,Kdim\,KsD\,Ksm\,STOT}{A\,KA\,Kd^2\,Kdim\,KsD + A^2\,(d+Kd)^2\,Ksm + Kd^2\,Kdim\,KsD\,Ksm}\right\}$$

In[67]:=
```
Simplify[RafActivity[vars] / RAF /. rep24]
```

Out[67]=
$$\frac{2\,A^2\,(d+Kd)\,(KsD+s)}{Kd\,Kdim\,KsD\,RAF}$$

In[68]:=
```
fnActiveRAF14331 = SimplifyPars[RafActivity[vars] / RAF /. rep24 /. sol24A];
repratios = {Kdim → (RAF / RAF_rel), d → (d_rel Kd)};
(*define concentrations relative to equilibrium rate constants*)
fnActiveRAF1433 = FullSimplify[SimplifyPars[fnActiveRAF14331 /. repratios]]
fnActiveRAF1433red = FullSimplify[SimplifyPars[fnActiveRAF1433 /.
    {RAF_rel → E8 / 8 / (1 + d_rel)^2 / (1 + s / KsD), KA → (-Ksm + E9 Ksm - Ksm d_rel)/(Ksm + s)}], {E8 > 0, E9 > 0}]
```

Out[70]=
$$\frac{KsD\left(Ksm+KA\,(Ksm+s)+Ksm\,d_{rel}-Ksm\sqrt{\frac{(Ksm+KA\,Ksm+KA\,s+Ksm\,d_{rel})^2}{Ksm^2}+\frac{8\,(KsD+s)\,(1+d_{rel})^2\,RAF_{rel}}{KsD}}\right)^2}{8\,Ksm^2\,(KsD+s)\,(1+d_{rel})^3\,RAF_{rel}}$$

Out[71]=
$$\frac{\left(E9-\sqrt{E8+E9^2}\right)^2}{E8\,(1+d_{rel})}$$

In[72]:=
```
Solve[Ksm + KA Ksm + KA s + Ksm d_rel == Ksm E9, E9]
```

Out[72]=
$$\left\{\left\{E9 \to \frac{Ksm+KA\,Ksm+KA\,s+Ksm\,d_{rel}}{Ksm}\right\}\right\}$$

In[73]:=
```
repE8 = Solve[RAF_rel == E8 / 8 / (1 + d_rel)^2 / (1 + s / KsD), E8][[1]]
```

Out[73]=
$$\left\{E8 \to \frac{8\,(KsD+s)\,(1+d_{rel})^2\,RAF_{rel}}{KsD}\right\}$$

▫ *Total Dimers*

In[74]:=
```
RafDimers[eqns_] := (eqns[[4]] + eqns[[6]] + eqns[[7]] + eqns[[9]] + eqns[[10]] + eqns[[11]]);
fnDimers = FullSimplify[SimplifyPars[(RafDimers[vars] / RAF /. rep24 /. sol24A) /. repratios]]
fnDimers / fnActiveRAF1433
```

Out[75]=
$$\frac{KsD\left(Ksm + KA\ (Ksm + s) + Ksm\, d_{rel} - Ksm\sqrt{\frac{(Ksm+KA\,Ksm+KA\,s+Ksm\,d_{rel})^2}{Ksm^2} + \frac{8\ (KsD+s)\ (1+d_{rel})^2\, RAF_{rel}}{KsD}}\right)^2}{16\, Ksm^2\ (KsD + s)\ (1 + d_{rel})^2\, RAF_{rel}}$$

Out[76]=
$$\frac{1}{2}\ (1 + d_{rel})$$

▪ 4.2. Baseline Signaling

As a function of unbound 14-3-3:

In[∘]:=
```
SimplifyPars1[x_] := Simplify[x, {Kd > 0, RAF > 0, DTOT > 0, drel > 0,
    RAFrel > 0, Kdim > 0, KA > 0, d > 0, srel > 0, s > 0, STOT > 0, RAF > 0, Ksm > 0, KsD > 0}];
fnActiveRAF1433nodrug = FullSimplify[SimplifyPars1[
    (fnActiveRAF1433 /. drel → 0) /. RAFrel → E10 (1 + KA + KA s / Ksm)^2 / (1 + s / KsD) / 8]]
repE10 = Solve[RAFrel == E10 (1 + KA + KA s / Ksm)^2 / (1 + s / KsD) / 8, E10][[1]]
d4ND = Simplify[SimplifyPars[D[fnActiveRAF1433nodrug, E10]], E10 > 0]
```

Out[∘]=
$$\frac{\left(-1 + \sqrt{1 + E10}\right)^2}{E10}$$

Out[∘]=
$$\left\{E10 \to \frac{8\, Ksm^2\ (KsD + s)\ RAF_{rel}}{KsD\ (Ksm + KA\, Ksm + KA\, s)^2}\right\}$$

Out[∘]=
$$\frac{\left(-1 + \sqrt{1 + E10}\right)^2}{E10^2\ \sqrt{1 + E10}}$$

As a function of E10, the derivative of baseline signaling is positive definite, therefore E10 shares a monotonic increasing relationship with baseline signaling.

While s_{rel} is itself a complex function of other parameters, we may ask under what conditions the relationship between baseline signaling and unbound 14-3-3 is monotonic?

In[∘]:=
```
dE10s = Simplify[D[(KsD + s) / (Ksm + KA Ksm + KA s)^2, s]]
dE10szero = Solve[dE10s == 0, s][[1]]
```

$$\frac{Ksm + KA\ (-2\, KsD + Ksm - s)}{(Ksm + KA\, Ksm + KA\, s)^3} \qquad (55)$$

Out[∘]=
$$s \to \frac{-2\, KA\, KsD + Ksm + KA\, Ksm}{KA}$$

In[∘]:=
```
d2E10s = SimplifyPars1[D[dE10s, s]];
SimplifyPars1[d2E10s /. dE10szero]
```

Out[∘]=
$$\frac{KA}{8\ (KA\ (KsD - Ksm) - Ksm)^3}$$

In[∘]:=
```
dE10c1 = SimplifyPars1[Reduce[dE10srel > 0]]
```

$$KA < \frac{Ksm}{2\, KsD - Ksm + s}\ ||\ 2\, KsD + s \le Ksm \qquad (56)$$

P0. Condition 64 is satisfied whenever, Ksm>2 KsD - therefore, a monotonic **increasing** relationship exists between the 14-3-3 unbound and

E10 and therefore between 14-3-3 unbound and baseline signaling. That is, when the equilibrium constants favors dimer stabilization, the induction of 14-3-3 induces baseline signaling.

```
In[•]:= dE10c2 = SimplifyPars1[Reduce[dE10srel < 0]]
```

$$\text{Ksm} < 2\,\text{KsD} + \text{s}\ \&\&\ (1 + \text{KA})\,\text{Ksm} < \text{KA}\,(2\,\text{KsD} + \text{s}) \qquad (57)$$

P1. Condition 65 is satisfied when Ksm<2KsD*KA/(1+KA). Since KA is always smaller than 1+KA, the condition Ksm<2KsD is automatically satisfied. In this case, a monotonic reducing relationship exists between 14-3-3 unbound and E10 and therefore between 14-3-3 unbound and baseline signaling.

```
In[•]:= BSdimerlimit = Limit[fnActiveRAF1433nodrug /. repE10, Ksm → ∞]
```

Out[•]=
$$\frac{(1+\text{KA})^2\,\text{KsD}\left(-1+\sqrt{1+\frac{8\,(\text{KsD}+\text{s})\,\text{RAF}_{\text{rel}}}{(1+\text{KA})^2\,\text{KsD}}}\right)^2}{8\,(\text{KsD}+\text{s})\,\text{RAF}_{\text{rel}}}$$

```
In[•]:= SimplifyPars[D[BSdimerlimit, s]]
```

Out[•]=
$$\frac{(1+\text{KA})^2\,\text{KsD}\left(-1+\sqrt{1+\frac{8\,(\text{KsD}+\text{s})\,\text{RAF}_{\text{rel}}}{(1+\text{KA})^2\,\text{KsD}}}\right)^2}{8\,(\text{KsD}+\text{s})^2\,\text{RAF}_{\text{rel}}\sqrt{1+\frac{8\,(\text{KsD}+\text{s})\,\text{RAF}_{\text{rel}}}{(1+\text{KA})^2\,\text{KsD}}}}$$

In the absence of CAS mechanism, the baseline signaling is always enhanced by 14-3-3 unbound increase as the derivative is a Positive definite function of unbound 14-3-3.

```
In[•]:= BSCASlimit = Limit[fnActiveRAF1433nodrug /. repE10, KsD -> ∞]
```

Out[•]=
$$\frac{(\text{Ksm}+\text{KA}\,\text{Ksm}+\text{KA}\,\text{s})^2\left(-1+\sqrt{1+\frac{8\,\text{Ksm}^2\,\text{RAF}_{\text{rel}}}{(\text{Ksm}+\text{KA}\,\text{Ksm}+\text{KA}\,\text{s})^2}}\right)^2}{8\,\text{Ksm}^2\,\text{RAF}_{\text{rel}}}$$

```
In[•]:= SimplifyPars[D[BSCASlimit, s]]
```

Out[•]=
$$-\frac{\text{KA}\,(\text{Ksm}+\text{KA}\,\text{Ksm}+\text{KA}\,\text{s})\left(-1+\sqrt{1+\frac{8\,\text{Ksm}^2\,\text{RAF}_{\text{rel}}}{(\text{Ksm}+\text{KA}\,\text{Ksm}+\text{KA}\,\text{s})^2}}\right)^2}{4\,\text{Ksm}^2\,\text{RAF}_{\text{rel}}\sqrt{1+\frac{8\,\text{Ksm}^2\,\text{RAF}_{\text{rel}}}{(\text{Ksm}+\text{KA}\,\text{Ksm}+\text{KA}\,\text{s})^2}}}$$

In the absence of DS mechanism, the baseline signaling is always reduced by unbound 14-3-3 as the derivative is a negative definite function of unbound 14-3-3.

- ***Unbound vs total 14-3-3***

- **4.3. Conditions on parameter regions for activation in response to the drug**

- ***Unbound 14-3-3 is a slowly varying function of drug compared to active kinase***

```
dAKbyd = D[SimplifyPars[RafActivity[vars] / RAF /. rep24] /. {s → s[d], A → A[d]}, d]
dAKbyd[[-1]]
dAKbyd[[;; -2]]
```

$$\frac{2\,\text{A[d]}^2\,(\text{KsD}+\text{s[d]})}{\text{Kd Kdim KsD RAF}}+\frac{4\,(\text{d}+\text{Kd})\,\text{A[d]}\,(\text{KsD}+\text{s[d]})\,\text{A}'\text{[d]}}{\text{Kd Kdim KsD RAF}}+\frac{2\,(\text{d}+\text{Kd})\,\text{A[d]}^2\,\text{s}'\text{[d]}}{\text{Kd Kdim KsD RAF}}$$

$$\frac{2\,(\text{d}+\text{Kd})\,\text{A[d]}^2\,\text{s}'\text{[d]}}{\text{Kd Kdim KsD RAF}}$$

$$\frac{2\,A[d]^2\,(KsD + s[d])}{Kd\,Kdim\,KsD\,RAF} + \frac{4\,(d + Kd)\,A[d]\,(KsD + s[d])\,A'[d]}{Kd\,Kdim\,KsD\,RAF}$$

In[ ]:=
```
dsbyd = s′[d] → FullSimplify[D[s /. sol4s /. A → A[d], d]]
```

Out[ ]=

$$s'[d] \to \frac{Kd^2\,Kdim\,KsD\,Ksm\,STOT\,\left(-2\,(d + Kd)\,Ksm\,A[d]^2 - \left(KA\,Kd^2\,Kdim\,KsD + 2\,(d + Kd)^2\,Ksm\,A[d]\right)\,A'[d]\right)}{\left(Kd^2\,Kdim\,KsD\,Ksm + KA\,Kd^2\,Kdim\,KsD\,A[d] + (d + Kd)^2\,Ksm\,A[d]^2\right)^2}$$

In[ ]:=
```
FullSimplify[Simplify[dAKbyd[[-1]] / dAKbyd[[ ;; -2]]] /. dsbyd]
```

Out[ ]=

$$\frac{Kd^2\,(d + Kd)\,Kdim\,KsD\,Ksm\,STOT\,A[d]\,\left(-2\,(d + Kd)\,Ksm\,A[d]^2 - \left(KA\,Kd^2\,Kdim\,KsD + 2\,(d + Kd)^2\,Ksm\,A[d]\right)\,A'[d]\right)}{\left(Kd^2\,Kdim\,KsD\,Ksm + KA\,Kd^2\,Kdim\,KsD\,A[d] + (d + Kd)^2\,Ksm\,A[d]^2\right)^2\,(KsD + s[d])\,(A[d] + 2\,(d + Kd)\,A'[d])}$$

while deriving the full set of conditions isn't possible without working with full solution for [s] and [A] which are unwieldy expressions. It is possible to derive a solution under the limit of slowly varying $s[d_{\mathrm{rel}}]$ such that Subscript[ds, rel]/dd_{rel}=0. So we first establish that the unbound 14-3-3 only slowly varies as a function

```
parset01 = {KA → 10., Kdim → 1. × 10^-7 , KsD → 10^-7, Ksm → 10^-6, STOT → 2 × 10^-6};
sol4SA = Solve[eqnsconsrv[[{1, 3}]], {A, s}];
sol4SA /. parset01 /. {Kd -> 0.1, d -> 0.1, RAF -> 4 × 10^-8}
```

Out[ ]=

```
{{A → -7.81213×10^-8 - 2.16194×10^-7 i, s → -8.30953×10^-8 - 5.8811×10^-8 i},
 {A → -7.81213×10^-8 + 2.16194×10^-7 i, s → -8.30953×10^-8 + 5.8811×10^-8 i},
 {A → -1.99435×10^-8, s → 2.08407×10^-6}, {A → 1.1861×10^-9, s → 1.97546×10^-6}}
```

```
Sty[x_] := Style[x, "Arial", 26];
dAK = D[fnActiveRAF14331 /. sol4SA[[4]], d];
repSA = {A'[d] → D[A /. sol4SA[[4]], d]};
sd = D[s /. sol4SA[[4]], d];
lpdats =
  Table[{d1, Chop[((dAKbyd[[-1]] /. A[d] → A) /. {s′[d] -> N[sd /. baseparams /. d → d1]} /. repSA) /.
       sol4SA[[4]] /. baseparams /. d → d1]}, {d1, Table[10^i, {i, -9, -3, 0.5}]}];
lpdatAK = Table[{d1, Chop[N[dAK /. baseparams /. d → d1]]}, {d1, Table[10^i, {i, -9, -3, 0.5}]}];
lpdatAK2sratio =
  Table[{lpdats[[i, 1]], lpdatAK[[i, 2]] / lpdats[[i, 2]]}, {i, 1, Length[lpdats]}];
```

In[ ]:=
```
ListLogLogPlot[{lpdats, lpdatAK}, Joined -> True,
 PlotLegends -> {"ds part of dAK", "dAK"}, PlotRange → Automatic]
```

Out[ ]=

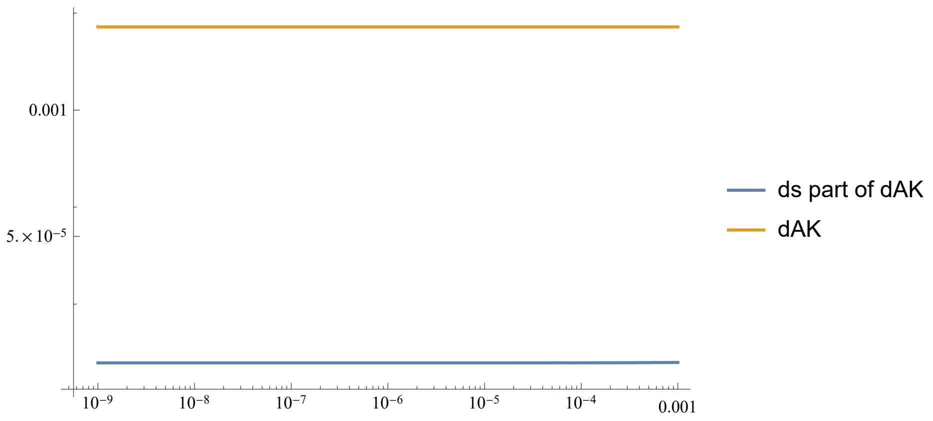

In[ ]:= `ListLogLogPlot[lpdatAK2sratio, Joined → True, PlotLegends → "dAK/ds", PlotRange → {0, 1}]`

Out[ ]=

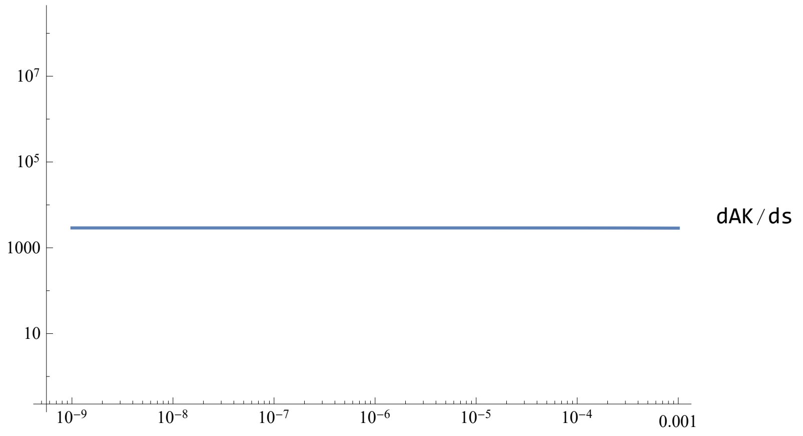

Hence, for almost all of the drug values the derivative of active kinase relative drug is in far excess of the derivate of 14-3-3 relative to drug. This also makes sense since the drug does not directly bind with 14-3-3 and 14-3-3 is affected by the indirect change in the dimer concentrations. Under the consideration that 14-3-3 changes slowly, the following expressions can be derived.

PA conditions

In[ ]:=
```
dfn4rel =
  Simplify[D[fnActiveRAF1433red /. {E8 → E8[drel], E9 → E9[drel]}, drel], {E9[drel] > 0, E8[drel] > 0}]
dfn4rel[[-1]]
rep4rel = {D[E9[drel], drel] → 1, (-E9[drel] + Sqrt[E8[drel] + E9[drel]^2]) → C2,
   D[E8[drel], drel] → 2 E8[drel] / (1 + drel), E9[drel] -> (Ksm + KA Ksm + KA s + Ksm drel)/Ksm};
```

$$\frac{1}{E8[d_{rel}]^2 \sqrt{E8[d_{rel}] + E9[d_{rel}]^2}\ (1+d_{rel})^2} \left(-E9[d_{rel}] + \sqrt{E8[d_{rel}] + E9[d_{rel}]^2}\right) \left(-E8[d_{rel}]^2 + E9[d_{rel}] \left(-E9[d_{rel}] + \sqrt{E8[d_{rel}] + E9[d_{rel}]^2}\right) (1+d_{rel})\, E8'[d_{rel}] + E8[d_{rel}] \left(-E9[d_{rel}] + \sqrt{E8[d_{rel}] + E9[d_{rel}]^2}\right) (E9[d_{rel}] - 2\,(1+d_{rel})\, E9'[d_{rel}])\right) \quad (58)$$

$$-E8[d_{rel}]^2 + E9[d_{rel}] \left(-E9[d_{rel}] + \sqrt{E8[d_{rel}] + E9[d_{rel}]^2}\right) (1+d_{rel})\, E8'[d_{rel}] + E8[d_{rel}] \left(-E9[d_{rel}] + \sqrt{E8[d_{rel}] + E9[d_{rel}]^2}\right) (E9[d_{rel}] - 2\,(1+d_{rel})\, E9'[d_{rel}]) \quad (59)$$

The first part of the derivative function is positive definite, - the denominator is positive function and the numerator is also positive definite as the E9 < Sqrt[$E9^2$+E8]. So we evaluate the second part in equation 67 in more detail.

In[ ]:=
```
dfn4rel1 = Simplify[dfn4rel[[-1]] /. rep4rel]
dfn4rel2 = Simplify[dfn4rel1[[-1]] /.
    C2 → (((-E9[drel] + Sqrt[E8[drel] + E9[drel]^2]) /. E9[drel] -> (Ksm + KA Ksm + KA s + Ksm drel)/Ksm) /.
      (repE8 /. E8 → E8[drel]))];
zeroes = SimplifyPars[Solve[dfn4rel2 == 0, drel]]
```

$$\frac{E8[d_{rel}]\ (-Ksm\ E8[d_{rel}] + C2\ (Ksm + 3\ KA\ Ksm + 3\ KA\ s + Ksm\ d_{rel}))}{Ksm} \quad (60)$$

$\{\{d_{rel} \to -1\},\ \{d_{rel} \to -((KsD\ Ksm\ (Ksm + KA\ Ksm + KA\ s) + 8\ Ksm^2\ (KsD + s)\ RAF_{rel} +$

$$2\,KA\sqrt{KsD\,Ksm^2\,(Ksm+s)^2\,(KsD+6\,(KsD+s)\,RAF_{rel})}\Big)\Big/\left(Ksm^2\,(KsD+8\,(KsD+s)\,RAF_{rel})\right)\Big)\Big\},$$

$$\Big\{d_{rel}\to\Big(-KsD\,Ksm\,(Ksm+KA\,Ksm+KA\,s)-8\,Ksm^2\,(KsD+s)\,RAF_{rel}+$$

$$2\,KA\sqrt{KsD\,Ksm^2\,(Ksm+s)^2\,(KsD+6\,(KsD+s)\,RAF_{rel})}\Big)\Big/\left(Ksm^2\,(KsD+8\,(KsD+s)\,RAF_{rel})\right)\Big\}\Big\}$$

Only third solution may be positive value of d_{rel}. The first and second are negative definite due to an overall negative sign followed by a positive combination of positive definite parameters and concentrations.

In[∘]:= **exist = SimplifyPars1[Reduce[((d_rel /. zeroes[[3]]) > 0)]]**

$$KA>\frac{Ksm+2\,Ksm\sqrt{1+\frac{6\,(KsD+s)\,RAF_{rel}}{KsD}}}{3\,(Ksm+s)} \qquad (62)$$

In[∘]:= **d2fn4 = SimplifyPars[D[(dfn4rel1 /. C2 → ((-E9[d_rel] + √(E8[d_rel] + E9[d_rel]^2)) /. E9[d_rel] -> (Ksm + KA Ksm + KA s + Ksm d_rel)/Ksm) /. (repE8 /. E8 → E8[d_rel])), d_rel]];**

In[∘]:= **d2fn4z = SimplifyPars[d2fn4 /. zeroes[[3]]]**

Out[∘]=

$$\Bigg(16\,KA^3\,(KsD+s)\,(Ksm+s)\,RAF_{rel}\left(KsD\,Ksm\,(Ksm+s)-2\sqrt{KsD\,Ksm^2\,(Ksm+s)^2\,(KsD+6\,(KsD+s)\,RAF_{rel})}\right)$$
$$\Bigg(48\,Ksm\,(KsD+s)^2\,(Ksm+s)\left(-11\,s+Ksm\left(-11+12\sqrt{\frac{(Ksm+s)^2}{Ksm^2}}\right)\right)RAF_{rel}^2-$$
$$2\,(KsD+s)\,RAF_{rel}\Bigg(-KsD\,Ksm\,(Ksm+s)\left(-51\,s+Ksm\left(-51+64\sqrt{\frac{(Ksm+s)^2}{Ksm^2}}\right)\right)-$$
$$8\left(-3\,s+Ksm\left(-3+2\sqrt{\frac{(Ksm+s)^2}{Ksm^2}}\right)\right)\sqrt{KsD\,Ksm^2\,(Ksm+s)^2\,(KsD+6\,(KsD+s)\,RAF_{rel})}\Bigg)+$$
$$KsD\Bigg(KsD\,Ksm\,(Ksm+s)\left(-4\,s+Ksm\left(-4+7\sqrt{\frac{(Ksm+s)^2}{Ksm^2}}\right)\right)+$$
$$\left(-7\,s+Ksm\left(-7+4\sqrt{\frac{(Ksm+s)^2}{Ksm^2}}\right)\right)\sqrt{KsD\,Ksm^2\,(Ksm+s)^2\,(KsD+6\,(KsD+s)\,RAF_{rel})}\Bigg)\Bigg)\Bigg)\Bigg/$$
$$\left(KsD\,Ksm^6\sqrt{\frac{(Ksm+s)^2}{Ksm^2}}\,(KsD+8\,(KsD+s)\,RAF_{rel})^3\right)$$

In[∘]:= **Reduce[(d2fn4z < 0)];**
SimplifyPars1[%]

Out[∘]= True

Note that no additional constraints are added on the model parameter space. Hence, existence equations above represent all of the constraints which allow for this model to produce a paradoxical activation.

- ***Monotonic Relationship between total and unbound drug.***

In[ ]:=
```
eqnsconsrv[[2]]
```

Out[ ]=
$$d + \frac{A\,d}{Kd} + \frac{2\,A^2\,d\,(d + Kd)\,(KsD + s)}{Kd^2\,Kdim\,KsD} == DTOT$$

Numerically :

In[ ]:=
```
sol4SA2[dval_, params_] :=
  NSolve[Flatten[{eqnsconsrv[[{1, 3}]] /. d → dval /. params, A ≥ 0., s > 0.}], {A, s}];
sol4SA2[10^-7, baseparams]
```

Out[ ]=
```
{{A → 0.000371541, s → 0.964134}}
```

Only the fourth solution is positive for both A and s,

```
fnDTOT[dval_, params_] := Module[{},
  numsol4SA = sol4SA2[dval, params];
  If[Length[numsol4SA] == 1,
   (eqnsconsrv[[2]][[1]] /. rep24) /. params /. d → dval /. numsol4SA[[1]], False]]
```

In[ ]:=
```
Sty[x_] := Style[x, 17, FontFamily → "Arial"];
parset01 = {KA → 10., Kdim → 1. × 10^-7 , KsD → 10^-7, Ksm → 10^-7, STOT → 10^-6};
KTarr = {1. × 10^-6, 1. × 10^-7, 1. × 10^-8};
kdarr = { 1. × 10^-7 , 1. × 10^-9};
pltfn = Flatten[Table[{d1, fnDTOT[d1, Join[parset01, {Kd -> kd, d → d1, RAF → rt}]]},
    {kd, kdarr}, {rt, KTarr}, {d1, Table[10^i, {i, -9, -5, 0.1}]}], 1];
ps = {Blue, Green, Directive[Red, Thick], Directive[Blue, Dashed],
   Directive[Green, Dashed], Directive[Red, Dashed, Thick]};
ListLogLogPlot[pltfn, Joined → True, Frame → True, FrameTicksStyle → 20, PlotStyle → ps,
 PlotLegends → Placed[LineLegend[ {Blue, Green, Directive[Red, Thick]}, Sty /@ KTarr,
    LegendLabel → Sty["RAF (M)"], LegendFunction → Panel, LabelStyle → 12], {Right, Bottom}],
 FrameLabel → (Sty /@ {"Unbound Drug [d] (M)", "Total Drug [Drug] (M)"}),
 ImageSize → {500}, FrameStyle → Thickness[0.004],
 PlotLabel → Sty["Kd: −100nM  - −1nM KA:10 KSdim=KSmon:100nM [14-3-3]:1μM"]
 (*N[TableForm[parset01,TableDirections→Row]]*)]
```

Out[ ]=

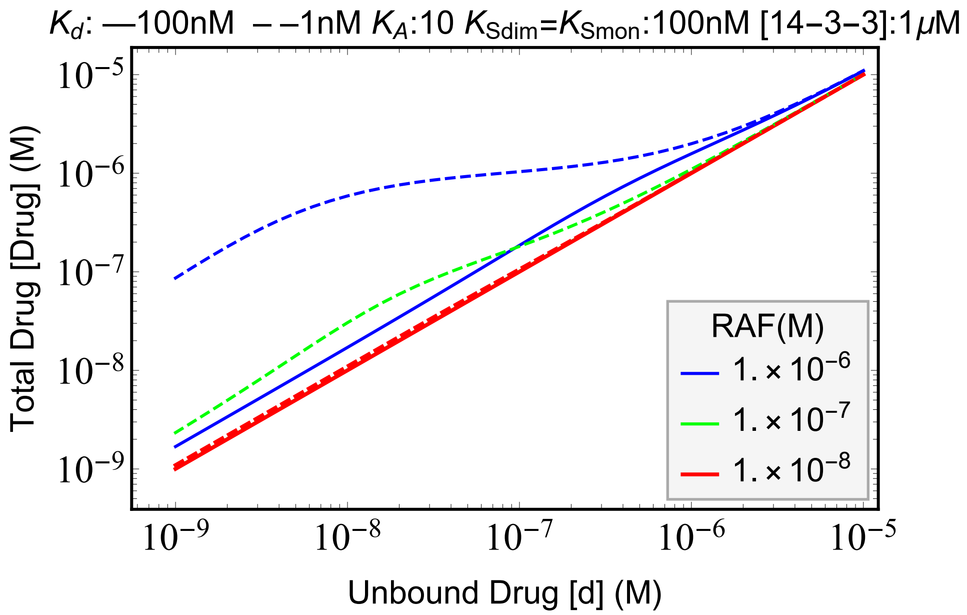


```
Sty[x_] := Style[x, 15, FontFamily → "Arial"];
parset01 = {KA → 10., Kdim → 1. × 10^-7 , KsD → 10^-5, Ksm → 10^-7, STOT → 10^-6};
KTarr = {1. × 10^-6, 1. × 10^-7, 1. × 10^-8};
kdarr = { 1. × 10^-7 , 1. × 10^-9};
pltfn = Flatten[Table[{d1, fnDTOT[d1, Join[parset01, {Kd -> kd, d → d1, RAF → rt}]]},
    {kd, kdarr}, {rt, KTarr}, {d1, Table[10^i, {i, -9, -5, 0.1}]}], 1];
ps = {Blue, Green, Directive[Red, Thick], Directive[Blue, Dashed],
   Directive[Green, Dashed], Directive[Red, Dashed, Thick]};
ListLogLogPlot[pltfn, Joined → True, Frame → True, FrameTicksStyle → 20, PlotStyle → ps,
 PlotLegends → Placed[LineLegend[ {Blue, Green, Directive[Red, Thick]}, Sty /@ KTarr,
    LegendLabel → Sty["RAF (M)"], LegendFunction → Panel, LabelStyle → 12], {Right, Bottom}],
 FrameLabel → (Sty /@ {"Unbound Drug [d] (M)", "Total Drug [Drug] (M)"}),
 ImageSize → {500}, FrameStyle → Thickness[0.004],
 PlotLabel → Sty["Kd: -100nM  - -1nM KA:10 KSdim:10^4nM KSmon:100nM [14-3-3]:1μM"]
 (*N[TableForm[parset01,TableDirections→Row]]*)]
```

Out[•]=

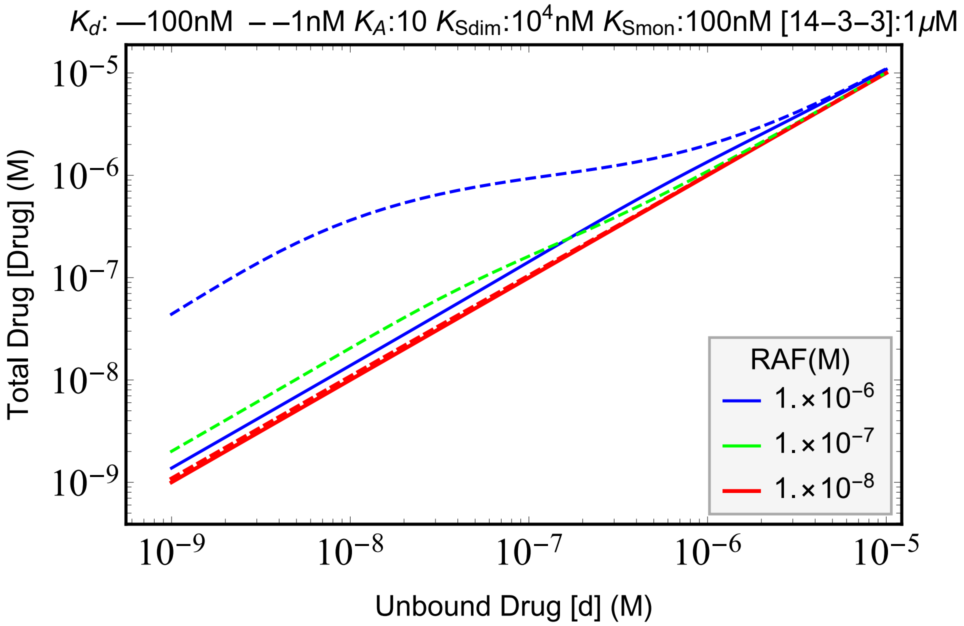


```
Sty[x_] := Style[x, 15, FontFamily → "Arial"];
parset01 = {KA → 10., Kdim → 1. × 10^-7 , KsD → 10^-7, Ksm → 10^-5, STOT → 10^-6};
KTarr = {1. × 10^-6, 1. × 10^-7, 1. × 10^-8};
kdarr = { 1. × 10^-7 , 1. × 10^-9};
pltfn = Flatten[Table[{d1, fnDTOT[d1, Join[parset01, {Kd -> kd, d → d1, RAF → rt}]]},
    {kd, kdarr}, {rt, KTarr}, {d1, Table[10^i, {i, -9, -5, 0.1}]}], 1];
ps = {Blue, Green, Directive[Red, Thick], Directive[Blue, Dashed],
   Directive[Green, Dashed], Directive[Red, Dashed, Thick]};
ListLogLogPlot[pltfn, Joined → True, Frame → True, FrameTicksStyle → 20, PlotStyle → ps,
 PlotLegends → Placed[LineLegend[ {Blue, Green, Directive[Red, Thick]}, Sty /@ KTarr,
    LegendLabel → Sty["RAF(M)"], LegendFunction → Panel, LabelStyle → 12], {Right, Bottom}],
 FrameLabel → (Sty /@ {"Unbound Drug [d] (M)", "Total Drug [Drug] (M)"}),
 ImageSize → {500}, FrameStyle → Thickness[0.004],
 PlotLabel → Sty["K_d: −100nM  - -1nM K_A:10 K_Sdim:100nM K_Smon:10^4nM [14-3-3]:1μM"]
 (*N[TableForm[parset01,TableDirections→Row]]*)]
```

Out[•]=

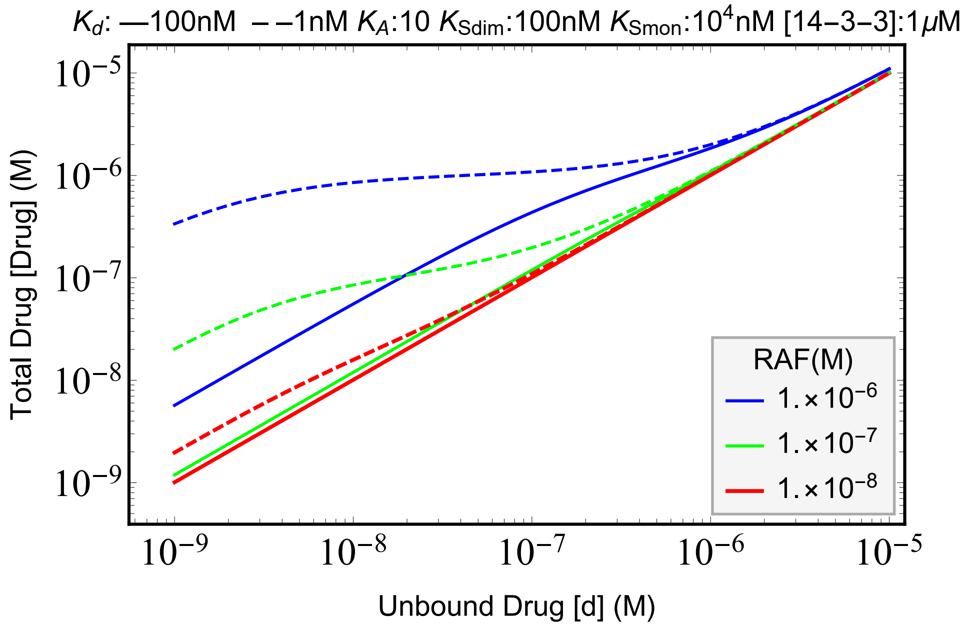


```
Sty[x_] := Style[x, 15, FontFamily → "Arial"];
parset01 = {KA → 10., Kdim → 1. × 10^-7 , KsD → 10^-7, Ksm → 10^-7, RAF → 4 × 10^-8};
KTarr = {1. × 10^-5, 1. × 10^-7, 1. × 10^-9};
kdarr = { 1. × 10^-7 , 1. × 10^-9};
pltfn = Flatten[Table[{d1, fnDTOT[d1, Join[parset01, {Kd -> kd, d → d1, STOT → rt}]]},
    {kd, kdarr}, {rt, KTarr}, {d1, Table[10^i, {i, -9, -5, 0.1}]}], 1];
ps = {Blue, Green, Directive[Red, Thick], Directive[Blue, Dashed],
   Directive[Green, Dashed], Directive[Red, Dashed, Thick]};
ListLogLogPlot[pltfn, Joined → True, Frame → True, FrameTicksStyle → 20, PlotStyle → ps,
 PlotLegends → Placed[LineLegend[ {Blue, Green, Directive[Red, Thick]}, Sty /@ KTarr,
    LegendLabel → Sty["[14-3-3] (M)"], LegendFunction → Panel, LabelStyle → 12], {Right, Bottom}],
 FrameLabel → (Sty /@ {"Unbound Drug [d] (M)", "Total Drug [Drug] (M)"}),
 ImageSize → {500}, FrameStyle → Thickness[0.004],
 PlotLabel → Sty["Kd: –100nM  - -1nM KA:10 KSdim=KSmon:100nM [RAF]:0.04μM"]
 (*N[TableForm[parset01,TableDirections→Row]]*)]
```

Out[ ]=

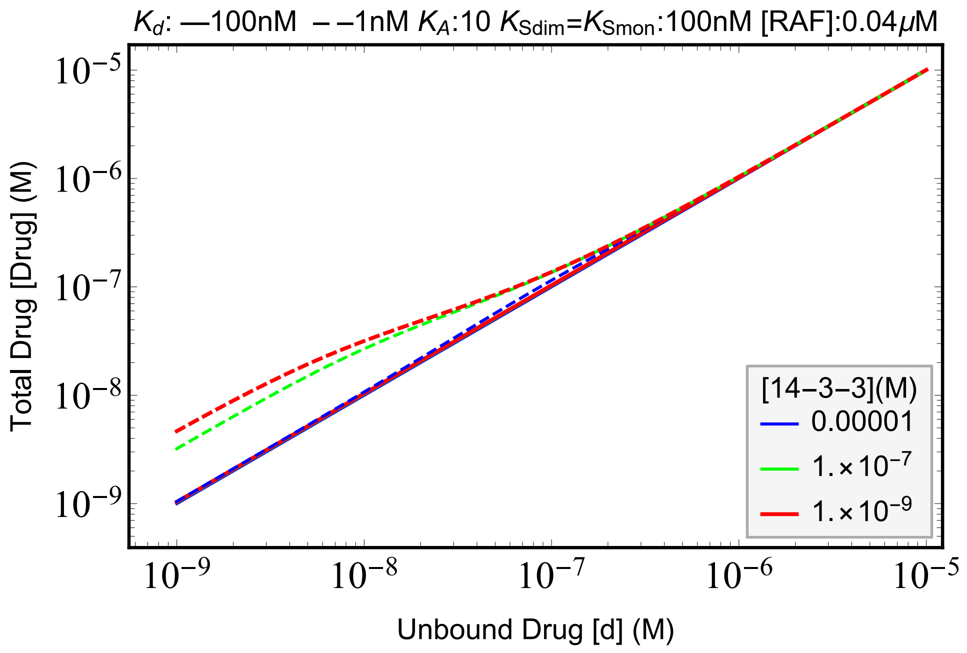

The relationship between unbound and total drug is monotonic for the wide range of parameter choices explored. Hence the analytic results obtained in previous sections apply to a wide range of parameter values and generalize to the drug administered and not just to the unbound drug available inside each cell.

- **4.4. Analytic Expressions for maximum Fold Change (FC)**

```
fnActiveRAF1433fc = SimplifyPars[fnActiveRAF1433 / SimplifyPars[fnActiveRAF1433 /. d_rel → 0]];
rep3 = {RAF_rel → E8 / 8 / (1 + d_rel)^2 / (1 + s / KsD), KA → (-Ksm + E9 Ksm - Ksm d_rel)/(Ksm + s)};
rep4 = {RAF_rel → E10 (1 + KA + KA s / Ksm)^2 / (1 + s / KsD) / 8};
SimplifyPars1[(fnActiveRAF1433 /. rep3) / Simplify[(fnActiveRAF1433 /. rep4) /. d_rel → 0, E8 > 0]]
```

Out[ ]=

$$\frac{E10\left(E9-\sqrt{E8+E9^2}\right)^2}{\left(-1+\sqrt{1+E10}\right)^2 E8\,(1+d_{rel})}$$

In[ ]:= `SimplifyPars[Solve[(RAF_rel /. rep4) == RAF_rel, E10]][[1]]`

Out[ ]=

$$\left\{E10\to\frac{8\,Ksm^2\,(KsD+s)\,RAF_{rel}}{KsD\,(Ksm+KA\,Ksm+KA\,s)^2}\right\}$$

- **4.5. Convert to Python**

Descriptive, example Plots

Note: Initialize baseparams variable (first line) and all first sub sections (*.1) to get the following plots.

In[77]:= `Sty[x_] := Style[x, 22, Bold, FontFamily → "Times"];`

In[88]:=
```
(*the fourth solution is positive definite -
 recheck each time run on a new mathematica version.*)
plfn1 = {(fnActiveRAFbase / (fnActiveRAFbase /. drel → 0)) /. {RAFrel → RAF / Kdim, drel → d / Kd},
    (fnActiveRAF1433CAS / (fnActiveRAF1433CAS /. drel → 0)) /. {RAFrel → RAF / Kdim,
      Srel → STOT / Ksm, drel → d / Kd}, ((fnActiveRAF1433DS / (fnActiveRAF1433DS /. drel → 0)) /.
       {RAFrel → RAF / Kdim, srel → s / KsD, drel → d / Kd}) /. numsol23As,
    (fnActiveRAF1433 / (fnActiveRAF1433 /. drel → 0) /. {RAFrel → RAF / Kdim, drel → d / Kd} /.
      numsol24As)} /. baseparams;
```

In[95]:=
```
legendlist = Sty["AK\n"];
rvals = {0.07, 0.03, 2., 0.02};
r1 = {Medium, Small, Tiny, Large};
cols = {Green, Orange, Blue, Black};
cs = Table[Directive[ Thickness[0.01], cols[[i]], Dashing[{rvals[[i]], r1[[i]]}]], {i, 4}];
(*dashing style by model*)
LogPlot[plfn1, {d, 0, 100}, Frame -> True, ImageSize -> {500},
 FrameStyle -> Thickness[0.004], FrameTicksStyle -> Directive[25, Black],
 PlotRange -> Full, PlotStyle → cs, PlotLegends -> {"no 14-3-3", "CAS", "DS", "CAS+DS"}]
```

Out[100]=

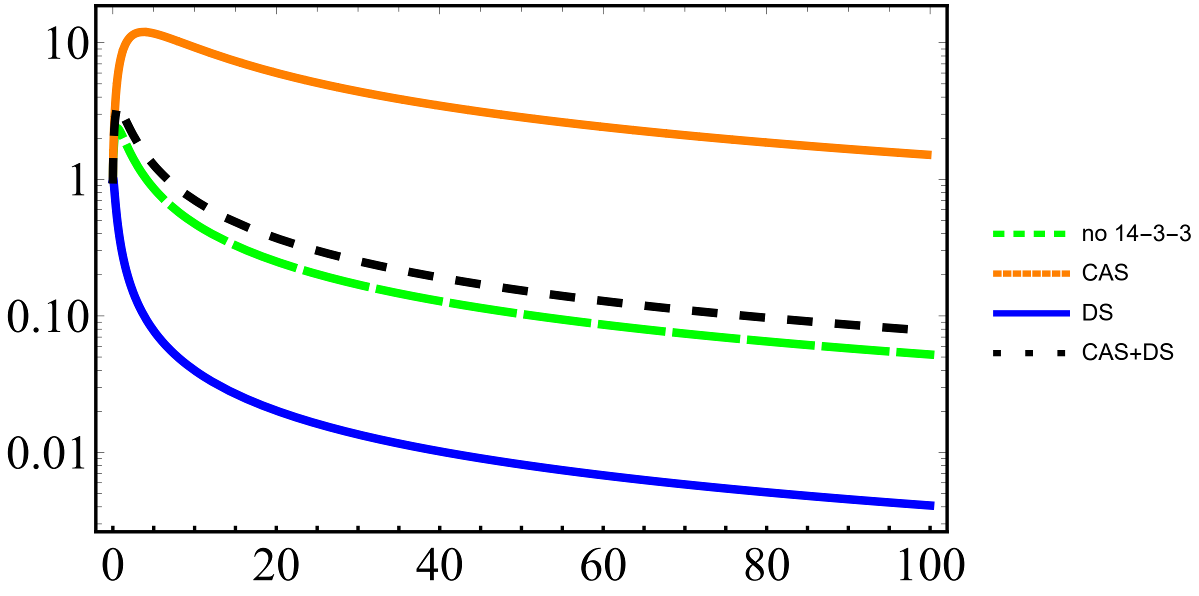

In[101]:=
```
fn2part[1] =
  SimplifyPars[({a, AA, AAd, AdAd, A, Ad} / RAF /. rep21 /. sol21A) /. drel → (d / Kd)] /. baseparams;
fn2part[2] = SimplifyPars[({a + as, AA, AAd, AdAd, A, Ad} / RAF /. rep22 /. sol22A) /. drel → (d / Kd)] /.
   baseparams;
fn2part[3] = ({a, AA + AAs, AAd + AAsd, AdAd + AAsdd, A, Ad} / RAF /. rep23 /. numsol23As) /.
    drel → (d / Kd) /. baseparams;
fn2part[4] = ({a + as, AA + AAs, AAd + AAsd, AdAd + AAsdd, A, Ad} / RAF /. rep24 /. numsol24As) /.
    drel → (d / Kd) /. baseparams;
plfn = Table[fn2part[i][[j]], {j, 6}, {i, 4}];
legendlist = Sty /@ { "a\n", "AA\n", " AAd\n", " AdAd\n", " A\n", " Ad\n"};
Table[Plot[Evaluate[plfn[[i]]], {d, 0, 100}, Frame -> True, ImageSize -> {500},
  FrameStyle -> Thickness[0.004], FrameTicksStyle -> Directive[25, Black],
  PlotRange -> Full, PlotStyle → cs, PlotLegends -> legendlist[[i]], {i, Length[plfn]}]
```

Out[107]= {

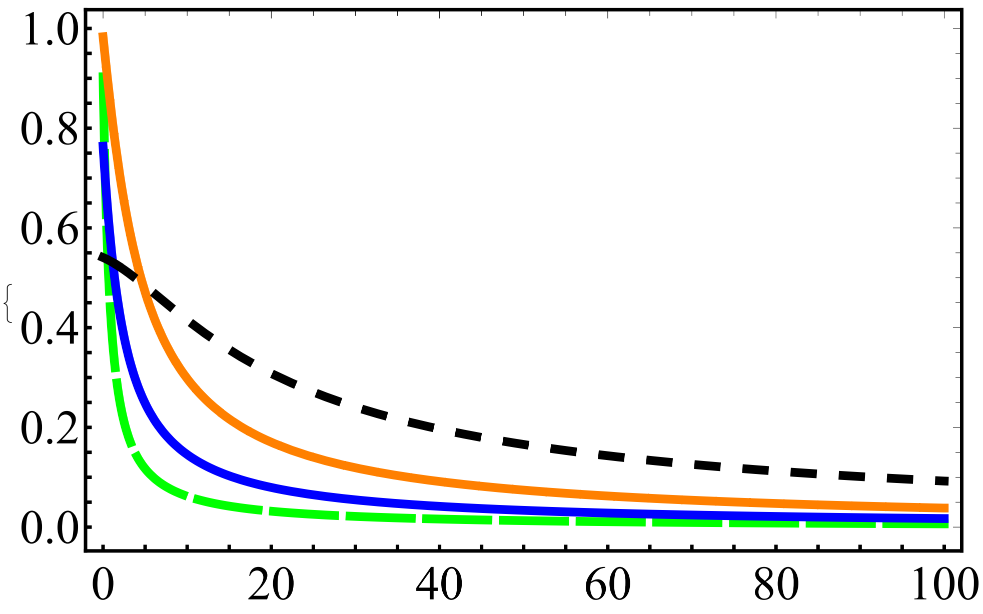
1.0
0.8
0.6
0.4
0.2
0.0
0
20
40
60
80
100

a ,

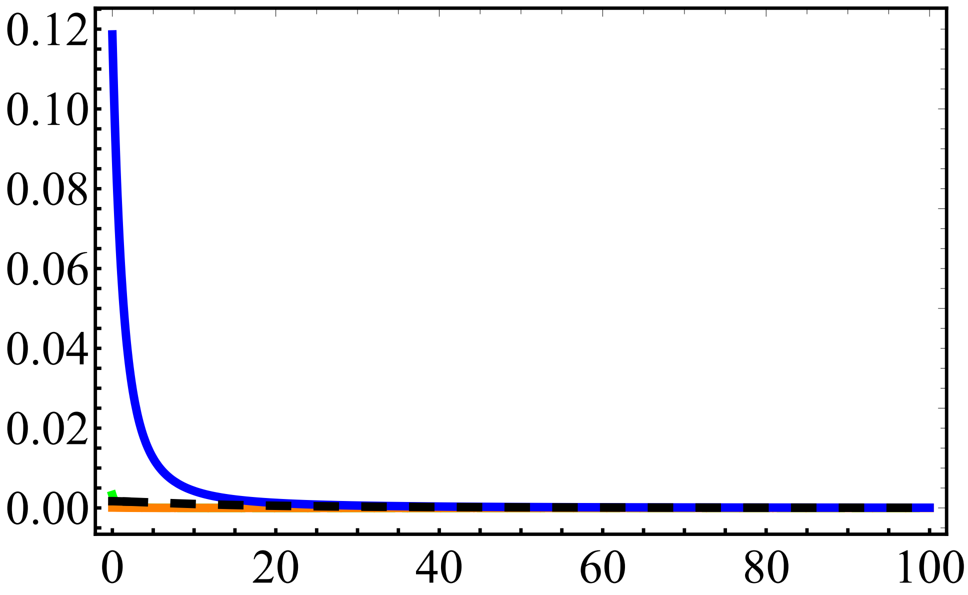
0.12
0.10
0.08
0.06
0.04
0.02
0.00
0
20
40
60
80
100

AA ,

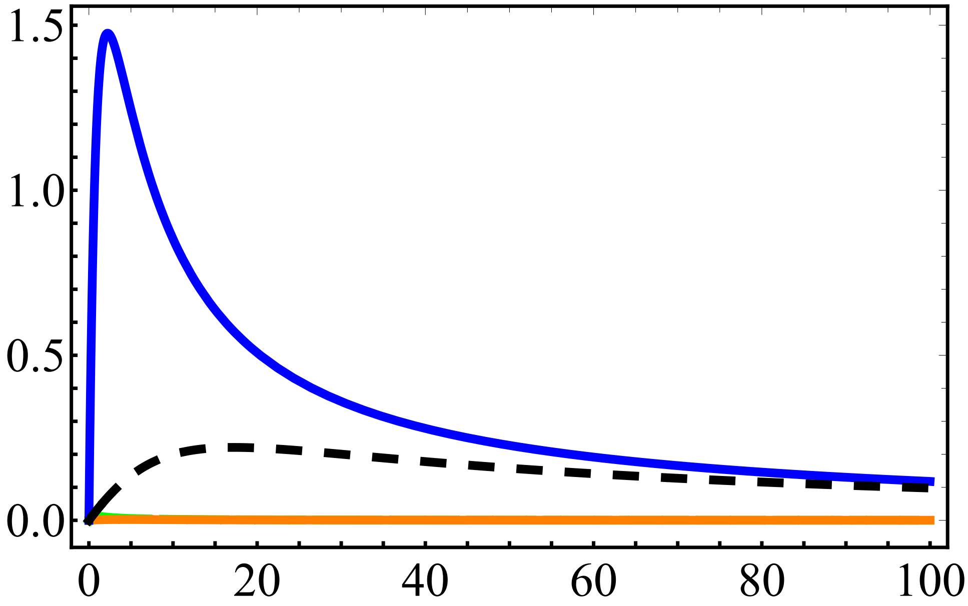

AAd ,

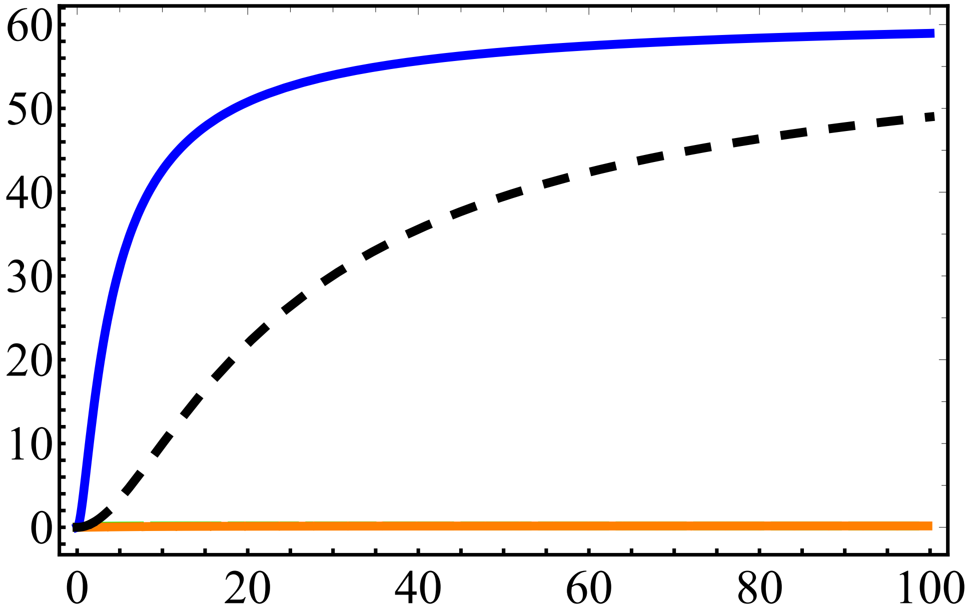

AdAd ,

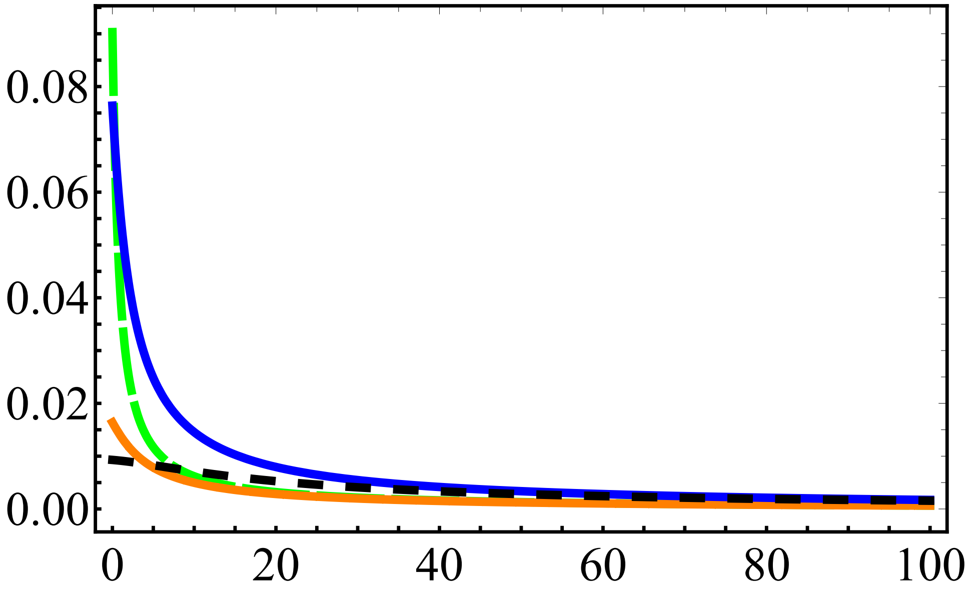

A ,

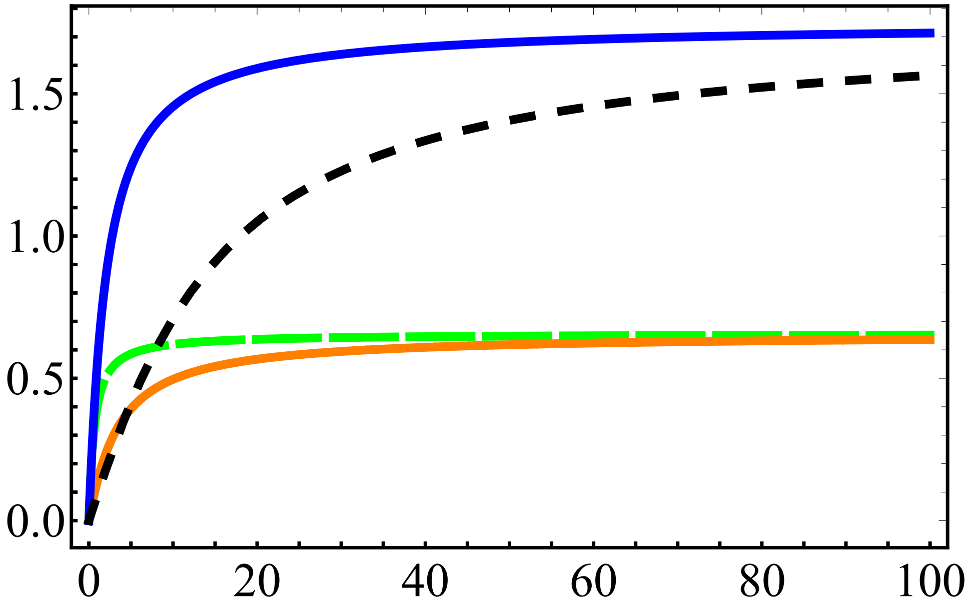

Ad }

{
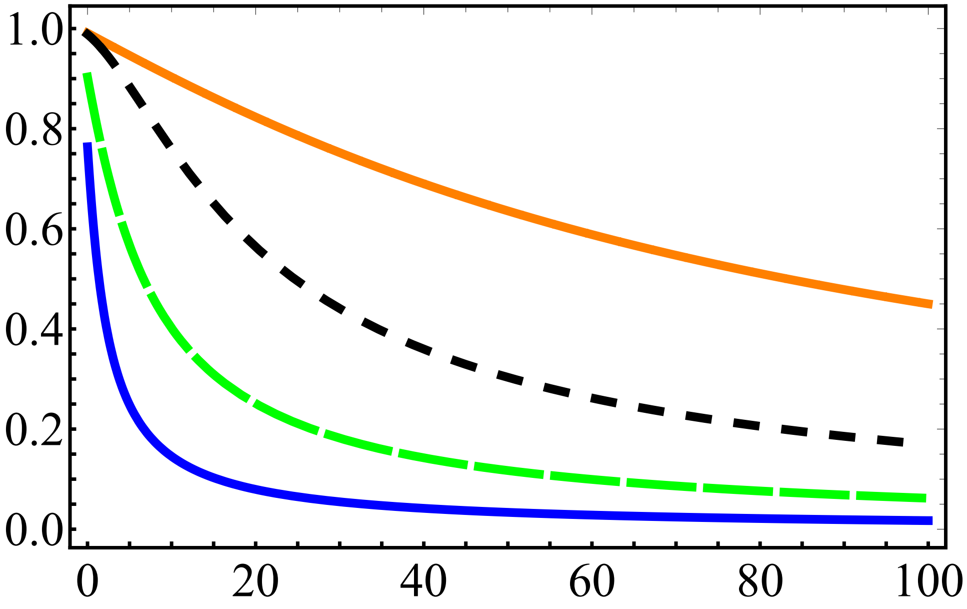
1.0
0.8
0.6
0.4
0.2
0.0
0
20
40
60
80
100

"a\n",

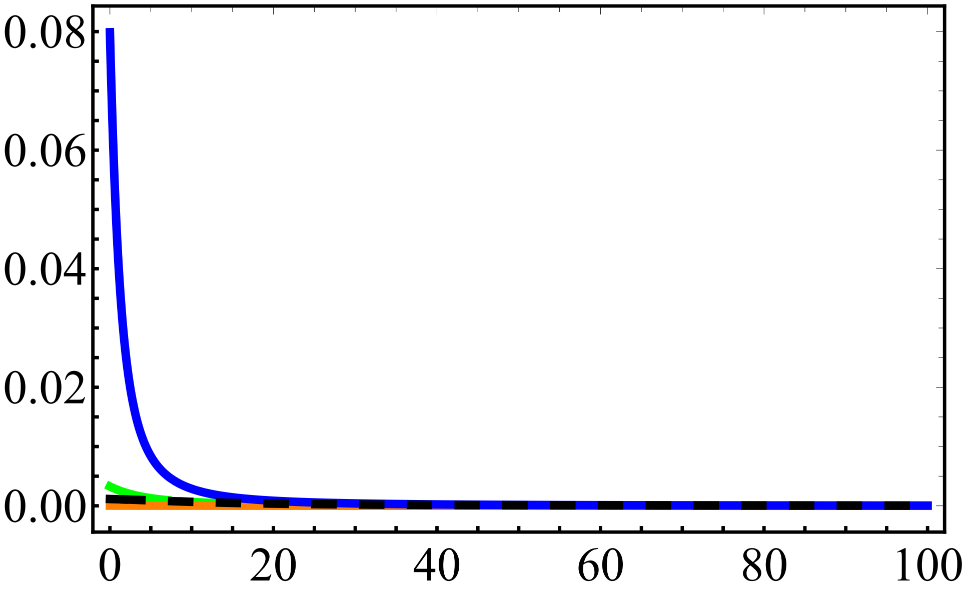
0.08
0.06
0.04
0.02
0.00
0
20
40
60
80
100

"AA\n",

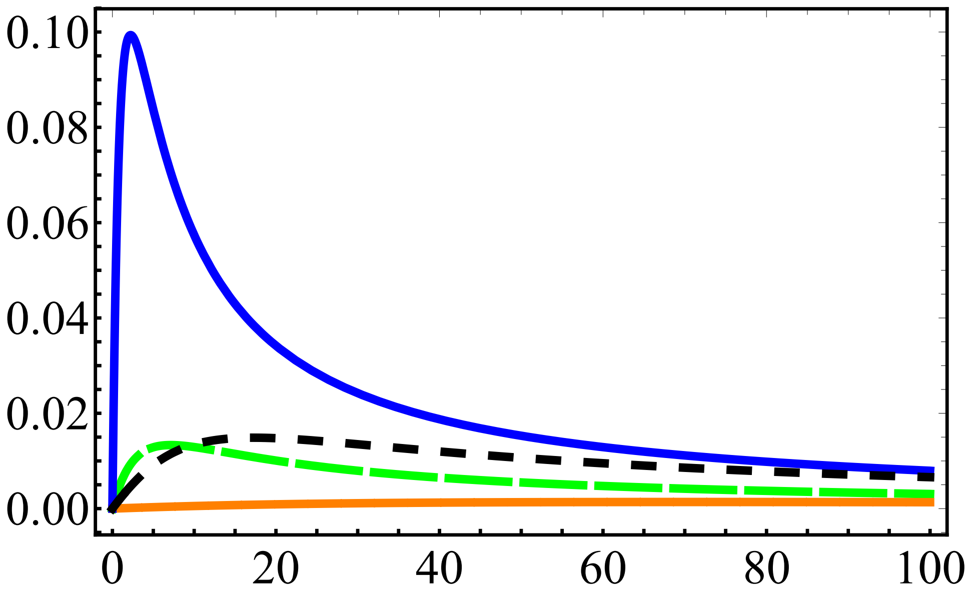
0.10
0.08
0.06
0.04
0.02
0.00
0
20
40
60
80
100

" AAd\n",

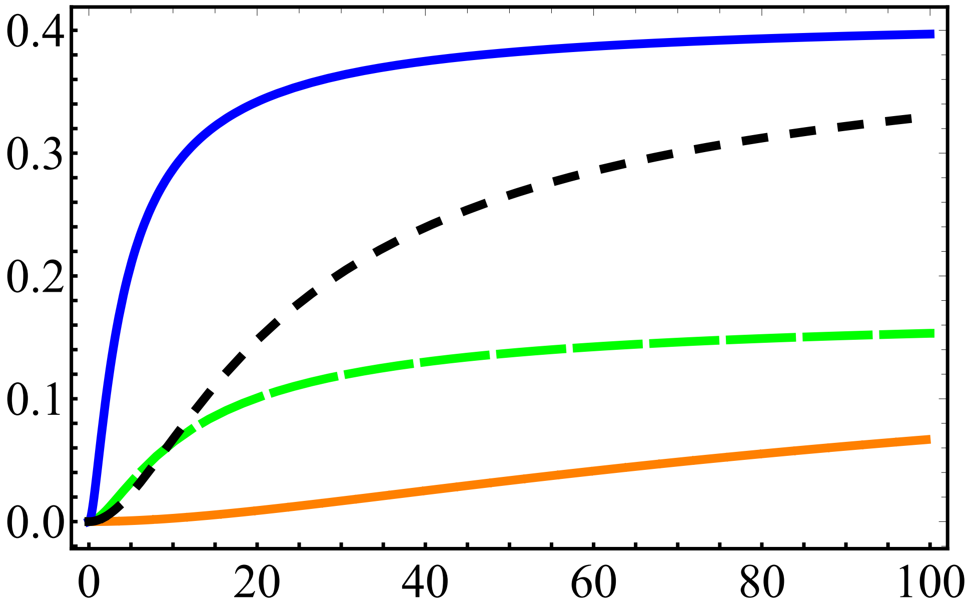
0.4
0.3
0.2
0.1
0.0
0
20
40
60
80
100

" AdAd\n",

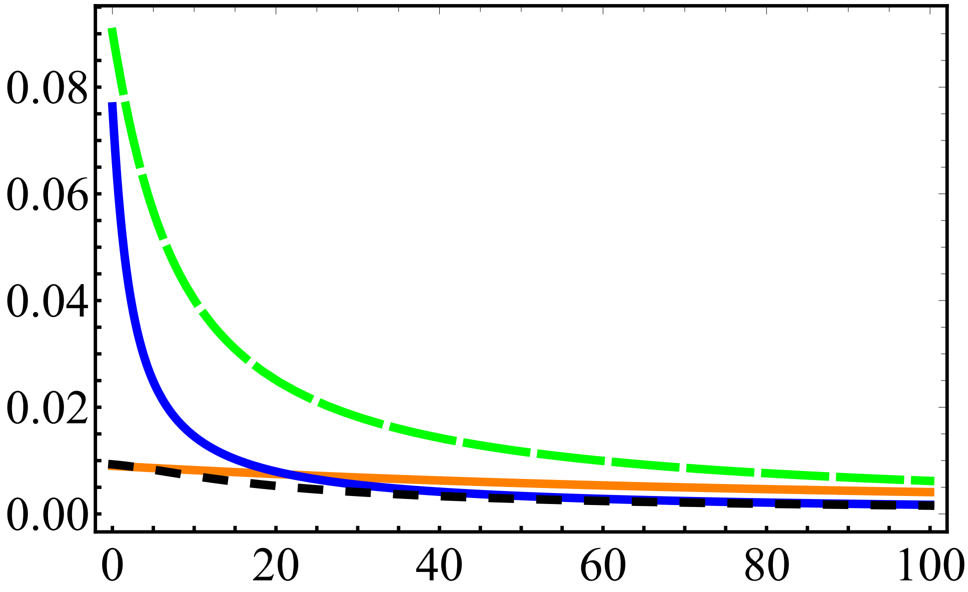
0.08
0.06
0.04
0.02
0.00
0
20
40
60
80
100

" A\n",

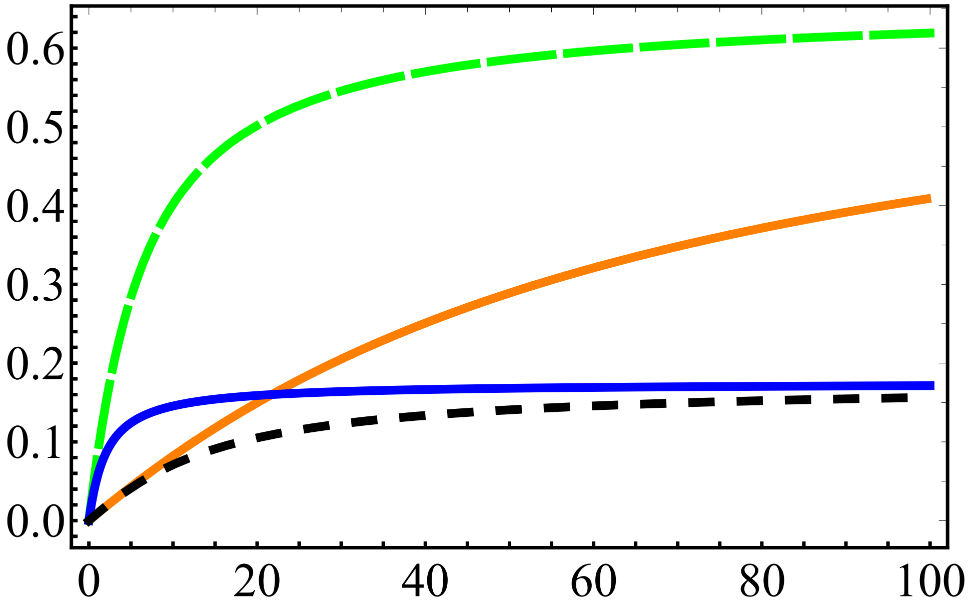
0.6
0.5
0.4
0.3
0.2
0.1
0.0
0
20
40
60
80
100

" Ad\n"}
