# Supplementary material for: Mathematical modeling suggests 14-3-3 proteins modulate RAF paradoxical activation
Source: PLoS Comput Biol. 2025 Aug 1;21(8):e1013297. doi: 10.1371/journal.pcbi.1013297 (PMC12407542; doi:10.1371/journal.pcbi.1013297)
Supplement: S1 Data — Supplementary files that include the code required to analyze and evaluate the models and to reproduce all of the results presented in this study. (ZIP) [file pcbi.1013297.s005.zip › Supplementary Data Mendiratta RAF 14-3-3/Code/RAF_1433_Roles/Generate_Plots_14-3-3CASmodel Figures2 and S1.pdf]

Generate_Plots_14-3-3CASmodel Figures2 and S1

January 26, 2025

[1]:
```
#Import Libraries
import numpy as np
import scipy.optimize as so
import matplotlib.pyplot as plt
import os
```

0.1 Model Definitions: CAS model

[2]:
```
DTOTMIN=10**-5 #micro-M = 100 pM
def checkpos(x,f=None,accept=None):
    """Returns 'True' if all elements in a list are positive numbers greater␣
 ↪than 10^-7 else False"""
    flag=True
    if type(x) is dict:
        x=list(x.values())
    for elem in x:
        if elem<=10**-7:
            flag=False
    return flag
def arrcompare(x1,x2):
    """returns the relative difference between corresponding values in lists x1␣
 ↪and x2. """
    try:
        res=[]
        for i in range(len(x1)):
            res=res+[(x1[i]-x2[i])/x1[i]]
        return res
    except: return 'ERROR'
def rafeqn(dr,A,params):
    raftoteqn=params['RAFTOT']-(A*(params['KA'] + (params['KA']*params['STOT'])/
 ↪(A*params['KA'] + params['KS']) + ((1 + dr)*(2*A + params['Kdim'] + 2*A*dr))/
 ↪params['Kdim']))
    return raftoteqn
def solA(dr,params):
    toteq=lambda A:rafeqn(dr,A,params) # initialize total equations with␣
 ↪parameters
```

```
    resas=so.brentq(toteq,0.,params['RAFTOT']) # solve the RAFTOT equation for␣
↪values of unbound raf protomers (A)
    return resas
def actkin(dr,params):
    """This function inputs dr=unbound-drug/Kd and parameter set to output the␣
↪active kinase in proportion to total raf kinase."""
    try:
        if dr>=0. and checkpos(list(params.values())):
            A=solA(dr,params)
            ak=((1 + dr)*2*A**2)/(params['Kdim']*params['RAFTOT'])
            return ak
    except:
        print("ERROR actkin:",dr,params)
        return 0
def dr2DTOT(dr,params):
    """Inputs unbound drug concentration alongwith a dictionary of parameters␣
↪to return the total drug concentration"""
    try:
        A=solA(dr,params)
        return (dr*(2*A**2 + (A + params['Kd'])*params['Kdim'] + 2*A**2*dr))/
↪params['Kdim']
    except:
        print("ERROR: d2DTOT: ",dr,params)
def DTOT2dr(DTOT,params):
    """Numerically solves the inverse function dtot2DTOT to convert input total␣
↪drug concentration and parameters into unbound drug conccentration"""
    try:
        if DTOT>DTOTMIN and checkpos(list(params.values())):
            objfn=lambda dr: dr2DTOT(dr,params)-DTOT
            drmax=DTOT/params['Kd'] # d is always smaller than DTOT
            return so.brentq(objfn,1.*10**-12,drmax)
        else:
            return 0
    except:
        print("ERROR DTOT2dr:",DTOT,params)
def DTOT2AK(DTOT,params):
#     print("DTOT,params",DTOT,params)
    if DTOT is None:
        dr=0.
        DTOT=0.
    try:
        if checkpos(list(params.values())) is False:
            return 10**10
        elif DTOT>=DTOTMIN:
            dr=DTOT2dr(DTOT,params)
        else:
            dr=0.
```

```python
        return actkin(dr,params)
    except:
        print("ERROR DTOT2AK:",DTOT,params)
def DTOT2AKnorm(DTOT,params):
    """This function inputs total drug values (in uM or same units as Kd in params), paramteres to output active RAF protomers normalized to no-drug"""
    return DTOT2AK(DTOT,params)/actkin(0,params)


def solrange(params):
    """This function inputs a set of absolute parameters and finds the solution for total drug concentration corresponding to maxima, maximal fold change and total drug concentration at which the drug becomes an inhibitor (activity levels equal drug free levels). Drug concentrations are given in micro molar."""
    mindbound=0.0001
    one=1-mindbound
    kinref=actkin(0.,params)
    objfn=lambda dr:kinref/actkin(dr,params)
    try:
        res=so.minimize_scalar(objfn,bounds=(mindbound,10.**5))
        if (res.x<1) or (res.success is False):
            res=so.minimize_scalar(objfn,bounds=(mindbound,1),method='Bounded')
        drroot=res.x
    except:# This exception handles cases that are essentially pure-inhibitors (hence minimization function fails)
        if objfn(mindbound)>one:
            drroot=0.
    kinmax=actkin(drroot,params)
    foldchange=kinmax/kinref
    if foldchange>one:
        Droot=dr2DTOT(drroot,params)
        AKref=DTOT2AK(0.,params)
        objfn1=lambda Dtot:(DTOT2AK(Dtot,params)-AKref)/AKref
        try:
            width=so.brentq(objfn1,Droot,10**6.) # to handle cases when the prameter values are smaller
        except:
            width=so.brentq(objfn1,Droot,10**9.) # to handle very high values of STOT and KA
        if Droot>10**-12:
            return Droot,foldchange,width
        else:
            return 0,0,0
    else:
        return 0,0,0
```

```
params0={'KA':10.,'Kd':0.1,'Kdim':0.1,'RAFTOT':0.04,'KS':0.2,'STOT':1.}#␣
↪dimensionful rates are in micro-Molar and sec
rafeqn(1.1,0.001,params0),solA(1.1,params0),actkin(1.1,params0),dr2DTOT(1.
↪1,params0),DTOT2dr(0.1104115,params0)
```

[2]:
```
(-0.019807247619047 62,
 0.0006605043206689,
 0.00045807925550339 93,
 0.11074671023997798,
 1.0966703638580144)
```

[3]:
```
# Define total dimer functions
def DTOT2Dimers(DTOT,params):
    try:
        if checkpos(list(params.values())) is False:
            return 10**10
        elif DTOT>=DTOTMIN:
            dr=DTOT2dr(DTOT,params)
        else:
            dr=0.
        return actkin(dr,params)*(1+dr)/2.
    except:
        print("ERROR DTOT2Dimers:",DTOT,params)
def DTOT2DimersNorm(DTOT,params):
    """This function inputs total drug values (in uM or same units as Kd in␣
↪params), paramteres to output active RAF protomers normalized to no-drug"""
    return DTOT2Dimers(DTOT,params)*2./actkin(0,params)
```

0.2 Solve Model

0.3 Contour Plots : Total drug

[4]:
```
%%time
paramsbase=dict(params0)# values in micro-molar
npts=20000 # total number of points to plot
npts=int(np.sqrt(npts)) # square root of the number of points to put on a␣
↪square grid
xlist = np.linspace(-1.0, 2.0,npts)
ylist = np.linspace(-3.0, 2.0,npts)
X, Y = np.meshgrid(xlist, ylist)
def callfnr(x1,y1):
    params1=dict(paramsbase)
    params1['RAFTOT']=10**y1
    params1['KA']=10**x1

    try:
        resarr=solrange(params1)
```

```
        if resarr[0]>10**-5:
            return [np.log10(resarr[1]),np.log10(resarr[2])]
        else:
            return [float('nan'),float('nan')]
    except:
        return [float('nan'),float('nan')]
zfc=[]
zr=[]
for itr in range(len(X)):
    zfc=zfc+[[]]
    zr=zr+[[]]
    for jtr in range(len(X[itr])):
        res=callfnr(X[itr][jtr],Y[itr][jtr])
        zfc[itr]=zfc[itr]+[res[0]]
        zr[itr]=zr[itr]+[res[1]]
```

```
CPU times: total: 1min 17s
Wall time: 2min 24s
```

0.4 Figure 2C

[5]:
```
font = {'family' : 'Arial',
        'size'   : 35}
plt.rc('font', **font)
plt.figure(figsize=(7,6))
colormaptype='viridis'
fileid="range"
minlevel=-1
levels=[minlevel]+[i/10 for i in range(0,60,10)]
cp = plt.contourf(X,Y,zr,levels=levels,cmap=colormaptype)
plt.contour(cp,colors='k',linewidths=1.5)
plt.colorbar(cp)
minlevel=0.007
lc=plt.contour(X,Y,zfc,colors='r',linewidths=5.
 ↪,levels=[minlevel],linestyles='solid')
plt.xlabel('Log10(KA)')
plt.ylabel(r'Log10(RAF ($\mu$M))')
# figname="DAK_1433full_DTOT_"+fileid+"_RAFrvsKA.pdf"
# plt.savefig(figname,dpi=300)
plt.show()
```

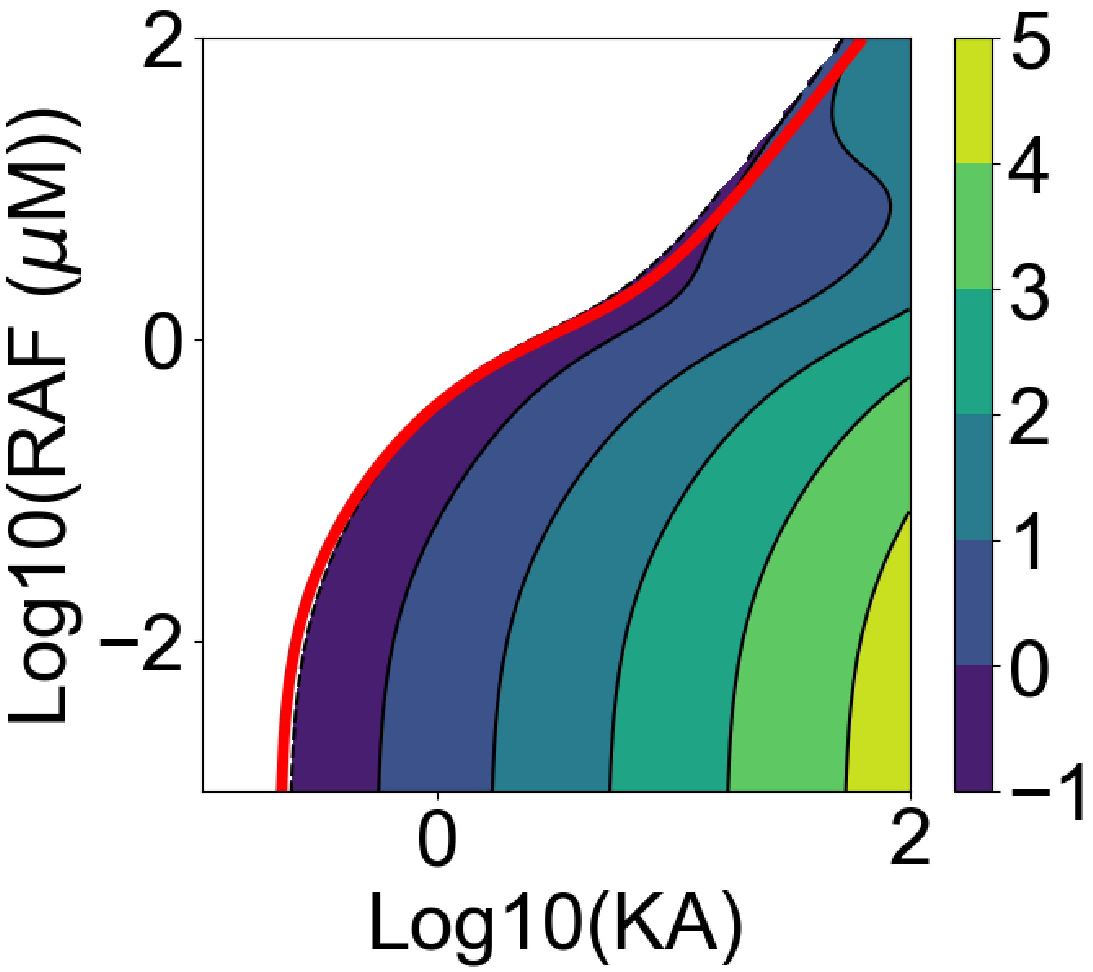

0.4.1 Figure S1C

[6]:
```
colormaptype='gnuplot'
fileid="foldchange"
plt.figure(figsize=(7,6))
minlevel=0.03
levels=[minlevel]+[i/10 for i in range(5,26,5)]
cp = plt.contourf(X,Y,zfc,levels=levels,cmap=colormaptype)
plt.contour(cp,colors='k',linewidths=1.5)
plt.colorbar(cp)
lc=plt.contour(cp,colors='r',linewidths=4.,levels=[minlevel],linestyles='solid')
plt.xlabel('Log10(KA)')
plt.ylabel('Log10(Total RAF [uM])')
# figname="DAK_1433full_"+fileid+"_RAFrvsKA.pdf"
# plt.savefig(figname,dpi=300)
plt.show()
```

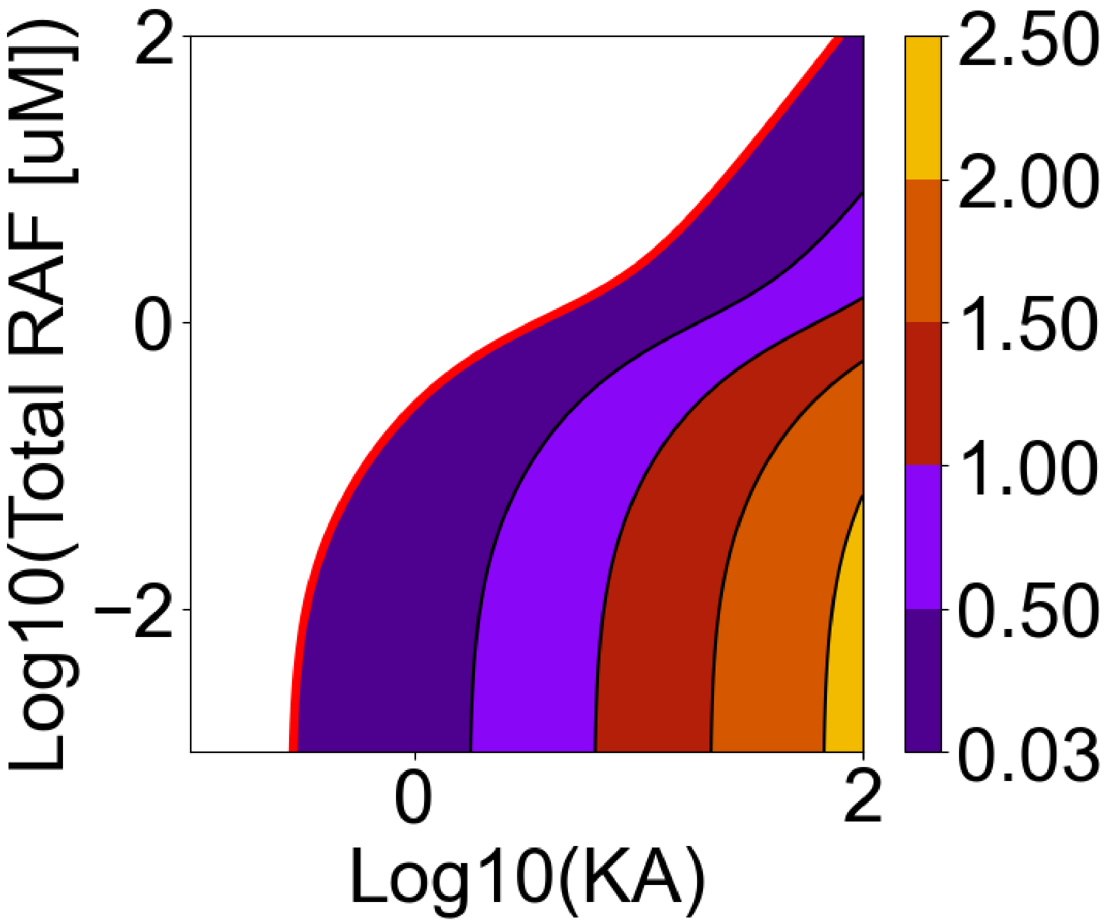

0.4.2 fix RAF and vary 14-3-3

[7]:
```
%%time
paramsbase=dict(params0)# values in micro-molar
npts=10000 # total number of points to plot
npts=int(np.sqrt(npts)) # square root of the number of points to put on a␣
  ↪square grid
xlist = np.linspace(-1.0, 2.0,npts)
ylist = np.linspace(-3.0, 2.0,npts)
X, Y = np.meshgrid(xlist, ylist)
def callfnr(x1,y1):
    params1=dict(paramsbase)
    params1['STOT']=10**y1
    params1['KA']=10**x1

    try:
        resarr=solrange(params1)
        if resarr[0]>10**-5:
```

```
            return [np.log10(resarr[1]),np.log10(resarr[2])]
        else:
            return [float('nan'),float('nan')]
    except:
        return [float('nan'),float('nan')]
zfc=[]
zr=[]
for itr in range(len(X)):
    zfc=zfc+[[]]
    zr=zr+[[]]
    for jtr in range(len(X[itr])):
        res=callfnr(X[itr][jtr],Y[itr][jtr])
        zfc[itr]=zfc[itr]+[res[0]]
        zr[itr]=zr[itr]+[res[1]]
```

```
CPU times: total: 35.5 s
Wall time: 1min 3s
```

0.5 Figure 2C

[8]:

```
font = {'family' : 'Arial',
        'size'   : 35}
plt.rc('font', **font)
plt.figure(figsize=(7,6))
colormaptype='viridis'
fileid="range"
minlevel=-1.5
levels=[minlevel]+[i/10 for i in range(0,91,15)]
cp = plt.contourf(X,Y,zr,levels=levels,cmap=colormaptype)
plt.contour(cp,colors='k',linewidths=1.5)
plt.colorbar(cp)
minlevel=0.001
lc=plt.contour(X,Y,zfc,colors='r',linewidths=5.
 ↪,levels=[minlevel],linestyles='solid')
plt.xlabel('Log10(KA)')
plt.ylabel('Log10([14-3-3] (uM))')
figname="DAK_1433full_DTOT_"+fileid+"_STOTvsKA.pdf"
# plt.savefig(figname,dpi=300)
plt.show()
```

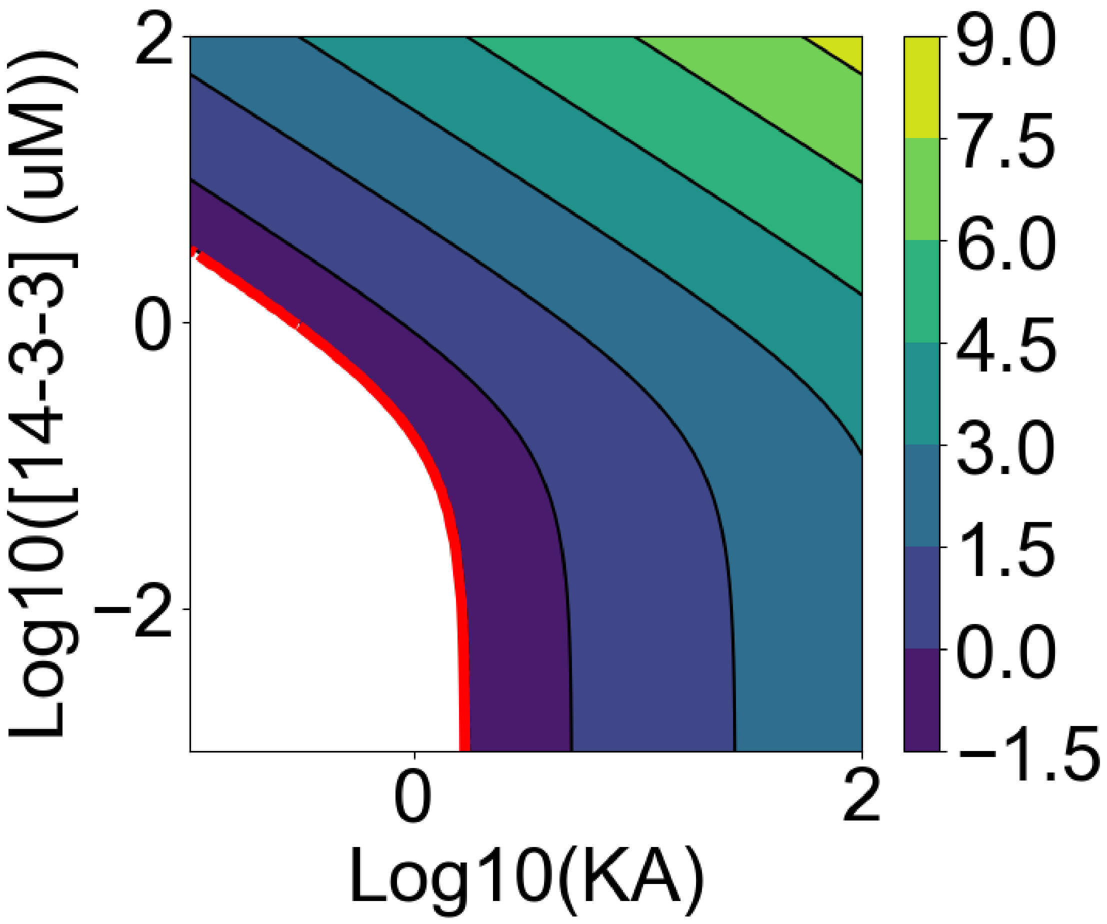

0.5.1 Figure S1D

[9]:
```
colormaptype='gnuplot'
fileid="foldchange"
plt.figure(figsize=(7,6))
minlevel=0
levels=[minlevel]+[i/10 for i in range(10,41,5)]
cp = plt.contourf(X,Y,zfc,levels,cmap=colormaptype)
plt.contour(cp,colors='k',linewidths=1.5)
plt.colorbar(cp)
minlevel=0.001
lc=plt.contour(cp,colors='r',linewidths=4.,levels=[minlevel],linestyles='solid')
# cp2 = plt.contourf(X1,Y1,Z1,levels)
plt.xlabel('Log10(KA)')
plt.ylabel('Log10([14-3-3] (uM))')
# figname="DAK_stableAI_DTOT_"+fileid+"_STOTvsKA.pdf"
plt.savefig(figname,dpi=300)
plt.show()
```

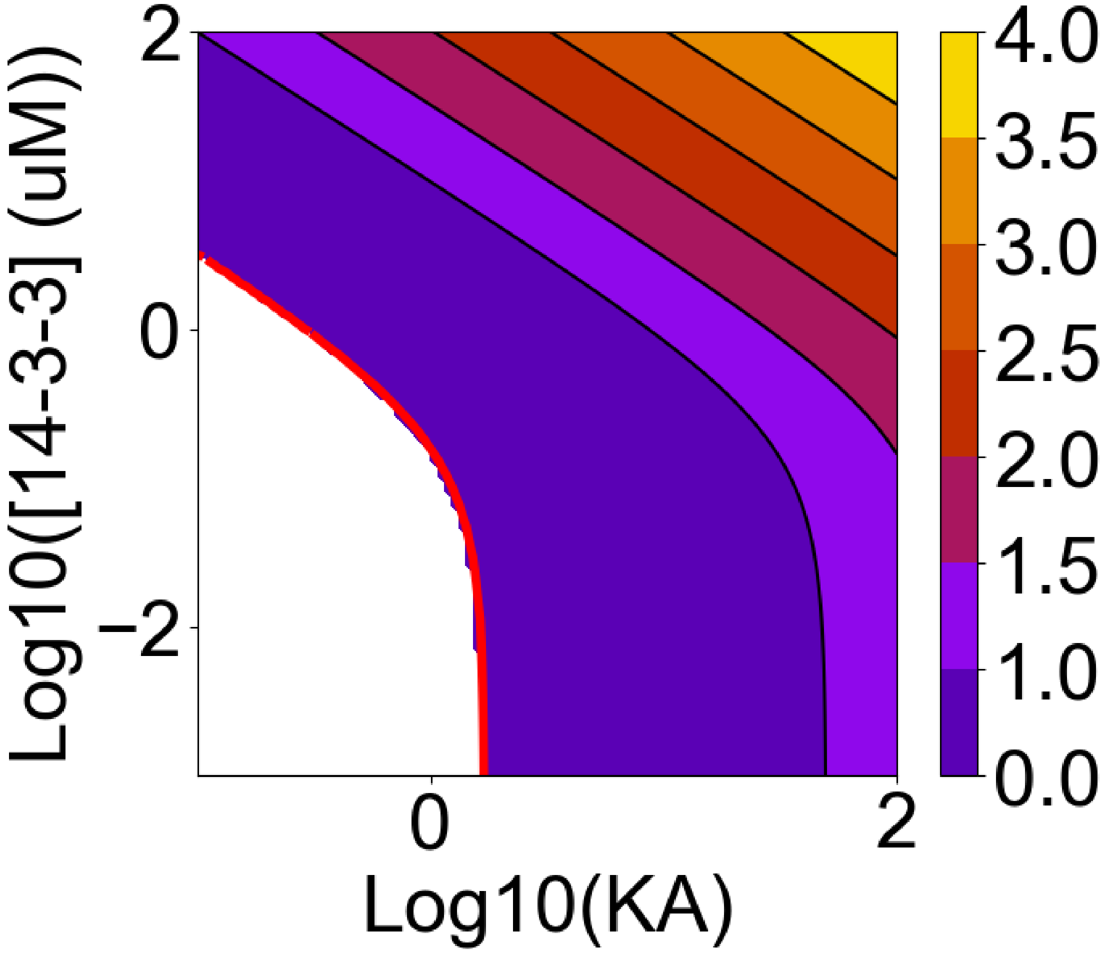

2
0
-2
Log10([14-3-3] (uM))
0
2
Log10(KA)
4.0
3.5
3.0
2.5
2.0
1.5
1.0
0.0
