# Supplementary material for: Mathematical modeling suggests 14-3-3 proteins modulate RAF paradoxical activation
Source: PLoS Comput Biol. 2025 Aug 1;21(8):e1013297. doi: 10.1371/journal.pcbi.1013297 (PMC12407542; doi:10.1371/journal.pcbi.1013297)

# Generate_Plots_14-3-3DualRoles_Figures2 and S1 and S2

January 26, 2025

[28]:
```
#Import Libraries
import numpy as np
import scipy.optimize as so
import matplotlib.pyplot as plt
import os
```

## 0.1 Model Definitions: DS+CAS 14-3-3 both roles model

[29]:
```
DTOTMIN=10**-5 #micro-M = 100 pM
def checkpos(x,f=None,accept=None):
    """Returns 'True' if all elements in a list are positive numbers greater␣
 ↪than 10^-7 else False"""
    flag=True
    if type(x) is dict:
        x=list(x.values())
    for elem in x:
        if elem<=10**-7:
            flag=False
    return flag
def arrcompare(x1,x2):
    """returns the relative difference between corresponding values in lists x1␣
 ↪and x2. """
    try:
        res=[]
        for i in range(len(x1)):
            res=res+[(x1[i]-x2[i])/x1[i]]
        return res
    except: return 'ERROR'

def rafeqn(dr,A,params):
```

```
    raftoteqn=params['RAFTOT']-((A*(2*A + params['Kdim'] +␣
↪params['KA']*params['Kdim']) + 2*params['Kdim']*params['STOT'] + A*dr*(4*A +␣
↪params['Kdim'] + 2*A*dr) -␣
↪(params['Kdim']**2*params['KSdim']*(A*params['KA'] +␣
↪2*params['KSmon'])*params['STOT'])/
↪(A*params['KA']*params['Kdim']*params['KSdim'] + A**2*params['KSmon'] +␣
↪params['Kdim']*params['KSdim']*params['KSmon'] + A**2*params['KSmon']*dr*(2␣
↪+ dr)))/params['Kdim'])
    return raftoteqn
def solA(dr,params):
    toteq=lambda A:rafeqn(dr,A,params) # initialize total equations with␣
↪parameters
    resas=so.brentq(toteq,0.,params['RAFTOT']) # solve the STOT and RAFTOT␣
↪equations for values of unbound raf protomers (A) and unbound stabilizing␣
↪protein
    return resas
def actkin(dr,params):
    """This function inputs dr=unbound-drug/Kd and parameter set to output the␣
↪active kinase in proportion to total raf kinase."""
    try:
        if dr>=0. and checkpos(list(params.values())):
            A=solA(dr,params)
            ak=(2*A**2*(1 + dr)*(A*params['KA']*params['Kdim']*params['KSdim']␣
↪+ A**2*params['KSmon'] + params['Kdim']*params['KSmon']*(params['KSdim'] +␣
↪params['STOT']) + 2*A**2*params['KSmon']*dr + A**2*params['KSmon']*dr**2))/
↪(params['Kdim']*params['RAFTOT']*(A*params['KA']*params['Kdim']*params['KSdim']␣
↪+ A**2*params['KSmon'] + params['Kdim']*params['KSdim']*params['KSmon'] +␣
↪2*A**2*params['KSmon']*dr + A**2*params['KSmon']*dr**2))
            return ak
    except:
        print("ERROR actkin:",dr,params)
        return 0
def dr2DTOT(dr,params):
    """Inputs unbound drug concentration alongwith a dictionary of parameters␣
↪to return the total drug concentration"""
    try:
        A=solA(dr,params)
        return dr*(A + params['Kd'] + (2*A**2*(1 + dr)*(params['KSdim'] +␣
↪(params['Kdim']*params['KSdim']*params['KSmon']*params['STOT'])/
↪(A*params['KA']*params['Kdim']*params['KSdim'] + A**2*params['KSmon'] +␣
↪params['Kdim']*params['KSdim']*params['KSmon'] + A**2*params['KSmon']*dr*(2␣
↪+ dr))))/(params['Kdim']*params['KSdim']))
    except:
        print("ERROR: d2DTOT: ",dr,params)
def DTOT2dr(DTOT,params):
```

```python
    """Numerically solves the inverse function dtot2DTOT to convert input total drug concentration and parameters into unbound drug conccentration"""
    if DTOT>10**8:
        DTOT=int(DTOT) # for very large numbers, the full floating point precision crashes optimization codes and the decimal digits are irrelevant to such large numbers anyway.
    try:
        if DTOT>DTOTMIN and checkpos(list(params.values())):
            objfn=lambda dr: dr2DTOT(dr,params)-DTOT
            drmax=DTOT/params['Kd'] # d is always smaller than DTOT
            return so.brentq(objfn,1.*10**-12,drmax)
        else:
            return 0
    except:
        print("ERROR DTOT2d_2:",DTOT,params)
def DTOT2AK(DTOT,params):
#     print("DTOT,params",DTOT,params)
    if DTOT is None:
        dr=0.
        DTOT=0.
    try:
        if checkpos(list(params.values())) is False:
            return 10**10
        elif DTOT>=DTOTMIN:
            dr=DTOT2dr(DTOT,params)
        else:
            dr=0.
        return actkin(dr,params)
    except:
        print("ERROR DTOT2AK:",DTOT,params)
def DTOT2AKnorm(DTOT,params):
    """This function inputs total drug values (in uM or same units as Kd in params), paramteres to output active RAF protomers normalized to no-drug"""
    return DTOT2AK(DTOT,params)/actkin(0,params)


def solrange(params):
    """This function inputs a set of absolute parameters and finds the solution for total drug concentration corresponding to maxima, maximal fold change and total drug concentration at which the drug becomes an inhibitor (activity levels equal drug free levels). Drug concentrations are given in micro molar."""
    mindbound=0.0001
    one=1-mindbound
    kinref=actkin(0.,params)
    objfn=lambda dr:kinref/actkin(dr,params)
    try:
```

```
        res=so.minimize_scalar(objfn,bounds=(mindbound,10.**5))
        if (res.x<1) or (res.success is False):
            res=so.minimize_scalar(objfn,bounds=(mindbound,1),method='Bounded')
        drroot=res.x
    except:# This exception handles cases that are essentially pure-inhibitors␣
 ↪(hence minimization function fails)
        if objfn(mindbound)>one:
            drroot=0.
    kinmax=actkin(drroot,params)
    foldchange=kinmax/kinref
    if foldchange>one:
        Droot=dr2DTOT(drroot,params)
        AKref=DTOT2AK(0.,params)
        objfn1=lambda Dtot:(DTOT2AK(Dtot,params)-AKref)/AKref
        try:
            width=so.brentq(objfn1,Droot,10**6.) # to handle cases when the␣
 ↪prameter values are smaller
        except:
            width=so.brentq(objfn1,10**5.,10**12.) # to handle very high values␣
 ↪of STOT and KA
        if Droot>10**-12:
            return Droot,foldchange,width
        else:
            return 0,0,0
    else:
        return 0,0,0
# # define parameters where dimerization rate is just a bit more than␣
 ↪autoinhibition rate - as compatible with cell lines in our experiments (so␣
 ↪dissociation constant is reverse).
# define parameters with which to compare all results for reference:
params0={'KA':10.,'Kd':0.1,'Kdim':0.1,'RAFTOT':0.04,'KSmon':0.2,'KSdim':0.
 ↪02,'STOT':1.}# dimensionful rates are in micro-Molar and sec
rafeqn(1.1,0.001,params0),solA(1.1,params0),actkin(1.1,params0),dr2DTOT(1.
 ↪1,params0),DTOT2dr(dr2DTOT(1.1,params0),params0)
```

[29]: (-0.0238986557, 0.0006320874, 0.0207351114, 0.1116076410, 1.1000000000)

[30]:
```
%precision 12

def rafeqn_DS(dr,A,params):
    raftoteqn=params['RAFTOT']-(A*(1 + params['KA'] + (2*A)/params['Kdim']) +␣
 ↪2*params['STOT'] + (A*(4*A + params['Kdim'])*dr + 2*A**2*dr**2 -␣
 ↪(2*params['Kdim']**2*params['KSdim']*params['STOT'])/(A**2 +␣
 ↪params['Kdim']*params['KSdim'] + A**2*dr*(2 + dr)))/params['Kdim'])
    return raftoteqn

def solA_DS(d1,params):
```

```
    toteq=lambda A:rafeqn_DS(d1,A,params) # initialize total equations with parameters
    resas=so.brentq(toteq,0.,params['RAFTOT']) # solve the STOT and RAFTOT equations for values of unbound raf protomers (A) and unbound stabilizing protein
    return resas

def dr2DTOT_DS(dr,params):
    """Inputs unbound drug concentration alongwith a dictionary of parameters to return the total drug concentration"""
    try:
        A=solA_DS(dr,params)
        return dr*(A + params['Kd'] + (2*A**2*(1 + dr)*(params['KSdim'] + (params['Kdim']*params['KSdim']*params['STOT'])/(A**2 + params['Kdim']*params['KSdim'] + A**2*dr*(2 + dr))))/(params['Kdim']*params['KSdim']))
    except:
        print("ERROR: d2DTOT_DS: ",dr,params)

def actkin_DS(dr,params):
    """This function inputs d1=unbound-drug/Kd and parameter set to output the active kinase in proportion to total raf kinase. in the absence of any stabilization of auto-inhibition by RAF kinase"""
    try:
        if dr>=0. and checkpos(list(params.values())):
            A=solA_DS(dr,params)
            ak=(2*A**2*(1 + dr)*(A**2 + params['Kdim']*(params['KSdim'] + params['STOT']) + 2*A**2*dr + A**2*dr**2))/(params['Kdim']*params['RAFTOT']*(A**2 + params['Kdim']*params['KSdim'] + 2*A**2*dr + A**2*dr**2))
            return ak
    except:
        print("ERROR actkin_DS:",d1,params)
        return 0
params_ds=dict(params0)
rafeqn_DS(1.1,0.001,params_ds),solA_DS(1.1,params_ds),actkin_DS(1.1,params_ds),dr2DTOT_DS(1.1,params_ds)
```

[30]: (0.023411502656, 0.001930872738, 0.198052855598, 0.120838285658)

[31]:
```
%precision 10
# DS model limit of dual roles model when KSmon is large.
params1=dict(params0)
params1['KSmon']=1000000
rafeqn(1.1,0.001,params1),solA(1.1,params1),actkin(1.1,params1),dr2DTOT(1.1,params1)
```

[31]: (0.0234114927, 0.0019308721, 0.1980527206, 0.1208382790)

[32]:
```
def rafeqn_CAS(dr,A,params):
    raftoteqn=params['RAFTOT']-(A*(params['KA'] + (params['KA']*params['STOT'])/
↪(A*params['KA'] + params['KSmon']) + ((1 + dr)*(2*A + params['Kdim'] +␣
↪2*A*dr))/params['Kdim']))
    return raftoteqn

def solA_CAS(dr,params):
    toteq=lambda A:rafeqn_CAS(dr,A,params) # initialize total equations with␣
↪parameters
    resas=so.brentq(toteq,0.,params['RAFTOT']) # solve the RAFTOT equation for␣
↪values of unbound raf protomers (A)
    return resas

def dr2DTOT_CAS(dr,params):
    """Inputs unbound drug concentration alongwith a dictionary of parameters␣
↪to return the total drug concentration"""
    try:
        A=solA_CAS(dr,params)
        return (dr*(2*A**2 + (A + params['Kd'])*params['Kdim'] + 2*A**2*dr))/
↪params['Kdim']
    except:
        print("ERROR: d2DTOT_CAS: ",dr,params)

def actkin_CAS(dr,params):
    """This function inputs dr=unbound-drug/Kd and parameter set to output the␣
↪active kinase in proportion to total raf kinase."""
    try:
        if dr>=0. and checkpos(list(params.values())):
            A=solA_CAS(dr,params)
            ak=((1 + dr)*2*A**2)/(params['Kdim']*params['RAFTOT'])
            return ak
    except:
        print("ERROR actkin_CAS:",dr,params)
        return 0
#Verify above functions
rafeqn_CAS(1.1,0.001,params0),solA_CAS(1.1,params0),actkin_CAS(1.
↪1,params0),dr2DTOT_CAS(1.1,params0)
```

[32]: (-0.0198072476, 0.0006605043, 0.0004580793, 0.1107467102)

[33]:
```
%precision 10
# CAS model limit of dual roles model when KSdim is large.
params1=dict(params0)
params1['KSdim']=1000000
```

```
rafeqn(1.1,0.001,params1),solA(1.1,params1),actkin(1.1,params1),dr2DTOT_CAS(1.
↪1,params1)
```

[33]: (-0.0198072477, 0.0006605043, 0.0004580797, 0.1107467102)

## 0.2 Figure 2B

[34]:
```
# make an example plot for 14-3-3 model variations

font = {'family' : 'Arial',
        'size'   : 20}
plt.rc('font', **font)


paramsplt0=dict(params0) #units: micro-molar
paramsplt0['STOT']=0.000001
xdatr=[0.]+list(10**np.linspace(-2,4,300))
xdat=[dr2DTOT(elem,paramsplt0) for elem in xdatr]
ydat1= [actkin(xval,paramsplt0)/actkin(0,paramsplt0) for xval in xdatr] #␣
↪solution with no 14-3-3
plt.plot(xdat,ydat1,label='No 14-3-3',color='b',linewidth=5)

paramsplt0['STOT']=1. #
xdat=[dr2DTOT_CAS(elem,paramsplt0) for elem in xdatr]
ydat= [actkin_CAS(xval,paramsplt0)/actkin_CAS(0,paramsplt0) for xval in xdatr]␣
↪# solution with no alpha (dimerization stabilization)
plt.plot(xdat,ydat,label='Auto-Inhibition␣
↪Stabilization',color='orange',linewidth=5)

xdat=[dr2DTOT_DS(elem,paramsplt0) for elem in xdatr]
ydat2= [actkin_DS(xval,paramsplt0)/actkin_DS(0,paramsplt0) for xval in xdatr] #␣
↪solution with no Auto-inhibition stabilization, only alpha
plt.plot(xdat,ydat2,label='Dimer Stabilization',color='k',linewidth=5)

xdat=[dr2DTOT(elem,paramsplt0) for elem in xdatr]
ydat= [actkin(xval,paramsplt0)/actkin(0,paramsplt0) for xval in xdatr] #␣
↪solution
plt.plot(xdat,ydat,label='Both 14-3-3␣
↪roles',linestyle='dashed',color='r',linewidth=5)

plt.xscale('log')
plt.xlabel('RAF inhibitor ($\mu$M)')
plt.ylabel('Active RAF Normalized')
plt.legend(prop={'size':12},bbox_to_anchor=[1.,0.5])
plt.show()

paramsplt0=dict(params0) #units: micro-molar
```

```
# make an example plot for 14-3-3 model variations with RAF un-normalized
paramsplt0['STOT']=0.000001
xdat=[dr2DTOT(elem,paramsplt0) for elem in xdatr]
ydat= [actkin(xval,paramsplt0)*100 for xval in xdatr] # solution with no 14-3-3
plt.plot(xdat,ydat,label='No 14-3-3',color='b',linewidth=5)

paramsplt0['STOT']=1. #
xdat=[dr2DTOT_CAS(elem,paramsplt0) for elem in xdatr]
ydat= [actkin_CAS(xval,paramsplt0)*100 for xval in xdatr] # solution with no␣
↪alpha (dimerization stabilization)
plt.plot(xdat,ydat,label='Auto-Inhibition␣
↪Stabilization',color='orange',linewidth=5)

xdat=[dr2DTOT_DS(elem,paramsplt0) for elem in xdatr]
ydat= [actkin_DS(xval,paramsplt0)*100 for xval in xdatr] # solution with no␣
↪Auto-inhibition stabilization, only alpha
plt.plot(xdat,ydat,label='Dimer Stabilization',color='k',linewidth=5)

xdat=[dr2DTOT(elem,paramsplt0) for elem in xdatr]
ydat= [actkin(xval,paramsplt0)*100 for xval in xdatr] # solution
plt.plot(xdat,ydat,label='Both 14-3-3␣
↪roles',linestyle='dashed',color='r',linewidth=5)

plt.yscale('log')
plt.xscale('log')
plt.xlabel('RAF inhibitor ($\mu$M)')
plt.ylabel('Active RAF Percent')
plt.legend(prop={'size':12},bbox_to_anchor=[1.,0.5])
plt.show()
```

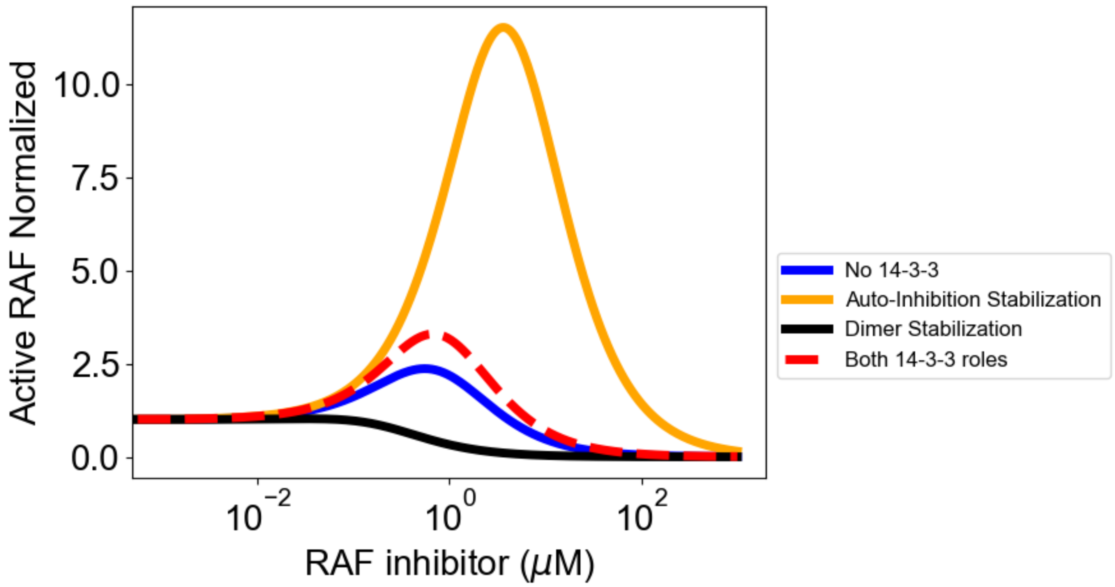

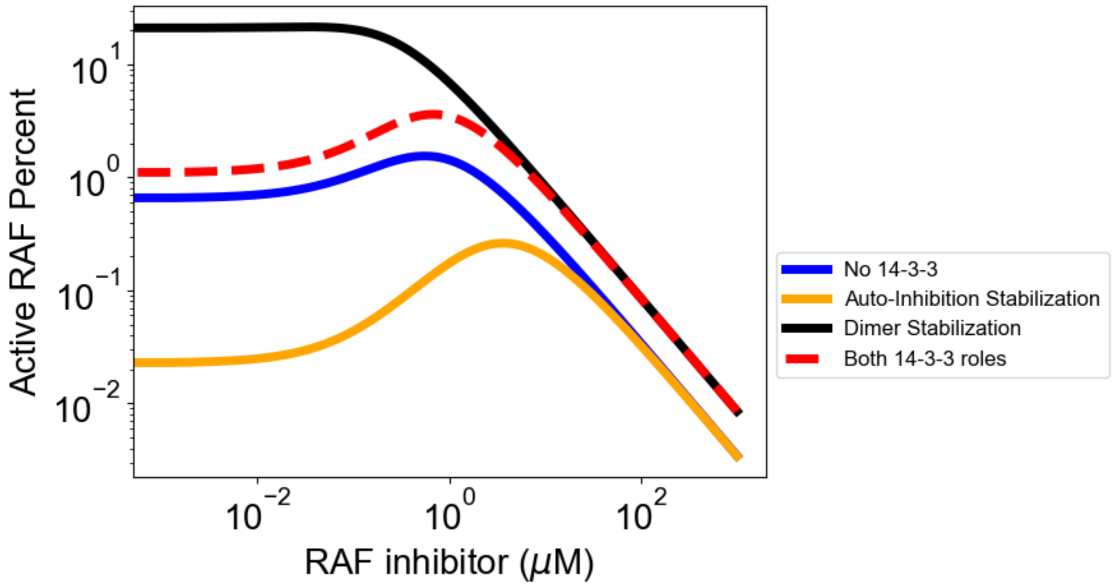


## 0.3 Figure S1A

[35]:
```
paramsplt0=dict(params0) #units: micro-molar
xdatr=[0.]+list(10**np.linspace(-2,4,300))

STOTvalslist=[0.000001,0.01,1,10.0]
STOTcolorlist=['b','orange','g','r']
ls=['solid','dashed','dashed','dashed']
labellist=[r'No 14-3-3','14-3-3:'+str(STOTvalslist[1])+'$\mu$M','14-3-3:
↪'+str(STOTvalslist[2])+'$\mu$M','14-3-3:'+str(STOTvalslist[3])+'$\mu$M']
for i in range(len(STOTvalslist)):
    paramsplt0['STOT']=STOTvalslist[i]
    ydat= [actkin(xval,paramsplt0)*100 for xval in xdatr]
    xdat=[dr2DTOT(elem,paramsplt0) for elem in xdatr]
    plt.
↪plot(xdat,ydat,label=labellist[i],color=STOTcolorlist[i],linestyle=ls[i],linewidth=5)

# plt.yscale('log')
plt.xscale('log')
plt.xlabel('RAF inhibitor ($\mu$M)')
plt.ylabel('% Active RAF')
plt.legend(prop={'size':12},loc='upper center', bbox_to_anchor=(0.5,1.
↪27),ncol=2)
plt.show()
```

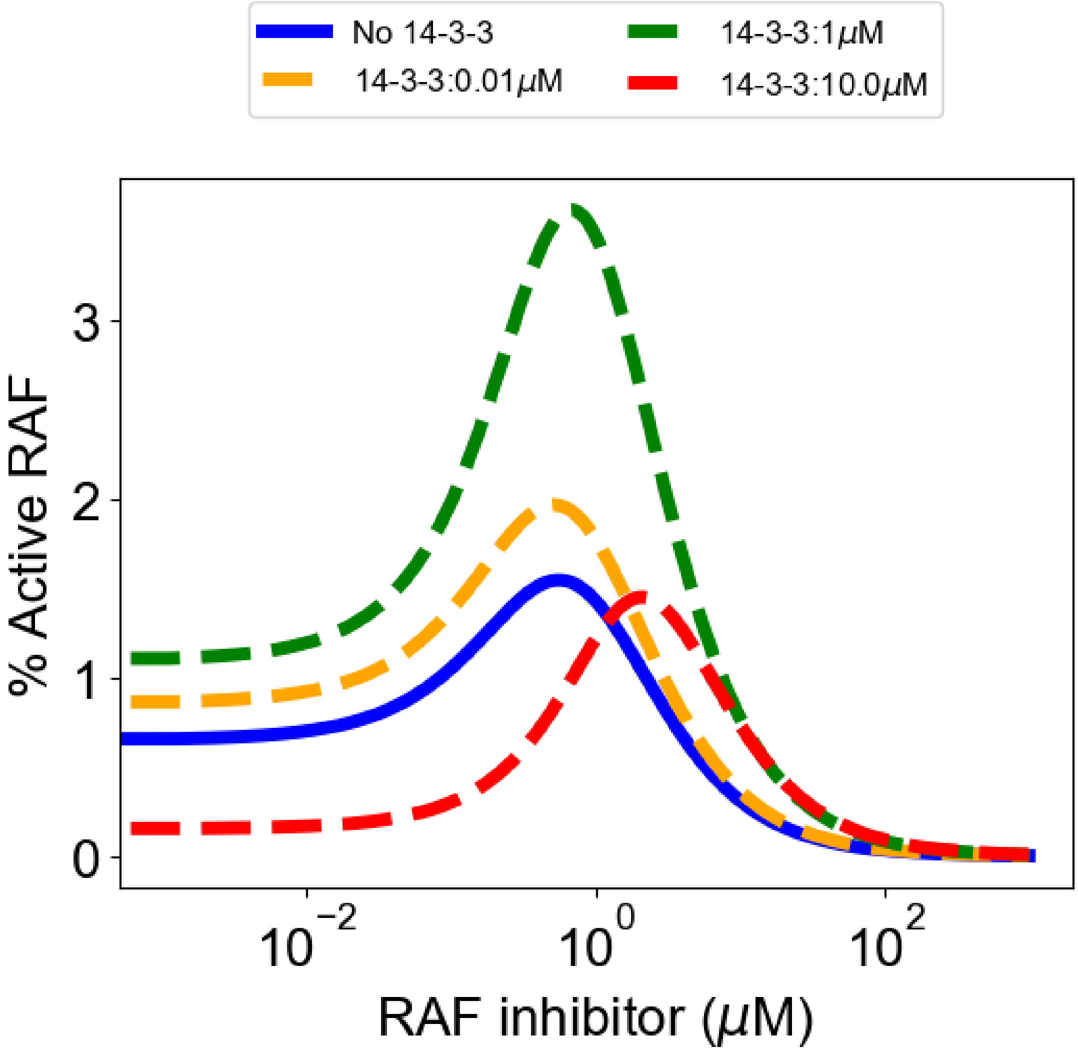


[36]:
```
font = {'family' : 'Arial',
        'size'   : 20}
plt.rc('font', **font)

paramsplt0=dict(params0) #units: micro-molar
xdatr=[0.]+list(10**np.linspace(-2,4,10000))

# STOTvalslist=[0.000001,0.01,1,10.0]
# STOTcolorlist=['b','orange','g','r']
# ls=['solid','dashed','dashed','dashed']
# labellist=[r'No 14-3-3','14-3-3:'+str(STOTvalslist[1])+'$\mu$M','14-3-3:
↪'+str(STOTvalslist[2])+'$\mu$M','14-3-3:'+str(STOTvalslist[3])+'$\mu$M']

for i in range(len(STOTvalslist)):
```

```
    paramsplt0['STOT']=STOTvalslist[i] #
    xdat=[dr2DTOT(elem,paramsplt0) for elem in xdatr]
    ydat= [actkin(xval,paramsplt0)/actkin(0.,paramsplt0) for xval in xdatr]
    plt.
 ↪plot(xdat,ydat,label=labellist[i],color=STOTcolorlist[i],linestyle=ls[i],linewidth=5)
plt.xscale('log')
plt.xlabel('RAF inhibitor ($\mu$M)')
plt.ylabel('Active RAF Normalized')
plt.legend(prop={'size':12},bbox_to_anchor=[1,0.5])
plt.show()
```

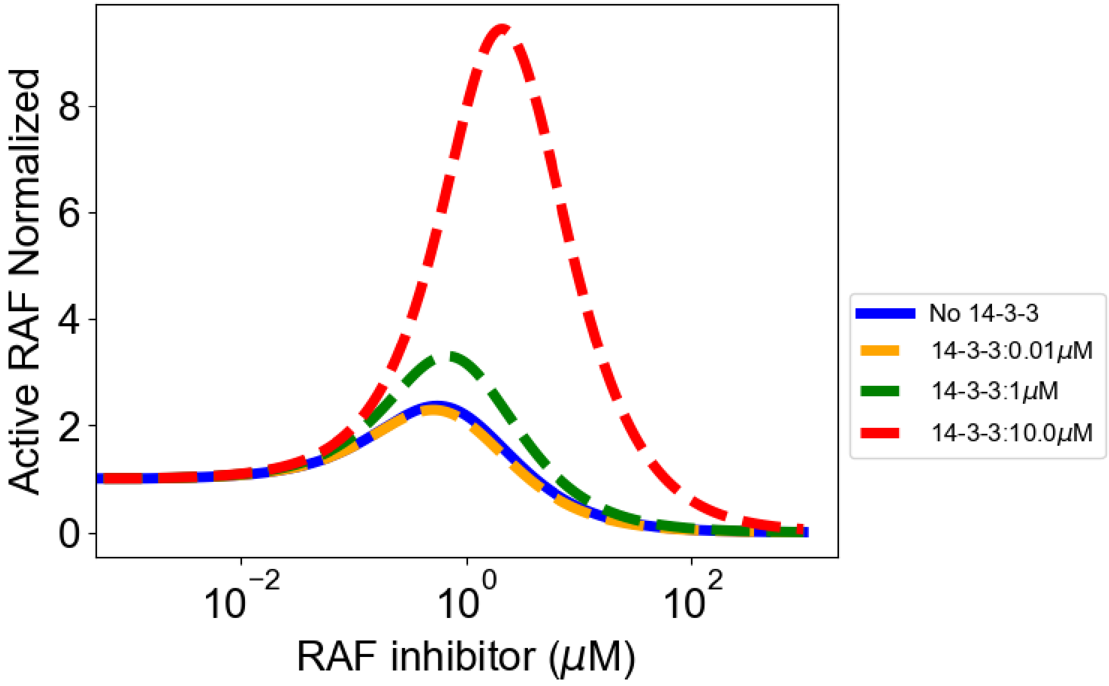


## 0.4 Figure S1B

[37]:
```
# Define total dimer functions
def DTOT2Dimers(DTOT,params):
    try:
        if checkpos(list(params.values())) is False:
            return 10**10
        elif DTOT>=DTOTMIN:
            dr=DTOT2dr(DTOT,params)
        else:
            dr=0.
        return actkin(dr,params)*(1+dr)/2.
    except:
```

```
        print("ERROR DTOT2Dimers:",DTOT,params)
def DTOT2DimersNorm(DTOT,params):
    """This function inputs total drug values (in uM or same units as Kd in␣
 ↪params), paramteres to output active RAF protomers normalized to no-drug"""
    return DTOT2Dimers(DTOT,params)*2./actkin(0,params)
```

[38]:
```
# Plot Total dimers as a function of the total Drug and with manipulating KSdim

font = {'family' : 'Arial',
        'size'   : 20}
plt.rc('font', **font)



paramsplt0=dict(params0) #units: micro-molar

xdatr=[0.]+list(10**np.linspace(-2,4,300))

KSdimvalslist=[1000000.,0.1,0.1] # very large KSdim = CAS model, medium KSdim,␣
 ↪very small KSdim=DS model
KSmonvalslist=[0.1,0.1,1000000]
KSdimcolorlist=['orange','r','k']
ls=['solid','dashed','solid']
labellist=['Conformal Autoinhibition Role','Both Roles','Dimer Stabilization']␣
 ↪#r'$\KSdim=$'+str(paramsplt0['KSdim'])
stotval=1.
paramsplt0['STOT']=stotval
for i in range(len(KSdimvalslist)):
    paramsplt0['KSdim']=KSdimvalslist[i]
    paramsplt0['KSmon']=KSmonvalslist[i]
    xdat=[dr2DTOT(elem,paramsplt0) for elem in xdatr]
    ydat= [DTOT2Dimers(xval,paramsplt0)*100 for xval in xdatr]
    plt.
 ↪plot(xdat,ydat,label=labellist[i],color=KSdimcolorlist[i],linestyle=ls[i],linewidth=5)

paramsplt0['STOT']=0.000001 # STOT limits 0
paramsplt0['KSdim']=10**10 # dissociation infinity is same as no reaction
paramsplt0['KSmon']=10**10 # dissociation infinity is same as no reaction
xdat=[dr2DTOT(elem,paramsplt0) for elem in xdatr]
ydat= [DTOT2Dimers(xval,paramsplt0)*100 for xval in xdat] # solution with no␣
 ↪14-3-3
plt.plot(xdat,ydat,label='No 14-3-3',color='b',linewidth=5)

plt.yscale('log')
plt.xscale('log')
plt.xlabel('RAF inhibitor ($\mu$M)')
plt.ylabel('% RAF Dimers')
plt.legend(prop={'size':12},bbox_to_anchor=[1.7,0.5])
```

```
plt.show()

paramsplt0=dict(params0) #units: micro-molar
paramsplt0['STOT']=stotval
for i in range(len(KSdimvalslist)):
    paramsplt0['KSdim']=KSdimvalslist[i] #
    paramsplt0['KSmon']=KSmonvalslist[i]
    xdat=[dr2DTOT(elem,paramsplt0) for elem in xdatr]
    ydat= [DTOT2Dimers(xval,paramsplt0)/DTOT2Dimers(0.,paramsplt0) for xval in␣
 ↪xdatr]
    plt.
 ↪plot(xdat,ydat,label=labellist[i],color=KSdimcolorlist[i],linestyle=ls[i],linewidth=5)

paramsplt0['STOT']=0.00001
paramsplt0['KSdim']=10**10 # dissociation infinity is same as no reaction
paramsplt0['KSmon']=10**10 # dissociation infinity is same as no reaction
xdat=[dr2DTOT(elem,paramsplt0) for elem in xdatr]
ydat= [DTOT2Dimers(xval,paramsplt0)/DTOT2Dimers(0.,paramsplt0) for xval in␣
 ↪xdatr] # solution with no 14-3-3
plt.plot(xdat,ydat,label='No 14-3-3',color='b',linewidth=5)


plt.xscale('log')
plt.yscale('log')
plt.xlabel('RAF inhibitor ($\mu$M)')
plt.ylabel('RAF Dimers Normalized')
plt.legend(prop={'size':12},bbox_to_anchor=[1,0.5])
plt.show()
```

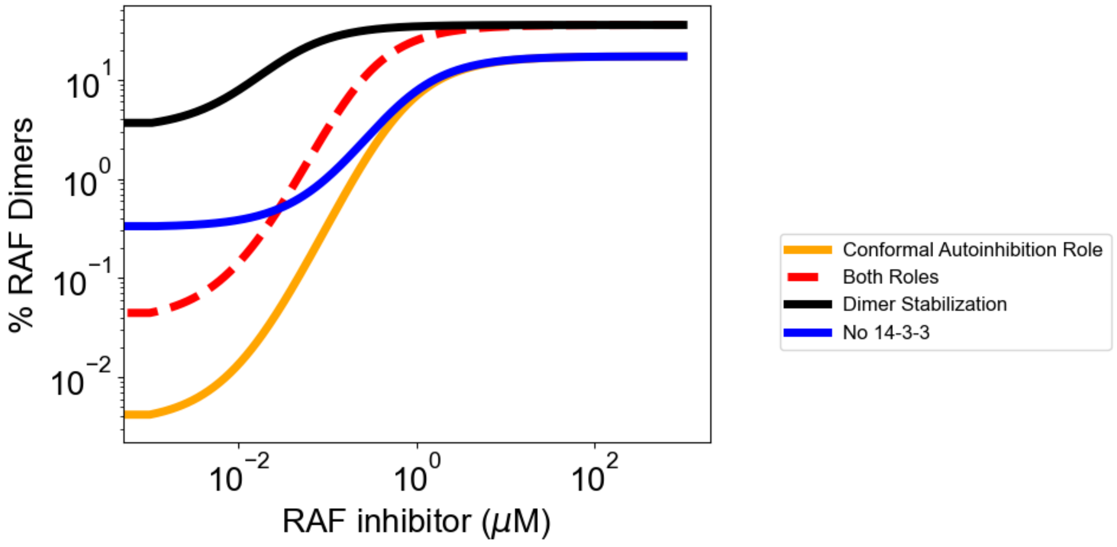

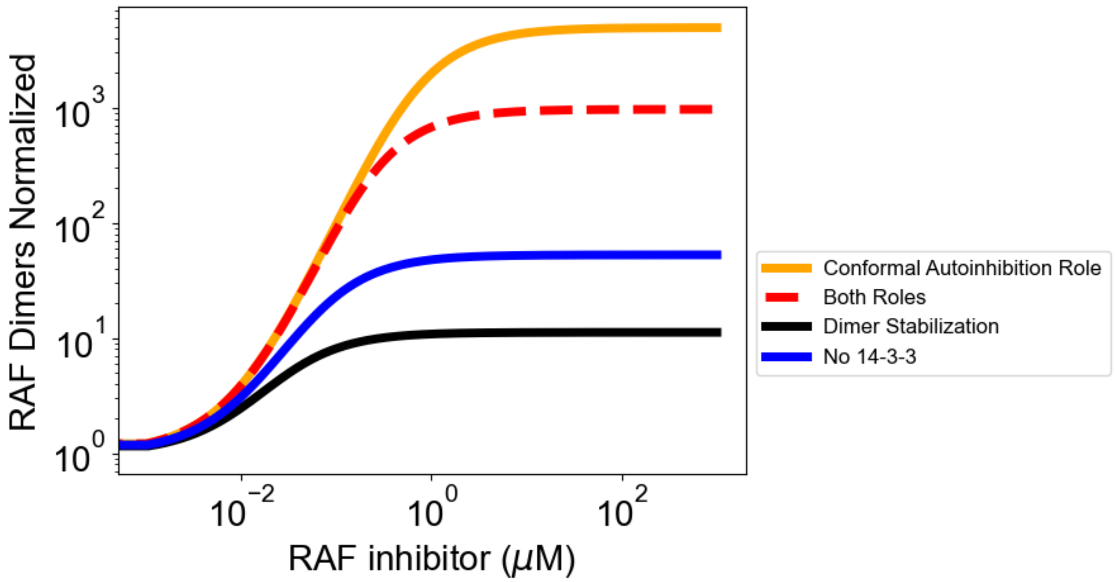


## 0.5 Figure 2C

[39]:
```
%%time
paramsbase=dict(params0)# values in micro-molar
npts=10000 # total number of points to plot
npts=int(np.sqrt(npts)) # square root of the number of points to put on a␣
↪square grid
xlist = np.linspace(-1.0, 2.0,npts)
ylist = np.linspace(-3.0, 2.0,npts)
X, Y = np.meshgrid(xlist, ylist)
def callfnr(x1,y1):
    params1=dict(paramsbase)
    params1['RAFTOT']=10**y1
    params1['KA']=10**x1
#     params1['rafr']=params1['RAFTOT']/params1['Kdim']
#     params1['sr']=params1['STOT']/params1['KS']
    try:
        resarr=solrange(params1)
        if resarr[0]>10**-5:
            return [np.log10(resarr[1]),np.log10(resarr[2])]
        else:
            return [float('nan'),float('nan')]
    except:
        return [float('nan'),float('nan')]
zfc=[]
zr=[]
for itr in range(len(X)):
```

```
    zfc=zfc+[[]]
    zr=zr+[[]]
    for jtr in range(len(X[itr])):
        res=callfnr(X[itr][jtr],Y[itr][jtr])
        zfc[itr]=zfc[itr]+[res[0]]
        zr[itr]=zr[itr]+[res[1]]
```

```
ERROR actkin: 140546.9104593961 {'KA': 100.0, 'Kd': 0.1, 'Kdim': 0.1, 'RAFTOT':
0.37649358067924676, 'KSmon': 0.2, 'KSdim': 0.02, 'STOT': 1.0}
ERROR actkin: 17.307127153916923 {'KA': 0.30538555088334157, 'Kd': 0.1, 'Kdim':
0.1, 'RAFTOT': 0.4229242874389499, 'KSmon': 0.2, 'KSdim': 0.02, 'STOT': 1.0}

C:\Users\GM\AppData\Local\Temp\ipykernel_15708\207397477.py:82: RuntimeWarning:
divide by zero encountered in scalar divide
  objfn=lambda dr:kinref/actkin(dr,params)

ERROR: d2DTOT:  0.38618455256250833 {'KA': 5.3366992312063095, 'Kd': 0.1,
'Kdim': 0.1, 'RAFTOT': 0.4229242874389499, 'KSmon': 0.2, 'KSdim': 0.02, 'STOT':
1.0}
ERROR DTOT2d_2: 0.08529679095122686 {'KA': 5.3366992312063095, 'Kd': 0.1,
'Kdim': 0.1, 'RAFTOT': 0.4229242874389499, 'KSmon': 0.2, 'KSdim': 0.02, 'STOT':
1.0}
ERROR actkin: None {'KA': 5.3366992312063095, 'Kd': 0.1, 'Kdim': 0.1, 'RAFTOT':
0.4229242874389499, 'KSmon': 0.2, 'KSdim': 0.02, 'STOT': 1.0}
CPU times: total: 1min 6s
Wall time: 1min 59s
```

[ ]:
```
font = {'family' : 'Arial',
        'size'   : 35}
plt.rc('font', **font)
plt.figure(figsize=(7,6))
colormaptype='viridis'
fileid="range"
minlevel=-1
levels=[minlevel]+[i/10 for i in range(0,60,10)]
cp = plt.contourf(X,Y,zr,levels=levels,cmap=colormaptype)
plt.contour(cp,colors='k',linewidths=1.5)
plt.colorbar(cp)
minlevel=0.0045
lc=plt.contour(X,Y,zfc,colors='r',linewidths=7.
 ↪,levels=[minlevel],linestyles='solid')
plt.xlabel('Log10(KA)')
plt.ylabel(r'Log10(RAF ($\mu$M))')
# figname="DAK_1433full_DTOT_"+fileid+"_RAFrvsKA.pdf"
# plt.savefig(figname,dpi=300)
plt.show()
```

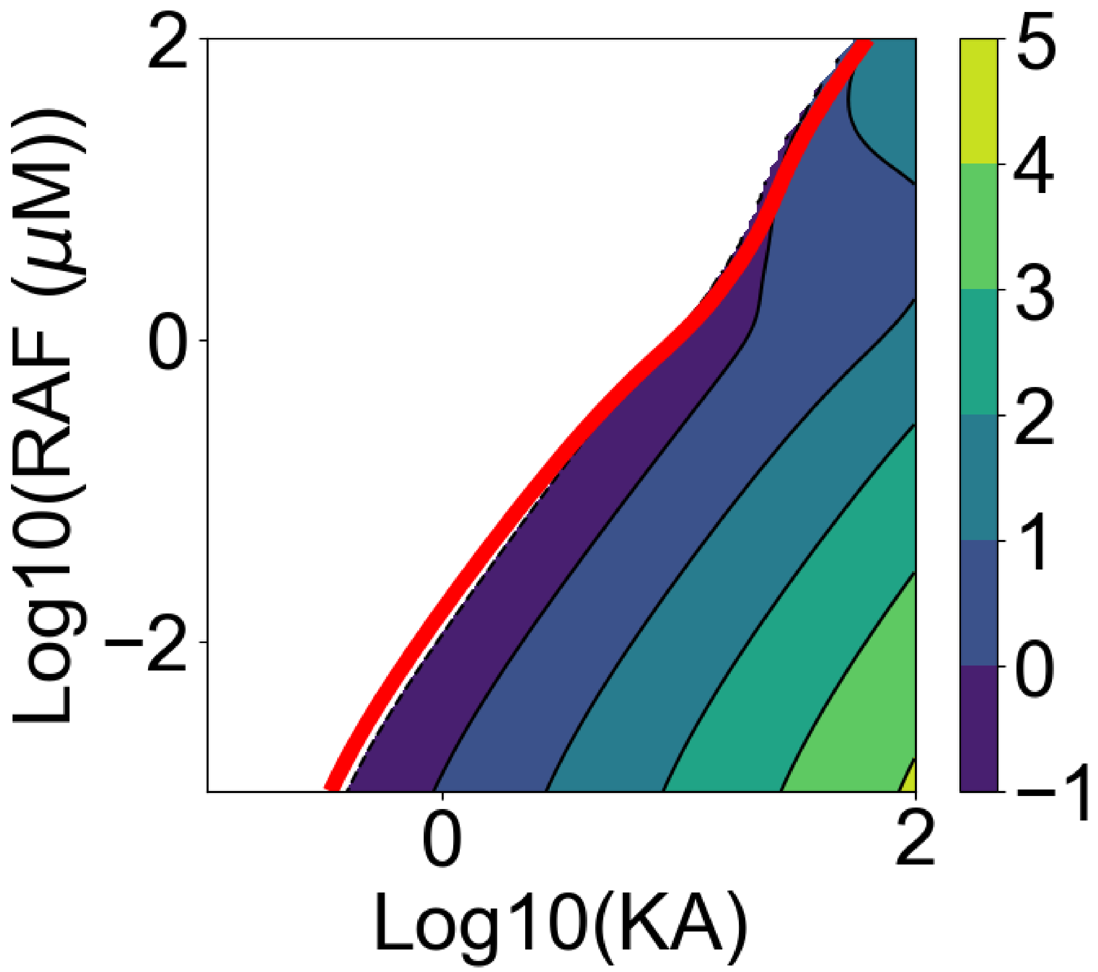


### 0.6 Figure S1

```
[ ]: colormaptype='gnuplot'
fileid="foldchange"
plt.figure(figsize=(7,6))
minlevel=0.03
levels=[minlevel]+[i/10 for i in range(5,26,5)]
cp = plt.contourf(X,Y,zfc,levels=levels,cmap=colormaptype)
plt.contour(cp,colors='k',linewidths=1.5)
plt.colorbar(cp)
lc=plt.contour(cp,colors='r',linewidths=4.,levels=[minlevel],linestyles='solid')
plt.xlabel('Log10(KA)')
plt.ylabel('Log10(Total RAF [uM])')
# figname="DAK_1433full_"+fileid+"_RAFrvsKA.pdf"
# plt.savefig(figname,dpi=300)
plt.show()
```

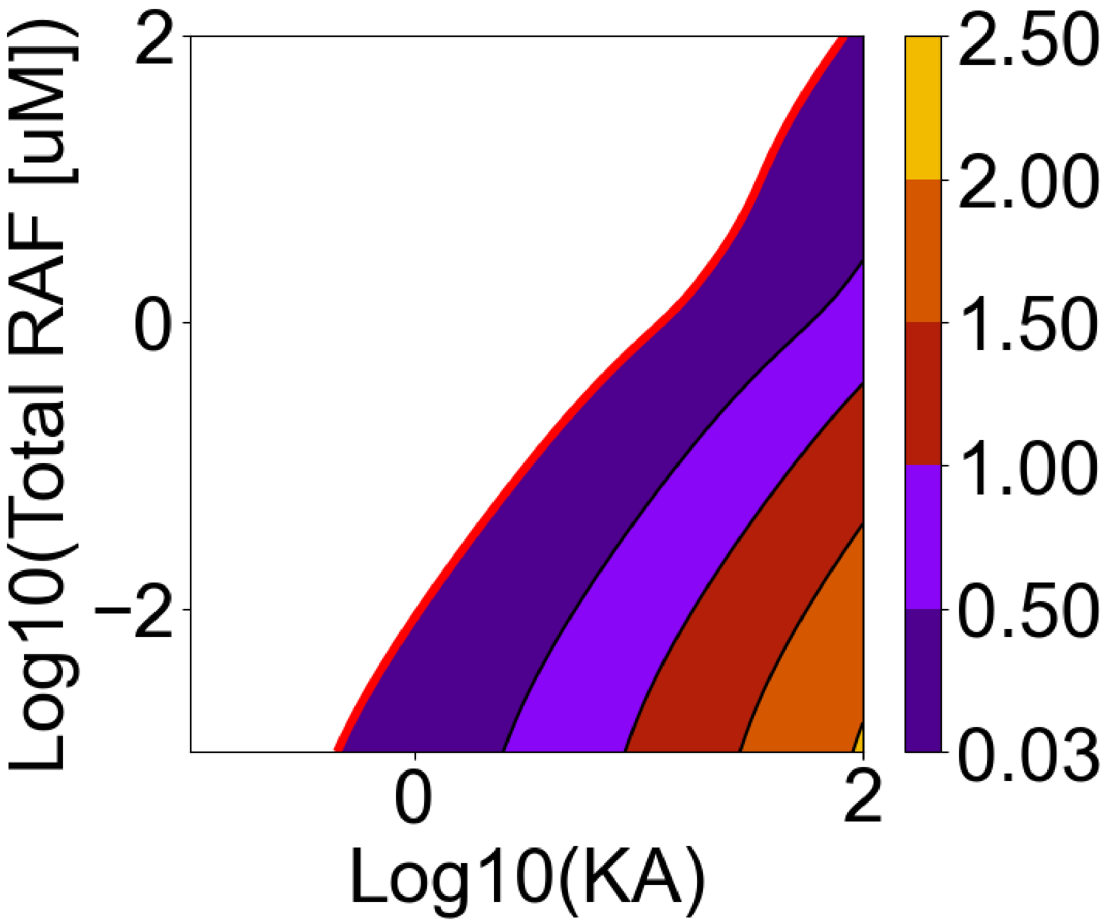


### 0.7 Figure 2C

[7]:
```
paramsbase=dict(params0)# values in micro-molar
npts=10000 # total number of points to plot
npts=int(np.sqrt(npts)) # square root of the number of points to put on a
  ↪square grid
xlist = np.linspace(-1.0, 2.0,npts)
ylist = np.linspace(-3.0, 2.0,npts)
X, Y = np.meshgrid(xlist, ylist)
def callfnr(x1,y1):
    params1=dict(paramsbase)
    params1['STOT']=10**y1
    params1['KA']=10**x1
#     params1['rafr']=params1['RAFTOT']/params1['Kdim']
#     params1['sr']=params1['STOT']/params1['KS']
    try:
        resarr=solrange(params1)
        if resarr[0]>10**-5:
```

```
            return [np.log10(resarr[1]),np.log10(resarr[2])]
        else:
            return [float('nan'),float('nan')]
    except:
        return [float('nan'),float('nan')]
zfc=[]
zr=[]
for itr in range(len(X)):
    zfc=zfc+[[]]
    zr=zr+[[]]
    for jtr in range(len(X[itr])):
        res=callfnr(X[itr][jtr],Y[itr][jtr])
        zfc[itr]=zfc[itr]+[res[0]]
        zr[itr]=zr[itr]+[res[1]]
```

[ ]:

```
font = {'family' : 'Arial',
        'size'   : 35}
plt.rc('font', **font)
plt.figure(figsize=(7,6))
colormaptype='viridis'
fileid="range"
minlevel=-1.5
levels=[minlevel]+[i/10 for i in range(0,91,15)]
cp = plt.contourf(X,Y,zr,levels=levels,cmap=colormaptype)
plt.contour(cp,colors='k',linewidths=1.5)
plt.colorbar(cp)
minlevel=0.005
lc=plt.contour(X,Y,zfc,colors='r',linewidths=5.
 ↪,levels=[minlevel],linestyles='solid')
plt.xlabel('Log10(KA)')
plt.ylabel('Log10([14-3-3] (uM))')
# plt.savefig(figname,dpi=300)
plt.show()
```

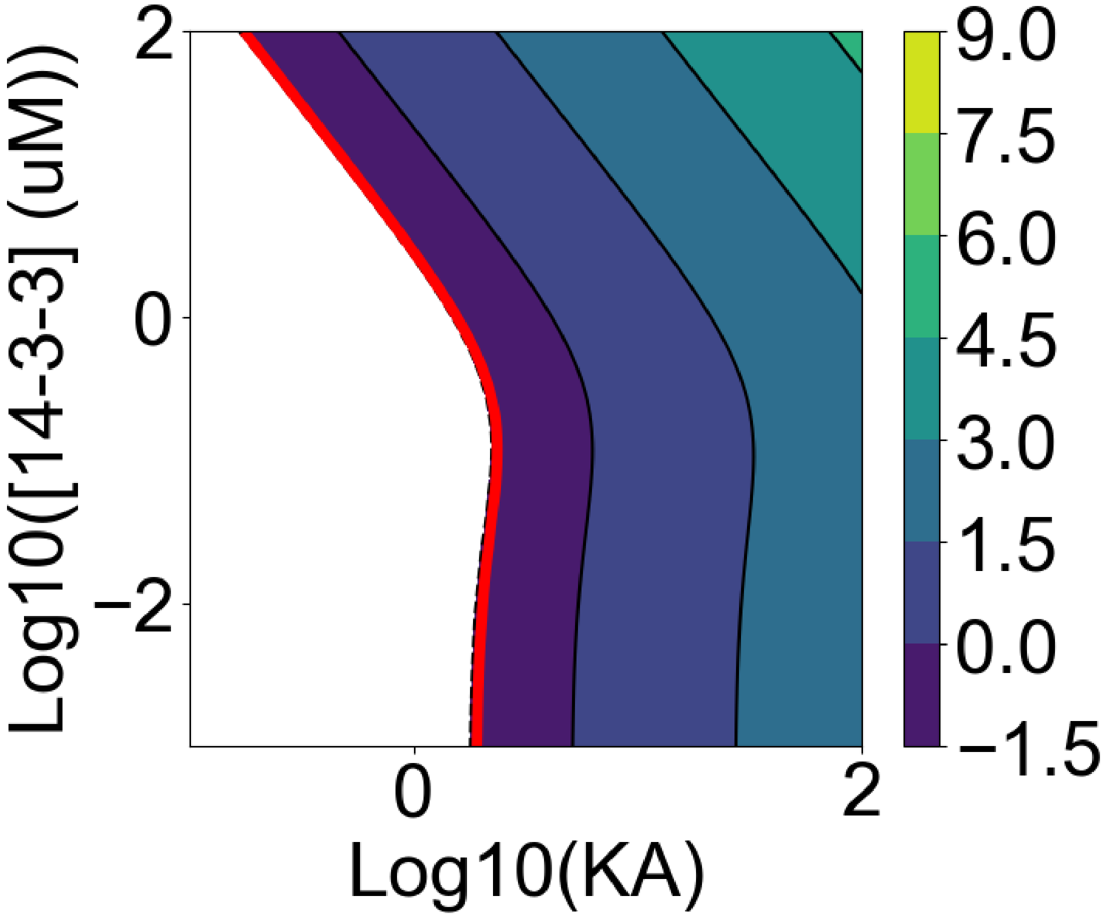


#### 0.8 Figure S1H

[12]:
```
colormaptype='gnuplot'
fileid="foldchange"
plt.figure(figsize=(7,6))
minlevel=0.0
levels=[minlevel]+[i/10 for i in range(10,41,5)]
cp = plt.contourf(X,Y,zfc,levels=levels,cmap=colormaptype)
plt.contour(cp,colors='k',linewidths=1.5)
plt.colorbar(cp)
minlevel=0.001
lc=plt.contour(cp,colors='r',linewidths=4.,levels=[minlevel],linestyles='solid')
# cp2 = plt.contourf(X1,Y1,Z1,levels)
plt.xlabel('Log10(KA)')
plt.ylabel('Log10([14-3-3] (uM))')
figname="DAK_stableAI_DTOT_"+fileid+"_STOTvsKA.pdf"
# plt.savefig(figname,dpi=300)
plt.show()
```

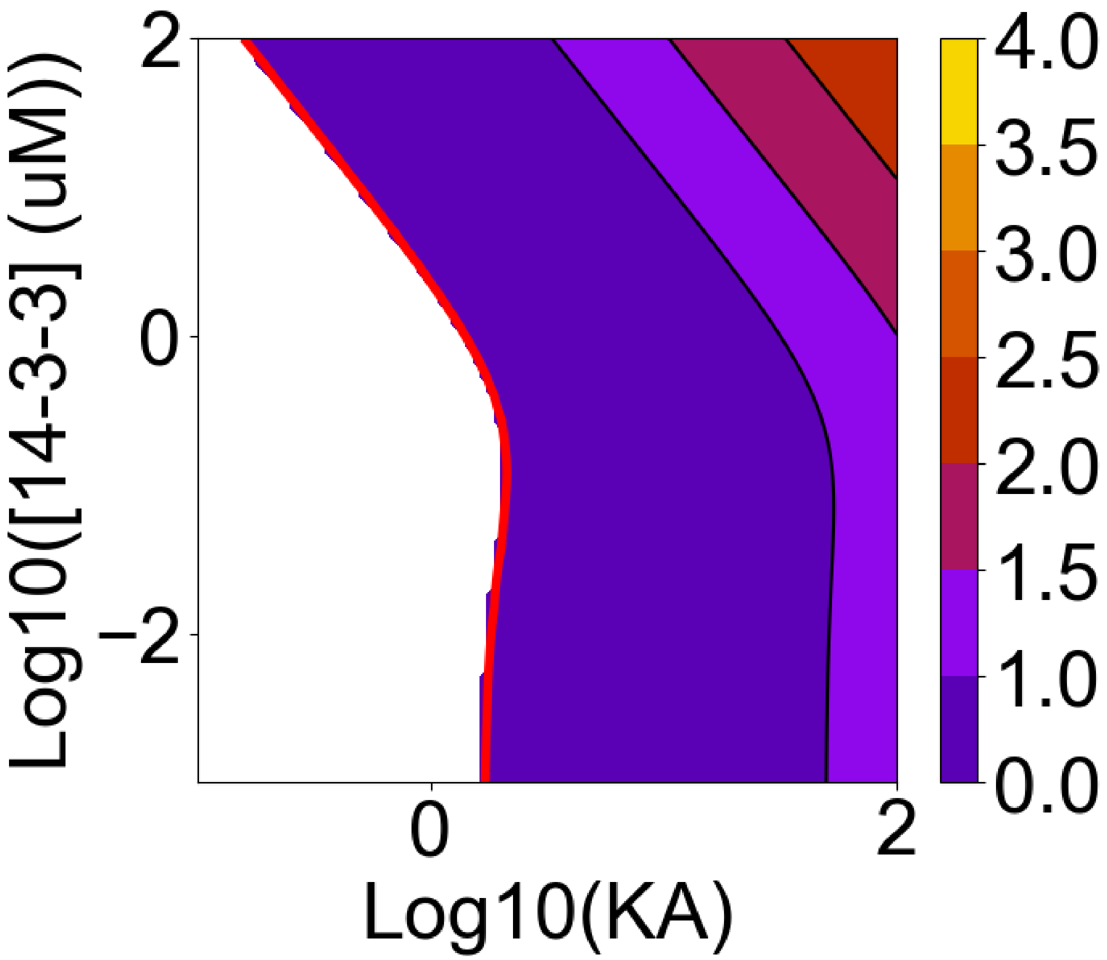

### 0.8.1 Additional figures describing DS model limit

[13]:
```
# make an example plot for 14-3-3 model variations for only DS model
font = {'family' : 'Arial',
        'size'   : 20}
plt.rc('font', **font)



paramsplt0=dict(params0) #units: micro-molar
paramsplt0['STOT']=0.000001
xdatr=[0.]+list(10**np.linspace(-2,4,300))
xdat=[dr2DTOT_DS(elem,paramsplt0) for elem in xdatr]
ydat= [actkin_DS(xval,paramsplt0)/actkin_DS(0,paramsplt0) for xval in xdatr] #␣
 ↪solution with no 14-3-3
plt.plot(xdat,ydat,label='No 14-3-3',color='b')

paramsplt0['STOT']=1.
paramsplt0['KSdim']=0.2
xdat=[dr2DTOT_DS(elem,paramsplt0) for elem in xdatr]
ydat= [actkin_DS(xval,paramsplt0)/actkin_DS(0,paramsplt0) for xval in xdatr] #␣
 ↪solution with no Auto-inhibition stabilization, only KSdim
plt.plot(xdat,ydat,label=r'Dimer Stab. KSdim=0.2',color='orange')

paramsplt0['KSdim']=1.
xdat=[dr2DTOT_DS(elem,paramsplt0) for elem in xdatr]
ydat= [actkin_DS(xval,paramsplt0)/actkin_DS(0,paramsplt0) for xval in xdatr] #␣
 ↪solution with no Auto-inhibition stabilization, only KSdim
plt.plot(xdat,ydat,label=r'Dimer Stab. KSdim=1',color='g')

paramsplt0['KSdim']=5.
xdat=[dr2DTOT_DS(elem,paramsplt0) for elem in xdatr]
ydat= [actkin_DS(xval,paramsplt0)/actkin_DS(0,paramsplt0) for xval in xdatr] #␣
 ↪solution with no Auto-inhibition stabilization, only KSdim
plt.plot(xdat,ydat,label=r'Dimer Stab. KSdim=5',color='r')

plt.xscale('log')
plt.xlabel('Relative Drug Conc')
plt.ylabel('Active RAF Normalized')
plt.legend(prop={'size':12})
plt.show()

paramsplt0=dict(params0) #units: micro-molar
# make an example plot for 14-3-3 model variations with RAF un-normalized
paramsplt0['STOT']=0.000001
xdatr=[0.]+list(10**np.linspace(-2,4,300))
xdat=[dr2DTOT_DS(elem,paramsplt0) for elem in xdatr]
```

```
ydat= [actkin_DS(xval,paramsplt0)*100 for xval in xdatr] # solution with no␣
↪14-3-3
plt.plot(xdat,ydat,label='No 14-3-3. (KA=10)',color='b')

paramsplt0['STOT']=1.
paramsplt0['KSdim']=0.2
xdat=[dr2DTOT_DS(elem,paramsplt0) for elem in xdatr]
ydat= [actkin_DS(xval,paramsplt0)*100 for xval in xdatr] # solution with no␣
↪Auto-inhibition stabilization, only KSdim
plt.plot(xdat,ydat,label=r'Dimer Stab. KSdim=0.2',color='orange')

paramsplt0['KSdim']=1.
xdat=[dr2DTOT_DS(elem,paramsplt0) for elem in xdatr]
ydat= [actkin_DS(xval,paramsplt0)*100 for xval in xdatr] # solution with no␣
↪Auto-inhibition stabilization, only KSdim
plt.plot(xdat,ydat,label=r'Dimer Stab. KSdim=1',color='g')

paramsplt0['KSdim']=5.
xdat=[dr2DTOT_DS(elem,paramsplt0) for elem in xdatr]
ydat= [actkin_DS(xval,paramsplt0)*100 for xval in xdatr] # solution with no␣
↪Auto-inhibition stabilization, only KSdim
plt.plot(xdat,ydat,label=r'Dimer Stab. KSdim=5',color='r')



plt.xscale('log')
plt.xlabel('Drug Conc')
plt.ylabel('Active RAF Percent')
plt.legend(prop={'size':12})
plt.show()
```

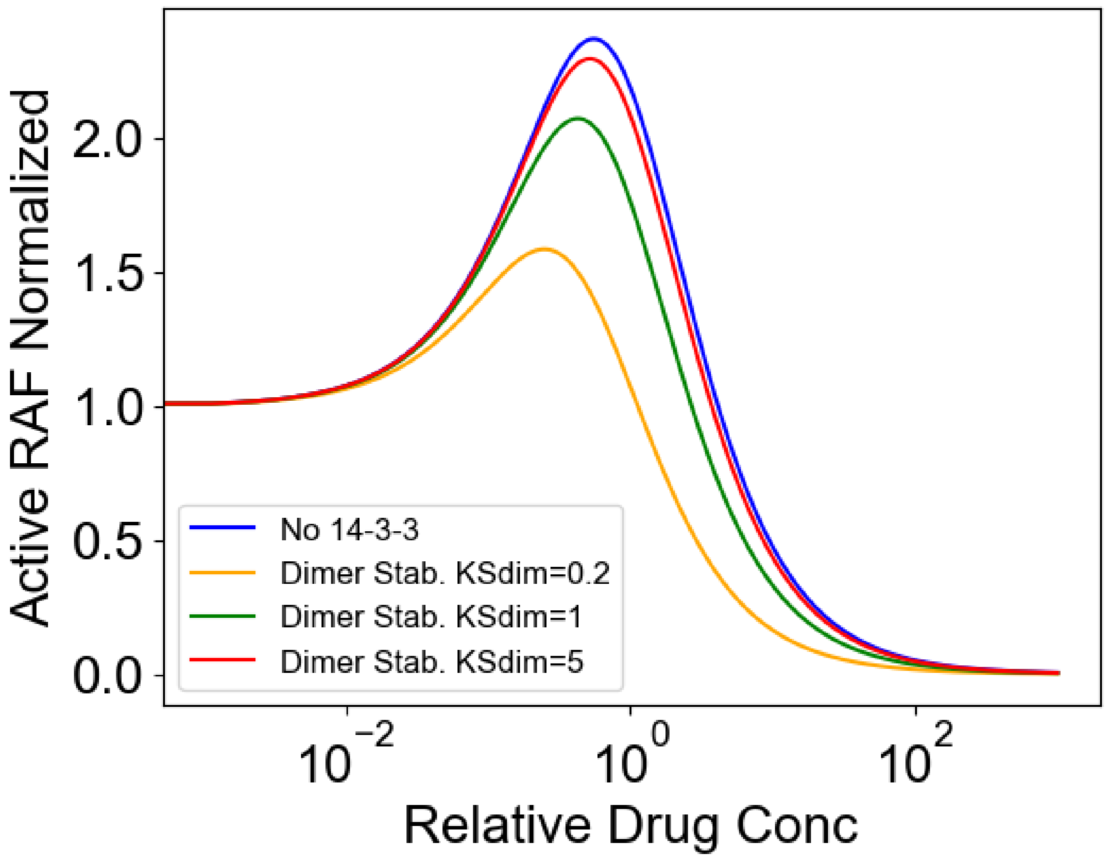

Active RAF Normalized
2.0
1.5
1.0
0.5
0.0
No 14-3-3
Dimer Stab. KSdim=0.2
Dimer Stab. KSdim=1
Dimer Stab. KSdim=5
$10^{-2}$
$10^{0}$
$10^{2}$
Relative Drug Conc

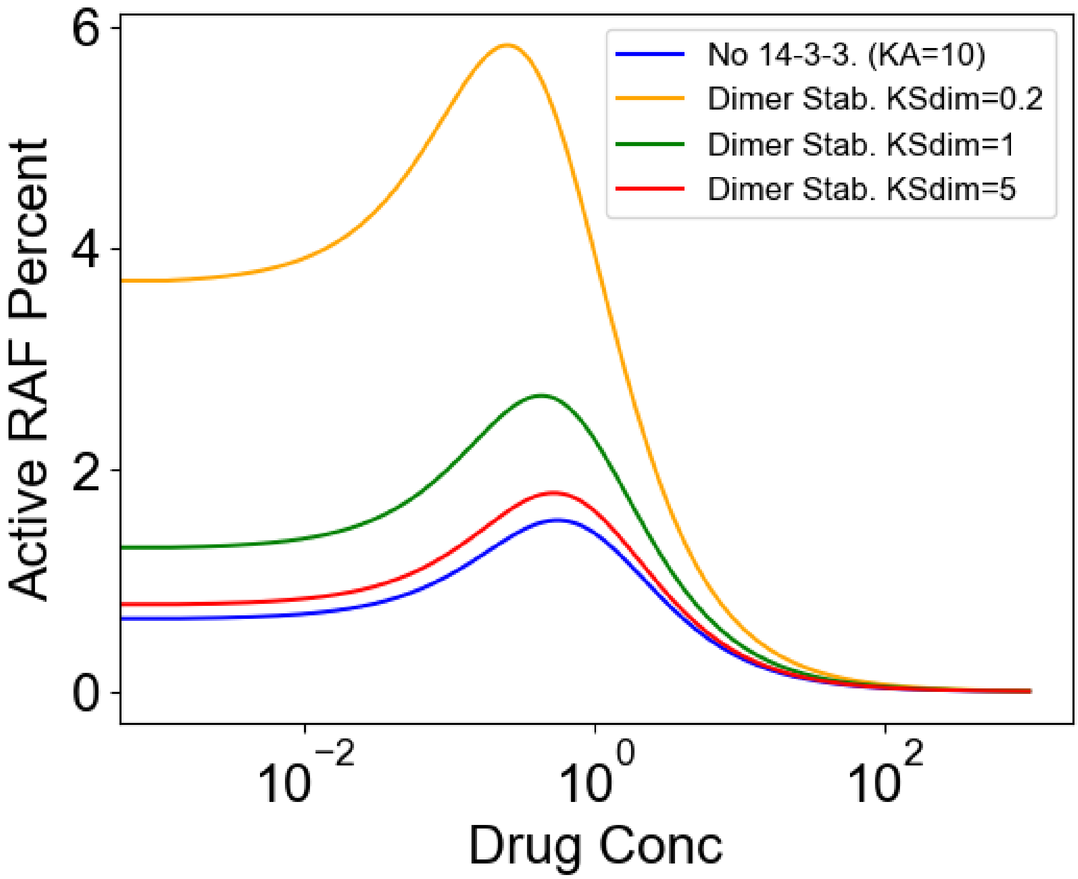


### 0.9 Additional Figures: Baseline Signaling

In figures below, large alpha implies CAS role dominant. Small alpha is DS role dominant. However, both mechanisms are present in all parameter regions shown.

[14]:
```
%%time
# Contour plot of actkin at d=0 with alpha and STOT varied.
paramsbase=dict(params0)# values in micro-molar
npts=10000 # total number of points to plot
npts=int(np.sqrt(npts)) # square root of the number of points to put on a␣
↪square grid
xlist = np.linspace(-3.0,2.0,npts)
ylist = np.linspace(-2.0,2.0,npts)
X, Y = np.meshgrid(xlist, ylist)
def callfnr(x1,y1):
    params2=dict(paramsbase)
    params2['STOT']=10**x1
    alpha=10**y1
```

```
    # parameterize using a single parameter to vary regions from DS limit to␣
 ↪CAS limit when alpha is varied from 0.01 to 100.
    params2['KSmon']=params2['KSmon']/alpha
    params2['KSdim']=params2['KSdim']*alpha
    try:
        resarr=actkin(0.0,params2)*100
        return np.log10(resarr)
    except:
        return float('nan')
zbl=[]
for itr in range(len(X)):
    zbl=zbl+[[]]
    for jtr in range(len(X[itr])):
        res=callfnr(X[itr][jtr],Y[itr][jtr])
        zbl[itr]=zbl[itr]+[res]
```

```
CPU times: total: 375 ms
Wall time: 767 ms
```

[15]:
```
max([ival for irow in zbl for ival in irow]) # % RAF can only be max 100%
```

[15]: 1.9677993736

## 0.10 Figure S2A

[16]:
```
# Log10(% RAF without drug)
font = {'family' : 'Arial',
        'size'   : 30}
plt.rc('font', **font)
plt.figure(figsize=(7,6))
colormaptype='brg'
fileid="BaselineSignaling"
cp = plt.contourf(X,Y,zbl,cmap=colormaptype)
plt.contour(cp,colors='k',linewidths=1.5)
plt.colorbar(cp,label='log10(% Active RAF w/o drug)')
# level1=0.5
# lc=plt.contour(X,Y,zbl,colors='r',linewidths=5.,linestyles='solid')

# plt.plot([np.log10(paramsbase['KSmon']/paramsbase['KA'])]*len(Y[:,0]),Y[:
 ↪,0],'b-',label='[14-3-3]<KS/KA',linewidth=3)
# plt.plot(X[0,:],[np.log10(np.sqrt(2))]*len(X[0,:]),'b--',label='KSm>2xKSd␣
 ↪(alpha<sqrt(2)): increasing',linewidth=3)

# plt.legend(bbox_to_anchor=(1.5,0.5),title='Monotonocity above these lines')

plt.ylabel(r'log10($\alpha$)')
plt.xlabel('Log10([14-3-3] (uM))')
```

```
figname="DAK_1433_"+fileid+"_STOTvsAlpha.pdf"
# plt.savefig(figname,dpi=300)
plt.show()
```

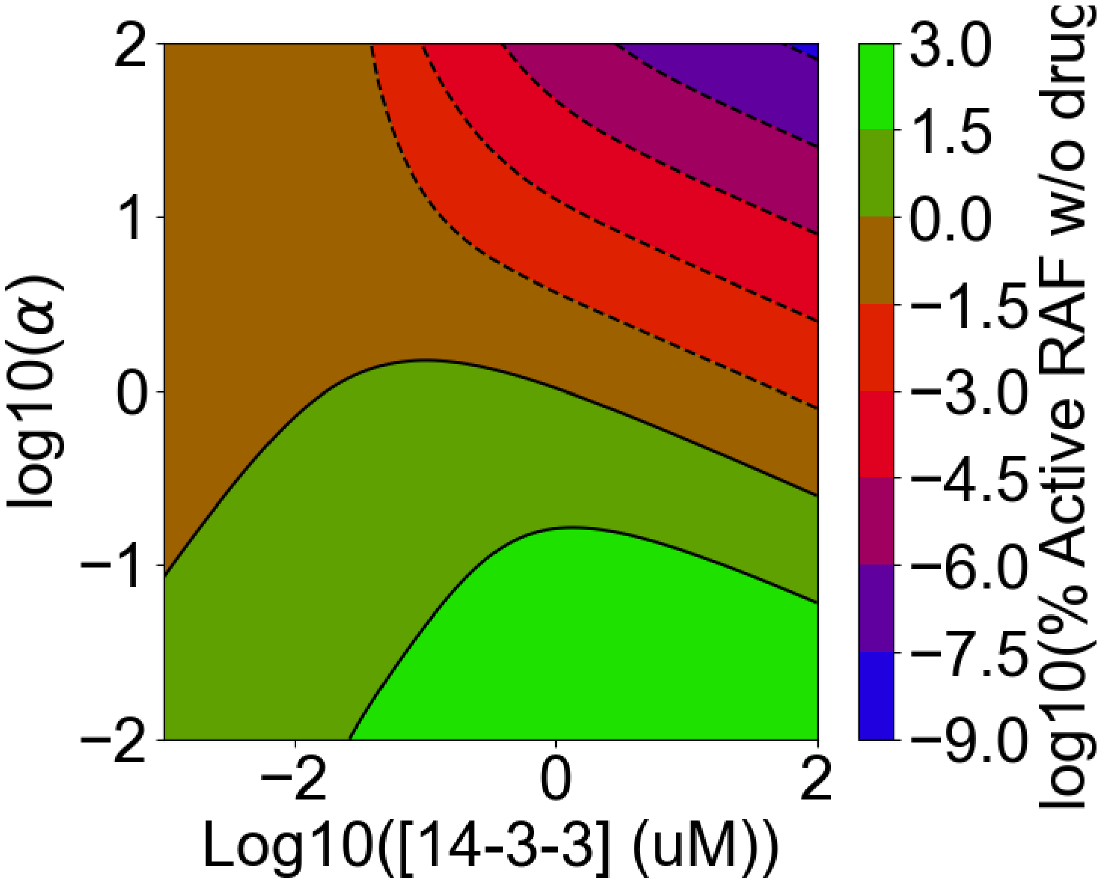


[42]:
```
%%time
# Contour plot of actkin at d=0 with alpha and STOT varied.
paramsbase=dict(params0)# values in micro-molar
paramsbase.pop('STOT')
npts=10000 # total number of points to plot
npts=int(np.sqrt(npts)) # square root of the number of points to put on a␣
↪square grid
xlist = np.linspace(-3.0,2.0,npts)
ylist = np.linspace(-2.0,2.0,npts)
X, Y = np.meshgrid(xlist, ylist)
def callfnr(x1,y1):
    """Inputs Values of STOT and alpha and outputs the [% peak activation RAF,␣
↪Peak fold change,activating range] on a log10 basis."""
    params1=dict(paramsbase)
    params1['STOT']=10**x1
```

```
    alpha=10**y1
    # parameterize using a single parameter to vary regions from DS limit to␣
↪CAS limit when alpha is varied from 0.01 to 100.
    params1['KSmon']=params1['KSmon']/alpha
    params1['KSdim']=params1['KSdim']*alpha
    try:
        resarr=solrange(params1)
        dr=DTOT2dr(resarr[0],params1)
        if resarr[0]>10**-6:
            return [np.log10(actkin(dr,params1)*100),np.log10(resarr[1]),np.
↪log10(resarr[2])]
        elif resarr[0] == 0:
            return [np.log10(actkin(dr,params1)*100),0.,0.]
        else:
            return [float('nan'),float('nan'),float('nan')]
    except:
        return [float('nan'),float('nan'),float('nan')]
zfc=[]
zr=[]
zpa=[]
for itr in range(len(X)):
    zfc=zfc+[[]]
    zr=zr+[[]]
    zpa=zpa+[[]]
    for jtr in range(len(X[itr])):
        res=callfnr(X[itr][jtr],Y[itr][jtr])
        zfc[itr]=zfc[itr]+[res[1]]
        zr[itr]=zr[itr]+[res[2]]
        zpa[itr]=zpa[itr]+[res[0]]
```

```
CPU times: total: 1min 17s
Wall time: 2min 20s
```

[43]:
```
max([ival for irow in zpa for ival in irow]) # % RAF can only be max 100%
```

[43]: 1.5199402717

## 0.11 Figure S2B

[ ]:
```
# Log10(% RAF without drug)
font = {'family' : 'Arial',
        'size'   : 30}
plt.rc('font', **font)
plt.figure(figsize=(7,6))
colormaptype='brg'
fileid="%Activation"
levels=[i/10 for i in range(-40,21,5)]
```

```
cp = plt.contourf(X,Y,zpa,cmap=colormaptype,levels=levels)
plt.contour(cp,colors='k',linewidths=1.5)
plt.colorbar(cp,label='log10(% Peak Active RAF)')

# plt.plot(X[0],[np.log10(0.5)]*len(X[0]),'k-',label=r'$\alpha$=0.5')
# plt.plot([np.log10(paramsbase['KS']/paramsbase['KA'])]*len(Y[:,0]),Y[:
↪,0],'b--',label='[14-3-3]=KS/KA',linewidth=3)
# plt.plot([np.log10(paramsbase['KS'])]*len(Y[:,0]),Y[:,0],'b-.
↪',label='[14-3-3]=KS',linewidth=3)

# plt.legend(bbox_to_anchor=(1.5,0.5))

plt.ylabel(r'log10($\alpha$)')
plt.xlabel('Log10([14-3-3] (uM))')
figname="DAK_1433_"+fileid+"_STOTvsAlpha.pdf"
# plt.savefig(figname,dpi=300)
plt.show()
```

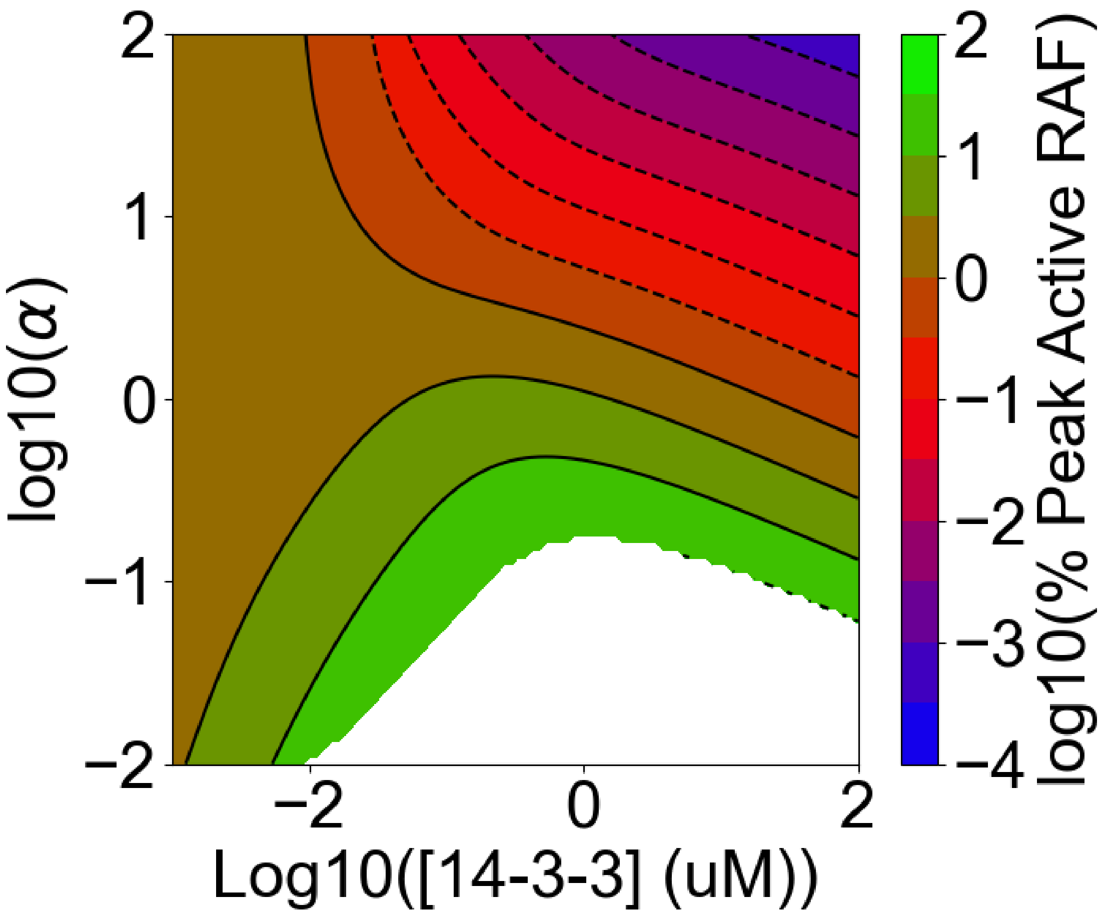

## 0.12 Figure S2C

[ ]:

```

```

[26]:

```
# Log10(% RAF without drug)
font = {'family' : 'Arial',
        'size'   : 30}
plt.rc('font', **font)
plt.figure(figsize=(7,6))
colormaptype='gnuplot'
fileid="%Activation"
# levels=[i/20 for i in range(-50,41,5)]
cp = plt.contourf(X,Y,zfc,cmap=colormaptype)
plt.contour(cp,colors='k',linewidths=1.5)
plt.colorbar(cp,label='log10(Fold Change)')

# plt.plot(X[0],[np.log10(0.5)]*len(X[0]),'k-',label=r'$\alpha$=0.5')
# plt.plot([np.log10(paramsbase['KS']/paramsbase['KA'])]*len(Y[:,0]),Y[:
 ↪,0],'b--',label='[14-3-3]=KS/KA',linewidth=3)
# plt.plot([np.log10(paramsbase['KS'])]*len(Y[:,0]),Y[:,0],'b-.
 ↪',label='[14-3-3]=KS',linewidth=3)

# plt.legend(bbox_to_anchor=(1.5,0.5))

plt.ylabel(r'log10($\alpha$)')
plt.xlabel('Log10([14-3-3] (uM))')
figname="DAK_1433_"+fileid+"_STOTvsAlpha.pdf"
# plt.savefig(figname,dpi=300)
plt.show()
```

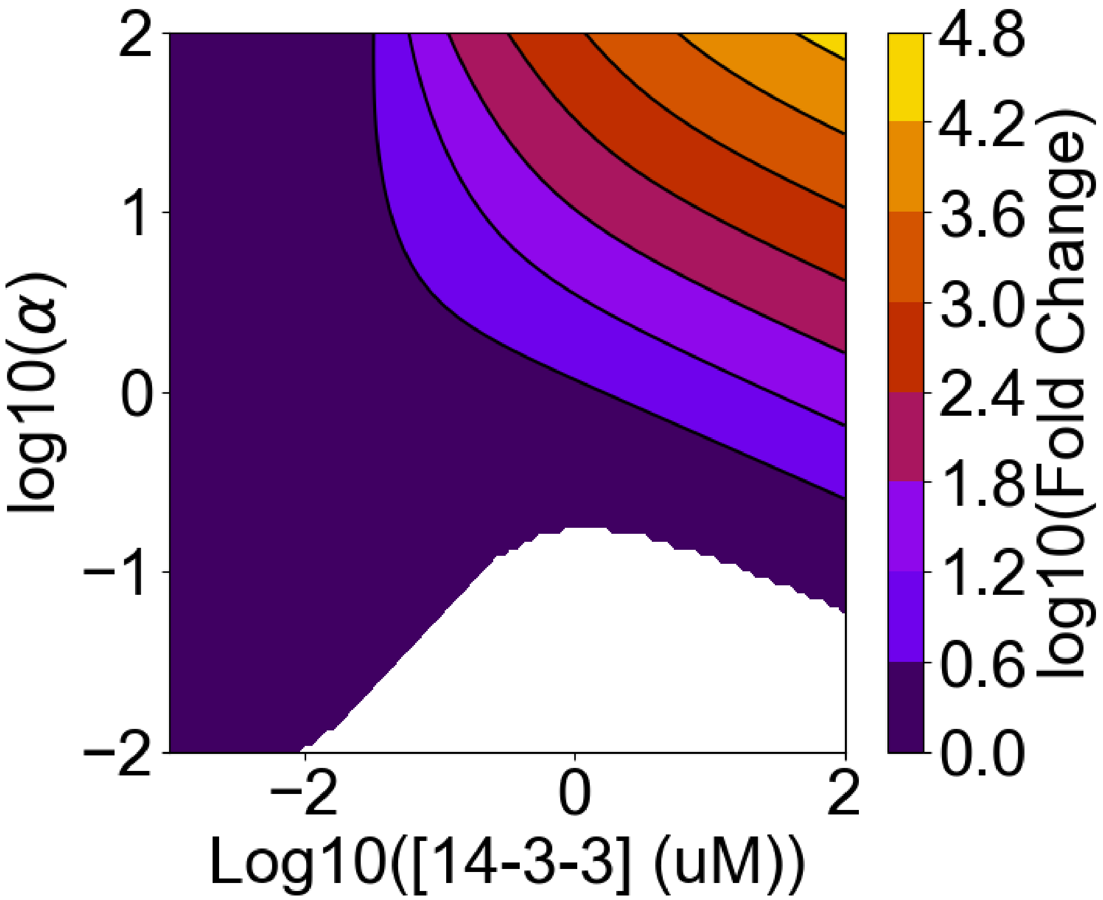


### 0.13 Figure S2D

[27]:
```
# Log10(% RAF without drug)
font = {'family' : 'Arial',

        'size'   : 30}
plt.rc('font', **font)
plt.figure(figsize=(7,6))
colormaptype='viridis'
fileid="ActivatingRange"
levels=[i/10 for i in range(-20,110,20)]
cp = plt.contourf(X,Y,zr,levels,cmap=colormaptype)
plt.contour(cp,colors='k',linewidths=1.5)
plt.colorbar(cp,label='log10(Activating Range)')

# plt.plot(X[0],[np.log10(0.5)]*len(X[0]),'k-',label=r'$\alpha$=0.5')
# plt.plot([np.log10(paramsbase['KS']/paramsbase['KA'])]*len(Y[:,0]),Y[:
 ↪,0],'b--',label='[14-3-3]=KS/KA',linewidth=3)
```

```
# plt.plot([np.log10(paramsbase['KS'])]*len(Y[:,0]),Y[:,0],'b-.
↪',label='[14-3-3]=KS',linewidth=3)

# plt.legend(bbox_to_anchor=(1.5,0.5))

plt.ylabel(r'log10($\alpha$)')
plt.xlabel('Log10([14-3-3] (uM))')
figname="DAK_1433_"+fileid+"_STOTvsAlpha.pdf"
# plt.savefig(figname,dpi=300)
plt.show()
```

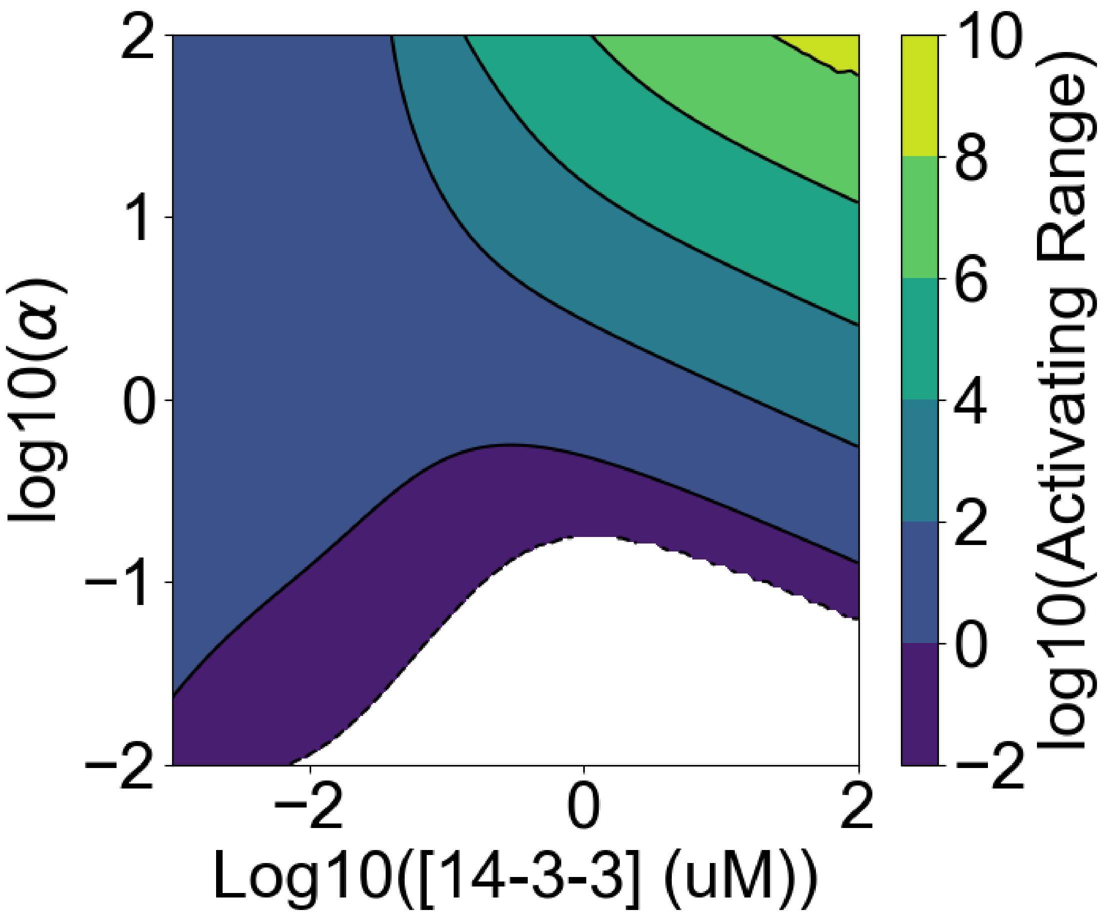

Supplement: S1 Data — Supplementary files that include the code required to analyze and evaluate the models and to reproduce all of the results presented in this study. (ZIP) [file pcbi.1013297.s005.zip › Supplementary Data Mendiratta RAF 14-3-3/Code/RAF_1433_Roles/Generate_Plots_14-3-3DualRoles_Figures2 and S1 and S2.pdf]
