# Supplementary material for: Mathematical modeling suggests 14-3-3 proteins modulate RAF paradoxical activation
Source: PLoS Comput Biol. 2025 Aug 1;21(8):e1013297. doi: 10.1371/journal.pcbi.1013297 (PMC12407542; doi:10.1371/journal.pcbi.1013297)
Supplement: S1 Data — Supplementary files that include the code required to analyze and evaluate the models and to reproduce all of the results presented in this study. (ZIP) [file pcbi.1013297.s005.zip › Supplementary Data Mendiratta RAF 14-3-3/Code/RAF_1433_Roles/Generate_Plots_14-3-3DSmodel_Figures2 and S1.pdf]

Generate_Plots_14-3-3DSmodel_Figures2 and S1

January 26, 2025

[1]:
```
#Import Libraries
import numpy as np
import scipy.optimize as so
import matplotlib.pyplot as plt
import os
```

0.1 Model Definitions: DS model

[2]:
```
DTOTMIN=10**-5 #micro-M = 100 pM
def checkpos(x,f=None,accept=None):
    """Returns 'True' if all elements in a list are positive numbers greater␣
↪than 10^-7 else False"""
    flag=True
    if type(x) is dict:
        x=list(x.values())
    for elem in x:
        if elem<=10**-7:
            flag=False
    return flag
def arrcompare(x1,x2):
    """returns the relative difference between corresponding values in lists x1␣
↪and x2. """
    try:
        res=[]
        for i in range(len(x1)):
            res=res+[(x1[i]-x2[i])/x1[i]]
        return res
    except: return 'ERROR'
def rafeqn(dr,A,params):
    raftoteqn=params['RAFTOT']-(A*(1 + params['KA'] + (2*A)/params['Kdim']) +␣
↪2*params['STOT'] + (A*(4*A + params['Kdim'])*dr + 2*A**2*dr**2 -␣
↪(2*params['Kdim']**2*params['KS']*params['STOT'])/(A**2 +␣
↪params['Kdim']*params['KS'] + A**2*dr*(2 + dr)))/params['Kdim'])
    return raftoteqn
def solA(dr,params):
    toteq=lambda A:rafeqn(dr,A,params) # initialize total equations with␣
↪parameters
```

```
    resas=so.brentq(toteq,0.,params['RAFTOT']) # solve the RAFTOT equation for values of unbound raf protomers (A)
    return resas
def actkin(dr,params):
    """This function inputs dr=unbound-drug/Kd and parameter set to output the active kinase in proportion to total raf kinase."""
    try:
        if dr>=0. and checkpos(list(params.values())):
            A=solA(dr,params)
            ak=(2*A**2*(1 + dr)*(A**2 + params['Kdim']*(params['KS'] + params['STOT']) + 2*A**2*dr + A**2*dr**2))/(params['Kdim']*params['RAFTOT']*(A**2 + params['Kdim']*params['KS'] + 2*A**2*dr + A**2*dr**2))
            return ak
    except:
        print("ERROR actkin:",dr,params)
        return 0
def dr2DTOT(dr,params):
    """Inputs unbound drug concentration alongwith a dictionary of parameters to return the total drug concentration"""
    try:
        A=solA(dr,params)
        return dr*(A + params['Kd'] + (2*A**2*(1 + dr)*(params['KS'] + (params['Kdim']*params['KS']*params['STOT'])/(A**2 + params['Kdim']*params['KS'] + A**2*dr*(2 + dr))))/(params['Kdim']*params['KS']))
    except:
        print("ERROR: d2DTOT: ",dr,params)
def DTOT2dr(DTOT,params):
    """Numerically solves the inverse function dtot2DTOT to convert input total drug concentration and parameters into unbound drug conccentration"""
    try:
        if DTOT>DTOTMIN and checkpos(list(params.values())):
            objfn=lambda dr: dr2DTOT(dr,params)-DTOT
            drmax=DTOT/params['Kd'] # d is always smaller than DTOT
            return so.brentq(objfn,1.*10**-12,drmax)
        else:
            return 0
    except:
        print("ERROR DTOT2dr:",DTOT,params)
def DTOT2AK(DTOT,params):
#     print("DTOT,params",DTOT,params)
    if DTOT is None:
        dr=0.
        DTOT=0.
    try:
```

```
        if checkpos(list(params.values())) is False:
            return 10**10
        elif DTOT>=DTOTMIN:
            dr=DTOT2dr(DTOT,params)
        else:
            dr=0.
        return actkin(dr,params)
    except:
        print("ERROR DTOT2AK:",DTOT,params)
def DTOT2AKnorm(DTOT,params):
    """This function inputs total drug values (in uM or same units as Kd in␣
 ↪params), paramteres to output active RAF protomers normalized to no-drug"""
    return DTOT2AK(DTOT,params)/actkin(0,params)


def solrange(params):
    """This function inputs a set of absolute parameters and finds the solution␣
 ↪for total drug concentration corresponding to maxima, maximal fold change␣
 ↪and total drug concentration at which the drug becomes an inhibitor␣
 ↪(activity levels equal drug free levels). Drug concentrations are given in␣
 ↪micro molar."""
    mindbound=0.0001
    one=1-mindbound
    kinref=actkin(0.,params)
    objfn=lambda dr:kinref/actkin(dr,params)
    try:
        res=so.minimize_scalar(objfn,bounds=(mindbound,10.**5))
        if (res.x<1) or (res.success is False):
            res=so.minimize_scalar(objfn,bounds=(mindbound,1),method='Bounded')
        drroot=res.x
    except:# This exception handles cases that are essentially pure-inhibitors␣
 ↪(hence minimization function fails)
        if objfn(mindbound)>one:
            drroot=0.
    kinmax=actkin(drroot,params)
    foldchange=kinmax/kinref
    if foldchange>one:
        Droot=dr2DTOT(drroot,params)
        AKref=DTOT2AK(0.,params)
        objfn1=lambda Dtot:(DTOT2AK(Dtot,params)-AKref)/AKref
        try:
            width=so.brentq(objfn1,Droot,10**6.) # to handle cases when the␣
 ↪prameter values are smaller
        except:
            width=so.brentq(objfn1,Droot,10**9.) # to handle very high values␣
 ↪of STOT and KA
        if Droot>10**-12:
```

```
            return Droot,foldchange,width
        else:
            return 0,0,0
    else:
        return 0,0,0

params0={'KA':10.,'Kd':0.1,'Kdim':0.1,'RAFTOT':0.04,'KS':0.02,'STOT':1.}#␣
↪dimensionful rates are in micro-Molar and sec
rafeqn(1.1,0.001,params0),solA(1.1,params0),actkin(1.1,params0),dr2DTOT(1.
↪1,params0),DTOT2dr(0.1104115,params0)
```

[2]:
```
(0.023411502655644477,
 0.0019308727379947562,
 0.1980528555982739,
 0.1208382856581183,
 1.0034845713266505)
```

[3]:
```
# Define total dimer functions
def DTOT2Dimers(DTOT,params):
    try:
        if checkpos(list(params.values())) is False:
            return 10**10
        elif DTOT>=DTOTMIN:
            dr=DTOT2dr(DTOT,params)
        else:
            dr=0.
        return actkin(dr,params)*(1+dr)/2.
    except:
        print("ERROR DTOT2Dimers:",DTOT,params)
def DTOT2DimersNorm(DTOT,params):
    """This function inputs total drug values (in uM or same units as Kd in␣
↪params), paramteres to output active RAF protomers normalized to no-drug"""
    return DTOT2Dimers(DTOT,params)*2./actkin(0,params)
```

0.2 Solve Model

0.3 Contour Plots : Total drug

[4]:
```
%%time
paramsbase=dict(params0)# values in micro-molar
npts=30000 # total number of points to plot
npts=int(np.sqrt(npts)) # square root of the number of points to put on a␣
↪square grid
xlist = np.linspace(-1.0, 2.0,npts)
ylist = np.linspace(-3.0, 2.0,npts)
X, Y = np.meshgrid(xlist, ylist)
def callfnr(x1,y1):
```

```
    params1=dict(paramsbase)
    params1['RAFTOT']=10**y1
    params1['KA']=10**x1

    try:
        resarr=solrange(params1)
        if resarr[0]>10**-5:
            return [np.log10(resarr[1]),np.log10(resarr[2])]
        else:
            return [float('nan'),float('nan')]
    except:
        return [float('nan'),float('nan')]
zfc=[]
zr=[]
for itr in range(len(X)):
    zfc=zfc+[[]]
    zr=zr+[[]]
    for jtr in range(len(X[itr])):
        res=callfnr(X[itr][jtr],Y[itr][jtr])
        zfc[itr]=zfc[itr]+[res[0]]
        zr[itr]=zr[itr]+[res[1]]
```

```
CPU times: total: 2min 48s
Wall time: 3min 45s
```

0.4 Figure 2C

[11]:
```
font = {'family' : 'Arial',
        'size'   : 35}
plt.rc('font', **font)
plt.figure(figsize=(7,6))
colormaptype='viridis'
fileid="range"
minlevel=-1
levels=[minlevel]+[i/10 for i in range(10,60,10)]
cp = plt.contourf(X,Y,zr,levels=levels,cmap=colormaptype)
plt.contour(cp,colors='k',linewidths=1.5)
plt.colorbar(cp)
minlevel=0.002
lc=plt.contour(X,Y,zfc,colors='r',linewidths=5.
 ↪,levels=[minlevel],linestyles='solid')
minlevel=0.004
lc=plt.contour(X,Y,zfc,colors='r',linewidths=5.
 ↪,levels=[minlevel],linestyles='solid')

plt.xlabel('Log10(KA)')
plt.ylabel(r'Log10(RAF ($\mu$M))')
```

```
# figname="DAK_1433full_DTOT_"+fileid+"_RAFrvsKA.pdf"
# plt.savefig(figname,dpi=300)
plt.show()
```

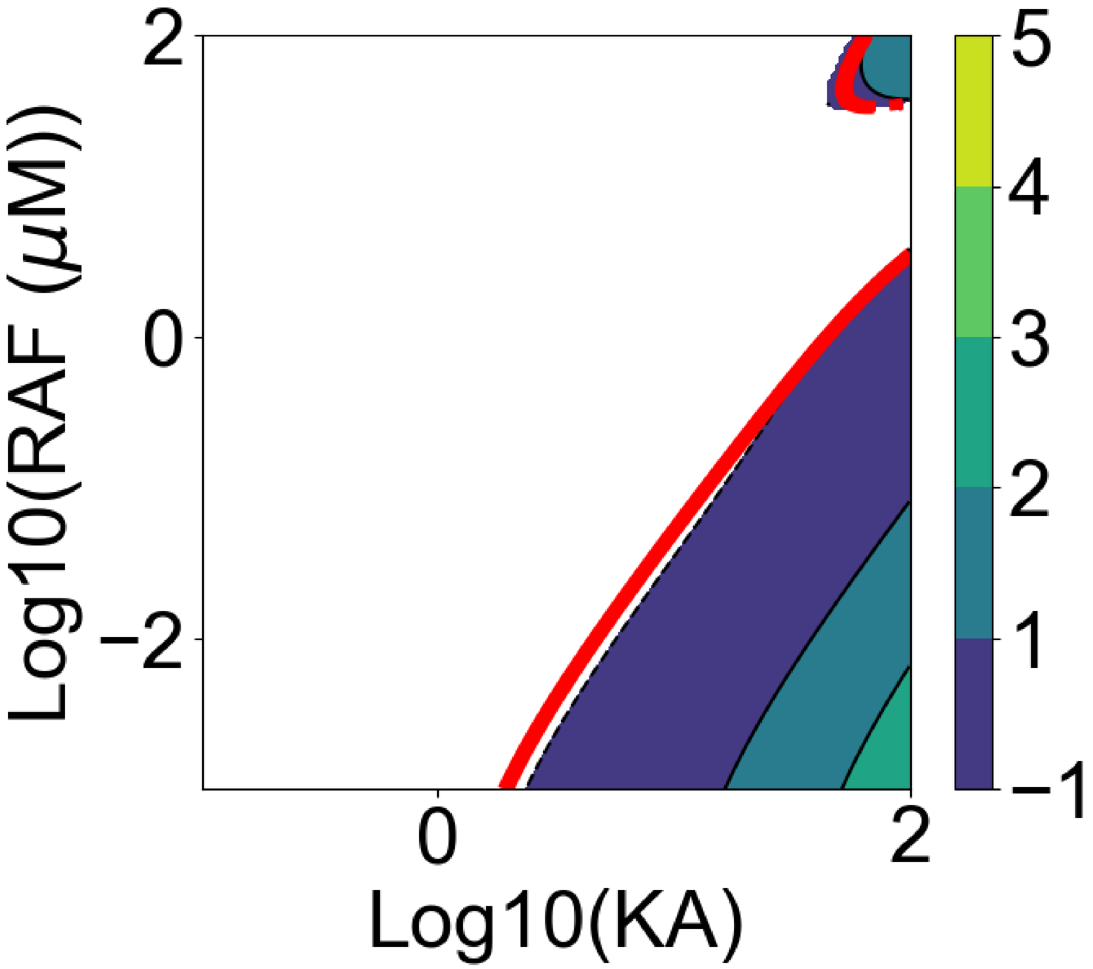

0.4.1 Figure S1E

[6]:
```
colormaptype='gnuplot'
fileid="foldchange"
plt.figure(figsize=(7,6))
minlevel=0.03
levels=[minlevel]+[i/10 for i in range(5,26,5)]
cp = plt.contourf(X,Y,zfc,levels=levels,cmap=colormaptype)
plt.contour(cp,colors='k',linewidths=1.5)
plt.colorbar(cp)
minlevel=0.022
lc=plt.contour(cp,colors='r',linewidths=4.,levels=[minlevel],linestyles='solid')
```

```
plt.xlabel('Log10(KA)')
plt.ylabel('Log10(Total RAF [uM])')
# figname="DAK_1433full_"+fileid+"_RAFrvsKA.pdf"
# plt.savefig(figname,dpi=300)
plt.show()
```

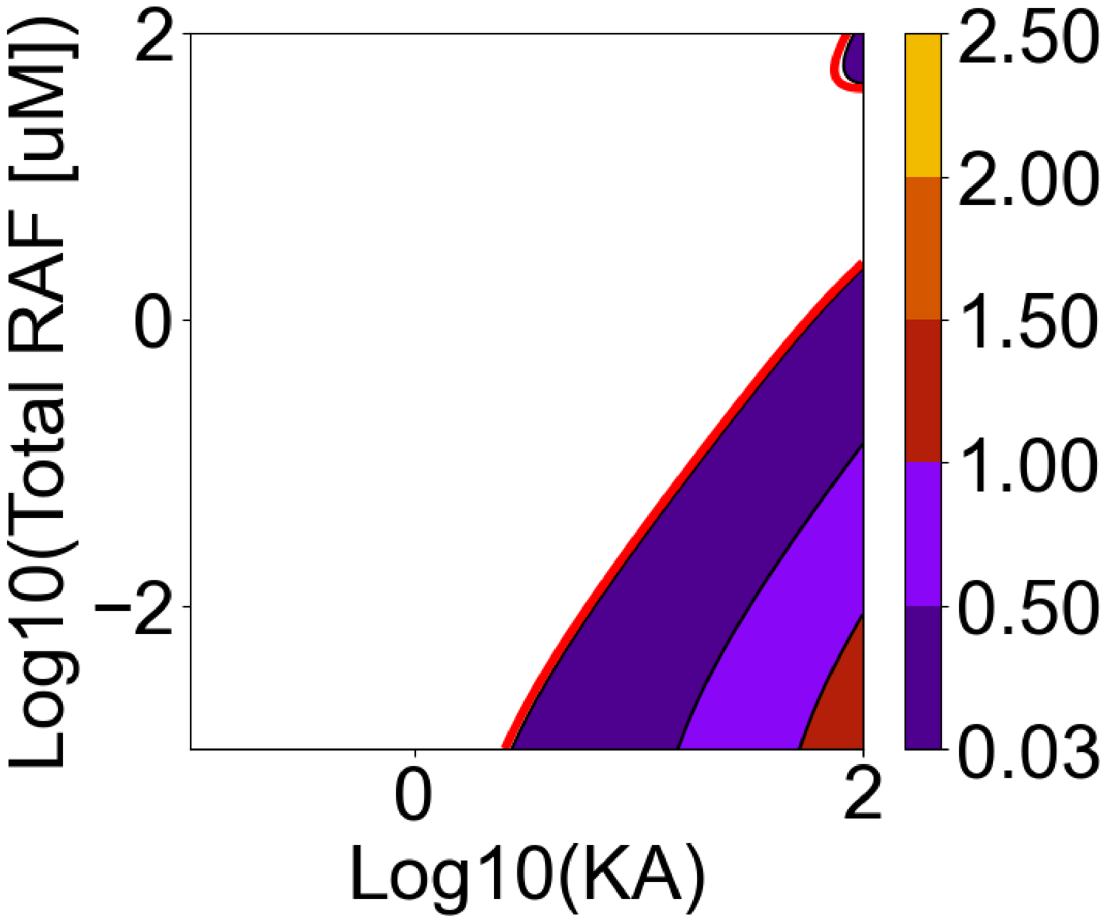

0.4.2 fix rdim and vary STOT

[12]:
```
%%time
paramsbase=dict(params0)# values in micro-molar
npts=10000 # total number of points to plot
npts=int(np.sqrt(npts)) # square root of the number of points to put on a␣
↪square grid
xlist = np.linspace(-1.0, 2.0,npts)
ylist = np.linspace(-3.0, 2.0,npts)
X, Y = np.meshgrid(xlist, ylist)
def callfnr(x1,y1):
    params1=dict(paramsbase)
```

```
    params1['STOT']=10**y1
    params1['KA']=10**x1

    try:
        resarr=solrange(params1)
        if resarr[0]>10**-5:
            return [np.log10(resarr[1]),np.log10(resarr[2])]
        else:
            return [float('nan'),float('nan')]
    except:
        return [float('nan'),float('nan')]
zfc=[]
zr=[]
for itr in range(len(X)):
    zfc=zfc+[[]]
    zr=zr+[[]]
    for jtr in range(len(X[itr])):
        res=callfnr(X[itr][jtr],Y[itr][jtr])
        zfc[itr]=zfc[itr]+[res[0]]
        zr[itr]=zr[itr]+[res[1]]
```

```
CPU times: total: 54 s
Wall time: 1min 41s
```

0.5 Figure 2C

[22]:
```
font = {'family' : 'Arial',
        'size'   : 35}
plt.rc('font', **font)
plt.figure(figsize=(7,6))
colormaptype='viridis'
fileid="range"
minlevel=-1.5
levels=[minlevel]+[i/10 for i in range(0,91,15)]
cp = plt.contourf(X,Y,zr,levels,cmap=colormaptype)
plt.contour(cp,colors='k',linewidths=1.5)
plt.colorbar(cp)
minlevel=0.0007
lc=plt.contour(X,Y,zfc,colors='r',linewidths=5.
 ↪,levels=[minlevel],linestyles='solid')
plt.xlabel('Log10(KA)')
plt.ylabel('Log10([14-3-3] (uM))')
figname="DAK_1433full_DTOT_"+fileid+"_STOTvsKA.pdf"
# plt.savefig(figname,dpi=300)
plt.show()
```

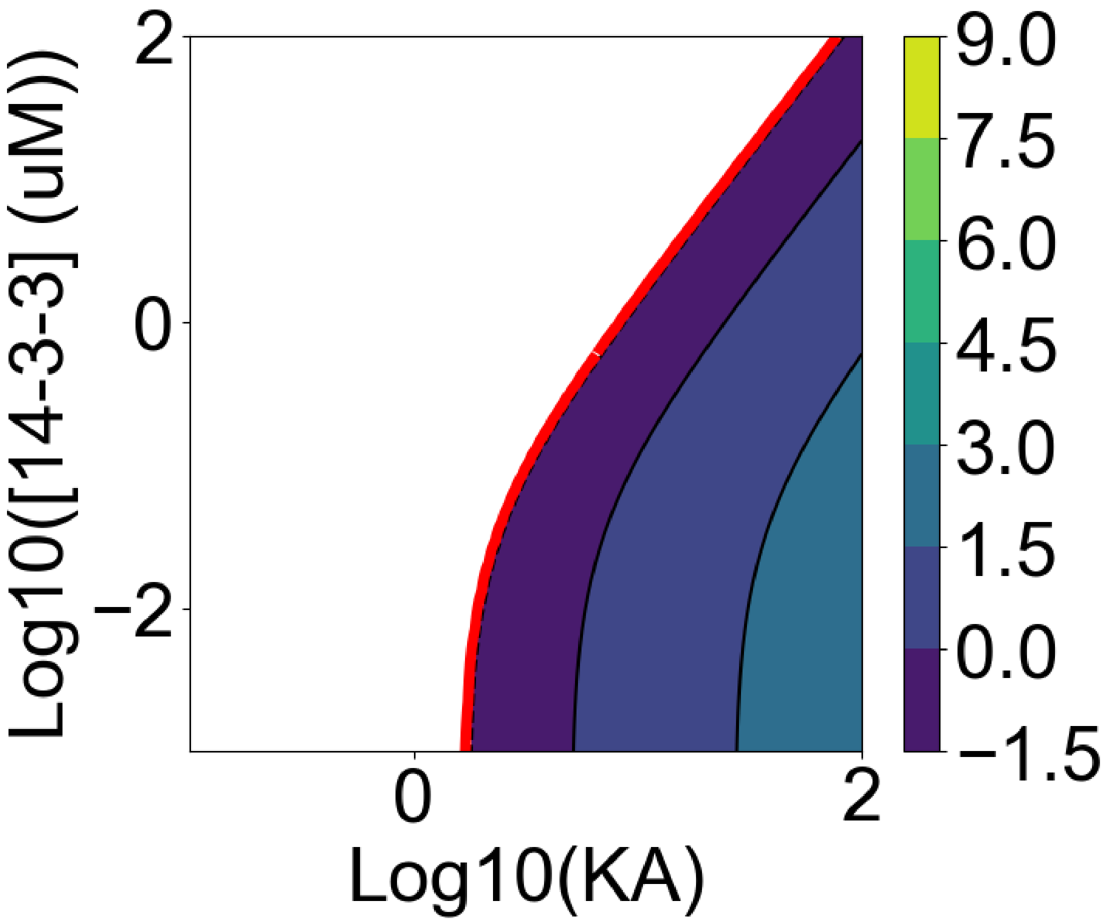

0.5.1 Figure S1F

[21]:
```
colormaptype='gnuplot'
fileid="foldchange"
plt.figure(figsize=(7,6))
minlevel=0
levels=[minlevel]+[i/10 for i in range(10,41,5)]
cp = plt.contourf(X,Y,zfc,levels,cmap=colormaptype)
plt.contour(cp,colors='k',linewidths=1.5)
plt.colorbar(cp)
minlevel=0.0007
lc=plt.contour(cp,colors='r',linewidths=4.,levels=[minlevel],linestyles='solid')
# cp2 = plt.contourf(X1,Y1,Z1,levels)
plt.xlabel('Log10(KA)')
plt.ylabel('Log10([14-3-3] (uM))')
# figname="DAK_stableAI_DTOT_"+fileid+"_STOTvsKA.pdf"
plt.savefig(figname,dpi=300)
plt.show()
```

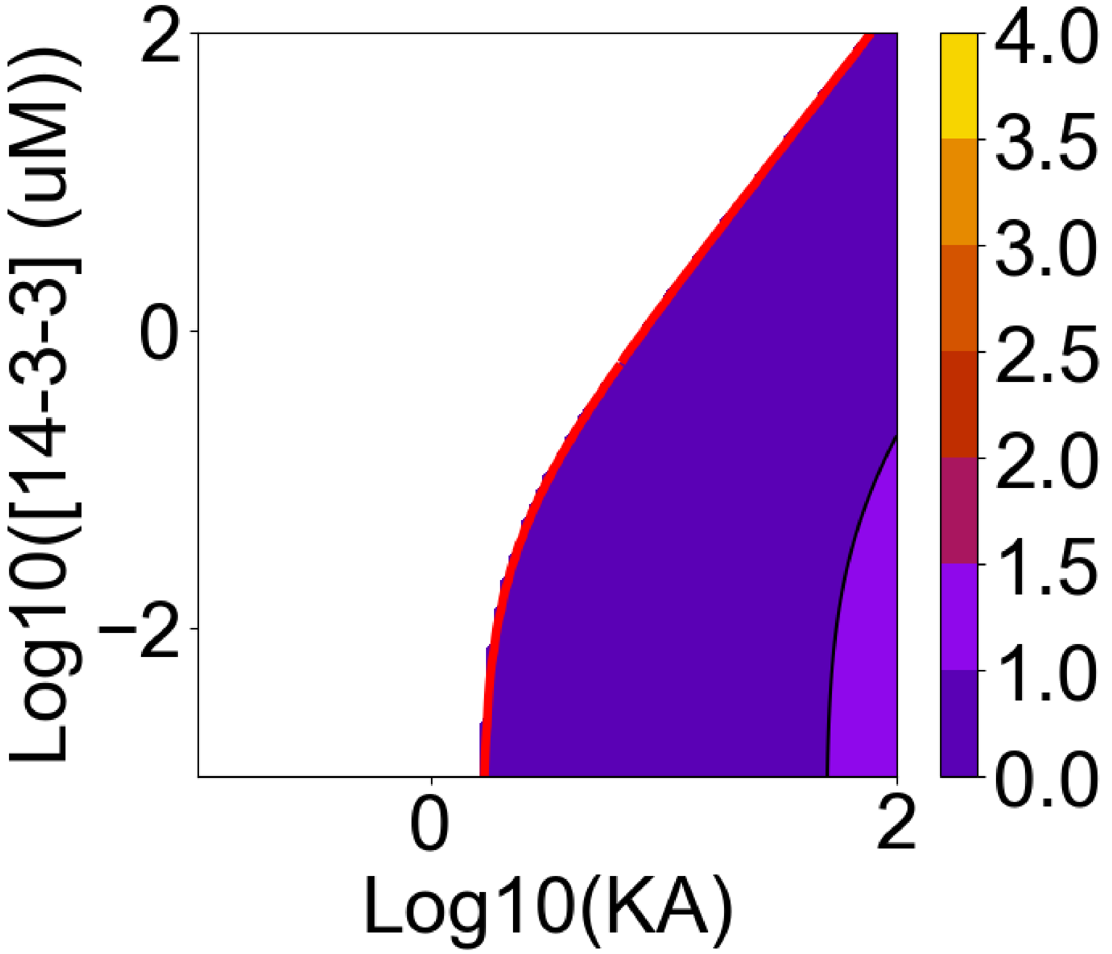

2
0
-2
Log10([14-3-3] (uM))
0
2
Log10(KA)
4.0
3.5
3.0
2.5
2.0
1.5
1.0
0.0

[ ]:
