# Supplementary figures and images for: Mathematical modeling suggests 14-3-3 proteins modulate RAF paradoxical activation
Source: PLoS Comput Biol. 2025 Aug 1;21(8):e1013297. doi: 10.1371/journal.pcbi.1013297 (PMC12407542; doi:10.1371/journal.pcbi.1013297)

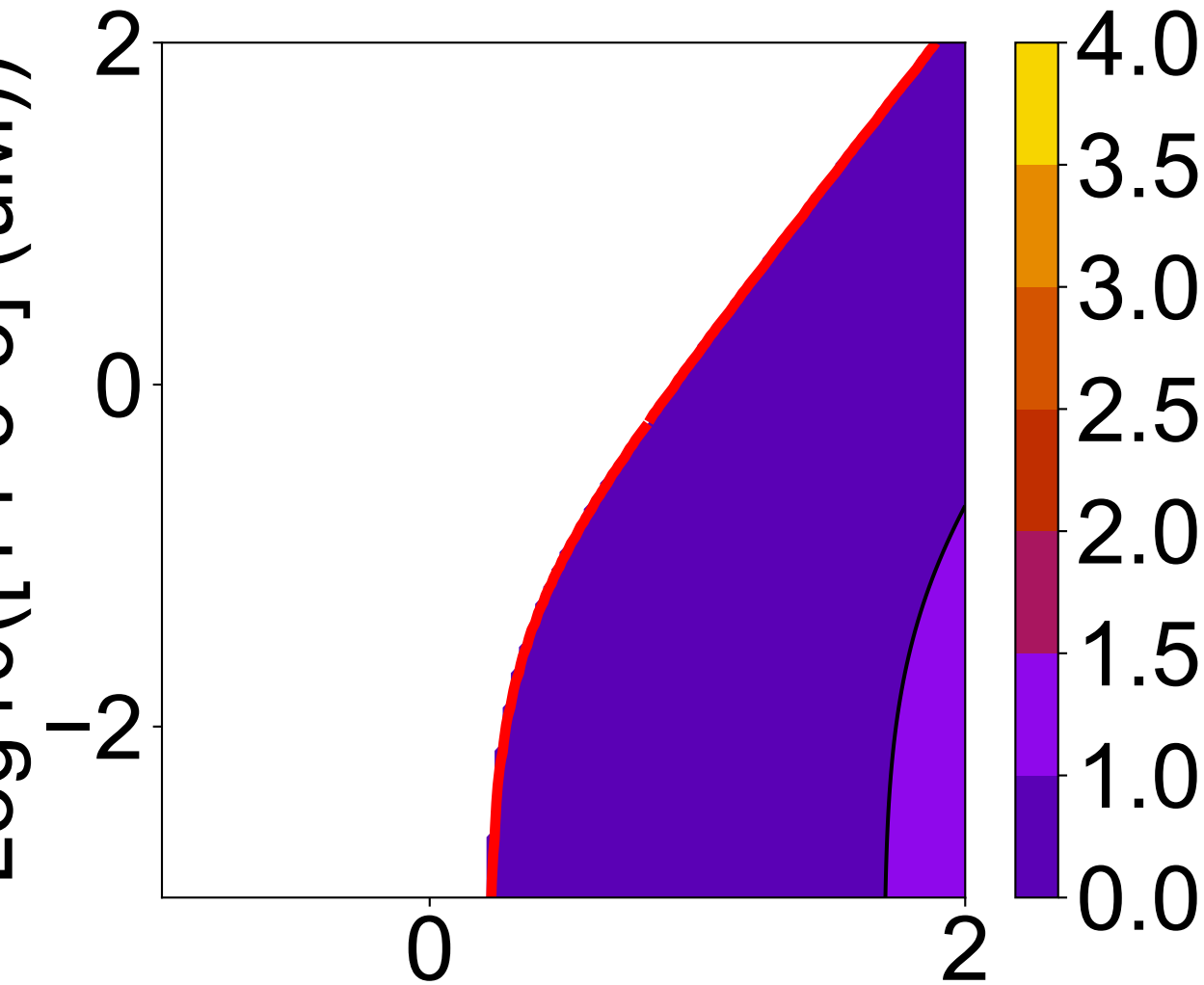

Supplement: S1 Data — Supplementary files that include the code required to analyze and evaluate the models and to reproduce all of the results presented in this study. (ZIP) [file pcbi.1013297.s005.zip › Supplementary Data Mendiratta RAF 14-3-3/Code/RAF_1433_Roles/DAK_1433full_DTOT_range_STOTvsKA.pdf]
